# Different routes for the construction of biologically active diversely functionalized bicyclo[3.3.1]nonanes: an exploration of new perspectives for anticancer chemotherapeutics

**DOI:** 10.1039/d3ra02003g

**Published:** 2023-07-25

**Authors:** Nilmadhab Roy, Rishav Das, Rupankar Paira, Priyankar Paira

**Affiliations:** a Department of Chemistry, School of Advanced Sciences, Vellore Institute of Technology Vellore 632014 Tamilnadu India priyankar.paira@vit.ac.in; b Department of Chemistry, Maharaja Manindra Chandra College 20 Ramkanto Bose Street Kolkata 700 003 India rupankarpaira@gmail.com

## Abstract

Cancer is the second most high-morbidity disease throughout the world. From ancient days, natural products have been known to possess several biological activities, and research on natural products is one of the most enticing areas where scientists are engrossed in the extraction of valuable compounds from various plants to isolate many life-saving medicines, along with their other applications. It has been noticed that the bicyclo[3.3.1]nonane moiety is predominant in most biologically active natural products owing to its exceptional characteristics compared to others. Many derivatives of bicyclo[3.3.1]nonane are attractive to researchers for use in asymmetric catalysis or as potent anticancer entities along with their successful applications as ion receptors, metallocycles, and molecular tweezers. Therefore, this review article discusses several miscellaneous synthetic routes for the construction of bicyclo[3.3.1]nonanes and their heteroanalogues in association with the delineation of their anticancer activities with few selective compounds.

## Abstract 2

Cancer is the second most high-morbidity disease throughout the world.

## Graphical abstract

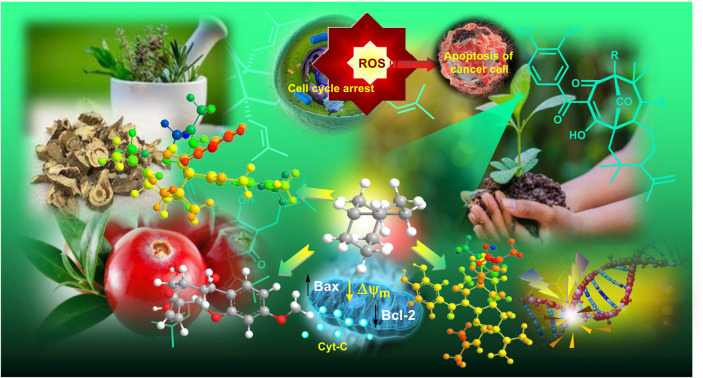

## 1. Introduction

Cleft-shaped entities have long been used as essential chemical tools in molecular recognition studies.^1^ The real development in this area started during the late 1970s with the discovery of Kagan's ether (2)^2^ and some other structurally diverse bicyclo[3.3.1]nonane derivatives (3–5),^3^ along with their successful application as ion receptors, metallocycles, and molecular tweezers.^4^ The first such molecule, Troger's base (1),^5^ was developed as early as the late 1880s, yet it continues to fascinate scientists by its uniqueness as an asymmetric catalyst^6^ as well as a DNA intercalator^7^ or enzyme inhibitor.^8^ Even 125 years after its discovery, the science of Troger's base is progressing at a fast pace.^9^

Apart from these synthetic bicyclo[3.3.1]nonanes, this important core moiety is quite plentiful among several bioactive natural products. For example, alkaloids such as isariotin A, nankakurine A, adaline, huperzine A, and lycodine^10^ and terpenoids such as upial, spirovibsanin, trifarienols A and B,^11^ and swietenine^12^ incorporate this one-carbon-bridged cyclooctane moiety in their structural framework. Some naturally occurring bioactive benzophenones^13^ and cytostatic metabolites such as gymnastatins F, G, and Q along with their diacetates and triacetates^14^ also contain this core unit, but bicyclo[3.3.1]nonanes are most abundant among the polyprenylated acylphloroglucinols (PPAPs), for example, clusianone, garsubellin A, aristophenone A, plukenetione, hyperforin, nemorosone, guttiferone A, hypersampsone F, and papuaforin A.^15^ Recent developments in synthetic chemistry have witnessed the successful application of appropriately modified bicyclo[3.3.1]nonane units as useful precursors for accessing more complex targets (both synthetic and natural products).^15^ It has also been observed that indole alkaloids containing azabicyclo[3.3.1]nonane architechture play a crucial role as anticancer, antimalarial, antiinflammatory, antiamebic, antileishmanial, antituberculosis, and antiarrhythmic drug candidates. Therefore, this skeleton always acquires a noticeable position in the history of natural products as they possess structural resemblance with the essential amino acid tryptophan as well as its related metabolite, the neurotransmitter serotonin. In particular, these indole alkaloids have gained special attention as they contain azacyclic and tryptophan-derived substructures that are widely regarded as “privileged structures” or efficient substructures suitable for binding to various types of protein receptors with higher responsiveness. Some important indole alkaloids are sarpagine, ajmaline, and macroline, which consist one of the major groups of structurally related indole natural products. These types of new alkaloids are now being isolated with a greater rate from various plant sources throughout the world because of their remarkable biological activity. A book chapter based on “Sarpagine and Related Alkaloids” written by O. A. Namjoshi and J. M. Cook has revealed the synthetic routes as well as their biological evaluation in a precise manner.^16^

Thus, the synthesis of diversely-functionalized bicyclo[3.3.1]nonanes has gained immense importance in recent times, resulting in the discovery of a large number of synthetic approaches toward this important core moiety. It is worth mentioning that a book chapter by Buchanan^17^ and a review by Peters^18*a*,*c*^ on this important core moiety have appeared during the 1970s. A short review, particularly on the asymmetric synthesis of bicyclo[3.3.1]nonanes, appeared during the beginning of this century.^18*b*,*d*,*e*^ However, this review also reveals the structurally-related bicyclo[3.2.1] octanes^19^ and bicyclo[4.2.1]nonanes,^20^ which cover further expansions and developments in the area of both asymmetric (chiral) or nonasymmetric (achiral) bicyclo[3.3.1]nonanes and their heteroanalogues. The chemistry of Troger's base and its analogues is, however, not included in this article as it is already very well-reviewed by several authors.^18*f*–*j*^ Hence, in this review article, we have aspired to assemble the recent chemistry of bicyclo[3.3.1]nonanes and their heteroanalogues, both as a synthetic target as well as a synthetic intermediate, discussing its importance in anticancer therapy to unveil a fruitful pathway for the future design of anticancer chemotherapeutics.

## 2. Conformational features

The chemistry of bicyclo[3.3.1]nonanes is very much dependent on their conformational properties. Thus, great efforts have been dedicated during the last four decades on the broad investigations of their conformational features. Unlike the structurally related bicyclo[3.2.1]octanes, the above hydrocarbons can exist in three possible conformations, namely, a *C*_2v_-symmetric twin chair (CC, 30a), *C*_s_-symmetric boat chair (BC, 30b), and *C*_2_-symmetric twisted twin boat (BB, 30c).^21–23^ The destabilizing steric factors present in the BB conformer ruled out the possibility of its existence in a detectable amount. Besides, the high energy difference (Δ*G*° = 2.3 kcal mol^−1^) between the most stable CC and the comparatively unstable BC meant that the latter went virtually undetected in NMR even at a low temperature range from −100° to −170 °C without any significant broadening of the NMR signal of the major conformer, as corroborated by statistical calculations.^24–27^ However, at very high temperature (400 °C) 25% population of BC was established by electron diffraction investigations.^24^ The corresponding 9-keto analogue, however, presents a different scenario. Although the twisted twin boat form 31c is as unpopulated as 30c,^28^ the equilibrium between BC (31b) and CC (31a) is less inclined toward CC, with much lower energy barrier (0.95 kcal mol^−1^) than in 30. Theoretical calculations by several research groups and the lanthanide-induced NMR shift (LIS) investigation determined a 0.9–2.4% population of the BC conformer at −165 °C, as indicated by the significant broadening of the NMR line width of the major isomer.^25*a*,28–32^

The CC conformer is, however, not always the predominant entity in all bicyclo[3.3.1]nonanes. Appropriate substitutions in the carbocyclic rings give rise to rather interesting features. For example, 2,4,6,8-tetraaryl-3,7-diazabicyclo[3.3.1]nonanes 32a,b–35a,b always adopt the BC conformation to avoid the 1,3-diaxial steric repulsion between the aryl groups and lone pair–lone pair (lp–lp) repulsion between the nitrogens. However, *N*-nitrosation imposes more sp^2^ character on the nitrogens, which lowers the lp–lp repulsion in the CC conformation. Also, this introduces a rather more dominating repulsive factor, the allylic A (1, 3) strain, between the nitroso groups and the neighboring α-aryl groups in the BC conformation, making the CC conformer the chief one.^33–38^ The conformational behavior of 3-borabicyclo[3.3.1]nonane 36 is rather more fascinating. The pπ–pπ backbonding between the filled p-orbital of oxygen and the vacant p-orbital of boron raises the bond order and restricts rotation around the B–O bond. Thus, repulsive steric factors favor the CC conformer. However, as the temperature increases, the rotation around both B–O and C–phenyl bonds is facilitated, thereby increasing the steric requirements of both methoxy and phenyl groups, resulting in a rise in the BC population, as corroborated by NMR studies. Moreover, when 36 was allowed to form a chelate with pyridine-d5 or dibenzoylmethane (complex 37, 38), boron gets tetracoordinated and its steric requirement rises further, making BC the major conformer.^39–41^ Simple 9-BBN (39a,b), however, always prefers the CC conformer and even takes part in the palladium-mediated arylation reactions through intermediate 40, also in the CC conformation.^41^

This kind of preference for the BC conformer is also very common in heavy atom-substituted bicyclo[3.3.1]nonanes. For example, 9-oxa-3,7-dithiabicyclo[3.3.1]nonane (41) as well as 9-oxa-3-selena-7-thiabicyclo[3.3.1]nonane (42) are rich in their BC conformers, mainly due to the lp–lp repulsion of heavy atoms (such as Se and S *etc.*) present at 3 and 7 positions in the CC conformer. Such phenomena are commonly known as the “Hockey Sticks” effect.^42–48^

However, 3,7-dithia-1,5-diazabicyclo[3.3.1]nonane (43) shows no such preference for the BC conformer. The presence of two additional stabilizing LP-N–C–S stereoelectronic interactions in this case favors the CC conformer over BC, as justified by QTAIM analysis.^49–52^ In some cases, the CC conformer is still preferred (*e.g.*, in 44), even in the absence of such stabilizing factors. The stabilization of the CC conformer through improper C–H⋯S hydrogen bonding between S and C7–H_ax_ is supported by the theoretically calculated 1.61 kcal mol^−1^ energy lowering due to *n*(*S*) → *σ* × (C–H) overlap interaction. Such an interaction is the strongest in 44 compared to the corresponding unsubstituted bicyclo[3.3.1]nonane (30), aza-anagues (45, 46), and oxa-analogues (47, 48).^53,54^ Protonated 3-aza-bicyclo[3.3.1]nonane (49), however, reveals a different scenario. It forms a dihydrogen bond with significant covalent character due to the close proximity (1.78 Å) between –CH and –HN^+^ hydrogens, resulting in an energy lowering of 4.24 kcal mol^−1^.^54^ Apart from these improper H-bonds, proper hydrogen bonding is also common in appropriately substituted bicyclo[3.3.1]nonanes such as 3-azabicyclo[3.3.1]nonane-2,4-dione and is responsible for its polymorphic property.^55^

Another interesting conformational feature of bicyclo[3.3.1]nonane arises during the substitution at the bridgehead-methylene group. Unlike the 7,7-diaryl norboranes, which exist as an inseparable mixture of atropisomers at ambient temperature due to the small rotational energy barrier around the C-aryl bonds,^56–58^ 9,9-diarylbicyclo[3.3.1]nonanes could exist in two separate atropisomeric forms, when properly substituted. For example, although the rotational energy barrier is small in 50 and it could not be isolated in its two isomeric forms, 51 and 52 have much higher energy barriers due to an orthogonal propeller-like orientation of the two aryl groups, which enables the separation of their atropisomers.^59^

## 3. Synthetic approaches

**Scheme 1:**
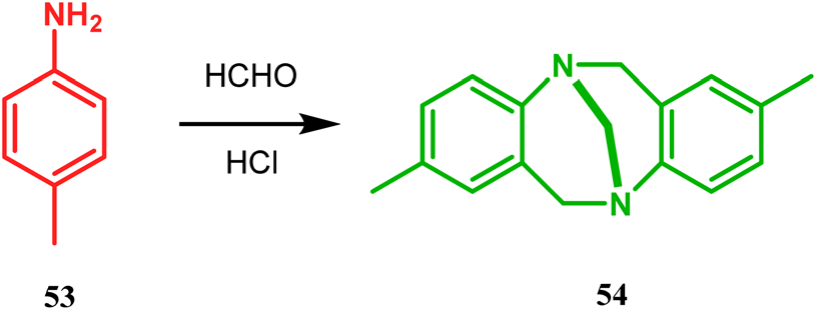
Synthesis of Troger's base.

Bicyclo[3.3.1]nonane first appeared in literature in the form of its aza-analogue, with the discovery of Troger's base (54) (Scheme 1) in 1887 by Julius Troger. He was able to synthesize it simply by reacting *p*-toluidine with formaldehyde in aqueous HCl. However, it took almost half a century to confirm its actual structure, when Spielman unveiled his work in 1935.^60*a*,*b*^ Within a decade, another fascinating feature was established when Prelog successfully separated both the enantiomers of 54 in 1944, showing that chirality can exist in atoms other than carbon. Rapid inversion in a chiral heteroatom center in other unstrained molecules had restricted their separation in isomeric forms. However, it can be stopped and the enantiomers could be separated by introducing conformational strain, as in Troger's base.^60*c*^ However, more practical syntheses of Troger's base and its analogues came into the literature during the end of the 20th century, apart from a few which were published during 1960s.^60*a*^

**Scheme 2:**
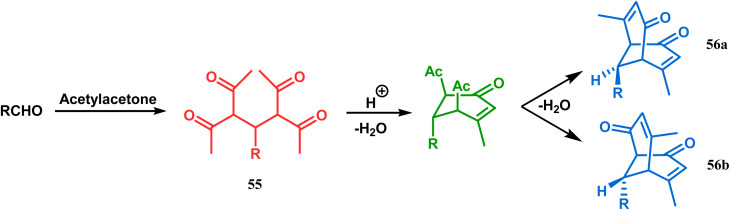
Condensation reaction between an aliphatic or aromatic aldehyde and acetylacetone to obtain bicyclo[3.3.1]nonane moieties.

**Scheme 3:**
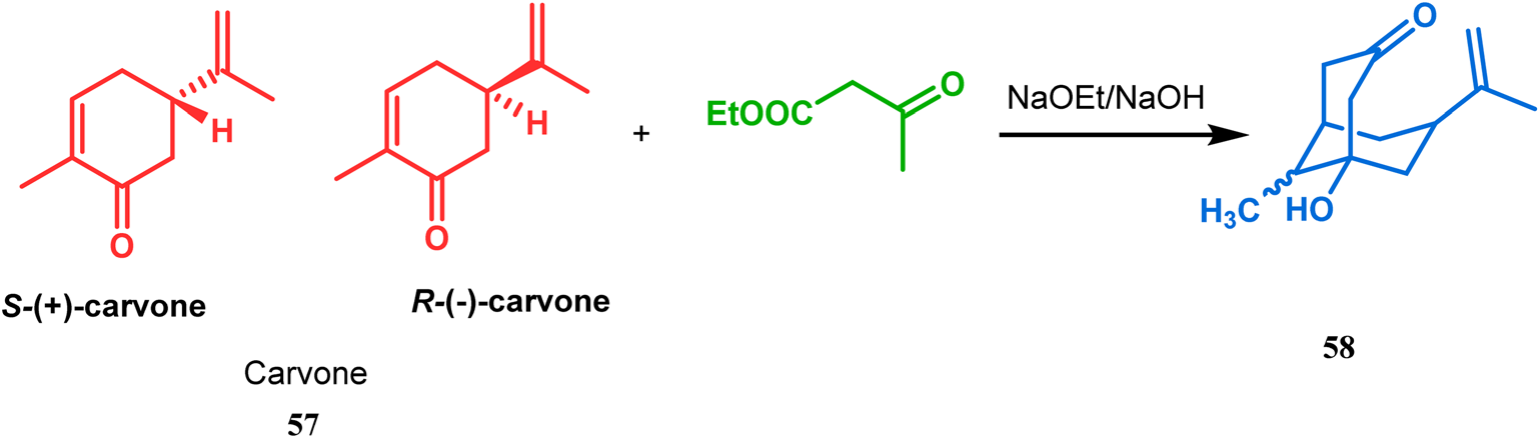
Formation of bicyclo[3.3.1]nonane moieties by the reaction between carvone and ethyl acetoacetate (EAA).

**Scheme 4:**
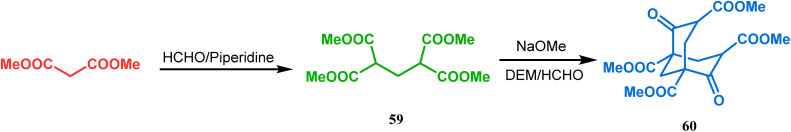
Synthesis of Meerwein's ester from dimethyl malonate and formaldehyde.

Although the structural uncertainties of Troger's base delayed the development of its chemistry, the progress in the chemistry of other bicyclo[3.3.1]nonanes proceeded smoothly from the very beginning of the 20th century. Condensation between aliphatic or aromatic aldehydes and acetylacetones, followed by acidic dehydration to produce 56 from 55 or 58 from carvone (57, a terpene) and ethyl acetoacetate, are some of those initiative routes to bicyclo[3.3.1]nonanes (Schemes 2 and 3).^61–67^ The synthesis of Meerwein's ester (60), the formerly used precursor for adamantane synthesis, was also achieved in this period, from dimethyl malonate and formaldehyde *via* intermediate 59 (Scheme 4).^68–70^ After these pioneering works on bicyclo[3.3.1]nonanes, the development of numerous synthetic methodologies to access this important moiety has been reported. These could be classified into three major groups: (a) intra- and intermolecular C–C bond formation, (b) intra- and intermolecular C–X bond formation, and (c) ring opening and ring expansion. The following sections of this review deal with all these.

### 3.1. Intra- and intermolecular C–C bond formation

#### 3.1.1. Aldol condensation

**Scheme 5:**
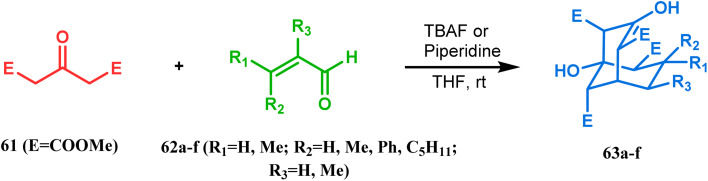
Formation of bicyclo[3.3.1] nonenols from the condensation reaction between dimethyl-1,3-acetonedicarboxylate and enals.

Base-promoted tandem Michael addition-intramolecular aldolizations are well documented in this category. For example, the condensation reaction between dimethyl-1,3-acetonedicarboxylate 61 and enals 62, promoted by piperidine or TBAF, gives high yields of bicyclo[3.3.1]nonenols 63 (Scheme 5).^71^ Another stereocontrolled route to such nonenols has been unveiled recently by Crowe, where 2-substituted cyclohex-2-enones were successfully condensed with some active methylene group containing esters and amides.

**Scheme 6:**
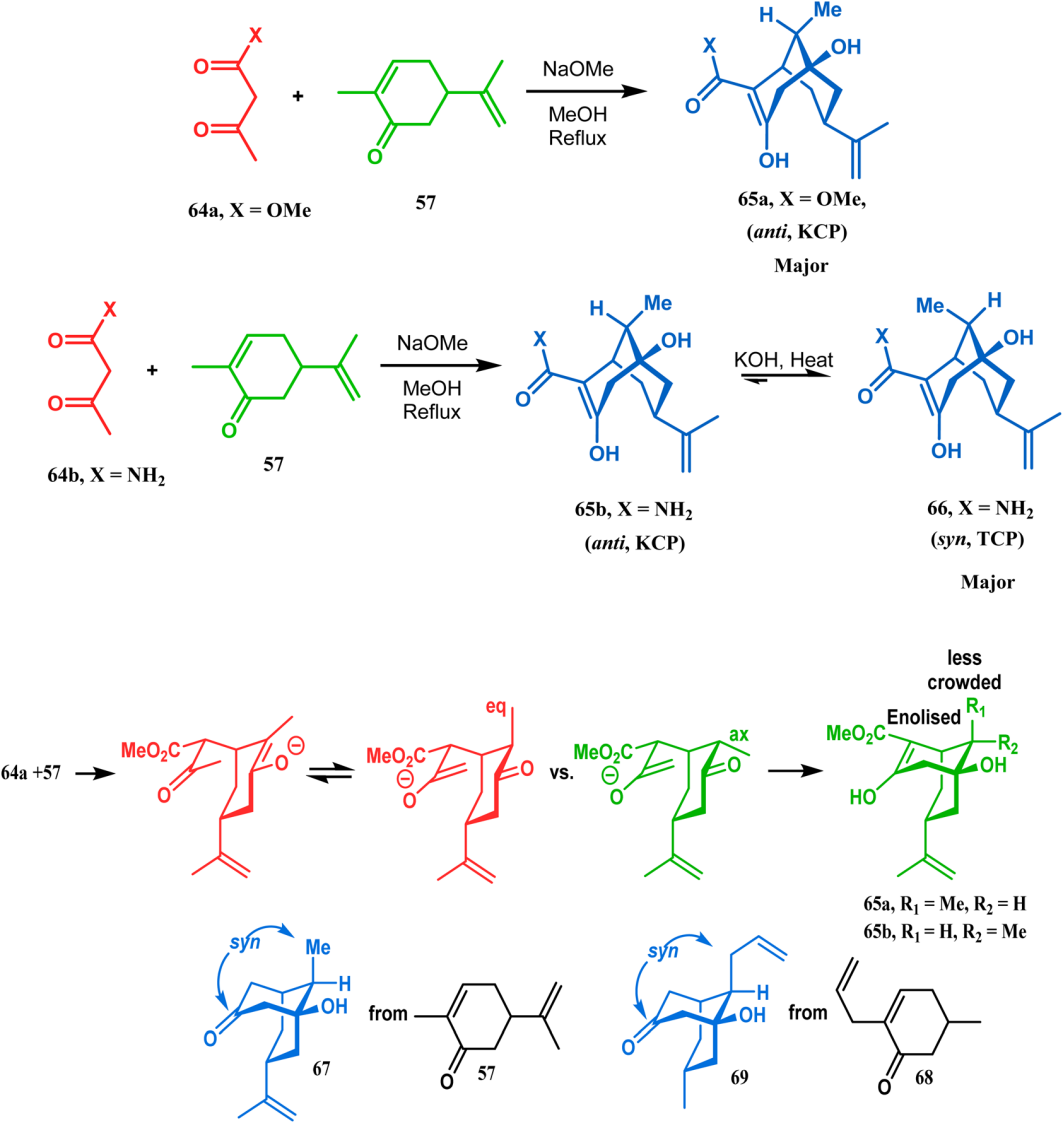
A synthetic route for the formation of stereocontrolled bicyclo[3.3.1] nonenols from carvone.

It was observed that the anti-product is always the kinetically-controlled product and the major one, irrespective of the nature of the starting materials. But the *syn* isomer 66, the thermodynamically-controlled one, becomes the major product (yield 80%) when carvone (57) is refluxed in methanolic KOH with amide 64b (Scheme 6).^72^

**Scheme 7:**
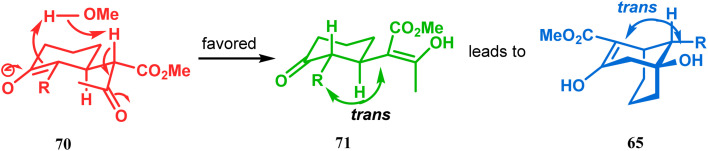
Formation of bicyclo[3.3.1] nonenols through intramolecular proton transfer.

However, these results were in direct conflict with those reported by Kraus and Theobold.^73–75^ Both the groups obtained the *syn* isomers (67 and 69) as the major one, supported by the fact that both these *syn* isomers should be formed from the lower energy conformer, with the methyl/allyl group in the equatorial position. Although a similar result should also be expected in the reaction between 64 and 57, there is also the possibility of an intramolecular proton transfer from 70 to give 71 (Scheme 7), similar to that proposed by Grossman,^76^ which could account for the unusual stereoselectivity obtained by Crowe.

**Scheme 8:**
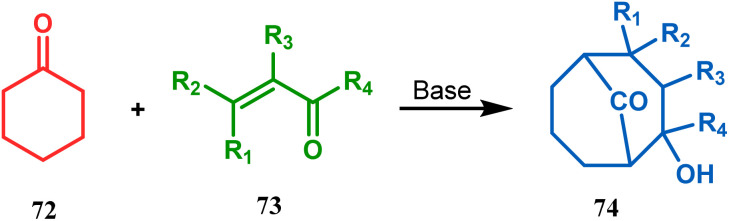
Synthesis of bicyclo[3.3.1]nonane from cyclohexanone and α,β-unsaturated aldehydes or ketones.

**Scheme 9:**
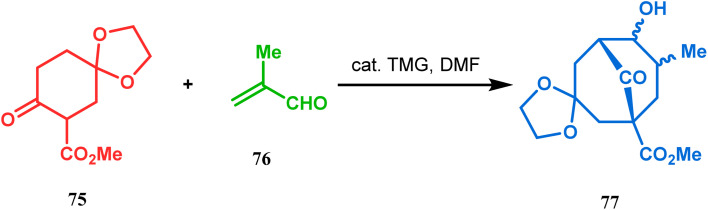
Annulation of β-keto ester with acrolein to from the bicyclo[3.3.1]nonane.

Cyclohexanones (72), when reacted in this manner with α,β-unsaturated aldehydes or ketones (73), yield a bicyclo[3.3.1]nonane (74) with a ketone functionality at the bridgehead position (Scheme 8).^71^ Similarly, Tückmantel and coworkers showed that β-keto ester (75) could also be annulated with acrolein (76) in the presence of a catalytic amount of TMG (1,1,3,3-tetramethylguanidine) to obtain the bicycle 77 (Scheme 9).^10*d*^

**Scheme 10:**
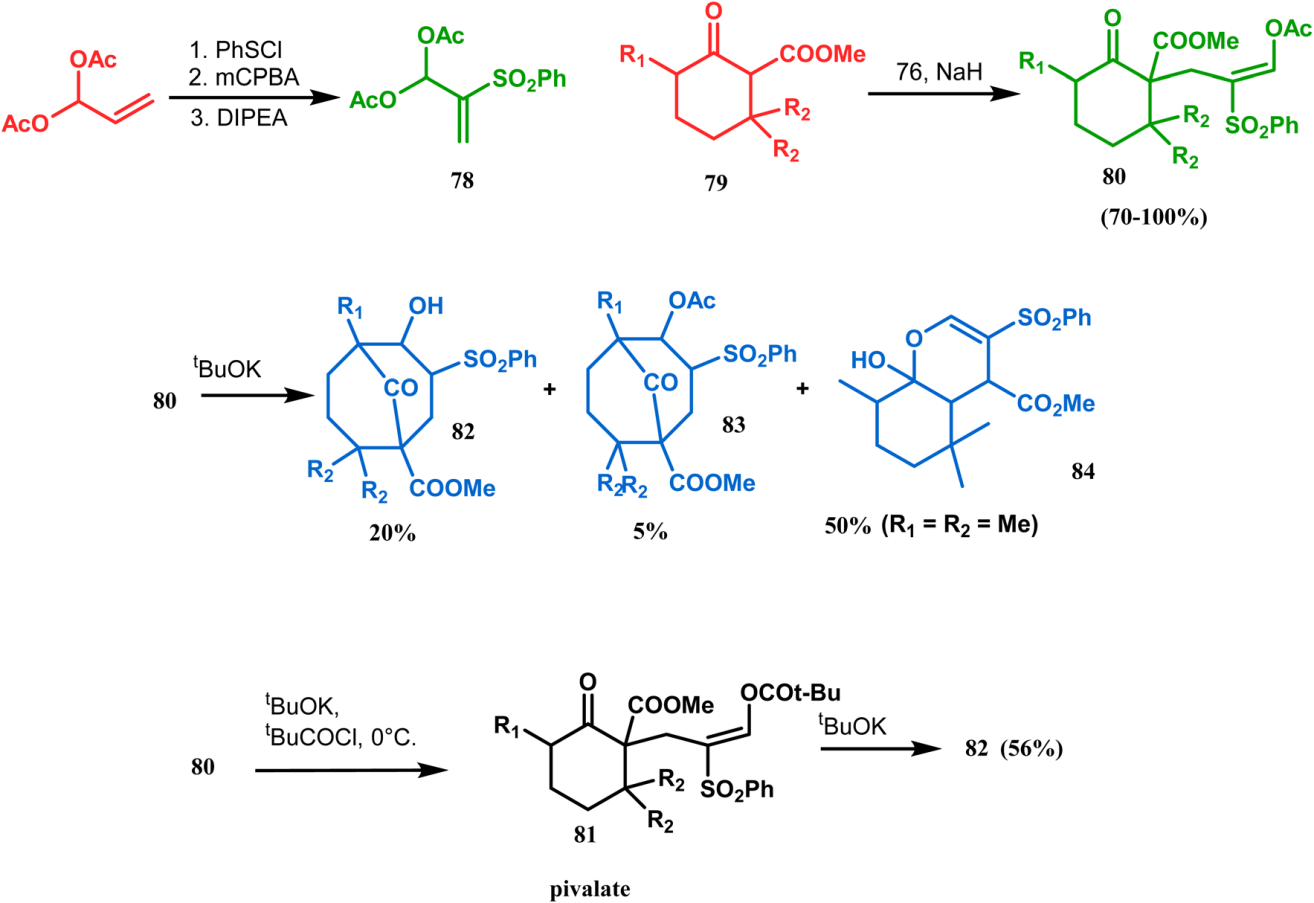
Reaction of diacetoxy sulfone and β-keto esters.

Kraus carried out a detailed investigation of the reaction between diacetoxy sulfone (78) and β-keto esters (79) (Scheme 10).^77^ The problem of easy deacetylation of 78, leading to unwanted deacetylated product 84 as the major product, was solved by converting 80 into the corresponding pivalate 81. When reacted with potassium *tert*-butoxide in THF, 79 gave the desired product 82 in 56% yield (Scheme 10). Similar results were also obtained with different R_1_ and R_2_.

**Scheme 11:**
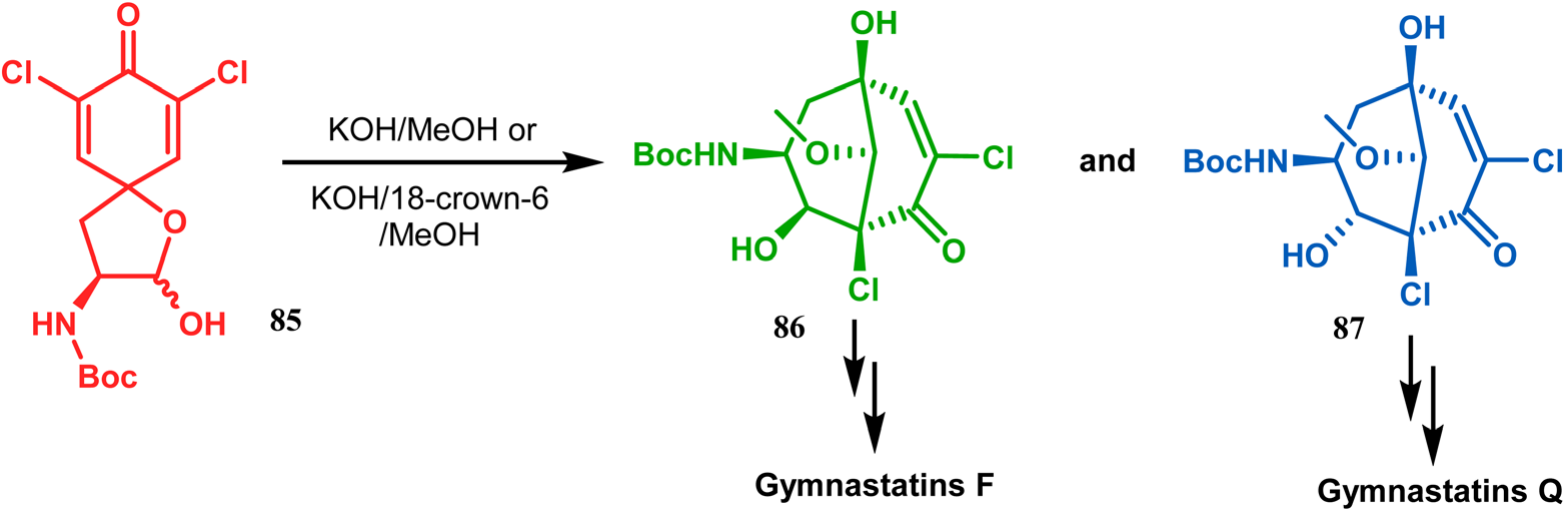
Synthetic route for the formation of the bicyclic core from a hemiacetal containing spirodienone through tandem Michael addition-intramolecular aldolization.

Base-induced tandem Michael addition-intramolecular aldolization is also well explored in natural product synthesis. A classic example in this category is the synthesis of gymnastatins F and Q.^78^ The synthesis of the key bicyclic core was accomplished by exposing a hemiacetal containing spirodienone (85) to KOH/MeOH or KOH/18-crown-6/MeOH to give 86 and 87, which were then converted to gymnastatins F (yield 36%) and Q (yield 64%), respectively, in a few synthetic steps (Scheme 11).

**Scheme 12:**
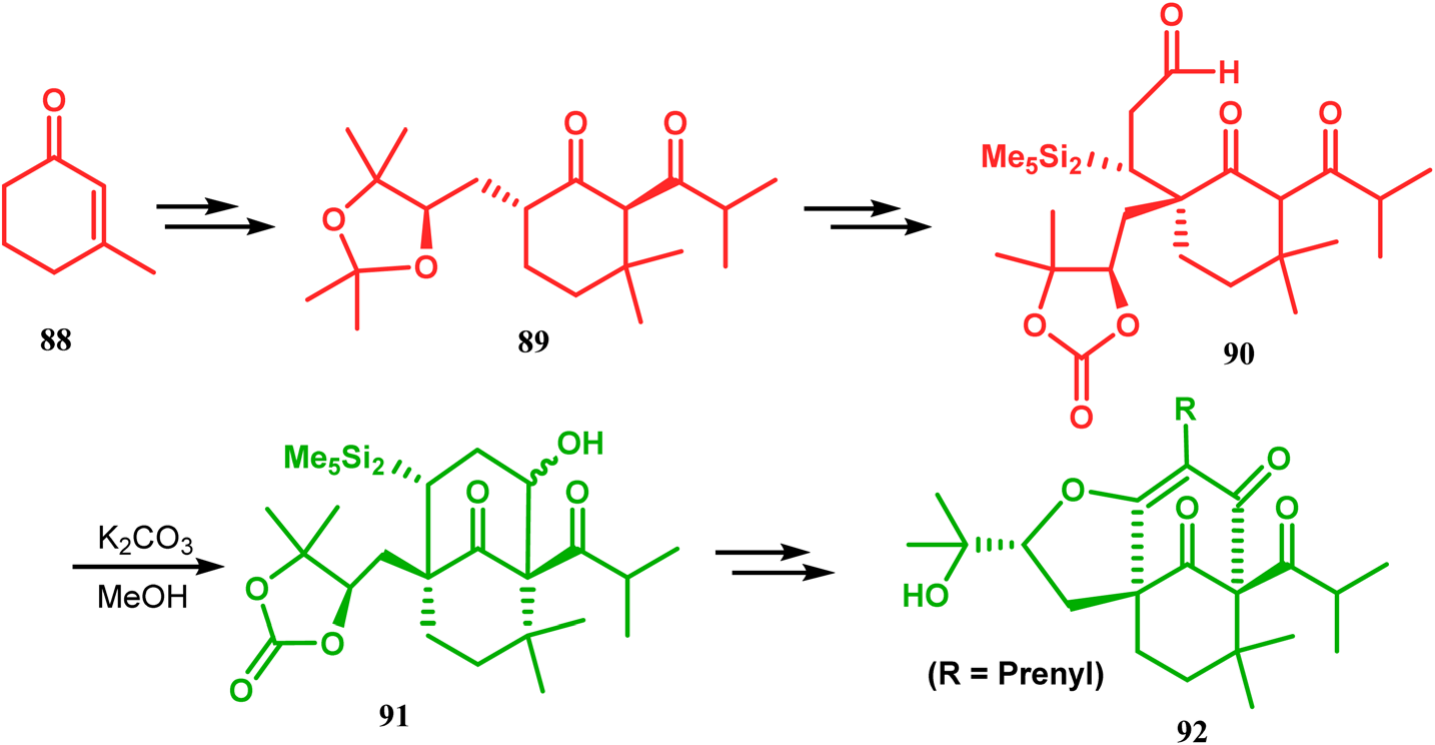
Base-promoted alodol condensation for the synthesis of 18-epimer of 8-deprenyl-garsubellin A.

**Scheme 13:**
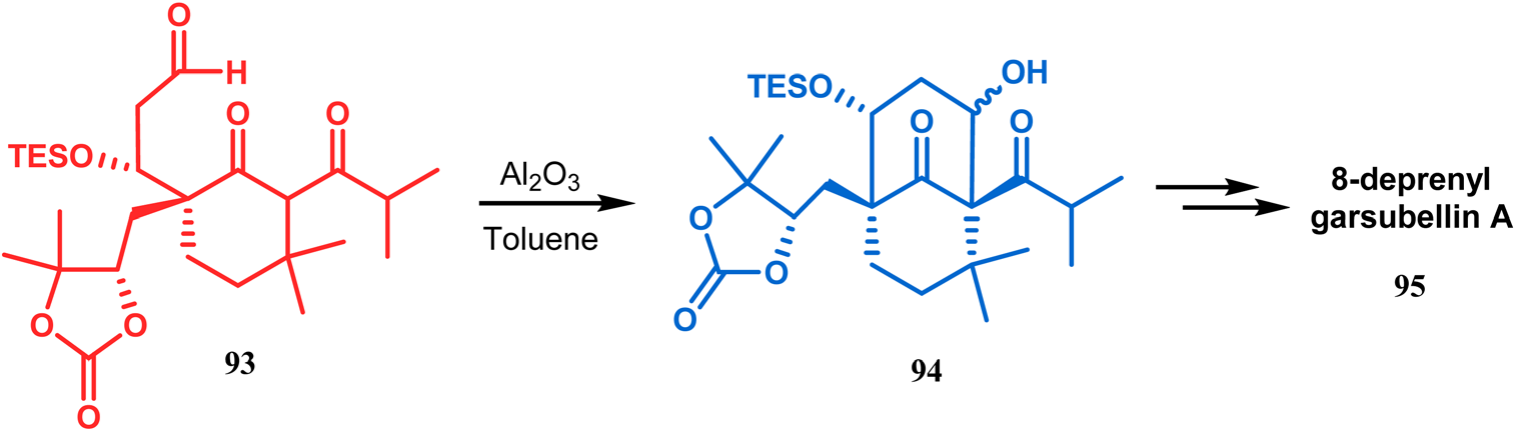
Synthetic route for the formation of garsubellin A in the presence of Al_2_O_3_.

Another application of base-promoted aldolization was unveiled by Usuda during the synthesis of 18-epimer (92) of 8-deprenyl-garsubellin A. The synthetic precursor (90) for this purpose was accessed in a stereocontrolled manner from enone 88 through intermediate 89. Precursor 90, upon-base promoted intramolecular aldol condensation, furnished the bicyclic core 91 (yield 98%) and then led to the formation of tricyclic compound 92 in 69% yield.^79^ In a similar report, the same authors (Usuda and coworkers) also accessed another garsubellin A analogue (95).^80^ However, in this case, Al_2_O_3_ was used as the base instead of K_2_CO_3_ because potassium carbonate leads to the β-elimination of TESOH, forming a mixture of products (Schemes 12 and 13).

**Scheme 14:**
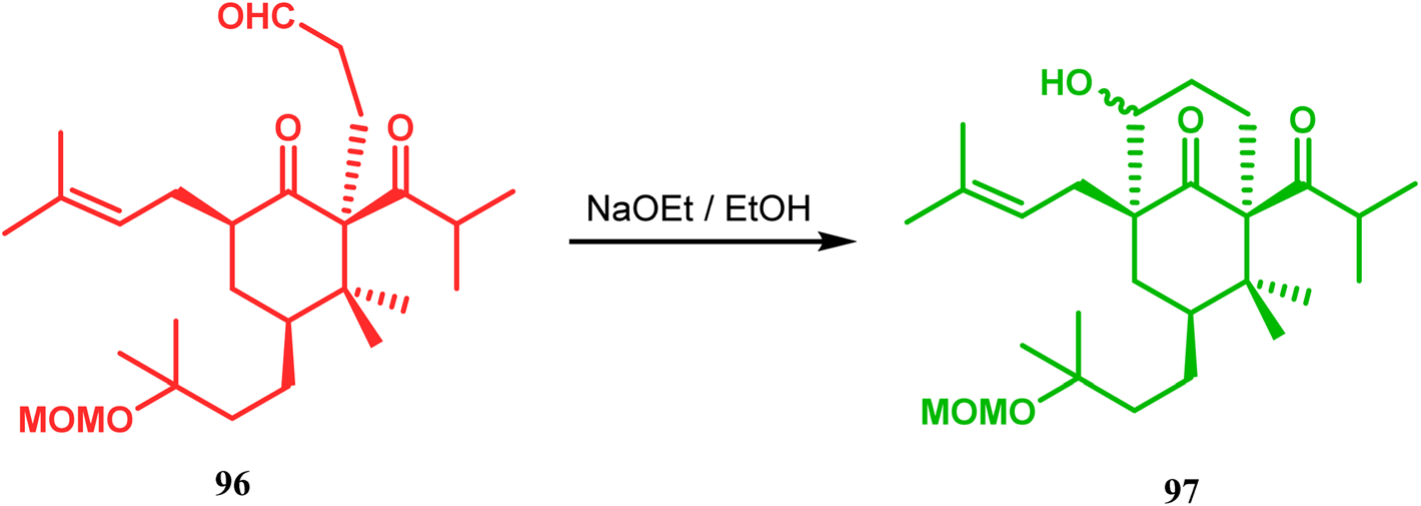
Construction of the bicyclic core of polycyclic polyprenylated acylphoroglucinol (PPAs) through base-promoted intramolecular aldolization.

**Scheme 15:**
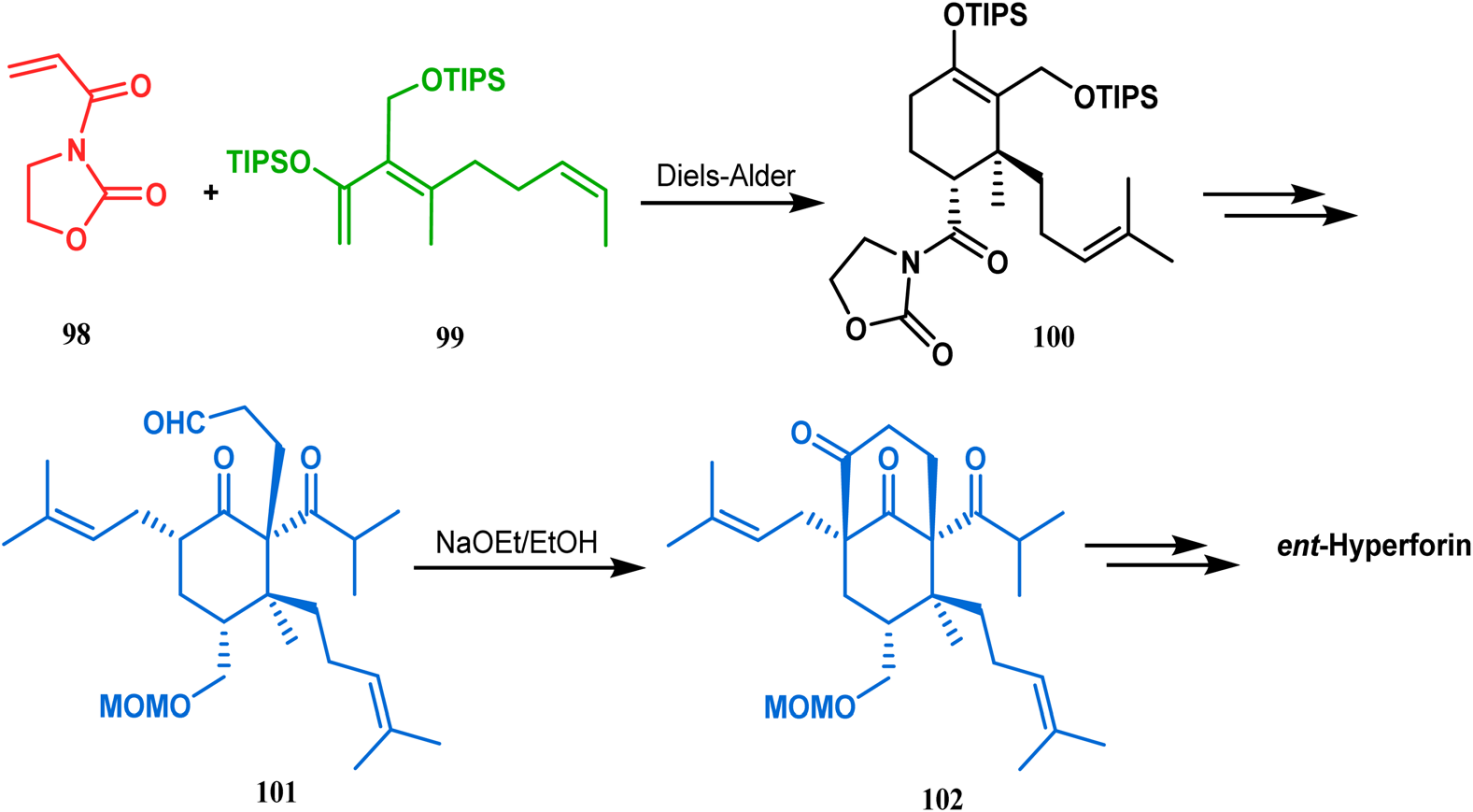
Formation of the bicyclic core of (−)-hyperforin through base-promoted intramolecular aldolization.

Similarly, the bicyclic core of PPAPs was constructed by Shibasaki's group through a base-promoted intramolecular aldolization technique. It proceeded through the synthesis of the annulation precursor 96, which was then intramolecularly aldolized to the bicycle 97 using NaOEt in EtOH (Scheme 14).^81^ Very recently, the same methodology was successfully utilized by them to synthesize enantiomerically pure (−)-hyperforin.^82^ Their targeted aldolization precursor (101) was prepared from the Diels–Alder adduct (100) of 98 and 99 in a stereocontrolled manner. NaOEt-promoted base-catalyzed intramolecular aldolization of 101 furnished the desired bicycle 102 (yield 86%), which was then sequentially converted to enantiomerically pure (−)-hyperforin (Scheme 15).^82^

**Scheme 16:**
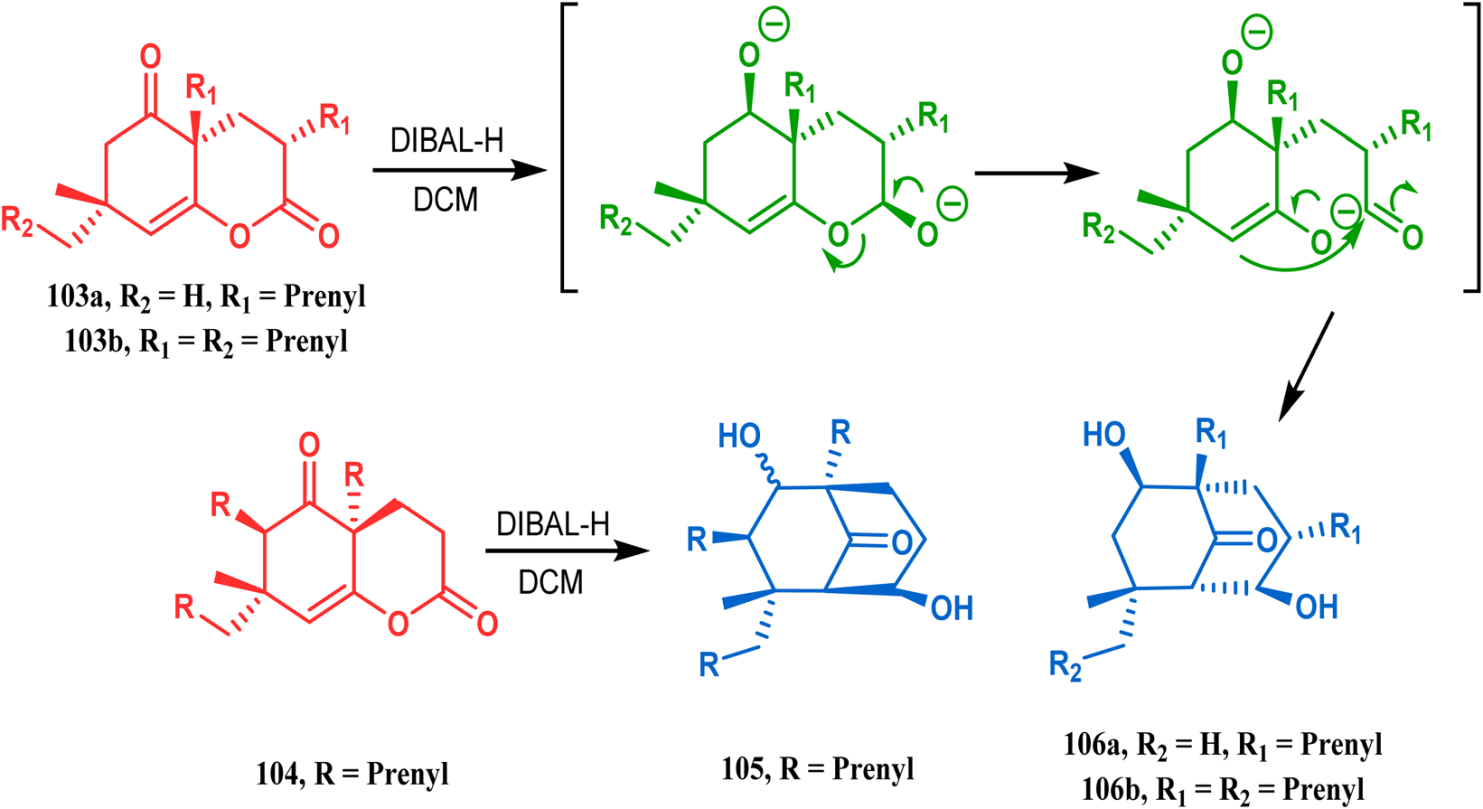
Synthetic strategy for obtaining the bicyclo[3.3.1]nonan-9-one cores of garsubellin A, hyperforin, guttiferone A, and hypersampsone F through DIBAL-H-mediated tandem lactone ring opening and intramolecular aldol condensation.

The synthesis of such prenylated bicyclononane core of phloroglucin natural products through base-promoted intramolecular aldolization was extensively investigated by Mehta's group. DIBAL-H-mediated tandem lactone ring opening and intramolecular aldol condensation were the key steps for synthesizing the bicyclo[3.3.1]nonan-9-one cores (105, 106) of garsubellin A, hyperforin, guttiferone A, and hypersampsone F. This is a classic example where the dual characteristic of DIBAL-H, both as a reducing agent (to reduce lactone 103 to a lactol anion) and as a base (to encourage aldol condensation), was successfully applied (Scheme 16).^83^

**Scheme 17:**
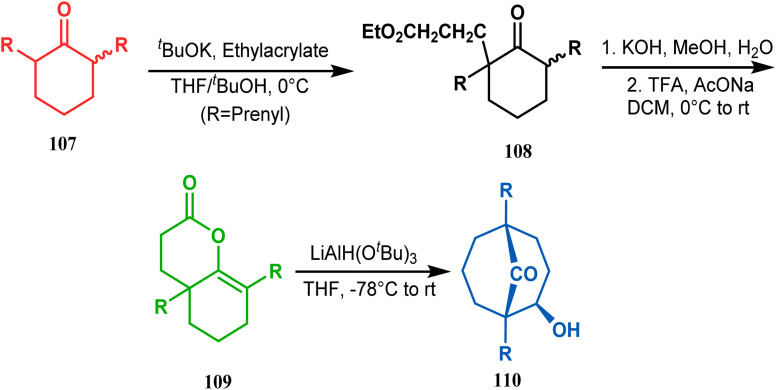
Synthesis of polyprenylated acylphloroglucinols *via* LiAlH(O^*t*^Bu)_3_-promoted intramolecular aldol condensation.

A similar attempt was also made by Marazano and coworkers to access models for polyprenylated acylphloroglucinols with a different reducing agent, LiAlH(O^*t*^Bu)_3_, instead of DIBAL-H. The desired bicyclo[3.3.1]nonan-9-one (110) was obtained in high yields (82%) by this procedure (Scheme 17).^84^

**Scheme 18:**
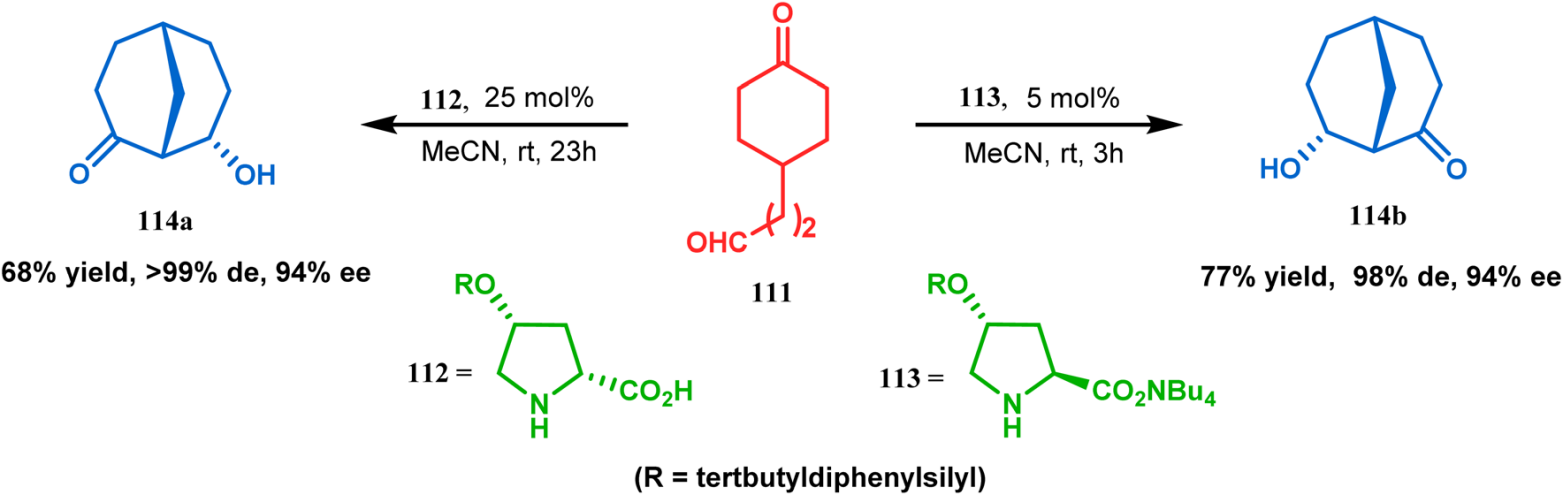
Formation of the bicyclo[3.3.1]nonane core *via* organo-catalyzed aldol condensation.

Apart from base-promoted aldolization techniques, the use of organocatalyzed aldol condensation to achieve the bicyclo[3.3.1]nonane core is well documented. An elaborate study was repored in this regard by Iwabuchi and coworkers. Although their initial attempt to synthesize 114 using l-proline as the catalyst produced the product in low yield and low stereoselectivity, modified proline-analogues 112 and 113 produced 114a and 112b, respectively, with high de and ee (Scheme 18). The result is attributed to the higher availability of catalytically active secondary amine of 112 and 113 than l-proline due to the lesser amount of zwitterion formation in the former two than in the last one. Besides, the hydrophobic environment produced by the tertbutyldiphenylsilyl group and the tetrabutylammonium ion around 112/113 lowered the p*K*_a_, thereby raising the nucleophilicity of amine nitrogen, which is required to catalyze the condensation. The reason behind the preference of 112 for 114a and that of 113 for 114b was rationalized from the favored H-bonding and dipole interaction between the developing ions in the transition state.^85^

**Scheme 19:**
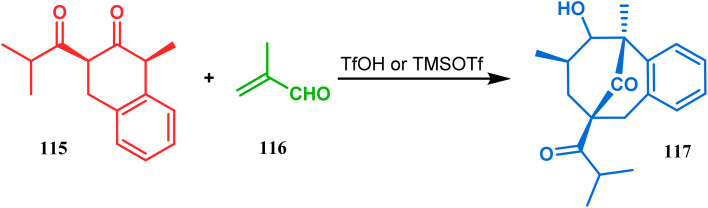
Synthesis of hyperforin model *via* acid-catalyzed tandem Michael addition-intramolecular aldol-type condensation of diketone with methyl acrolein.

An acid-catalyzed tandem Michael addition-intramolecular aldol-type condensation of diketones to achieve bicyclo[3.3.1]nonenone has been reported by Nicolaou's group. Their studies toward the synthesis of a hyperforin model system involved the treatment of diketone 115 with methyl acrolein (116) in the presence of TfOH or TMSOTf, which promoted Michael addition on the doubly activated carbon of 115, followed by an intramolecular aldolization, leading to bicycle 117 in 63% yield (Scheme 19).^86^

**Scheme 20:**
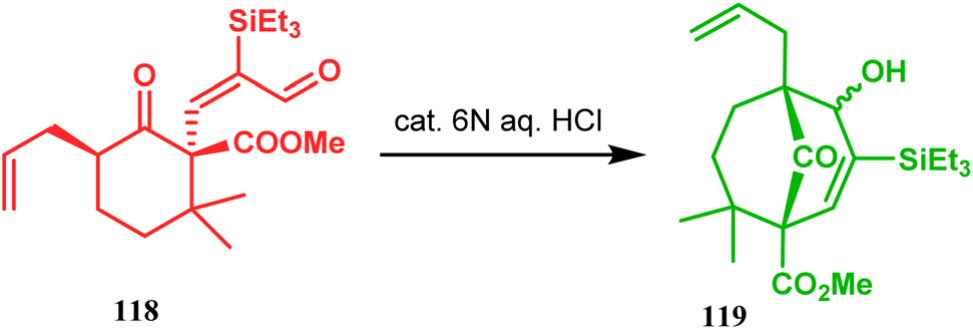
Synthesis of the bicyclic core of PPAPs *via* the acid-catalyzed intramolecular aldolization of α-silylenal.

**Scheme 21:**
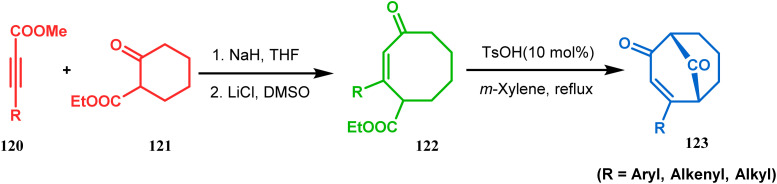
Synthesis of bicyclo[3.3.1] noneone *via* acid-catalyzed intramolecular *C*-alkylation.

**Scheme 22:**
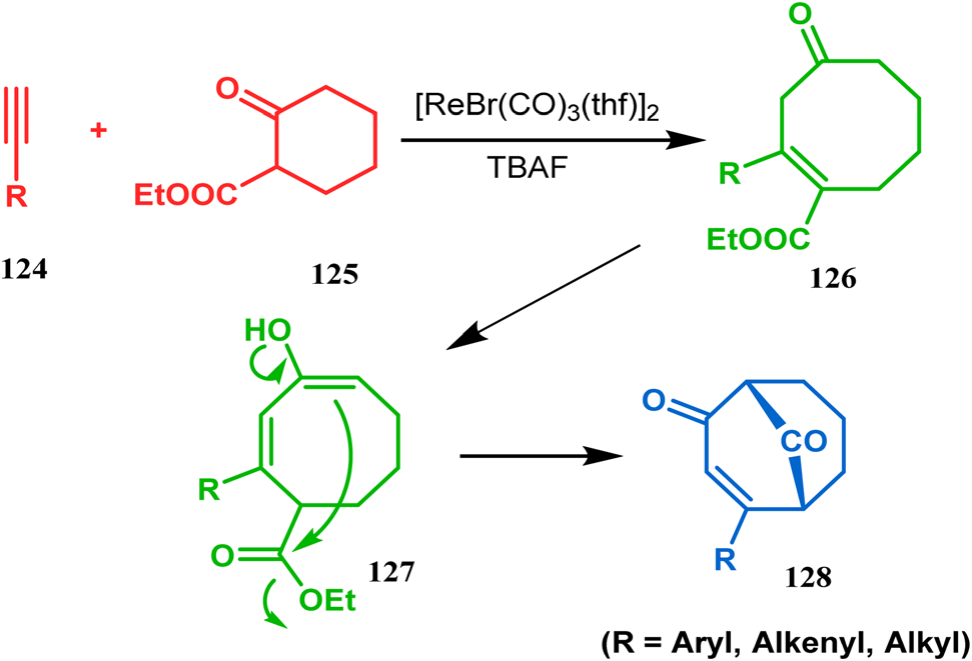
Synthesis of bicyclo[3.3.1]nonane-dione *via* [ReBr(CO)_3_(thf)]-catalyzed intramolecular condensation.

Acid-promoted routes toward the synthesis of bicyclic core of PPAPs are, however, also followed in Grossman's strategy, which involves an acid-catalyzed intramolecular aldolization of α-silyl enal 118 to furnish bicycle 119 in 72% yield (Scheme 20).^87^ Another acid-catalyzed methodology, developed by Dixon's group, involves the reaction with ester-carbonyl group, although it does not involve aldolization. Thus, when cyclooctanone derivative 122, obtained from 120 and 121, was treated with TsOH, an intramolecular *C*-alkylation at the α-position of the carbonyl group occurred, producing the bicyclo[3.3.1]noneone 123 in moderate to high yields (46–80%) (Scheme 21).^88^ Recently, Y. Kuninobu and coworkers utilized similar reaction partners 124 and 125 and treated them with [ReBr(CO)_3_(thf)]_2_ in the presence of TBAF. The reaction proceeded through the formation of cyclooctanone (126), which underwent an intramolecular condensation between the α-methylene of ketone and the ester functionality and produced another bicyclo[3.3.1]nonane-dione (128) (yield 79–93%) (Scheme 22).^89^

#### 3.1.2. Michael-type addition

**Scheme 23:**
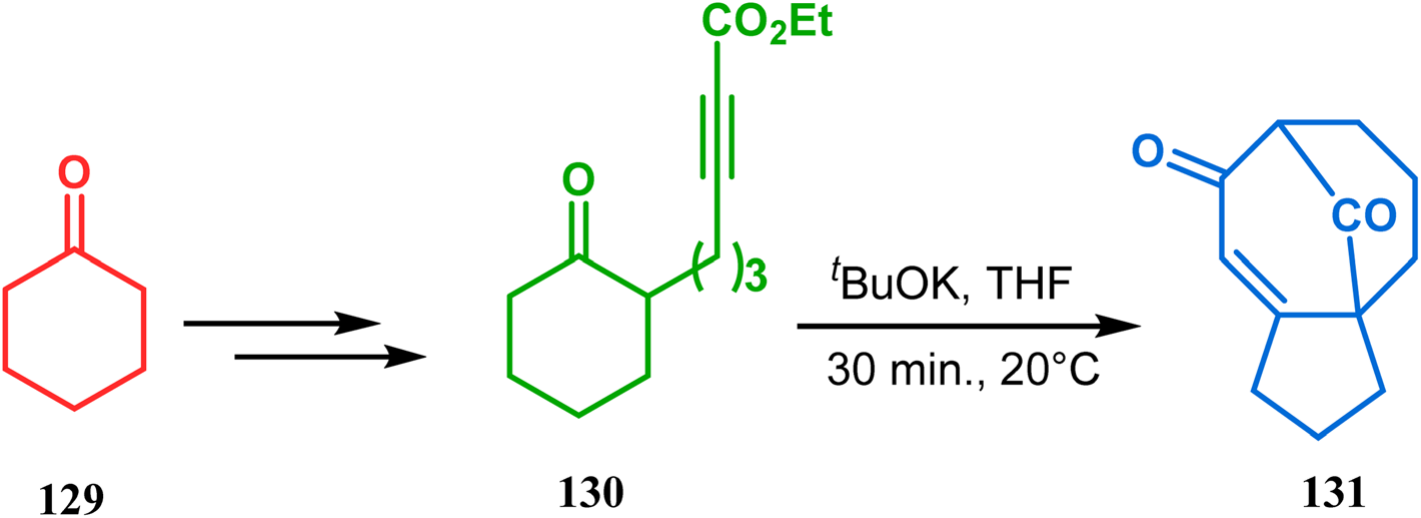
Synthesis of the bicyclo[3.3.1]nonane core containing tricyclic derivative through a tandem Michael addition–Claisen condensation cascade in the presence of ^*t*^BuOK.

The syntheses of such bicyclic ketones were also achieved by Michael addition reaction on acetylenic ω-ketoesters. Miesch and coworkers, while attempting the synthesis of novel oxetane derivatives from acetylenic ω-ketoesters (130) (yield 51%), accidentally found that though use of TBAF produces the desired product, the result was surprisingly modified when TBAF was replaced with ^*t*^BuOK. The reaction now yielded the bicyclo[3.3.1]nonane core containing tricyclic derivative 131 in 42% yield through a tandem Michael addition–Claisen condensation cascade (Scheme 23).^90,91^

**Scheme 24:**
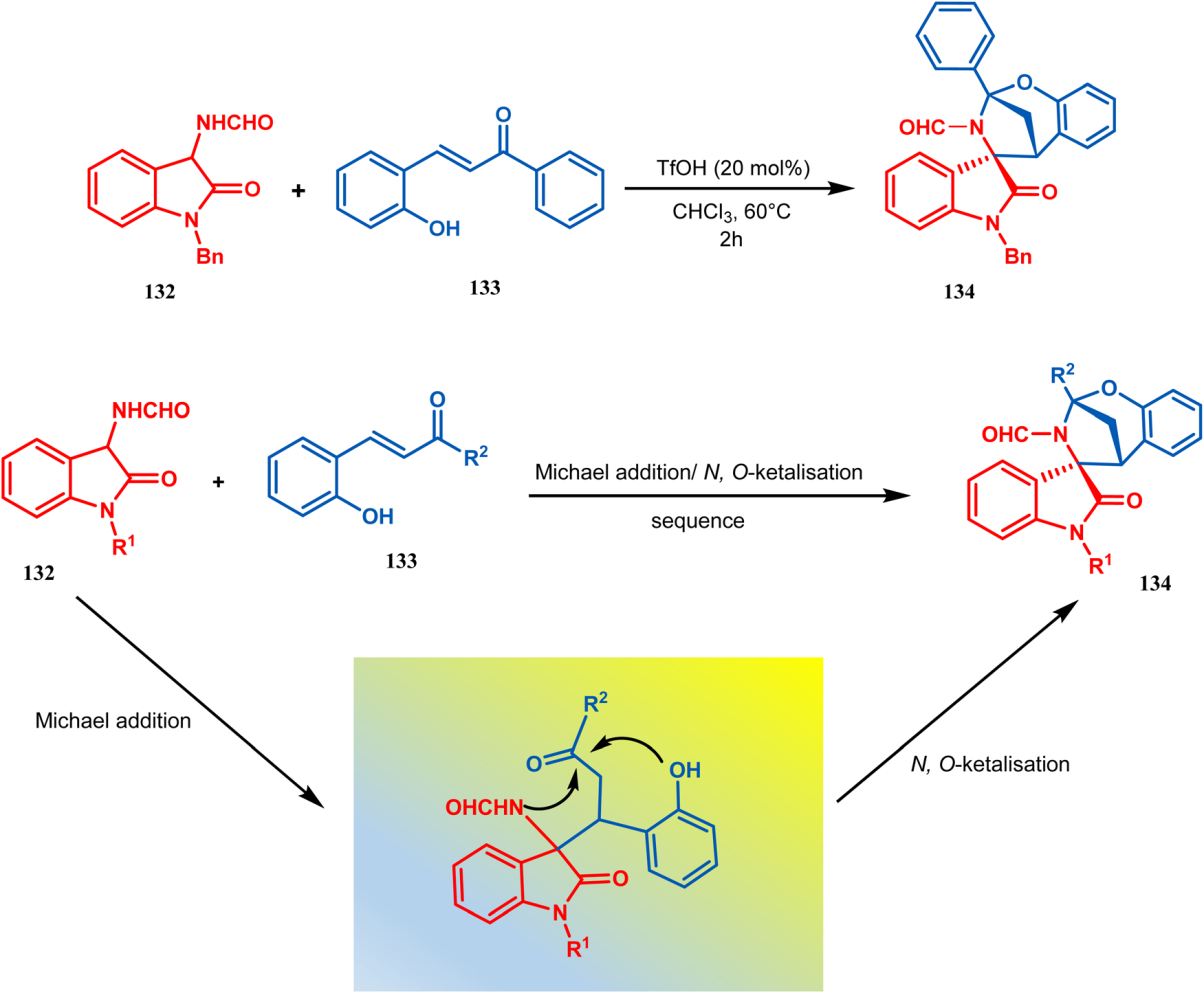
Synthesis of diastereoselective bridged cyclic *N*,*O*-ketal spirooxindole having a bicyclic core *via* the Michael addition-driven cyclization reaction.

Zhanwei Bu's group developed a new synthetic strategy for the synthesis of bridged cyclic *N*,*O*-ketal spirooxindoles *via* the Michael addition-driven cyclization reaction of 3-hydroxyoxindoles with ortho-hydroxy-chalcones (133). Also, the Michael addition/*N*,*O*-ketalization sequence of 3-aminooxindoles (132) with *ortho*-hydroxychalcones (133) may take place under particular conditions, but 3-amino-oxindole (132) showed relatively lower reactivity compared with 3-hydroxyoxindole. Therefore, they accomplished the Michael addition/*N*,*O*-ketalization sequence of 3-amino-oxindoles (132) with *ortho*-hydroxychalcones under the catalytic influence of TfOH. Thus, they were able to construct a series of diastereoselective bridged cyclic *N*,*O*-ketal spirooxindoles (134) with rigid and new skeletons (yield 73%) (Scheme 24).^92^

**Scheme 25:**
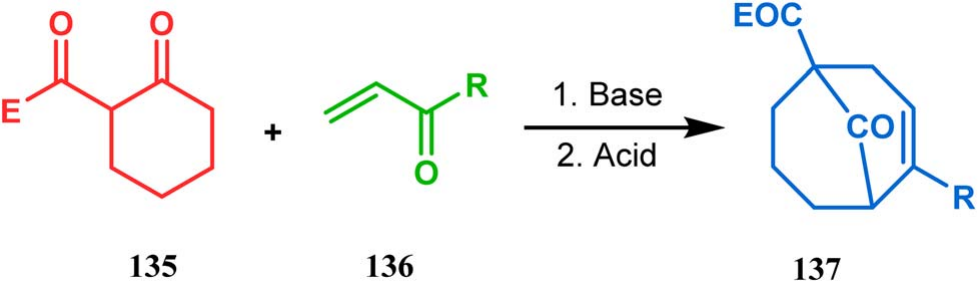
Synthesis of bicyclo[3.3.1]nonenones through the annulation of β-keto thiolesters or β-keto sulfones *via* base-catalyzed Michael addition and then acid-catalyzed aldol condensation reaction.

**Scheme 26:**
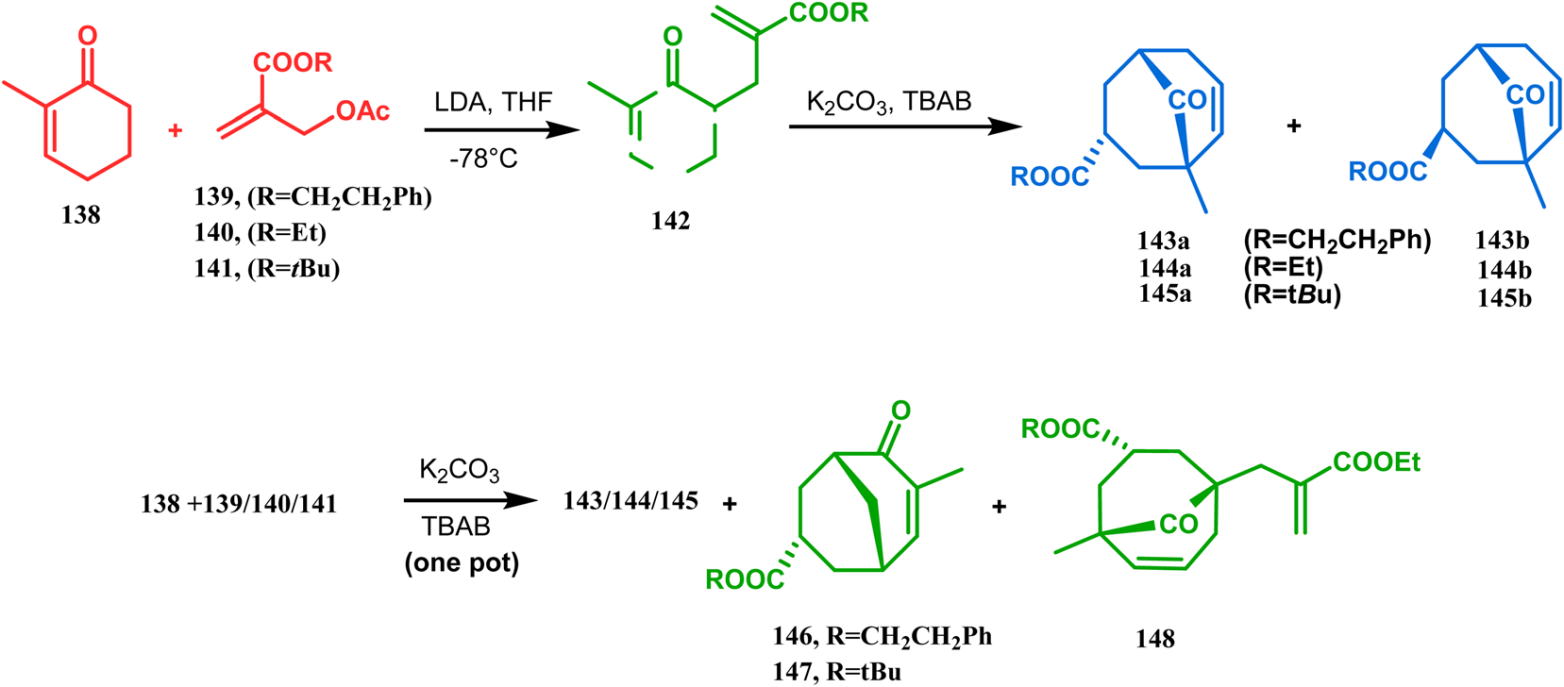
Synthesis of the bicyclo[3.3.1]nonane core *via* successive Michael addition reactions.

**Scheme 27:**
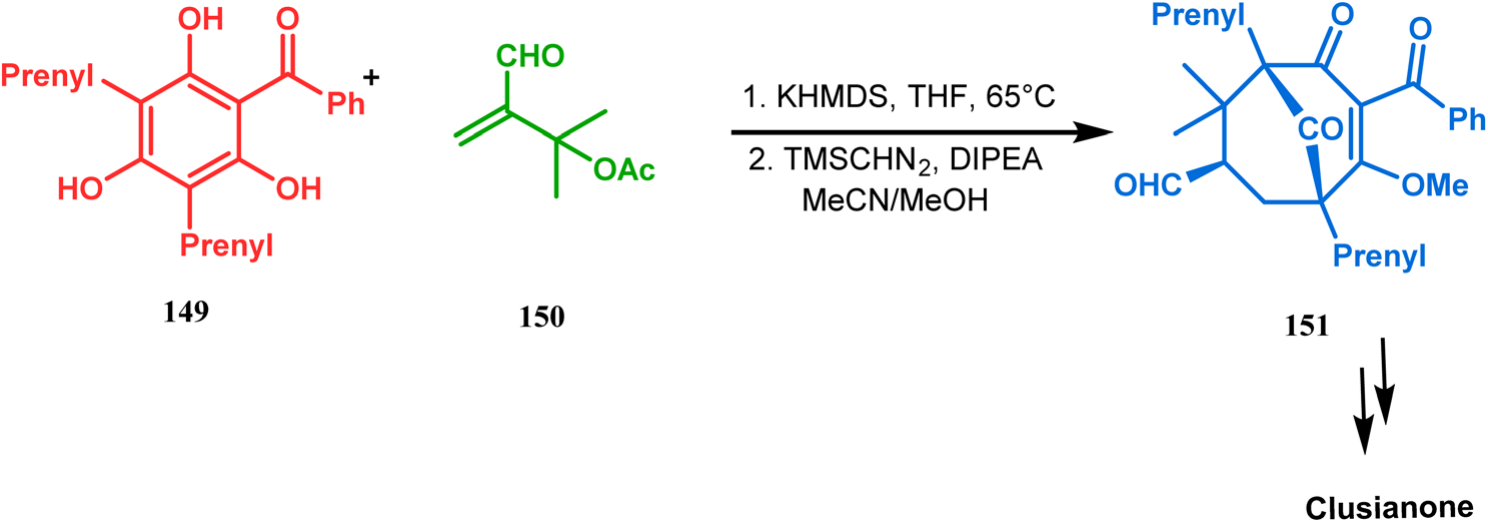
Synthesis of the bicyclo[3.3.1]nonane core of clusianone through alkylative dearomatization-annulation methodology.

Apart from this, the annulation of β-keto thiolesters or β-keto sulfones^93^ is also well explored for the synthesis of bicyclo[3.3.1] nonenones. The process involves a base-catalyzed Michael addition between 135 and 136, followed by an acid-catalyzed aldol condensation reaction, forming a β,γ-unsaturated bicyclo[3.3.1]nonenone (137) (Scheme 25). Such nonenones could also be accessed through Michael addition reaction on acrylate derivatives. For example, the β,γ-unsaturated bicyclo[3.3.1]nonenone (143) could be synthesized from 2-cyclohexenone (138) using two successive Michael addition reactions on 139. Although isomerization studies proved that 143b is thermodynamically more stable, the major product becomes 143a (yield 35–36%) *via* intermediate 142 (yield 50–74%). More interestingly, when a one-pot operation is performed instead of two consecutive operations, a minor amount of α,γ-annulation product (146) (yield 2–7%) is formed along with the α,α′-annulation products (143–145) (Scheme 26). In some cases, for example, with ethyl acrylate derivatives (145), three consecutive Michael addition-derived product 148 with 15% yield is also formed in addition (Scheme 26).^94^ Applying the same strategy, Porco and coworkers developed the alkylative dearomatization-annulation methodology, which was successfully utilized to achieve the bicyclo[3.3.1]nonane core of clusianone during its total synthesis. The methodology employed LiHMDS or KHMDS to realize the target 151 in 54% yield (Scheme 27) and was also successfully applied to construct multisubstituted bicyclic cores such as clusianone (yield 74%).^94^

**Scheme 28:**
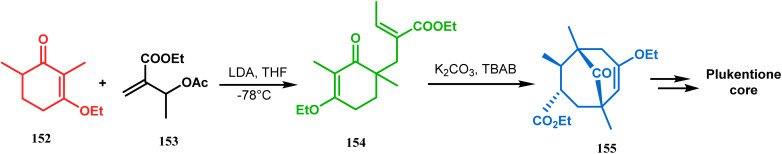
Synthesis of the adamantane core of plukentione-type PPAP.

**Scheme 29:**
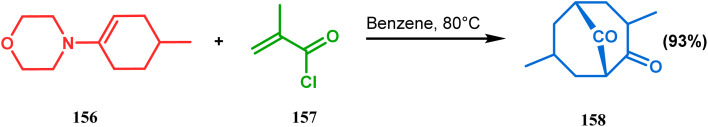
Synthesis of the bicyclo[3.3.1]nonane core *via* Michael addition and concomitant cyclization reaction.

Shortly after the communication by Porco's group, the same strategy was applied by Takagi and coworkers to successfully synthesize the adamantane core of plukentione-type PPAP.^95^ The cyclohexenone derivative 152 was reacted with acrylate 153 and the annulation precursor 154 (*E*/*Z* 25 : 1) was obtained in 92% yield. To achieve 155, best results were obtained when 154 was treated with K_2_CO_3_ and TBAB. Although the use of cesium carbonate increases the yield of the targeted bicyclic core (155) (from 41–55% yield), the unwanted α,γ-annulation product was also produced as a substantial impurity. However, in both cases, product 155 was obtained as a diastereoisomeric mixture (Scheme 28). Once the bicyclic core was synthesized, a few more synthetic steps led to the desired skeleton.^97^ In another report by Gambacorta's group, the morpholine derivative 156 was utilized as the Michael donor and methacryloyl chloride (157) as the Michael acceptor. Refluxing in benzene facilitated Michael addition and concomitant cyclization to yield the bicyclo[3.3.1]nonane 158 in 93% yield (Scheme 29).^96^

**Scheme 30:**
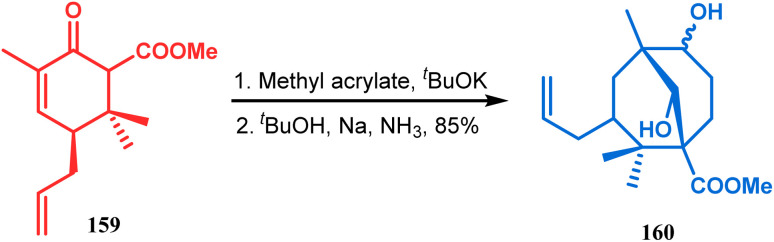
Synthesis of papuaforin A *via* Michael addition, followed by Birch reduction and cyclization.

The application of such a reactive starting material pair (cyclohexenone and acrylate) was also extensively studied by Kraus and coworkers. Their route for the total synthesis of papuaforin A unveils one such attempt. In this case, the cyclohexenone derivative 159 was treated with methyl acrylate in the presence of ^*t*^BuOK, and the intermediate Michael addition product was subjected to Birch reduction/cyclization^97,98^ to give 160 (Scheme 30), which was then used as the precursor for the synthesis of papuaforin A.^15*a*^

**Scheme 31:**
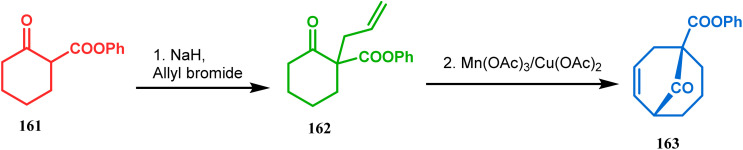
Synthesis of the bicyclo[3.3.1]nonane core by the reaction of allyl bromide and cyclohexanone derivative.

**Scheme 32:**
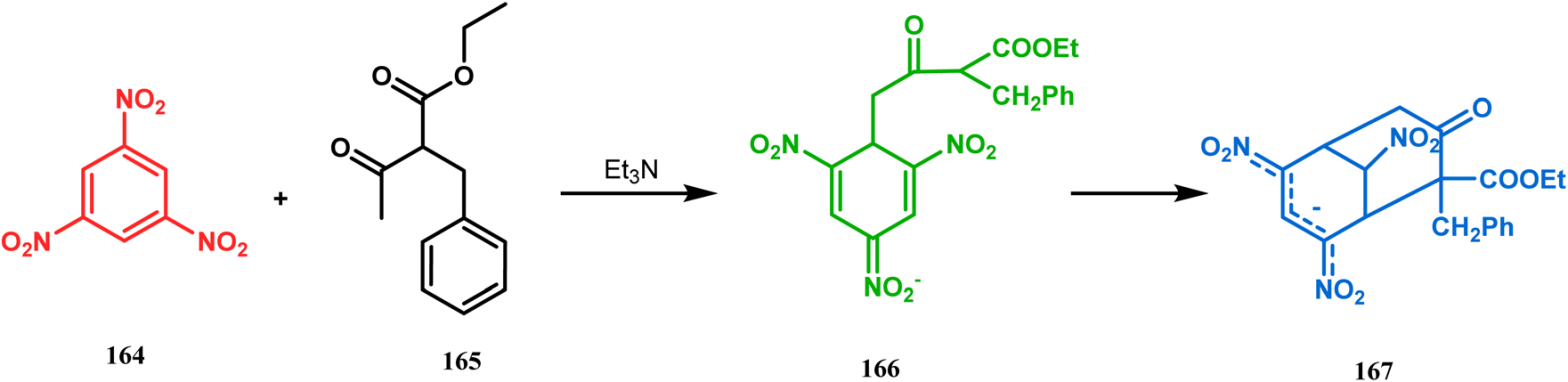
Formation of the bicyclo[3.3.1]nonane core by the Michael addition of 1,3-dicarbonyl compound and trinitrobenzene.

Another application of cyclohexanone by the same group utilizes allyl bromide instead of acrylate derivatives to achieve a similar bicyclo[3.3.1]nonane core. In this case, the synthesis of the bicyclic core of hyperforin and nemorosone was attempted by treating 161 with allyl bromide in the presence of sodium hydride. The intermediate 162 thus formed was then intramolecularly cyclized to 163 by manganic triacetate and cupric acetate having yield of 60% (Scheme 31).^15*b*,99^ The use of such 1,3-dicarbonyls as the Michael donor is also exemplified by Kalaivani's group. Thus, 1-benzyl-1-(ethoxycarbonyl)-2-propanone (BEP, 165) was treated with trinitrobenzene (TNB, 164) in the presence of triethyl amine, where TNB acted as the Michael acceptor and the anionic sigma complex 167 was formed (Scheme 32).^99^

**Scheme 33:**
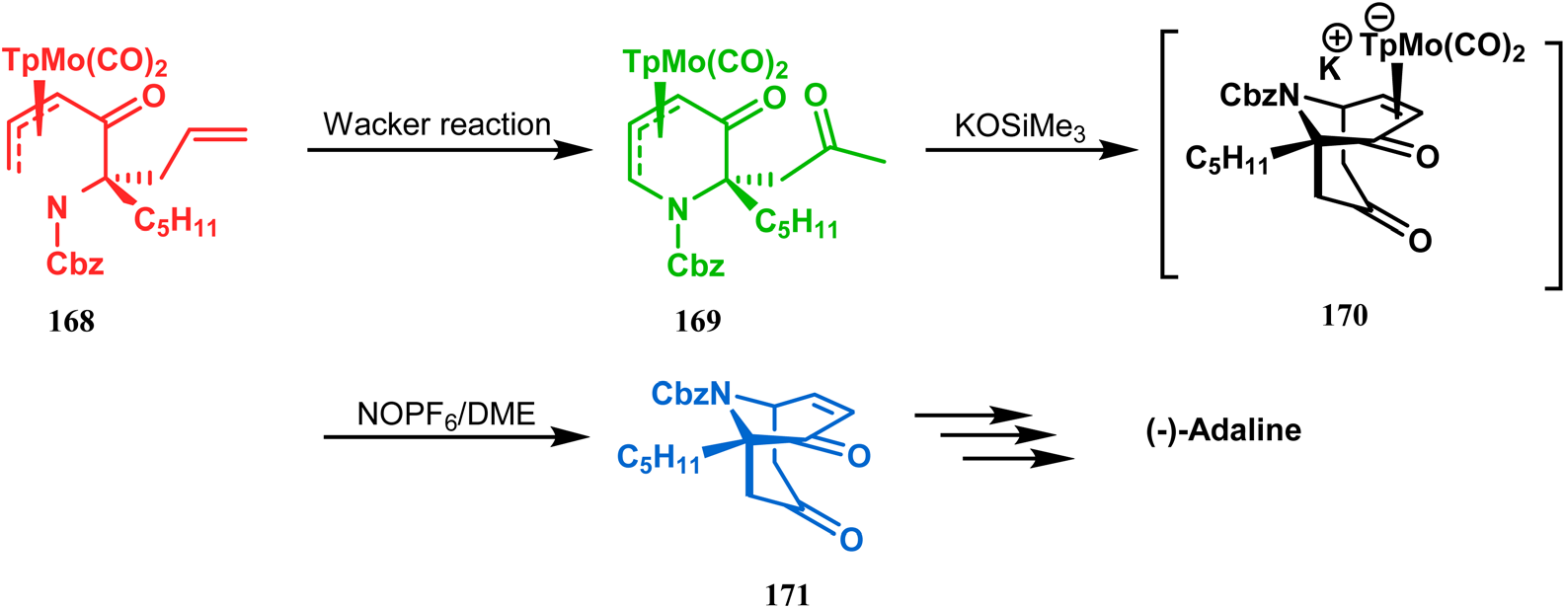
Synthetic route for the formation of enantiomerically pure (−)-adaline.

However, Liebeskind and coworkers have described a unique application of a Michael-like addition reaction in the synthesis of natural products using organometallic enantiomeric scaffolding. The authors utilized a TpMo(CO)_2_(5-oxo-η^3^-pyridinyl) complex (168) to create the methylketone (169) by Wacker reaction. The Michael-like addition precursor (169) was then reacted with KOSiMe_3_, which promoted the 1,5-Michael-like bond forming reaction through attack at the neutral η^3^-allylmolybdenum by the enolized ketomethyl group. The anionic intermediate 170 furnished the bicycle 171 when treated with NOPF_6_ in DME. A sequential ketalization, reduction, and carbobenzyloxy-deprotection then led to the formation of enantiomerically pure (−)-adaline (Scheme 33).^10^

#### 3.1.3. Intramolecular cation capture

**Scheme 34:**
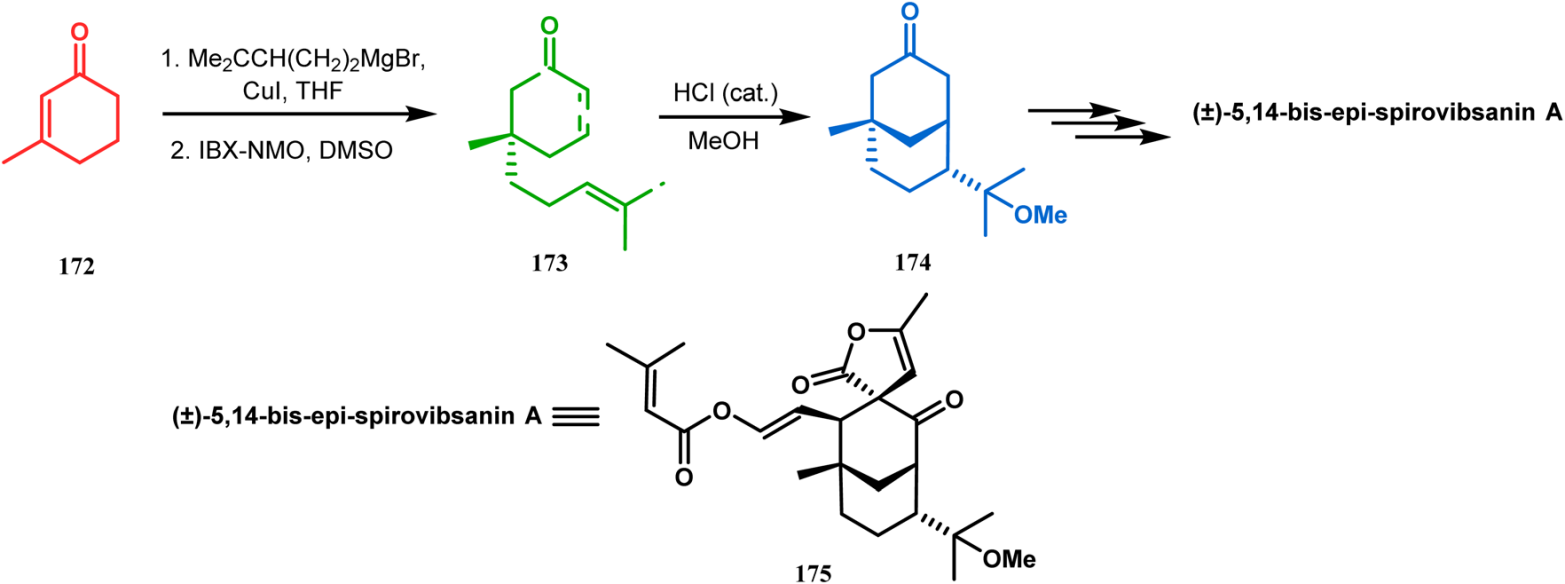
Synthesis of (±)-5,14-bis-*epi*-spirovibsanin A containing the bicyclo[3.3.1]nonane core through intramolecular cation capture.

**Scheme 35:**
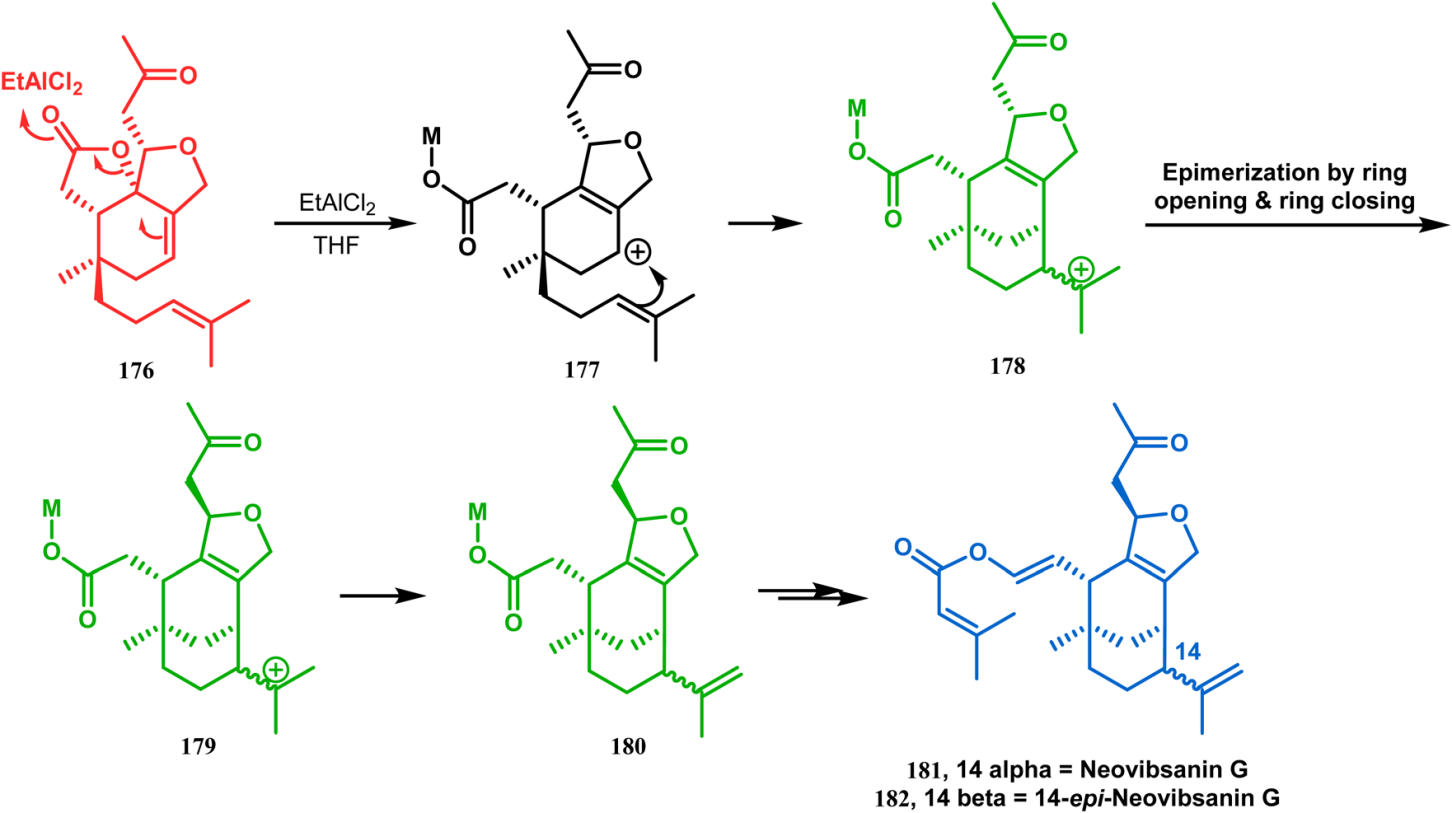
Synthesis of (−)-neovibsanin G and 14-*epi*-neovibsanin G through intramolecular cation capture.

Apart from these two major classes of intramolecular C–C bond forming approaches, there are several other unique strategies that have applied for the synthesis of the bicyclo[3.3.1]nonane core. One such example is intramolecular carbocation capture. The application of this strategy to obtain the bicyclic core was first demonstrated by Williams's group during the total synthesis of (±)-5,14-bis-*epi*-spirovibsanin A. Following Bernhardt's method,^100–103^ initially, they synthesized the racemic enone 173 (yield 70%, 91% ee) from the cyclohexenone derivative 172. The enone (173) was then treated with HCl in methanol, thereby generating a carbocation, which was intramolecularly captured to give the bicyclic ketone 174. Subsequently, through a number of synthetic steps, this bicyclic precursor then produced (±)-5,14-bis-*epi*-spirovibsanin A (175) (Scheme 34).^11^ A similar strategy was again used by the same group very recently during the total synthesis of (−)-neovibsanin G and 14-*epi*-neovibsanin G (181 & 182). However, instead of HCl or H_2_SO_4_, the Lewis acid EtAlCl_2_ was employed this time to carry out the reaction on 176. The desired bicycle (180) was formed due to the epimerization of 178 to 179 to relieve the steric strain between the adjacent carbonyl functionalities. Compound 180 was then sequentially converted into the targeted natural products (Scheme 35).

**Scheme 36:**
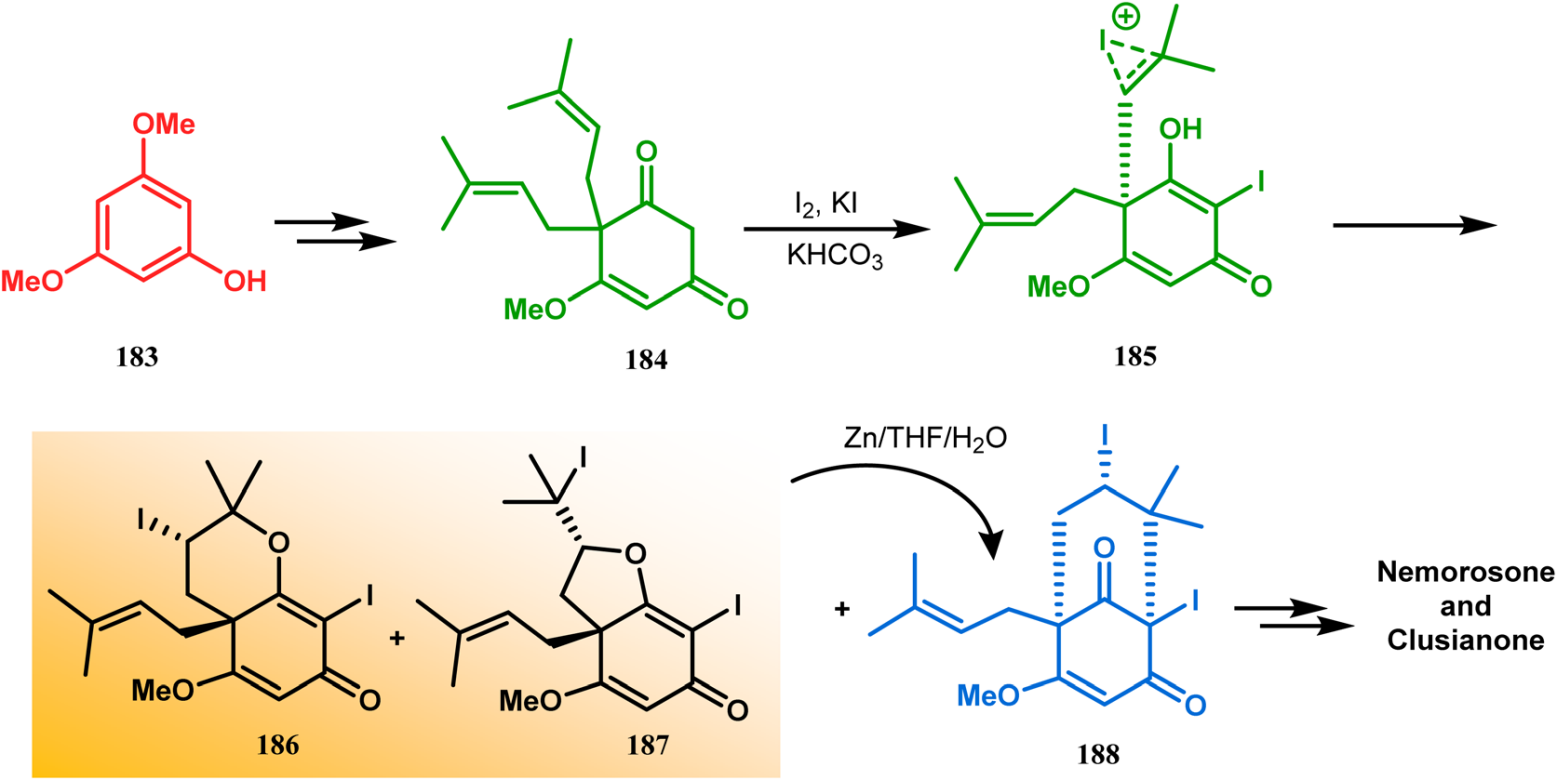
Synthesis of nemorosone and clusianone through intramolecular iodonium capture.

**Scheme 37:**
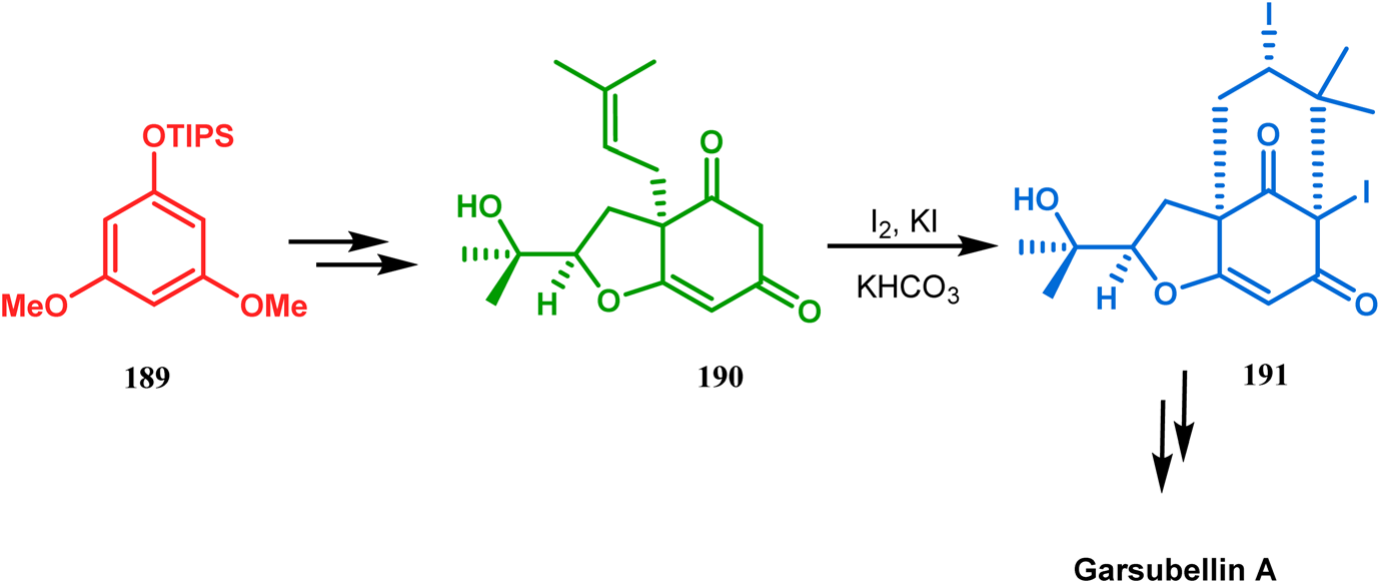
Synthesis of garsubellin a through intramolecular iodonium capture.

Intramolecular iodonium capture is another such pioneering approach applied by Danishefsky's group for preparing the bicyclo[3.3.1]nonane core during the total synthesis of nemorosone and clusianone. Properly substituted precursor 185, required for their envisioned iodonium formation and carbocyclization, was synthesized from phloroglucinol derivative 183 and reacted with iodine in the presence of KI-KHCO_3_ to form the desired bicyclic core (188) through iodonium intermediates. During the carbocyclization of 185, two other undesired cyclization products (186, 187) were also formed with yield 78% and 87%, respectively, along with 188. This problem was solved through a high yielding conversion of 186 and 187 into 188 (Scheme 36). However, an identical route toward garsubellin A did not encounter such problems. It is believed that the tetrahydrofuran group present in 190 tilted the conformation of the intermediate iodonium such that the iodinative cyclization proceeded strictly through C–C bond formation, resulting in the exclusive formation of the desired bicycle 191 (Scheme 37). The bicycles (188 and 191) thus formed were then converted to the targeted polyprenylated acylphloroglucinols (PPAPs) through a few more synthetic steps.^104,105^

**Scheme 38:**
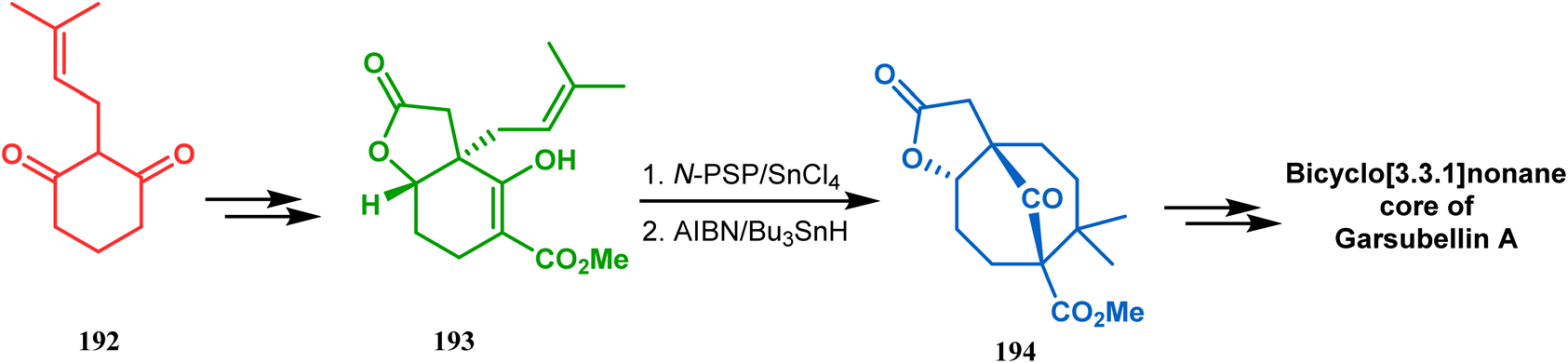
Synthesis of the bicyclo[3.3.1]nonane core of garsubellin A.

A strategy developed by Nicolaou's group, similar to this iodonium-induced carbocyclization, uses the oxyselenation of olefins.^106–108^ During the synthesis of the fully functionalized bicyclic core of garsubellin A, this group disclosed a unique application of the *N*-(phenylseleno)phthalimide/SnCl_4_ pair for bicyclo[3.3.1]nonane synthesis. The precursor 193, prepared from 1,3-cyclohexadione (192), produced the desired bicycle 194 in 95% yield when treated with *N*-PSP/SnCl_4_ in DCM at −23 °C (Scheme 38). The same group utilized this important methodology even in the solid phase synthesis of resin-bound polyfunctionalized bicyclo[3.3.1]nonanes using a one-step loading/cyclization method.

**Scheme 39:**
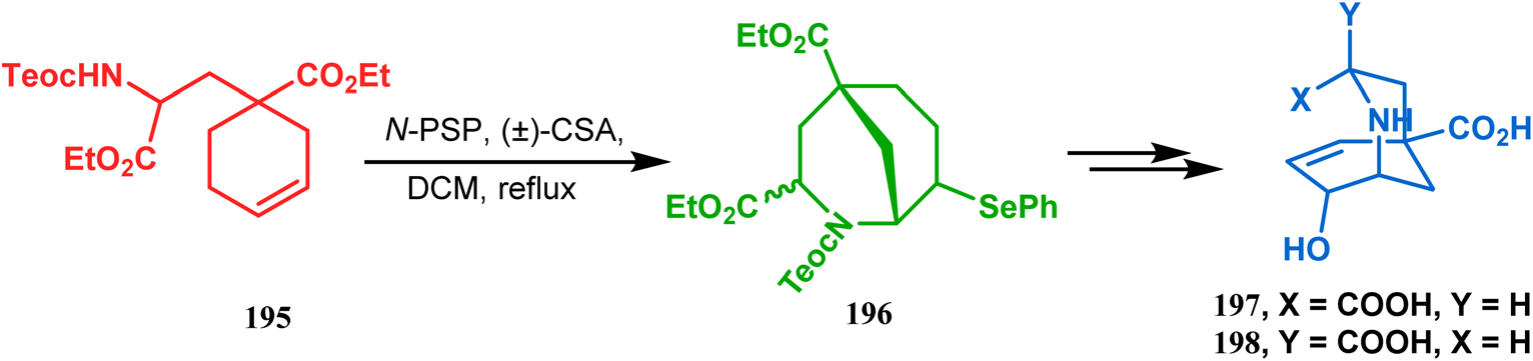
Synthesis of azabicyclo[3.3.1]nonane systems.

**Scheme 40:**
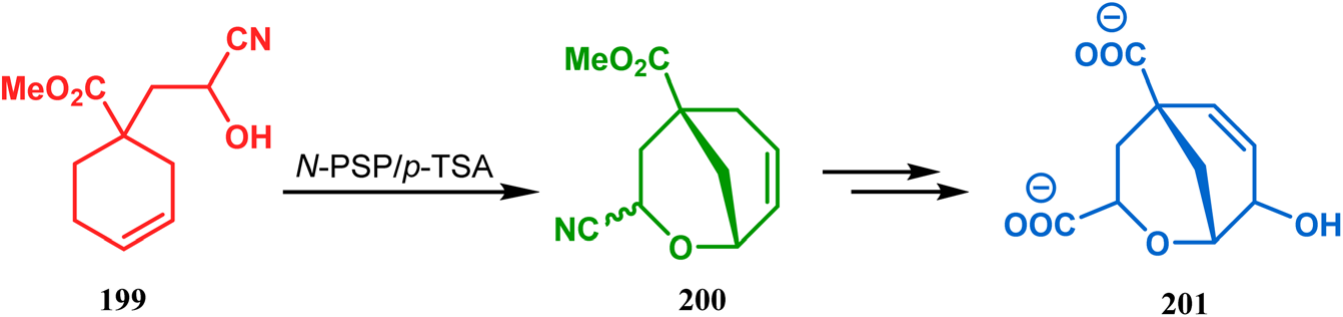
Synthesis of another potent inhibitor of chorismate mutase.

This versatile strategy was also used by Hediger to synthesize a novel class of chorismate mutase inhibitors based on azabicyclo[3.3.1]nonane systems. The key bicyclic core 196 was synthesized from 195 by treating with *N*-PSP and camphorsulfonic acid (Scheme 39).^109^ The synthesis of this class of chorismate mutase inhibitors was, however, well investigated by Bartlett's group.^110,111^ Starting from a cyclohexene derivative 199, they utilized the same *N*-PSP-mediated strategy to access the ether bicycle 200, which after a few synthetic steps led to 201, a potent inhibitor of chorismate mutase (Scheme 40).

#### 3.1.4. Effenburger-type cyclization

**Scheme 41:**
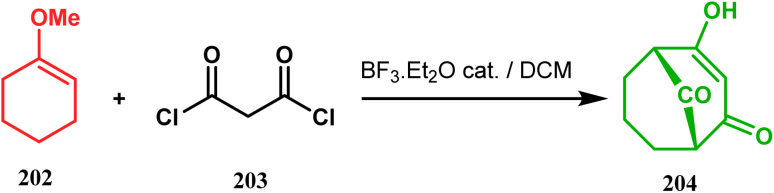
Synthesis of the bicyclo[3.3.1]nonane core following Effenburger-type cyclization.

**Scheme 42:**
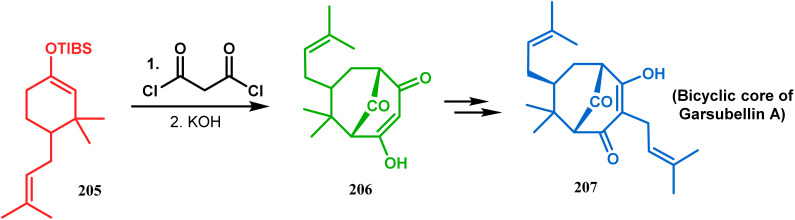
Formation of the bicyclic core of garsubellin A following Effenburger-type cyclization.

**Scheme 43:**
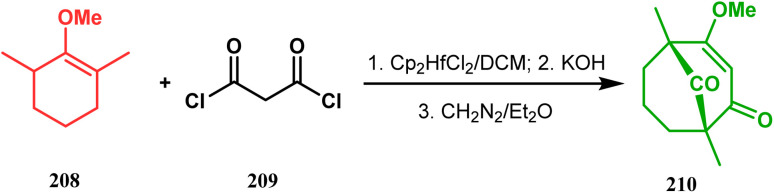
Synthesis of the bicyclic core with the use of methyl enol ether.

In 1984, Effenberger discovered an efficient route toward the synthesis of bicyclo[3.3.1]nonane ring systems through the reaction of 1-methoxy-1-cyclohexene (202) and malonyl dichloride (203). Since then, this versatile methodology was used by several research groups to achieve synthetic targets containing the bicyclic core 204 (Scheme 41). For example, Stoltz and coworkers applied this strategy on properly functionalized cyclohexanone enol ether 205 and obtained their desired bicycle 206 in 36–55% yield, required for the synthesis of the bicyclic core (207) of garsubellin A in high yield (Scheme 42).^112^ To achieve this target, the authors reversed the ratio of the starting materials compared to the Effenberger study and used a TBS enol ether instead of methyl enol ether. When a more hindered enol ether (α,α′-disubstituted) was subjected to Effenberger-type cyclization under the similar reaction protocol using TBS enol ether, the reaction failed. However, the methyl enol ether 208 could be cyclized to 210 in 25% yield in the presence of bis(cyclopentadienyl)hafnium dichloride, a Lewis acid mediator (Scheme 43).^113^ Simpkins and coworkers also proved that the methyl enol ether is the best choice for hindered substrates.

**Scheme 44:**
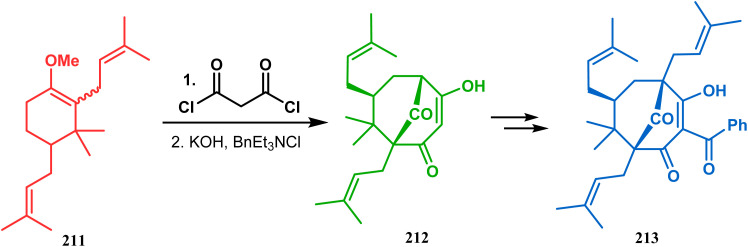
Synthesis of clusianone from substituted methyl enol ether under Effenberger cyclization condition.

**Scheme 45:**
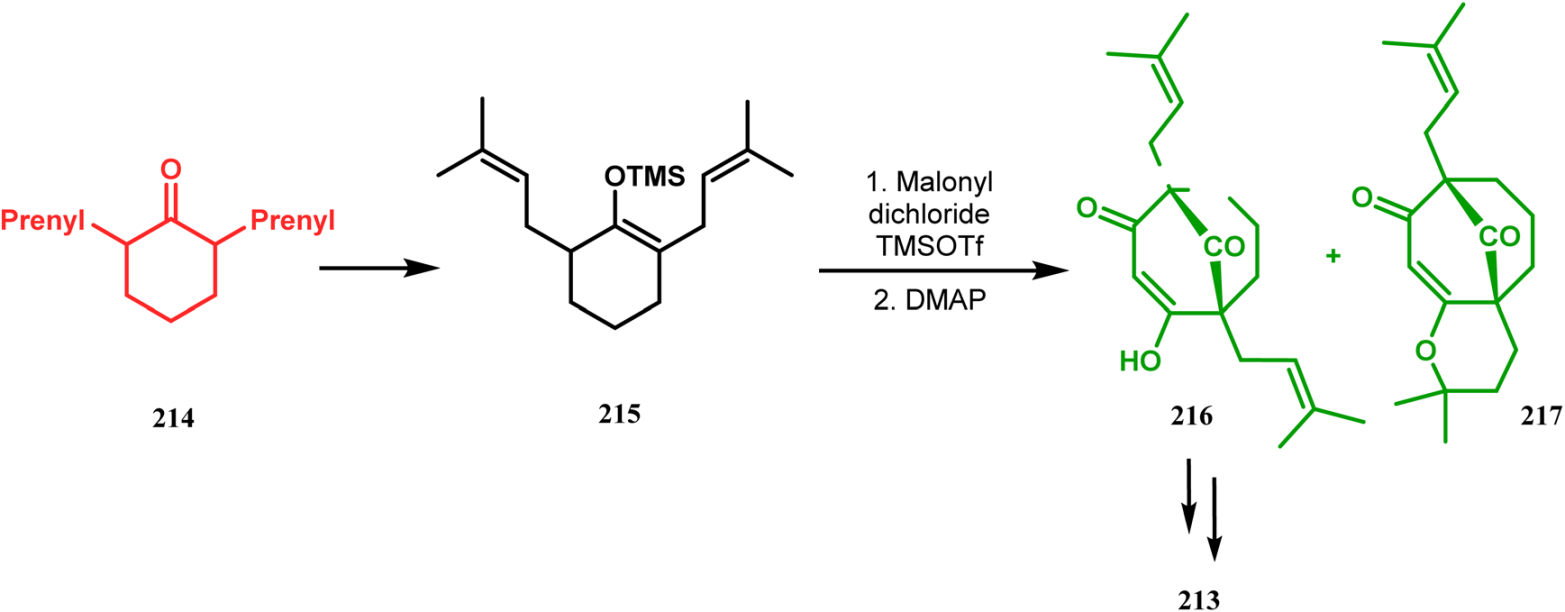
Synthesis of the bicyclo[3.3.1]nonane core of clusianone using a hindered TMS enol ether of 2,6-diprenyl cyclohexanone.

**Scheme 46:**
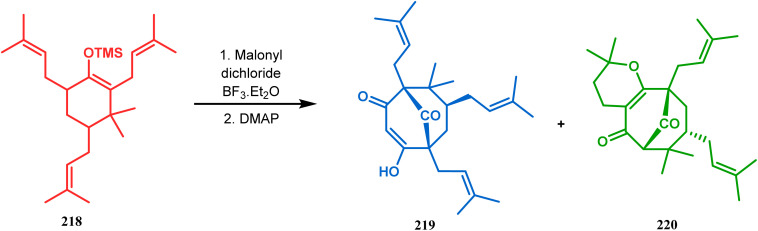
Synthesis of the bicyclic core along with a tricyclic derivative by the reaction of hindered TMS enol ether and malonyl dichloride.

Thus, while attempting the total synthesis of clusianone, the authors subjected an appropriately substituted methyl enol ether 211 to Effenberger cyclization condition and obtained the bicycle 212, which then led to racemic clusianone 213 (yield 90%) in a few synthetic steps (Scheme 44).^114^ The separation of these racemates was also achieved by them through a bridgehead lithiation strategy using chiral bislithum amides.^115^ Marazano and coworkers applied this important strategy to achieve the same target (clusianone) starting from a different substrate, a hindered TMS enol ether (215) of 2,6-diprenyl cyclohexanone (214). This α,α′-disubstituted TMS enol ether (215) reacted smoothly with malonyl dichloride and produced the desired bicycle 216 along with another structurally complex unexpected bicyclo[3.3.1]nonane product 217.^116^ Subsequently, 216 was transformed into clusianone in a few synthetic steps (Scheme 45).^117^ The results were also similar with a more hindered TMS enol ether 218. When 218 was annulated with malonyl dichloride through Effenberger-type cyclization using BF_3_·Et_2_O, the desired bicycle 219 was produced in moderate yields along with another tricyclic derivative 220 (Scheme 46).^118^

**Scheme 47:**
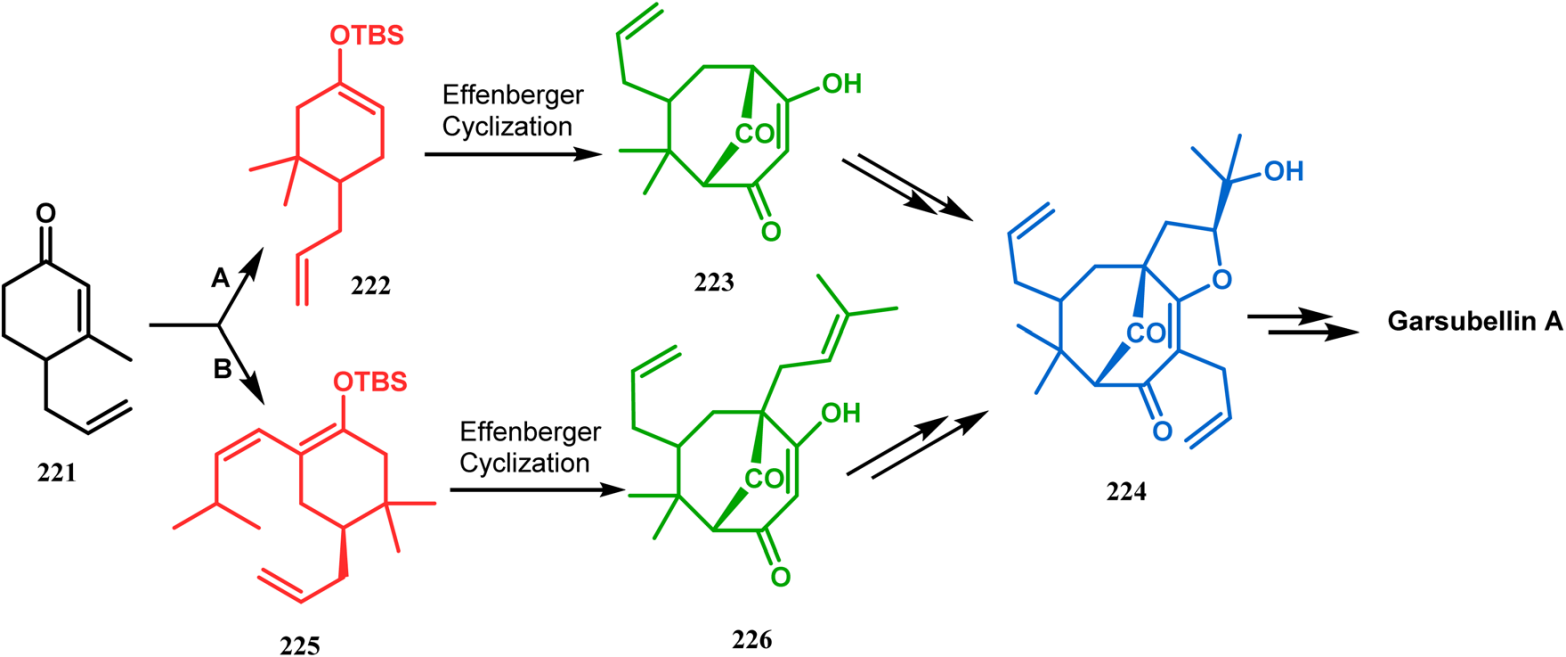
Synthesis of various PPAPs following Effenberger-type cyclization *via* the formation of Danishefsky's bicyclic intermediate.

Simpkin's group reported another detailed study on the efficacy of Effenberger-type cyclization in the total synthesis of PPAPs such as garsubellin A, clusianone, and nemorosone. Their target was to synthesize the Danishefsky's bicyclic intermediate 224 in fewer but effective synthetic steps from the TBS enol ether 222 (route A).^104,105^ Employing the Effenberger cyclization, they then achieved 224 in the shortest route but with a modest yield (Scheme 47).

**Scheme 48:**
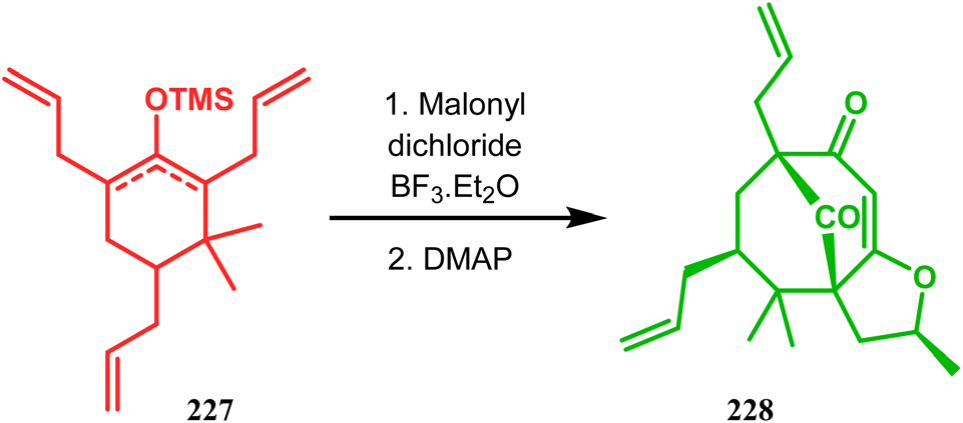
Synthesis of the bicyclo[3.3.1]nonane-2,4,9-trione system following the Effenberger cyclization technique.

In a modified approach, they minimized the problems with yield-reducing nonstereoselective side-chain reactions, introduced the *C*-5 prenyl group prior to Effenberger cyclization (route B), and accessed 224 from a different TBS enol ether 225 through the cyclization product 226 (Scheme 47).^15*c*^ Apart from natural product synthesis, the Effenberger cyclization technique is well explored in the synthesis of other bicyclo[3.3.1]nonane cores. For example, T. J. Blacklock and coworkers described another application of this methodology to synthesize the bicyclo[3.3.1]nonane-2,4,9-trione system 228 from TMS enol ether 227 (Scheme 48).^119^

#### 3.1.5. Visible light-driven cyclization

**Scheme 49:**
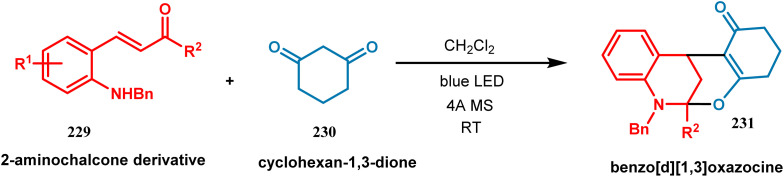
Synthesis of polycyclic benzo[*d*][1,3]oxazocine from 2-aminochalcone derivative upon irradiation of blue LED at room temperature.

The use of visible light for the synthesis of chemical compounds in the presence of photoredox catalysts has gained incredible success owing to the growing demand for green and sustainable chemistry. A photoredox catalyst is very proficient in transferring light energy to the reacting molecules *via* a redox neutral pathway. In this synthetic method, single electron transfer (SET) takes place and, thereby, the molecules can be activated depending on the distinctive mode of activation by the photoredox catalyst. Weiqing Xie and coworkers performed the tandem cycloisomerization of 2-aminochalcone (229) with bifunctional nucleophiles in the presence of visible light. This cascade process was accomplished by the irradiation of blue LED at room temperature, which helped to synthesize a structurally-diverse benzo[*d*][1,3]oxazocine scaffold (231). Benzoxazocine belongs to a family of molecules where both oxygen and nitrogen atoms are embedded in an eight-membered ring, which displays some crucial pharmaceutical properties such as antithrombotic, analgesic, antioxidant, and anticancer activities. Upon irradiation of visible light, 2-amiochalcone took part in tandem *E*–*Z* isomerization to yield an intermediate quinolinium molecule, which was subsequently involved in cascade nucleophilic addition as well as cyclization and thus converted to polycyclic benzo[*d*][1,3]oxazocine (231) in 99% yield (Scheme 49).^120^

**Scheme 50:**
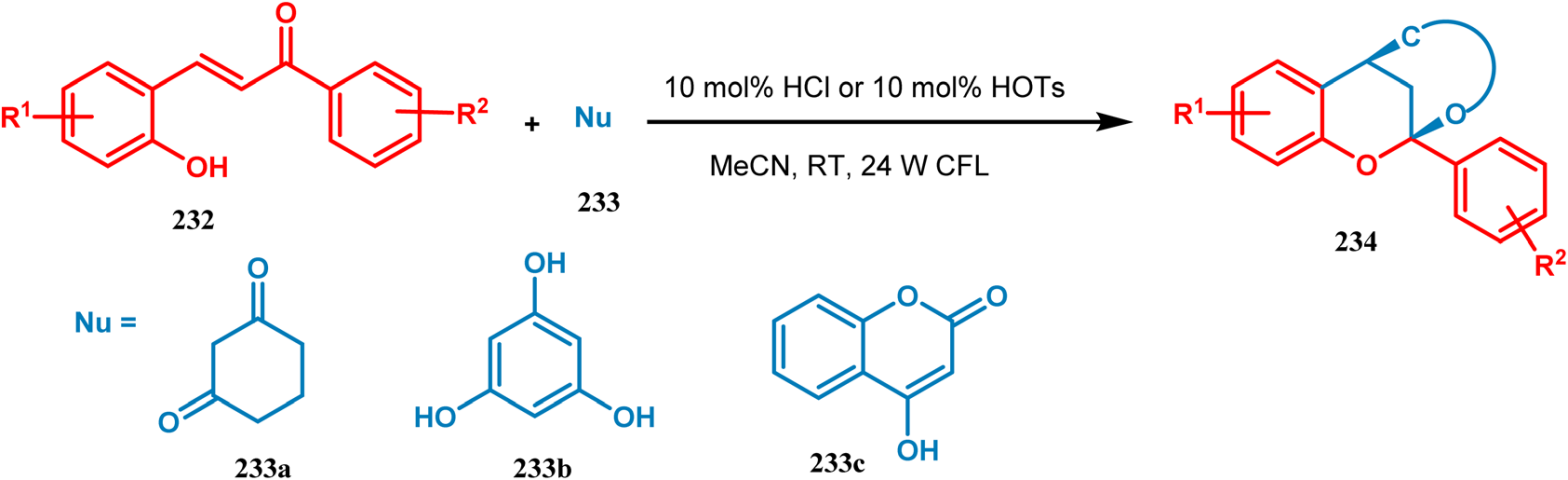
Synthesis of bioinspired hybrid flavonoids from 2-hydroxychalcone upon irradiation of 24 W CFL.

Xie's group also prepared bioinspired hybrid flavonoids from 2-hydroxychalcone upon irradiation of 24 W CFL with a great yield in the presence of a Brønsted acid. The reaction was proposed to occur through tandem double-bond isomerization and then the dehydration cyclization process of 2-hydroxychalcone (232), giving rise to the flavylium cation, which is transferred to hybrid flavonoids (234) by the attack of nucleophiles *in situ* with a yield of 33–99% (Scheme 50).^121^

#### 3.1.6. Catalytic asymmetric synthesis

**Scheme 51:**
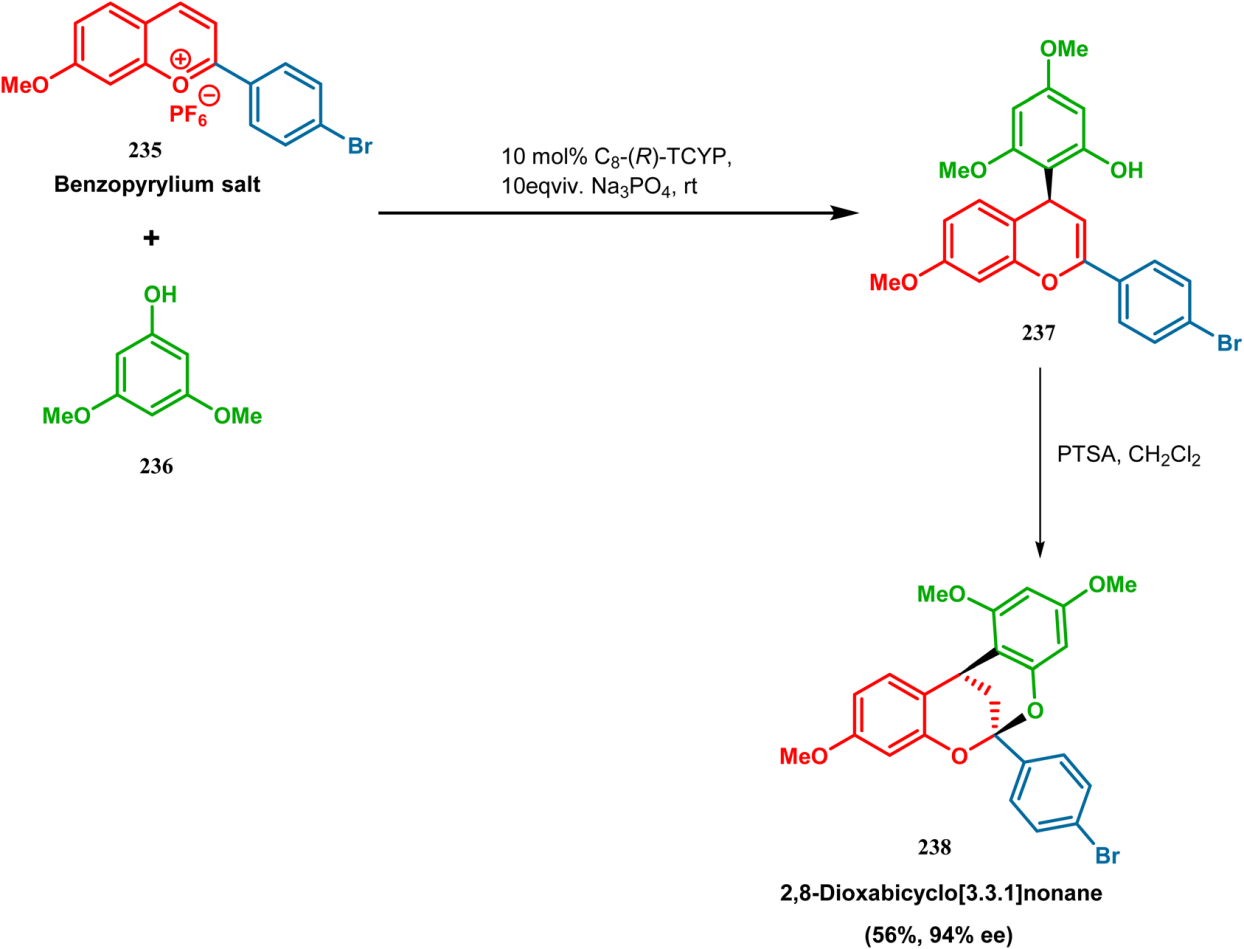
Synthesis of 2,8-dioxabicyclo[3.3.1]nonane by the reaction between benzopyrylium salt and 3,5-dimethoxyphenol in the presence of a chiral catalyst.

F. D. Toste and coworkers performed a reaction betweem benzopyrylium salt (235) and 3,5-dimethoxyphenol (236) in the presence of chiral anionic catalyst, where the product was subjected to acid-catalyzed cyclization to construct 2,8-dioxabicyclo[3.3.1]nonane (238) skeleton in 56% yield and 94% ee (Scheme 51). It is noteworthy that this moiety is found in various biologically active natural products.^122^

**Scheme 52:**
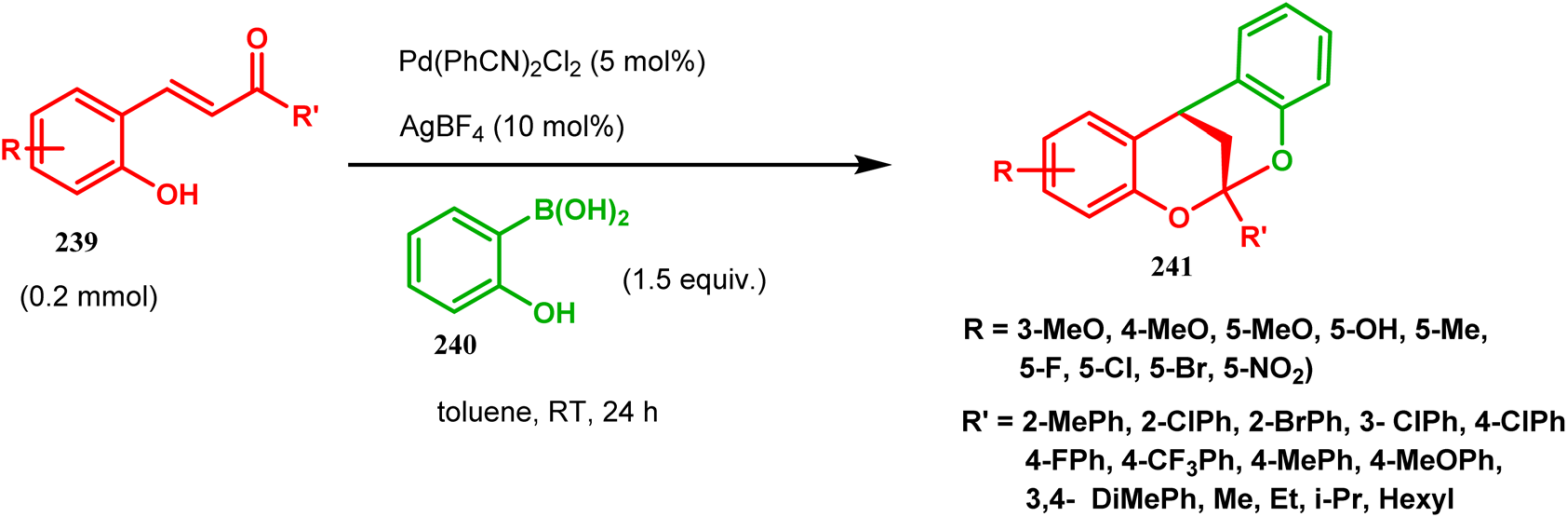
One pot asymmetric synthesis of enantioselective [3.3.1] bicyclic ketals from 2-hydroxyphenylboronic acid.

[3.3.1] Bicyclic ketals are an important framework in many biologically important natural products. Few [3.3.1] bicyclic ketals containing compounds such as diinsininol and diinsinin are regarded as effective inhibitors of platelet-activating factor (PAF) that prompt exocytosis compared to the famous PAF antagonist ginkgolide BN 52021. Proanthocyanidin A2 exhibit *in vitro* selective antiviral efficiency toward the canine distemper virus (CDV) in comparison to ribavirin, thereby making it applicable as a potential anti-CDV compound restricting the replication of that particular virus. Ephedrannin B is also exhibits antiinflammatory effects and subdues the transcription of necrosis factor-a (TNF-a) of the tumor. Therefore, Shi's group was inspired to synthesize chiral heteroannular ketals, which is a great challenge to the organic chemists. They overcame this challenge by accomplishing the reaction in the presence of Pd(ii) catalyst and were able to construct highly enantioselective [3.3.1] bicyclic ketals from 2-hydroxyphenylboronic acid (240) and enone 239 in one pot through the asymmetric cascade reaction (Scheme 52).^123^

#### 3.1.7. Organometallic approaches

**Scheme 53:**
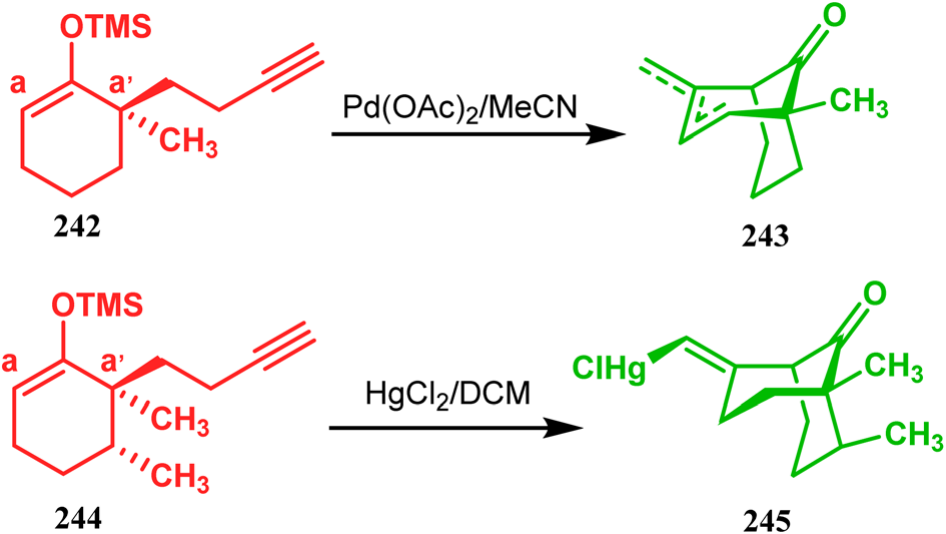
Synthesis of bicyclic core by palladium acetate-mediated annulation of α-(3-alkenyl)-tethered cyclohexanone TMS-enol ether.

Organometallic chemistry has always been used as a powerful tool to achieve synthetic targets with selective stereocontrolled outcomes. Thus, its use to realize complex bicyclo[3.3.1]nonane cores of both natural and nonnatural entities has gained much importance in recent years. The palladium-catalyzed cycloalkenylation technique, developed by Kende^118,119^ and Saegusa,^124^ is well explored in this regard. One such example is the palladium acetate-mediated annulation of α-(3-alkenyl)-tethered cyclohexanone TMS-enol ether 242 to a regioisomeric mixture of bicycle 243 (Scheme 53). The issue of stereo- and regioselectivity was addressed by Drouin's group in their intramolecular carbomercuration strategy.^125^ When this tool was applied on the related TMS-enol ether 244, the bridged bicycle 245 was obtained in regio- and stereochemically pure form (Scheme 53). This methodology, especially the exocyclic vinylmercurial cyclization, witnessed wide application to synthesize diversely-functionalized alkenes.^126–130^ For example, the strategy was utilized to form the key bicycle 245,^11*c*^ which in a few synthetic steps was converted into the targeted sesquiterpenes trifarienols A and B by Forsyth's group.

**Scheme 54:**
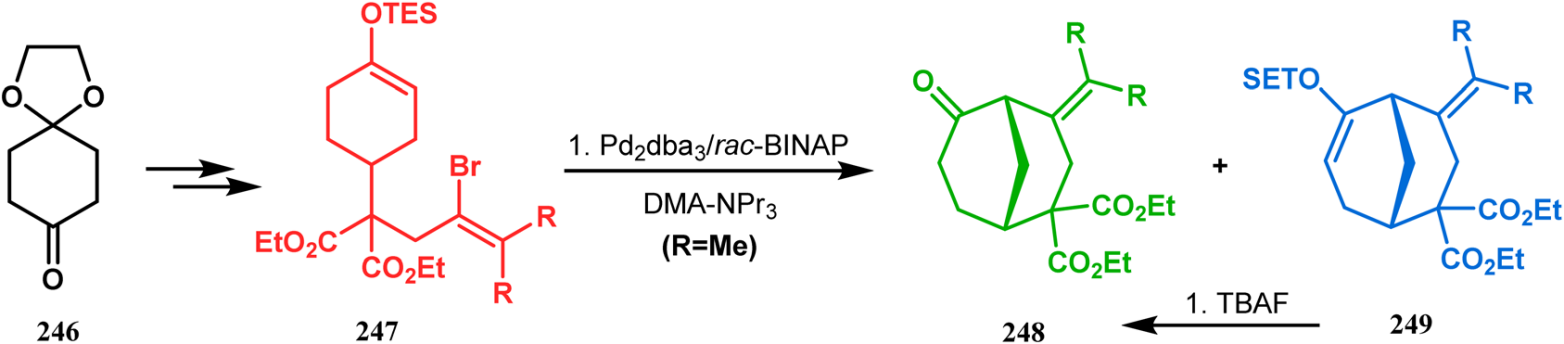
Synthesis of exocyclic double bonds containing bicyclic cores through the intramolecular alkenylation technique.

A similar intramolecular alkenylation technique to synthesize such exocyclic double bonds containing bicyclic cores was also unveiled by Honda's team very recently. They synthesized the annulation precursor 247 (yield 89–93%) from commercially available monoprotected 1,4-cyclohexanedione (246) using literature procedures and subjected the TES enol ether to Pd_2_dba_3_-catalyzed intramolecular annulation to obtain the desired bicycle 248 along with the intermediate TES enol ether 249, which was converted to 248 in good yields (90–99%) using TBAF (Scheme 54). However, the yield decreases substantially when R=H, probably due to facile H–Pd elimination of active hydrogens.^131^

**Scheme 55:**
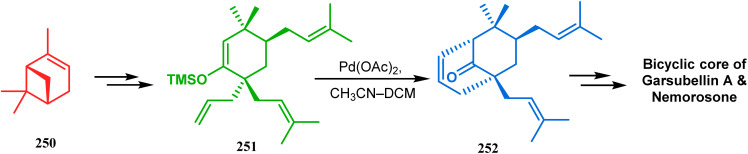
Synthesis of bicyclo[3.3.1]nonane framework of garsubellin A and nemorosone through the intramolecular alkenylation technique.

Another intramolecular alkenylation approach to construct the bicyclo[3.3.1]nonane framework was unveiled by Mehta's group. Their idea was to employ α-pinene (250) as the chiron to ensure the desired stereochemistry of the resulting bicyclic core. At the outset, the TMS enol ether 251 was synthesized from 250 in a sequential manner and subjected to Kende's intramolecular alkenylation condition^118,119^ to form the bicycle 252 in moderate yield (Scheme 55), thereby ensuring a successful enantiospecific route toward the appropriately functionalized bicyclic core of garsubellin A and nemorosone.

**Scheme 56:**
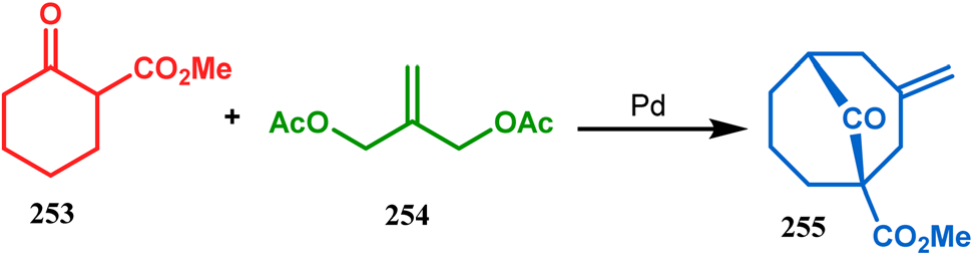
Synthesis of bicyclo[3.3.1]nonenone through the Pd-mediated fusion of cyclohexanone derivative with 2-methylene-1,3-propanediol diacetate.

**Scheme 57:**
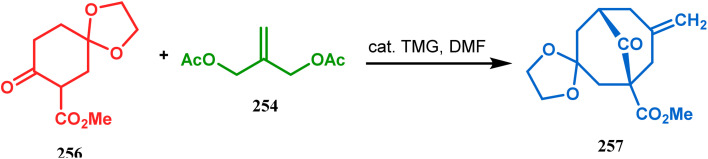
Synthesis of bicyclo[3.3.1]nonane through Pd(OAc)_2_-catalyzed cycloalkylation.

However, the application of palladium chemistry is not limited to intramolecular alkenylation procedures. An intermolecular strategy was also developed by Sivaramakrishnan and coworkers. Their approach toward targeted bicyclo[3.3.1] nonenone 255 employs the Pd-mediated fusion of cyclohexanone derivative 253 with 2-methylene-1,3-propanediol diacetate (254), forming 255 in high yields (Scheme 56).^132^ Besides, an identical Pd-catalyzed cycloalkylation strategy was also demonstrated by Tuckmental's group, which utilizes the Pd(OAc)_2_-catalyzed annulation of 256 with 254 to produce bicyclo[3.3.1]nonane 257 (Scheme 57).^10*d*^

**Scheme 58:**
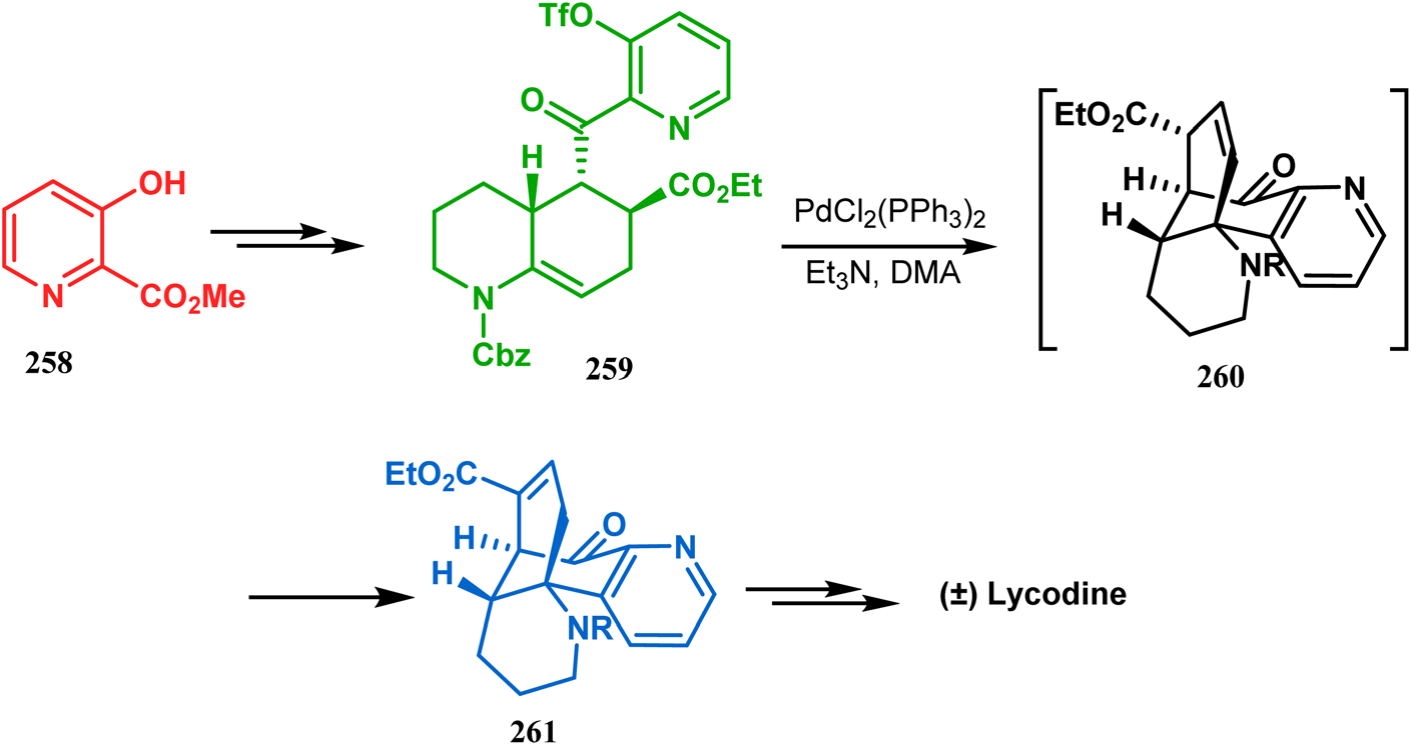
Palladium-catalyzed total synthesis of lycodine from 3-hydroxypicolinate having a bicyclic core.

Such a simple palladium-catalyzed technique has been very recently applied by Hirama and Tsukano's group during the total synthesis of lycodine. Starting from 3-hydroxypicolinate (258), they achieved the annulation precursor 259 in a few synthetic steps. With the required precursor in hand, the palladium-catalyzed Mizoroki–Heck cyclization was attempted, resulting in the formation of the desired bicycle 261 through a 6-*exo-trig* product 260 with only 18% yield. The deactivation of the palladium center by chelation through the pyridyl ketone moiety was attributed to such a low yield of 261, which was cleverly bypassed utilizing a high dilution condition (Scheme 58).^10*e*^

**Scheme 59:**
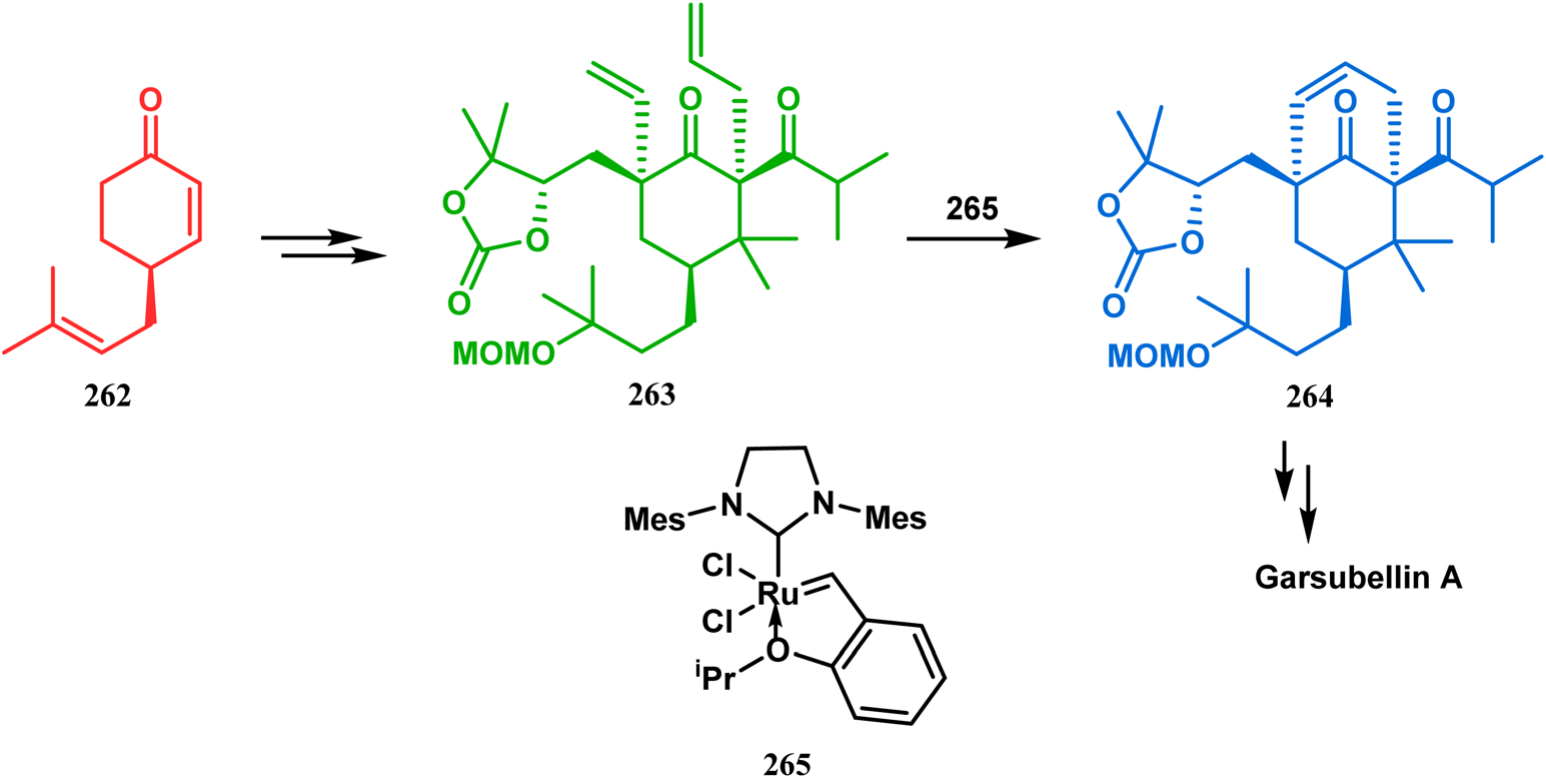
Construction of the bicyclo[3.3.1]nonane core of garsubellin A using ring-closing metathesis (RCM) in the presence of the Hoveyda–Grubbs catalyst.

**Scheme 60:**
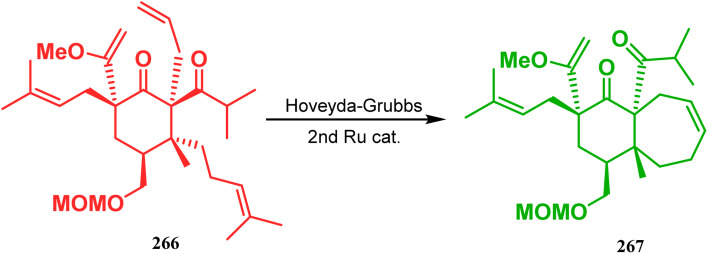
Ring closing metathesis reaction between the terminal vinyl and prenyl groups in the presence of the Hoveyda-Grubbs catalyst.

**Scheme 61:**
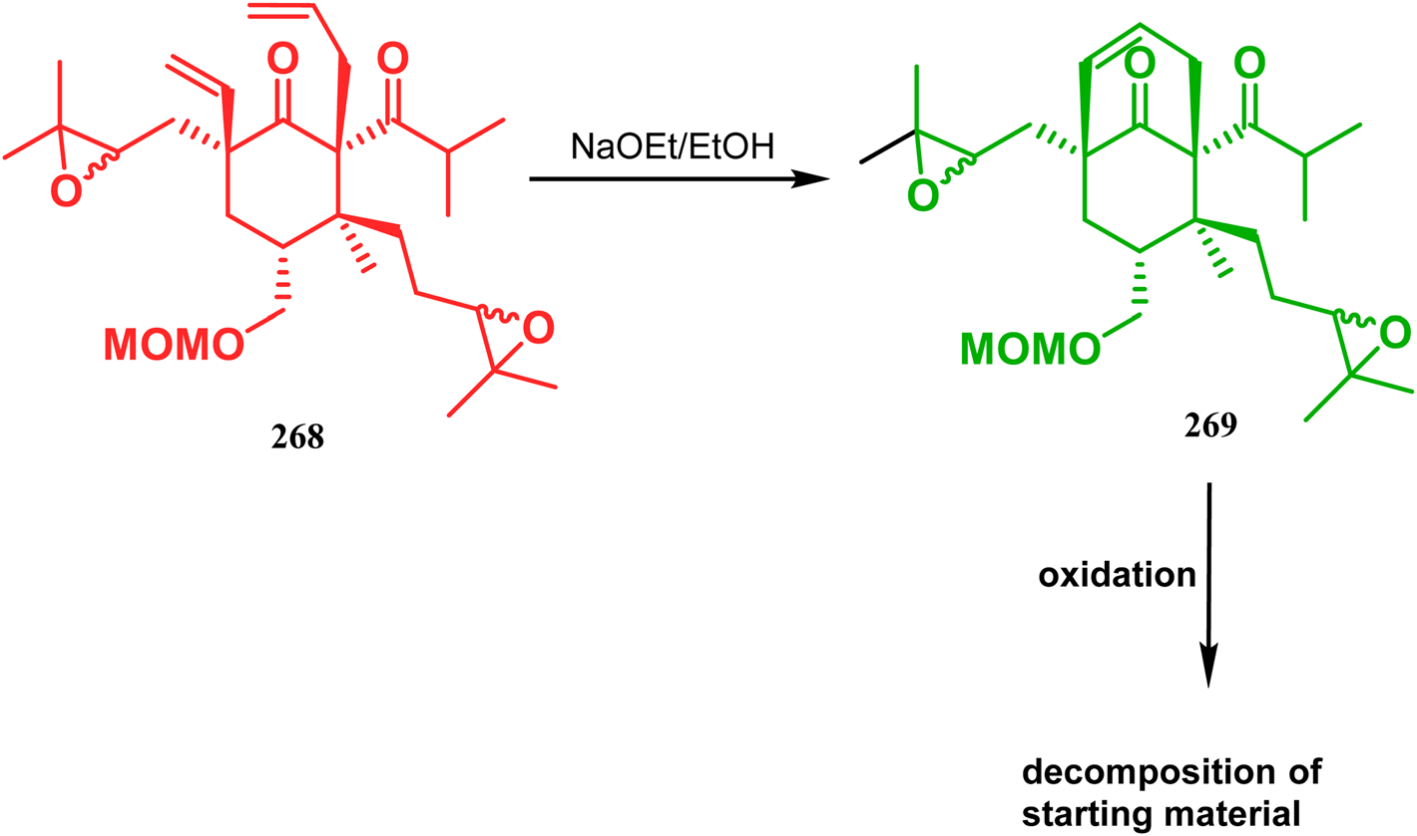
Synthesis of the bicyclo[3.3.1]nonane core of hyperforin through base-promoted intramolecular aldolization.

Inspite of such wide variety of palladium-catalyzed strategies, ruthenium and platinum chemistries have also proved their own efficacy to construct bicyclo[3.3.1]nonane cores. One such successful application of Ru catalysis was demonstrated by Shibasaki's group for the total synthesis of racemic garsubellin A^133^ using ring closing metathesis (RCM) through the Hoveyda–Grubbs catalyst. The necessary cyclohexanone derivative 263, with *cis*-alkene groups at the α and α′ positions of the ketone, was synthesized from enone 262, and they envisioned ring-closing metathesis using catalyst 265, which was then applied to achieve the bicycle 264 in 92% yield. Sequential allylic oxidation, oxidative cyclization, prenyl regeneration, and Stille coupling then led to the formation of racemic garsubellin A (Scheme 59). However, this strategy did not work for the synthesis of similar bicyclic core of hyperforin due to the presence of excess prenyl groups, leading to a ring-closing metathesis reaction between the terminal vinyl and prenyl groups of 266 to form 267 (Scheme 60),^134^ which prompted the author to change their route, leading to the synthesis of the Hoveyda–Grubbs precursor 268. Although Hoveyda–Grubbs catalysis was successful in constructing bicycle 269 in 74% yield (Scheme 61),^135^ the next steps led to the decomposition of the starting materials and again forced the authors to change their strategy. Finally, the authors achieved enantiopure hyperforin through a base-promoted intramolecular aldolization technique.

**Scheme 62:**
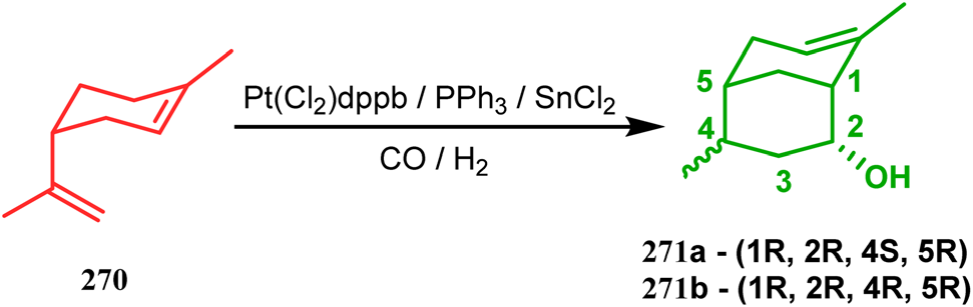
Synthesis of 4,8-dimethyl-bicyclo[3.3.1]non-7-en-2-ol through the hydroformylation/cyclization reaction of limonene in the presence of platinum-tin combined catalyst.

Another such nonpalladium catalysis was reported by Gusevskaya's group during the one-pot access toward 4,8-dimethyl-bicyclo[3.3.1]non-7-en-2-ol (271). Their strategy to synthesize the targeted bicycles (due to their potentiality to be used as perfumes) utilized a simple hydroformylation/cyclization reaction of limonene (270) through a platinum-tin combined catalysis (Scheme 62).^136^

#### 3.1.8. Radical cyclization

**Scheme 63:**
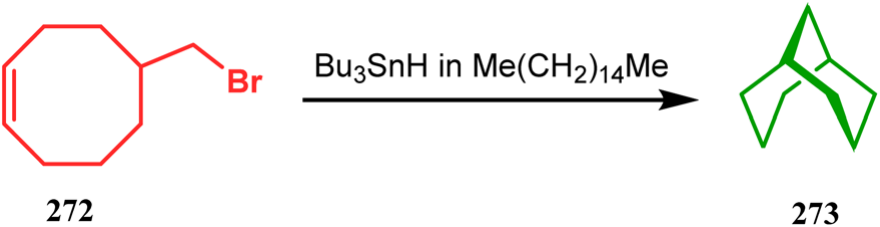
Synthesis of bicyclo[3.3.1]nonane through the transannular radical cyclization of cyclooctenylmethyl bromide with the help of Bu_3_SnH in Me(CH_2_)_14_Me.

**Scheme 64:**
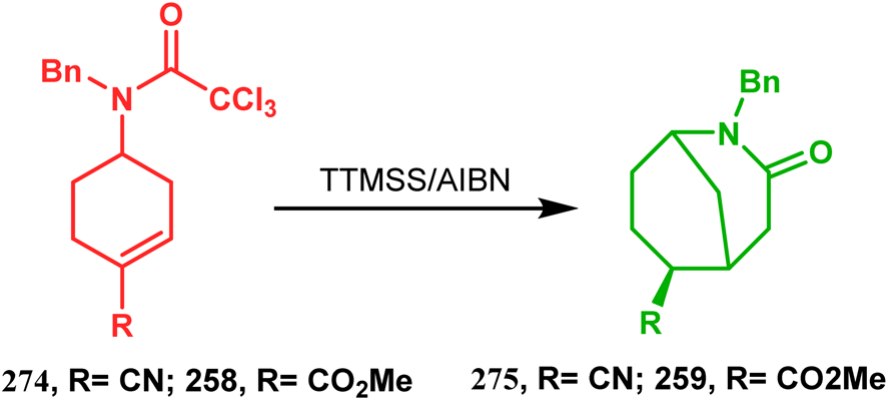
Synthesis of 2-azabicyclo[3.3.1]nonane through a radical ring closing reaction in the presence of the (SiMe_3_)_3_SiH/AIBN catalyst.

The fascinating chemistry of radicals, unlike carbocations or carboanions, has always attracted the attention of chemists and prompted to explore it in the synthesis of structurally unique cyclic cores. Bicyclo[3.3.1]nonanes are also not an exception. The pioneering and probably the first report in this category appeared in 1987 due to Finlay and Walton.^137^ They described a transannular radical cyclization of cyclooctenylmethyl bromide (272) to achieve bicyclo[3.3.1]nonane (273) by irradiating a solution of 272 and Bu_3_SnH in Me(CH_2_)_14_Me (Scheme 63). However, a more practical approach and detailed advancement in this area was made by Bonjoch's group and, after a decade of Finlay and Walton's report, they unveiled a new route toward 2-azabicyclo[3.3.1]nonane through a radical ring closure method. Their initial report was a (SiMe_3_)_3_SiH/AIBN-mediated radical cyclization of amidocyclohexene 274 to give the desired bicyclic nitrile (275) in good yields (57–70%) (Scheme 64). The corresponding bicyclic carboxylate (277) was obtained with even a better yield when amidocyclohexane 276 was exposed to an identical reaction condition. The amount of radical mediator has shown to have a high influence on the product yield, corroborated by the fact that while 2 equivalents of TTMSS produces a mirror amount of product along with chloro and dichloro analogues, the use of 3.5 equivalents of the same furnishes the desired products in moderate yields.^138^

**Scheme 65:**
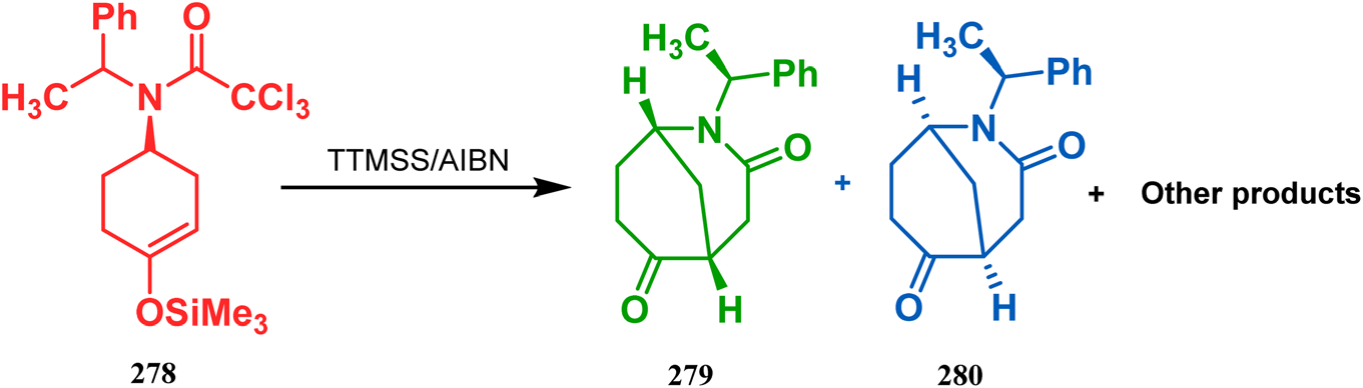
Synthesis of diastereomeric bicycles along with a β-lactam from TMS enol ether in the presence of (SiMe3)3SiH/AIBN.

In a following report by the same group, the TMS enol ether 278 was treated using a similar protocol to form diastereomeric bicycles 279 (yield 10%) and 280 (yield 21%) along with a β-lactam and another undesired bicycle (Scheme 65).^139^

**Scheme 66:**
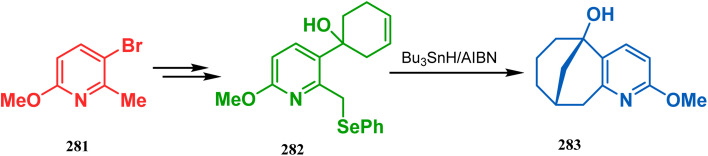
Synthesis of the bicyclic core of Huperzine A through a 6-*exo*-trig radical cyclization in the presence of Bu_3_SnH/AIBN.

The application of radical cyclization is also found in natural product core synthesis. One such report is due to Ward and Caprio. They demonstrated a radical-mediated approach toward the core structure of huperzine A and synthesized the required precursor 282 from a trisubstituted pyridine derivative 281. 282 was then treated with Bu_3_SnH/AIBN, leading to the bicyclic core structure (283) of Huperzine A through a 6-*exo*-trig radical cyclization in 97% yield (Scheme 66).^140,141^

### 3.2. Intra- and intermolecular C–X bond formation

#### 3.2.1. *N*-Cyclization

**Scheme 67:**
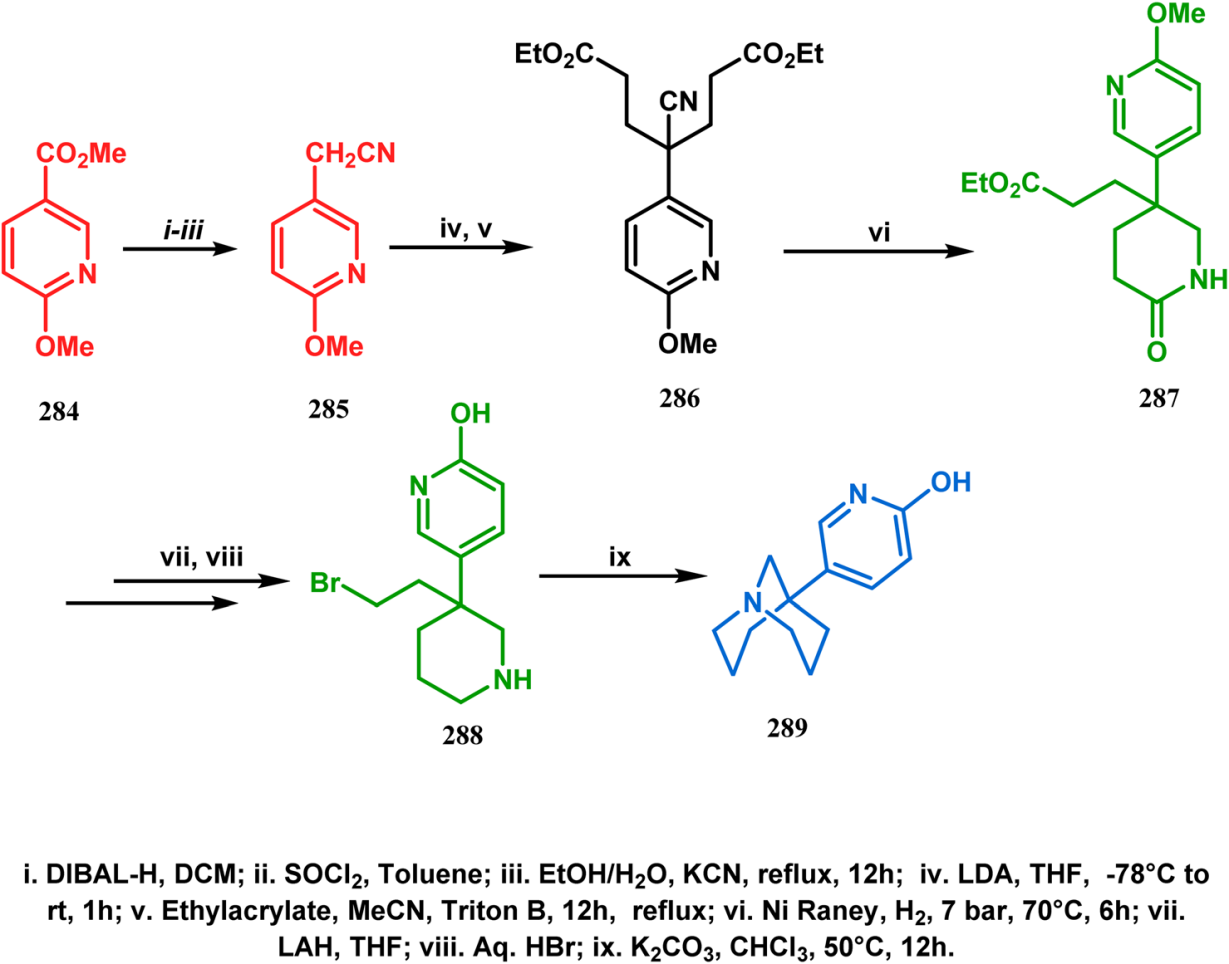
Sequential synthesis of the bicyclo[3.3.1]nonane core through intramolecular alkylation reaction.

Among the several available methods to construct C–X linkage, intramolecular *N*-alkylation in the presence of a base is very well known for synthesizing heterobicyclo[3.3.1]nonane derivatives. Interest in this area was originated from the potentiality of these bicyclononane derivatives to be used as selective α7 nicotinic ligands, inhibitors, *etc.*^142^ Although the synthetic route toward one such entity was reported in as early as the 1950s by Walker's group,^143^ a much elaborate study was done by Slowinski's group very recently.^144^ Their journey commenced from 6-methoxynicotinic acid methyl ester (284), which was converted into 288 in a sequential manner. The alkylation precursor was then treated with potassium carbonate in chloroform, leading to the formation of the desired bicycle 289 in 74% yield through an intramolecular alkylation (Scheme 67).

**Scheme 68:**
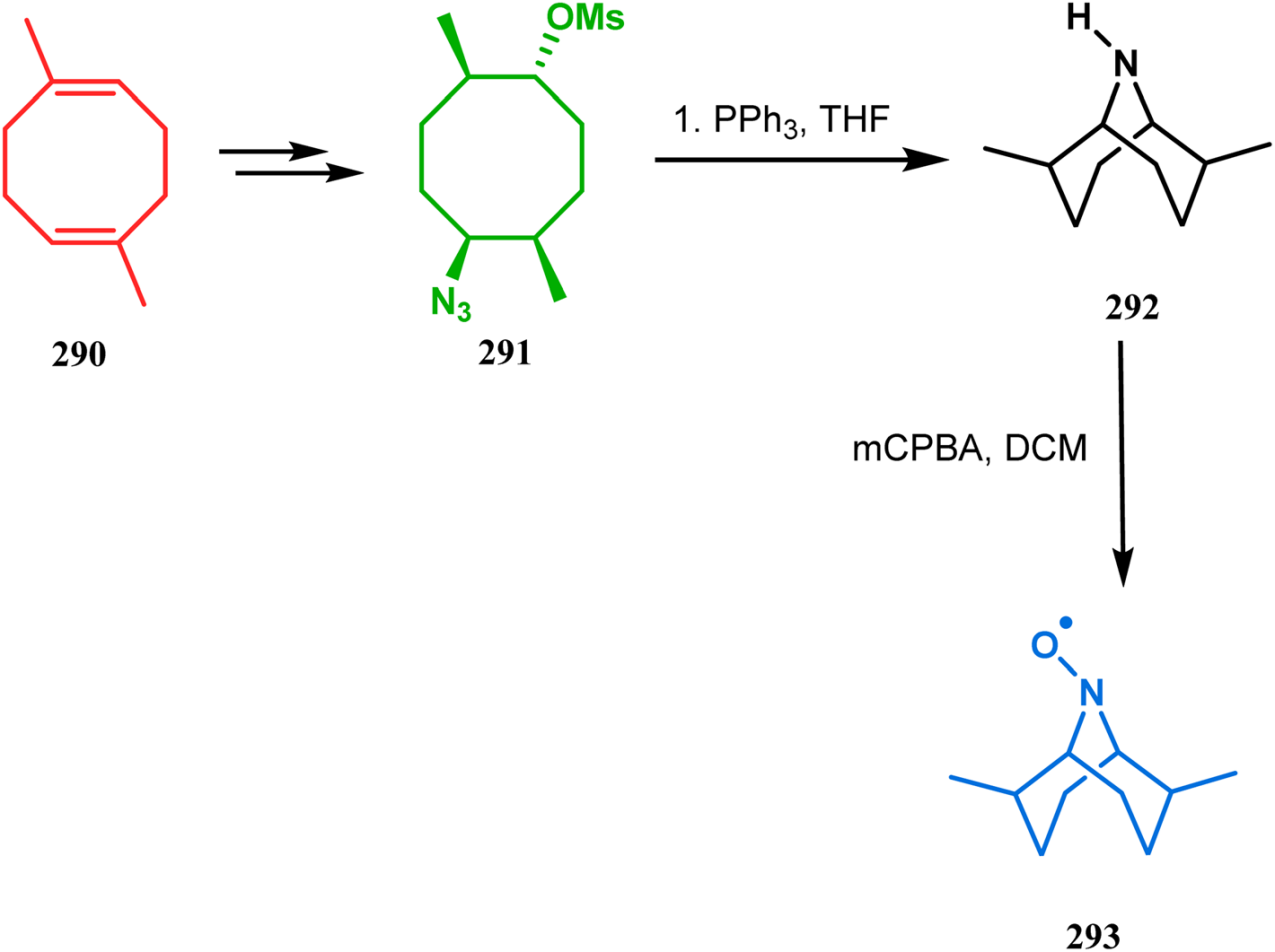
Synthesis of nitroxide through triphenylphosphine-promoted azide reduction and then cyclization, followed by mCPBA oxidation.

**Scheme 69:**
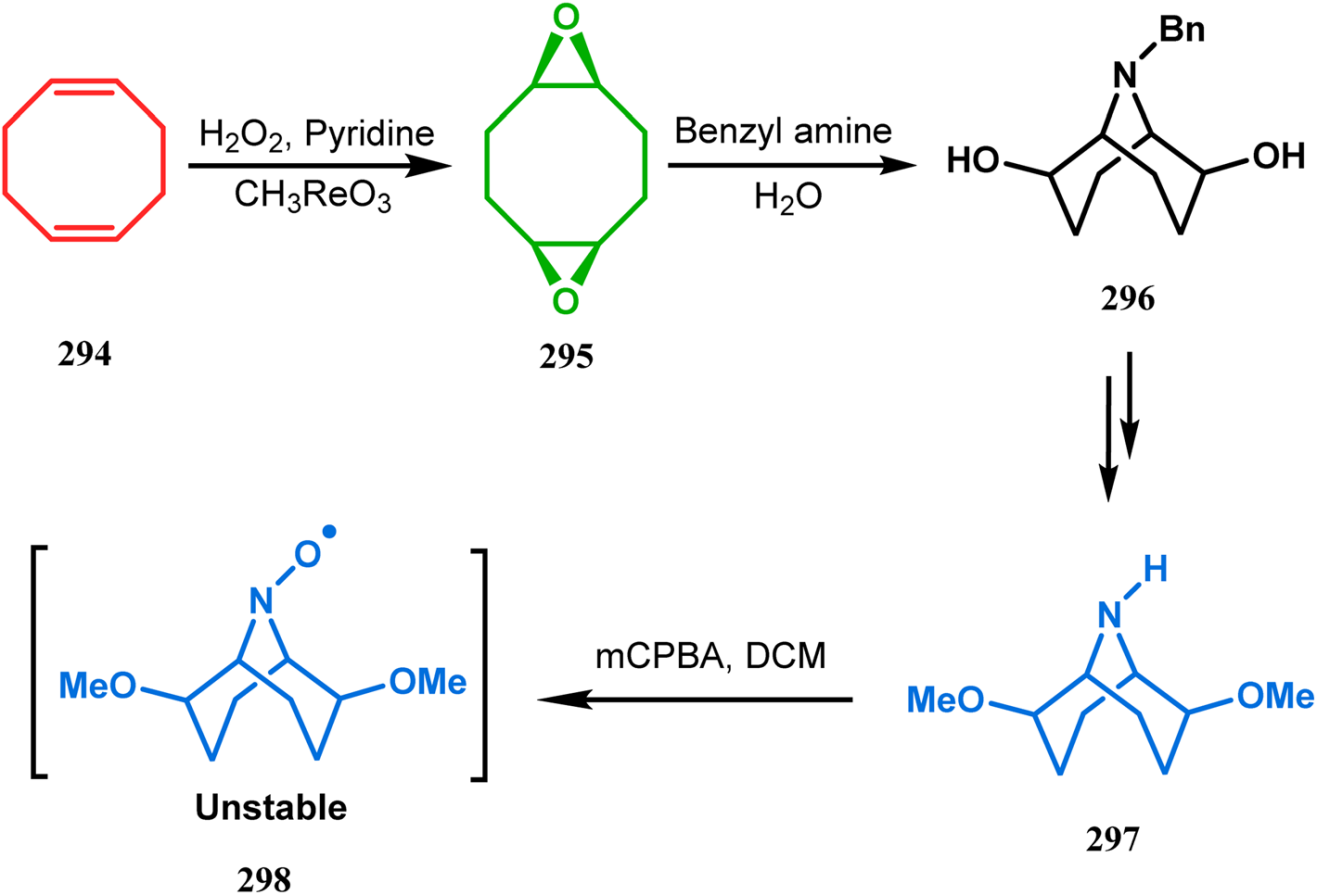
Synthesis of nitroxide through the rhenium-catalyzed hydrogen peroxide oxidation of 1,5-cyclooctadiene, followed by mCPBA oxidation.

The method was then employed to achieve a series of new chemical entities and was proved to be sufficiently potent as α7 nicotinic ligands, having fascinating selectivity compared to the α4β2 nicotinic receptor. A similar intramolecular substitution, resulting in the formation of another class of azabicyclo[3.3.1]nonane, was also reported in the same decade by Rychnovsky and coworkers.^145^ The idea was to access some novel *C*_2_-symmetric nitroxides to be used as enantioselective oxidants. The synthetic route began with the hydroboration-oxidation of 1,5-dimethyl-1,5-cyclooctadiene (290) to produce the corresponding diol, which in a sequential manner was converted to methanesulfonate 291 in 74% yield. Triphenylphosphine-promoted azide reduction and concomitant cyclization of 291 then produced amine 292 (yield 41%), which on mCPBA oxidation led to the desired nitroxide 293 having a yield of 67% (Scheme 68). The synthesis of the corresponding azabicyclo[2.2.1] heptane nitroxide was reported to be far more difficult.^145^ To access a more complex bicyclo[3.3.1]nonane nitroxide, the same author utilized another methodology originally developed by Michel and Rassat.^146^ This study started with the rhenium-catalyzed hydrogen peroxide oxidation of 1,5-cyclooctadiene 294 to furnish diepoxide 295 (yield 50%), which upon reacting with benzyl amine in water produced diol 296 in 91% yield. The attempted oxidation of 297 (yield 91%) remained unsuccessful, and the targeted nitroxide 298 was not isolated due to its instability (Scheme 69).^146^

**Scheme 70:**
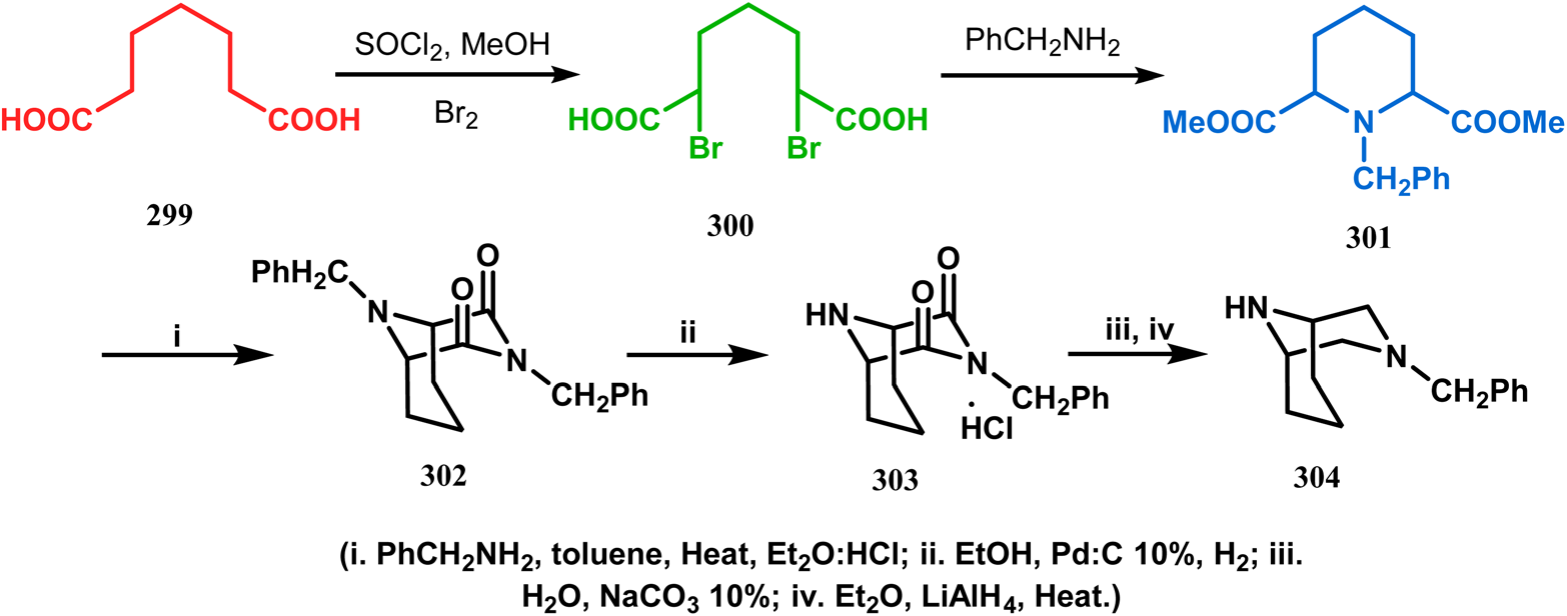
Synthesis of diazabicyclo[3.3.1]nonanes through the α,α′-dibromination of pimelic acid, followed by several condensations of benzylamine.

An identical application of benzyl amine through a different route to achieve diazabicyclo[3.3.1]nonanes was also developed by Cingarella's group. Their target was to study the μ-opioid receptor affinity of properly substituted diazabicyclo[3.3.1]nonanes, and it commenced with the α,α′-dibromination of pimelic acid (299) to give 300, followed by the double condensation of benzylamine to furnish *N*-benzyl-2,6-dicarbomethoxypiperidine (301). This piperidine derivative was then again condensed with the same amine, producing the diazabicycle 304 (Scheme 70).^147^

**Scheme 71:**
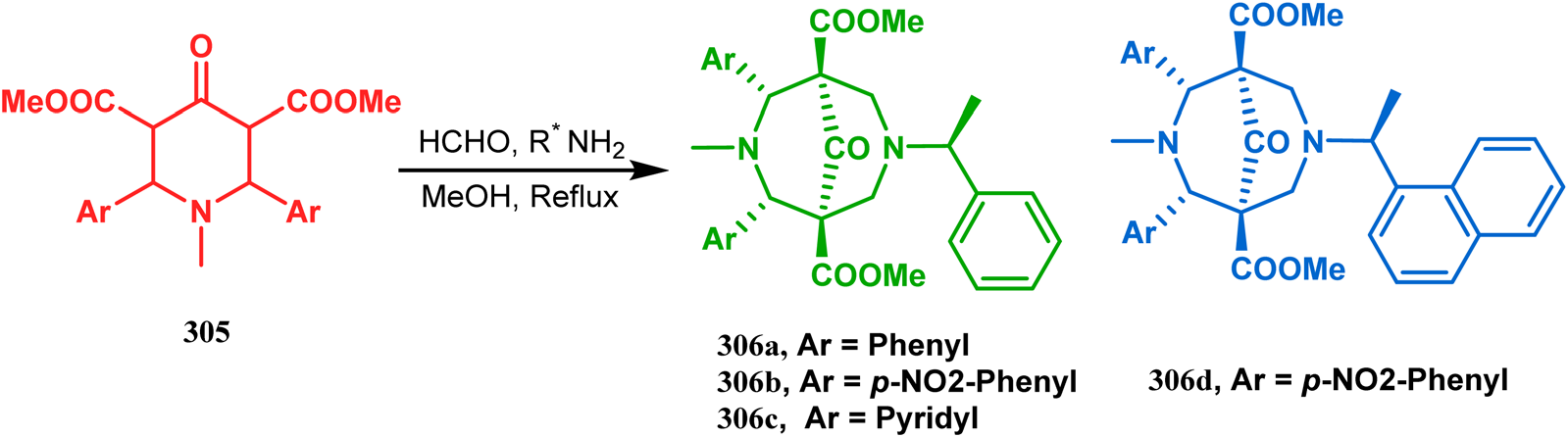
Synthesis of the bicyclo[3.3.1]nonane skeletons through the condensation of piperidine-4-ones with formaldehyde and amines in methanol under refluxing condition.

A similar condensation strtategy of piperidine-4-ones (305) with chiral amines were also utilized to synthesize bicyclo[3.3.1]nonane skeletons. Kuhl's and Sacchetti's teams described one such useful application of chiral amines in the synthesis of new diamino chiral ligands, and their approach involved the condensation of 305 with formaldehyde and amines in methanol under refluxing condition, thereby forming chiral ligands 306a–d (yield 73–92%) (Scheme 71).

**Scheme 72:**
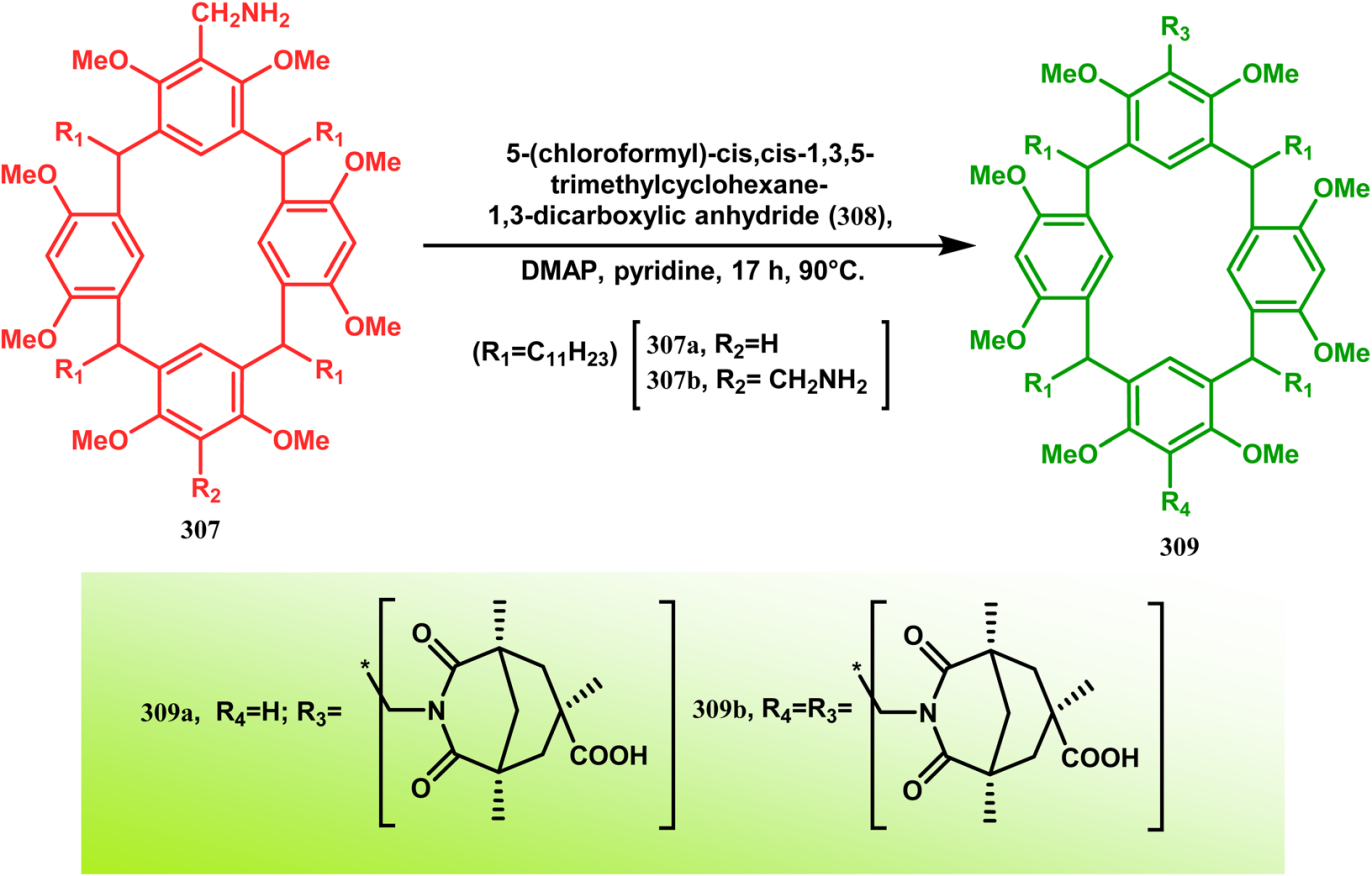
Synthesis of azabicycle through the double condensation of a macrocyclic amine with bicyclic anhydride.

These ligands were then successfully employed in the kinetic oxidative resolution of alcohols.^148,149^ Another application of such double condensation of amines with esters was found in Mattay's report. The employed amine was a macrocyclic amine (307) this time. Thus, when compound 307 was reacted with bicyclic anhydride 308, the double condensation produced the azabicycle 309 (Scheme 72).^150^

**Scheme 73:**
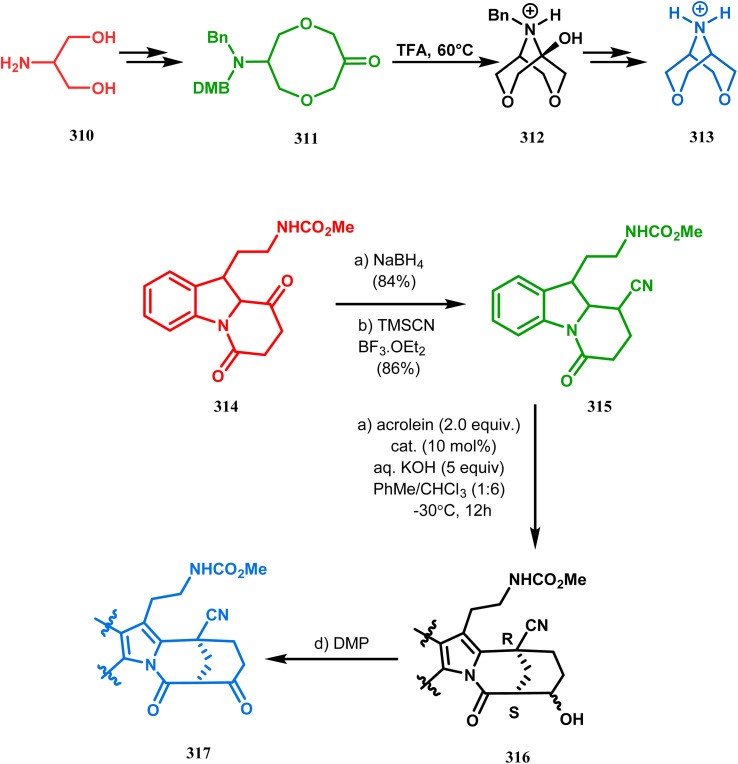
(a)Sequential synthesis of 3,7-dioxa-9-aza-bicyclo[3.3.1]nonane through the intramolecular *N*-cyclization of amine diol in the presence of trifluoroacetic acid (b) synthesis of azabicyclo[3.3.1]nonane moiety using the Michael/aldol cascade reaction.

Intramolecular *N*-cyclization to construct azabicyclo[3.3.1]nonane was also reported by C. M. Park. Beginning with an amine diol 310, the author successfully reached the cyclization precursor 311, which on treatment with trifluoroacetic acid underwent rapid intramolecular cyclization to produce the hemiaminal 312 (yield 80%). The targeted 3,7-dioxa-9-aza-bicyclo[3.3.1]nonane (313) was then synthesized from this hemiaminal in a few synthetic steps having a yield of 99% (Scheme 73a).^151*a*^

Also, F.-S. Han's research group followed the asymmetric Michael/aldol cascade reaction to synthesize the desired compound (317) (Scheme 73b) in good yield with excellent enantiomeric excess by accomplishing the reaction between 314 and 315 in the presence of NaBH_4_ and TMSCN in BF_3_ ether, followed by the reaction with acroleinthe. The yield of the 317 was 56%. It is important to mention that the reaction can be performed at the multigram scale with a considerable yield.^151*b*^

**Scheme 74:**
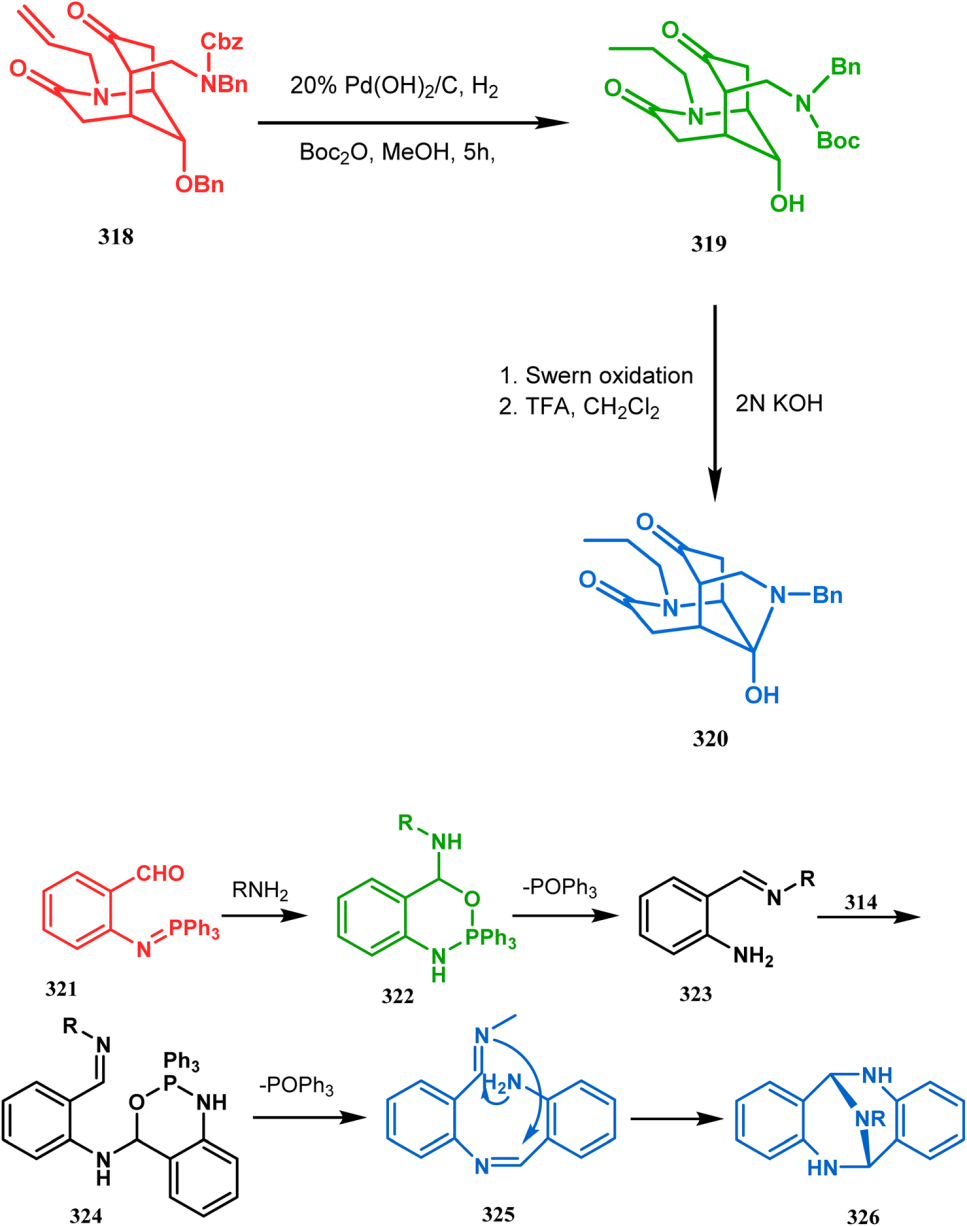
(a) The diastereoconvergent synthesis of the diazatricyclic core moiety (b) synthesis of triazabicyclo[3.3.1]nonane through the *N*-cyclization mechanism.

P.-Q. Huang and coworkers explored the construction of the tricyclic core from compound 318 by subjecting the diastereomer 318 to catalytic hydrogenolysis in the presence of Boc_2_O and they isolated alcohol 319 in 62% yield (Scheme 74a). Then, the alcohol 319 was subjected to a Swern oxidation, followed by treatment with TFA to remove the Boc group. On workup using 2 N KOH, the diazatricyclic core 320 was obtained in 51% yield.^152*a*^

Apart from these aza and diaza analogues of bicyclo[3.3.1]nonane, the triaza analogues are also accessible and, according to Molina's strategy, even the highly unreactive iminophosphorane (321) could be activated toward *N*-cyclization to achieve the triazabicyclo[3.3.1]nonane (326) (Scheme 74b).

**Scheme 75:**
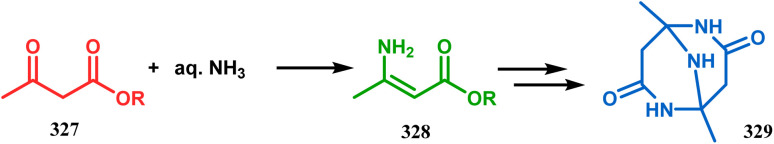
Synthesis of 1,5-dimethyl-2,6,9-triaza-bicyclo[3.3.1]nonane-3,7-dione *via* the ammonolysis of alkyl acetoacetates in water.

Iminophosphorane (321), originally synthesized by Staudinger reaction, constitutes resonance-stabilized chelates due to its reluctance toward both intra- and intermolecular aza Wittig condensation, even though it has the requisite reactive groups for this reaction. However, when 321 was reacted with primary amines in the presence of catalytic amount of acetic acid, amine 323 was formed through intermediate 322. The aniline derivative 323 thus formed then again reacts with iminophosphorane 321 and proceeds toward the cyclization precursor 325 through intermediate 324. Once compound 325 was formed, two consecutive C–N cyclizations furnished the triazabicyclo[3.3.1]nonane derivative 326 in good yields (40–80%).^152*b*^ The synthesis of a similar triaza-analogue (329) is reported by Abonia's group. According to their report, it could be achieved *via* the ammonolysis of alkyl acetoacetates in water.^153^ Thus, when MeCOCH_2_CO_2_Me (327) was treated with aqueous ammonia at room temperature, white crystalline methyl-β-aminocrotonate (328) was found to form rapidly. It was then kept undisturbed for 4 weeks, resulting in the transformation of 328 into another white solid, 1,5-dimethyl-2,6,9-triaza-bicyclo[3.3.1]nonane-3,7-dione (329) (Scheme 75).

#### 3.2.2. *O*-Cyclization

**Scheme 76:**
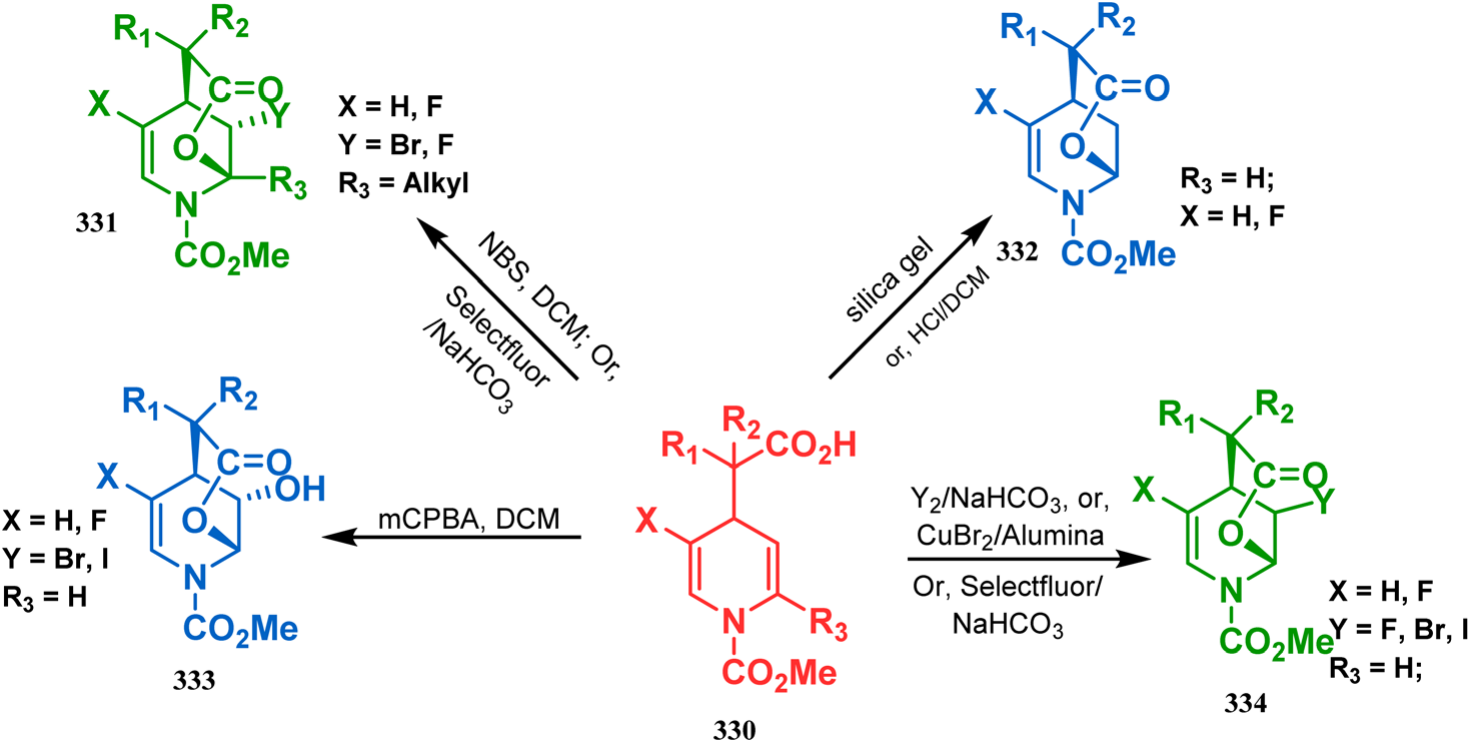
Synthesis of the bicyclo[3.3.1]nonane moiety through the acid-catalyzed intramolecular lactonization of dihydropyridines.

Cyclization through oxygen center is also a useful technique to synthesize heteroanalogues of bicyclo[3.3.1]nonane. Among the few reports available in literature, the acid-catalyzed intramolecular lactonization of dihydropyridines (330) is well investigated. Rudler and coworkers showed that dihydropyridines, which can be obtained from pyridine derivatives, when treated with acids, the appended carboxylic acid group undergo a regioselective intramolecular lactonization reaction and produce oxaza analogues of bicyclo[3.3.1]nonane (332). More interestingly, when these cyclizations were attempted in the presence of halogenated compounds (as Lewis acid), halogens almost unexceptionally get incorporated in the product bicycles (331, 332). Even, when mCPBA was used as the acid catalyst, a hydroxyl group is inserted in the product (333). This cyclization is so facile that even silica gel can catalyze the lactonization procedure (Scheme 76).^154^

**Scheme 77:**
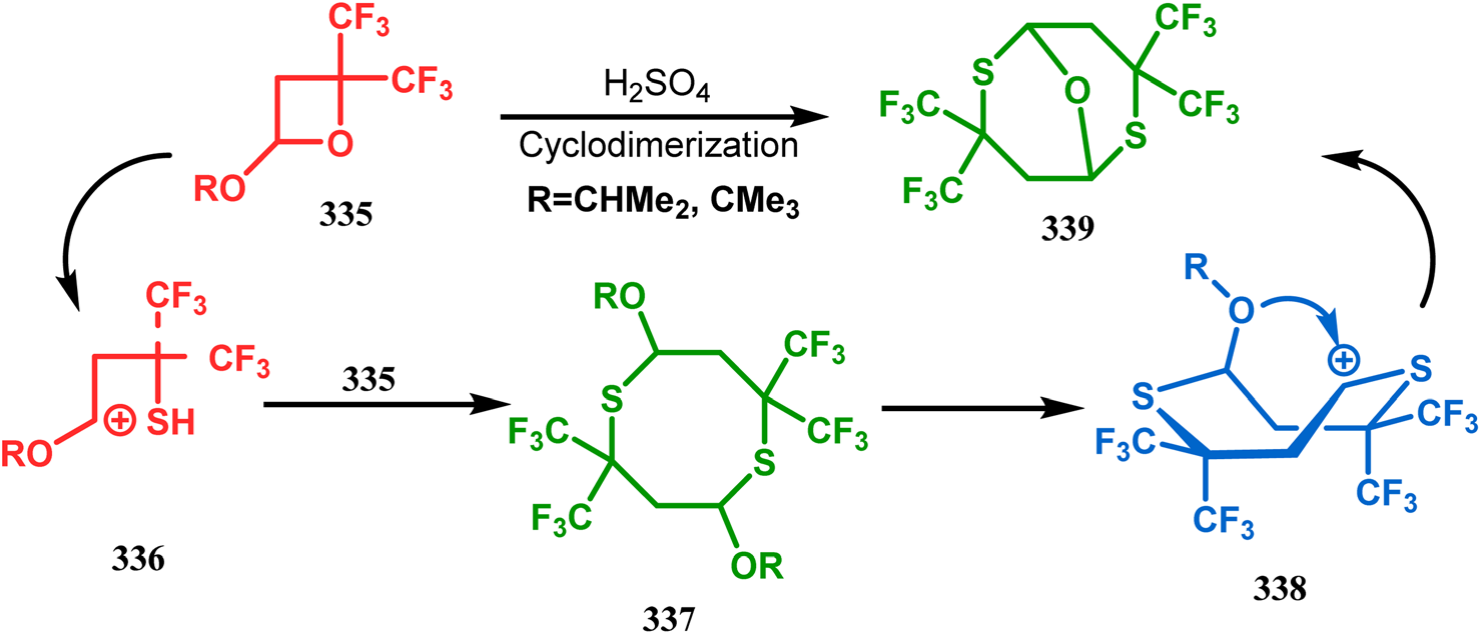
Synthesis of oxa-bridged dithiabicyclo[3.3.1]nonane through the acid-catalyzed cyclodimerization of 2,2-bis-(trifluoromethyl)-4-alkoxy-thietane.

Petrov and Marshall exemplified another type of acid-catalyzed route toward bicyclo[3.3.1]nonane analogues. Their report on an acid-catalyzed dimerization of 2,2-bis-(trifluoromethyl)-4-alkoxy-thietane (335) demonstrated a trouble-free access toward oxa-bridged dithiabicyclo[3.3.1]nonane (339). It is believed that the reaction proceeds through the ring opening of thietane (335) to form a stable carbocation (336), which upon cyclodimerization gives 337. Acid-promoted MeOH elimination then produced carbocation 338, which was intramolecularly captured by the remaining alkoxy group to form the oxa-bridged dithiabicyclo[3.3.1]nonane (339) (yield 35–50%) (Scheme 77).^155^

**Scheme 78:**
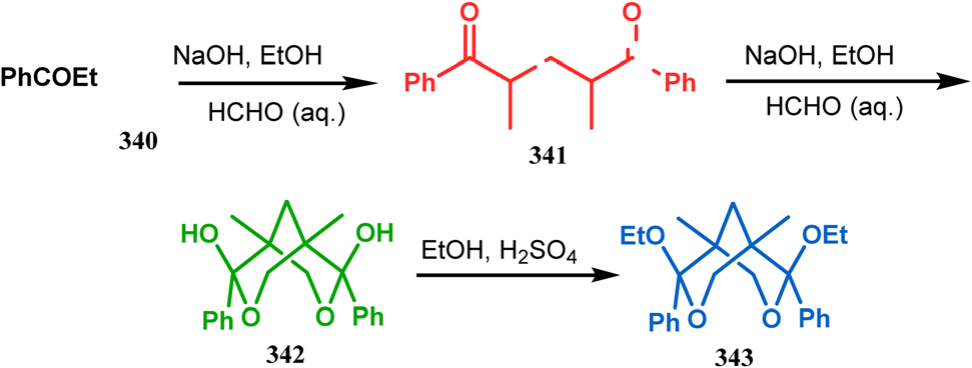
Synthesis of bis-ethoxy analogues from propiophenone.

Apart from these reports, E. J. Corey's route toward chelating bis-ethers and bis-amines is also remarkable. According to his findings, when propiophenone (340) was treated with aqueous formalin solution in the presence of a base, the diketone 341 becomes the major product (yield 95%), which on further reaction with formalin solution transforms into bicyclic bis-hemiketal 342 in 55% yield. This bis-ether on treatment with EtOH/H_2_SO_4_ transforms into the corresponding bis-ethoxy analogues (343) in 90% yield (Scheme 78).^156^

**Scheme 79:**
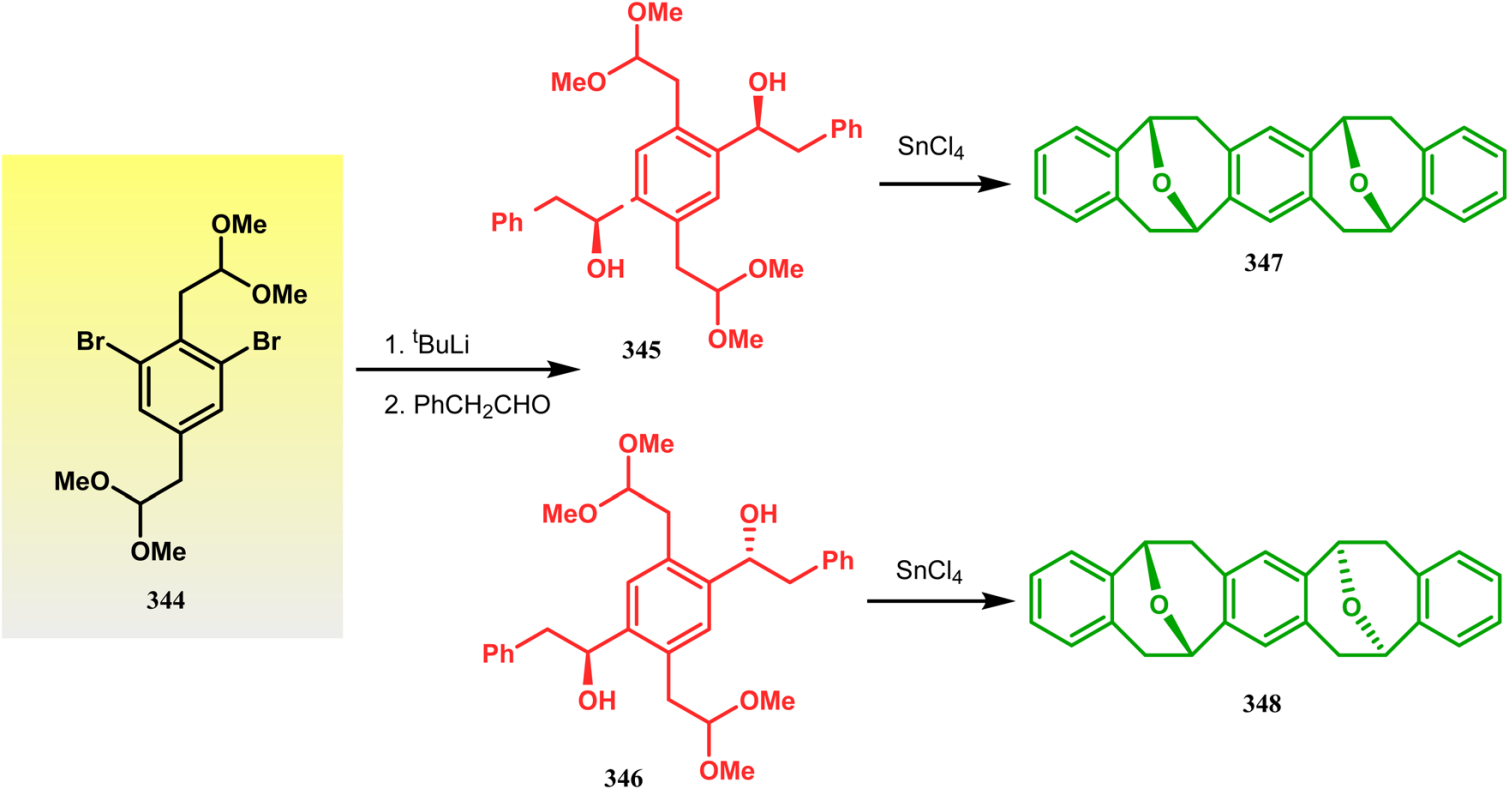
Stepwise synthesis of oxabicyclo[3.3.1]nonane-based molecular tweezers in the presence of butyl lithium and benzyl aldehyde, followed by SnCl_4_-mediated cyclization reaction in DCM.

Harmata and coworkers described an interesting synthesis of molecular tweezers based on the oxabicyclo[3.3.1]nonane framework. Starting from the dibromo derivative 344, the annulation precursors (345, 346) were synthesized by the stepwise reaction of butyl lithium and benzyl aldehyde, which on SnCl_4_-mediated cyclization reaction in DCM yielded the bis-kagan's ethers 347 and 348 in good yields (57% and 75%, respectively) (Scheme 79).^157–159^

#### 3.2.3. Heavy atom-cyclization

**Scheme 80:**
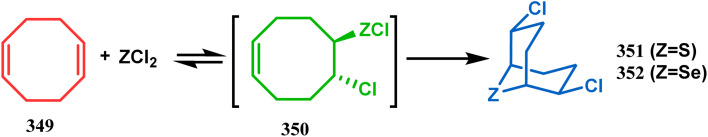
Synthesis of sulphur/selenium-containing bicyclo[3.3.1]nonane through the heavy-atom cyclization method.

Apart from nitrogen and oxygen cyclizations, the ring closure mechanism is also common with heavy atoms such as sulphur and selenium. Weil,^160^ Corey,^161^ and Lautenschlaeger^162^ were the very first authors to discover one such cyclization. They demonstrated that when 1,5-cyclooctadiene (349) was treated with sulphur or selenium dichloride/dibromide, the bicyclic adducts (351) were produced in high yields through an intermediate (350) (Scheme 80).

**Scheme 81:**
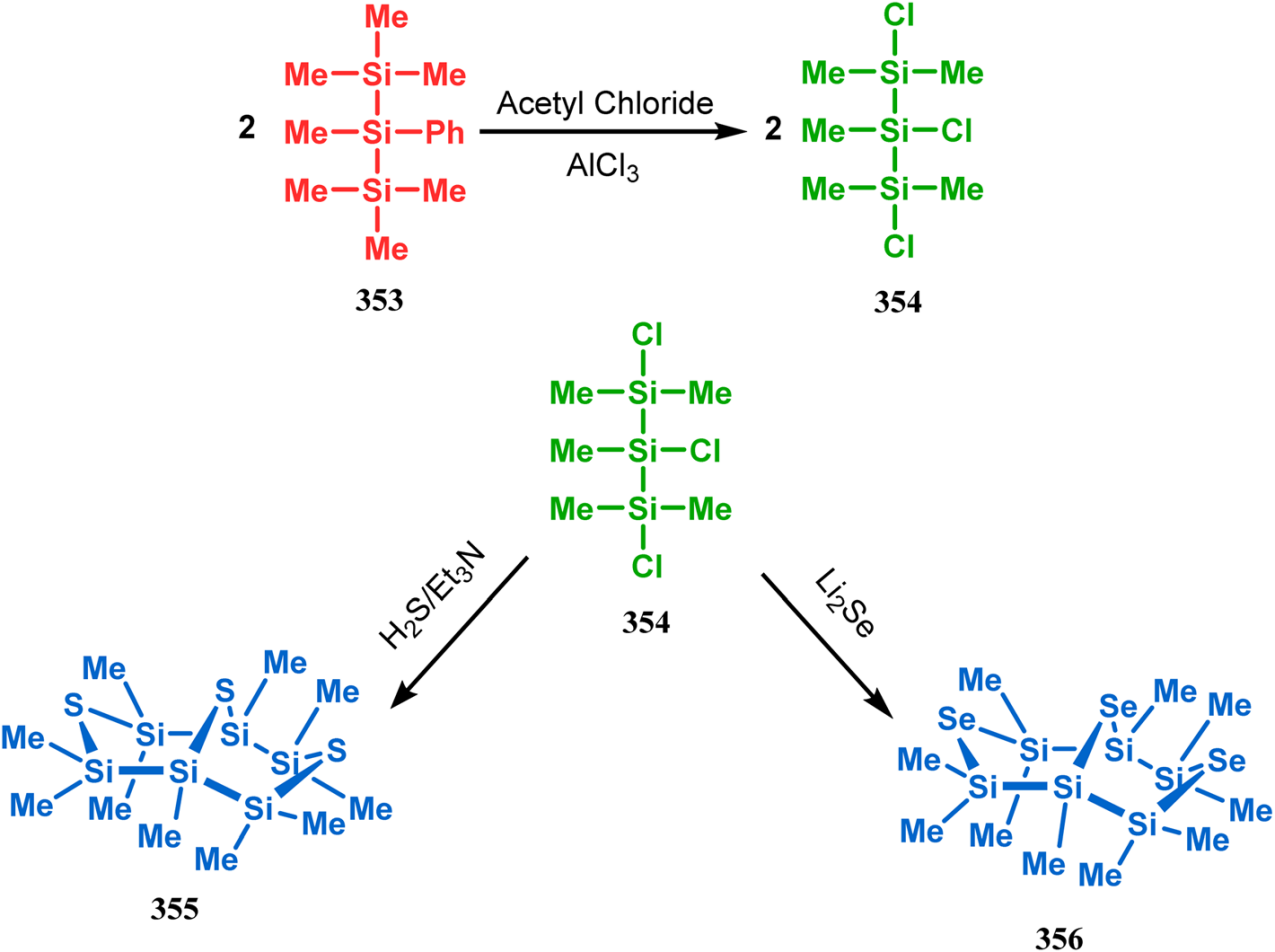
Synthesis of the bicyclo[3.3.1]nonane skeleton in the presence of trimethylamine/H_2_S and in the presence of Li_2_Se.

Such entities, having the donor at the 9th position and the leaving group at the β position to the donor, are known as “WCL” electrophiles, after the names of their discoverers. Finn and coworkers very recently developed another application of this methodology to access selenium-containing bicyclo[3.3.1]nonanes and demonstrated that the reaction of 349 with selenium dichloride/dibromide proceeds at a much faster rate to form selenabicycle 352 (Scheme 80).^163^ Such heavy atom-containing bicyclo[3.3.1]nonanes are found in organosilicon chalcogenides. Herzog and Borrmann showed that organochlorosilanes (354), derived from 2-phenylheptamethyltrisilane (353) on reacting with H_2_S in the presence of triethylamine led to formation of a bicyclo[3.3.1]nonane skeleton with two trisilane units (355) as the major product along with a bicyclo[3.2.2]nonane skeleton as the minor isomer. However, when Li_2_Se was reacted with 354 in THF, the corresponding bicyclo[3.3.1]nonane (356) was formed as the sole product (yield 99%). Both these chalcogenides (355, 356) are found to prefer the twin-boat conformer (Scheme 81).^164^

**Scheme 82:**
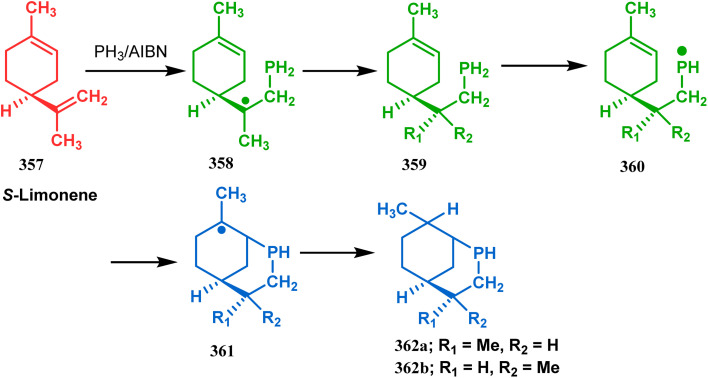
Synthesis of phosphorus analogues of sulphur/selenium-containing bicyclo[3.3.1]nonanes on treatment with PH_3_ and AIBN.

Apart from these sulphur/selenium-containing bicyclo[3.3.1]nonanes, the synthesis of their phosphorus analogues are also well-documented. A unique application of phosphorous-cyclization in this regard was demonstrated by Capretta and coworkers. Their journey began from enantiomerically pure limonene (357), which on treatment with PH_3_ and AIBN transforms into radical 358 and gets trapped as a mixture of two isomeric phosphines (359) through H-abstraction from PH_3_. Phosphinyl radical 360 was then generated from 359 and underwent subsequent intramolecular cyclization to give the desired phosphine ligands 362 in >85% yield (Scheme 82).^165^ These ligands were successfully employed in the cobalt-catalyzed hydroformylation of alkenes.^166^

#### 3.2.4. Tandem *C*-alkylation and heteroatom-cyclization

**Scheme 83:**
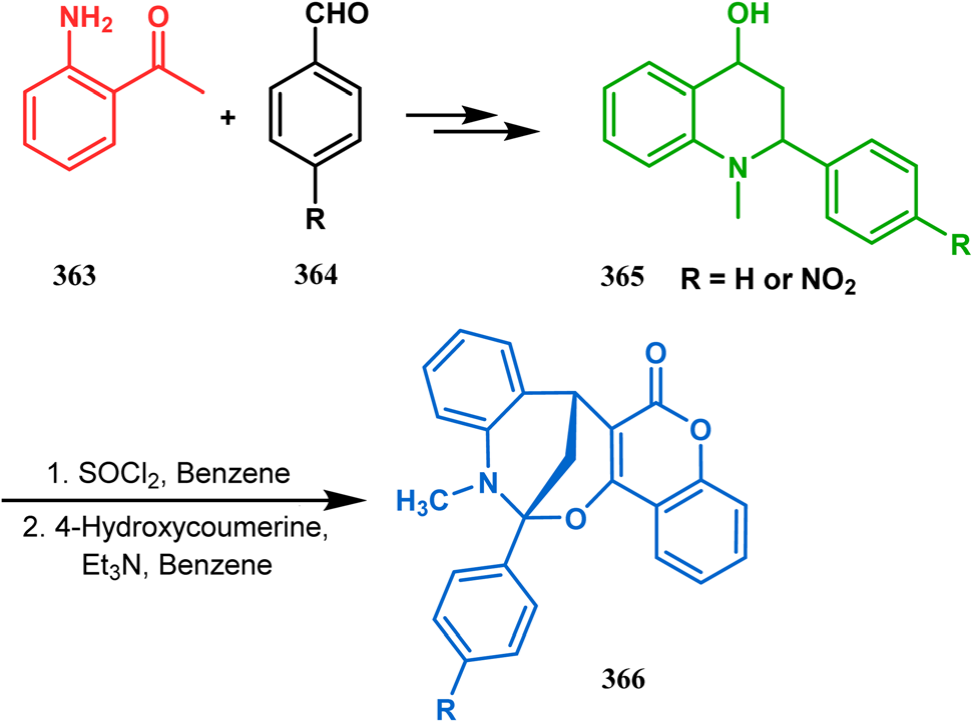
Synthesis of oxazabicyclo[3.3.1]nonane through *C*-alkylation and concomitant *O*-cyclization of 4-hydroxycoumarin.

Tandem *C*-alkylation & heteroatom-cyclization also constitute a well-explored technique for the synthesis of such heteroanalogues of bicyclo[3.3.1]nonane. The initial advancement of this strategy was made by Yang's team in the year 2006. Starting from 2′-amino acetophenone (363) and benzaldehyde derivatives (364), they synthesized the cyclization precursor 365 (yield 90–92%) effortlessly. *C*-alkylation and the concomitant *O*-cyclization of 4-hydroxycoumarin then easily led to the oxazabicyclo[3.3.1]nonane (366) in 43–45% yield (Scheme 83).^167^

**Scheme 84:**
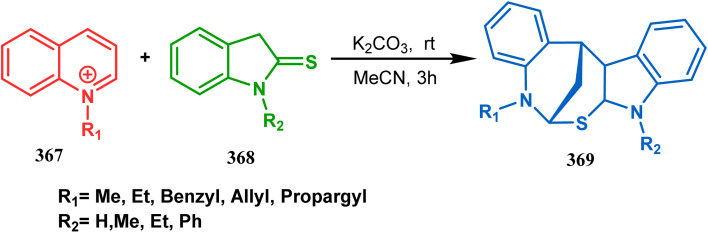
Synthesis of thiaazabicyclo[3.3.1]nonane by the reaction of indolin-2-thione and *N*-alkylquinoliniums through tandem *C*-alkylation and intramolecular *S*-alkylation.

The exploration of this strategy is, however, most extensively done by F. M. Moghaddam's group. Their initial report was a potassium carbonate-mediated single step route toward a thiaazabicyclo[3.3.1]nonane (369). Thus, when indolin-2-thione (367) was treated with *N*-alkylquinoliniums (368), tandem *C*-alkylation and intramolecular *S*-alkylation yielded the bicycle 369 in high yields of 89% (Scheme 84).^168^

**Scheme 85:**
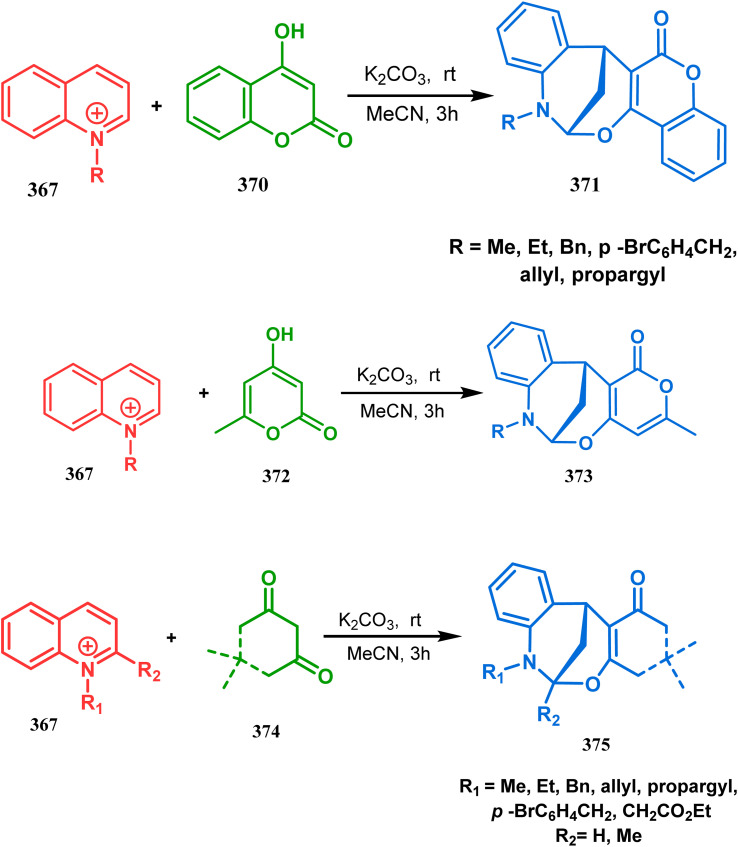
(a) Synthesis of oxazabicyclo[3.3.1]nonane by the reaction of 4-hydroxycoumarin and *N*-alkylquinoliniums through tandem *C*-alkylation and intramolecular *O*-cyclization (b): synthesis of oxazabicyclo[3.3.1]nonane by the reaction of 4-hydroxy-pyronederivative and *N*-alkylquinoliniums through tandem *C*-alkylation and intramolecular *O*-cyclization (c) synthesis of methylene-bridged benzoxazocines by the reaction of active methylene-containing 1,3-diketones and *N*-alkylquinoliniums through tandem *C*-alkylation and intramolecular *O*-cyclization.

Within a few months after this communication, the same group disclosed another report where the same *N*-alkylquinoliniums (367) were exposed to a different binucleophile, 4-hydroxycoumarin (370) this time. This led to a tandem *C*-alkylation and intramolecular *O*-cyclization and produced the oxazabicyclo[3.3.1]nonane 371 in excellent yields (72–89%) (Scheme 85a). The strategy also worked well when the coumarin derivative was replaced by a 4-hydroxypyrone derivative (372), giving high yields of bicycle 373 (71–88%) (Scheme 85b).^169^ This communication was immediately followed by another letter, where a series of novel benzoxazocine derivatives were achieved by applying the same *C*-alkylation and concomitant *O*-cyclization strategy. Active methylene containing 1,3-diketones (374) were utilized this time to get condensed with *N*-alkylquinoliniums (367), resulting in the formation of methylene-bridged benzoxazocines (375) (yield 61–83%) in an identical manner (Scheme 85c).^170^

**Scheme 86:**
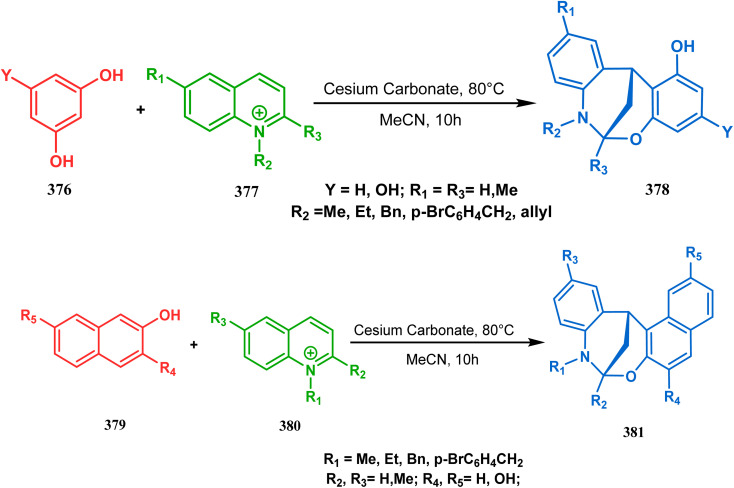
(a)Synthesis of dibenzoxazocines by the reaction of 1,3-dihydroxy aromatics with *N*-alkylquinoliniums through tandem *C*-alkylation and intramolecular *O*-cyclization (b) synthesis of methylene-bridged naphthoxazocines by the reaction of 2-hydroxy-naphthalenes with *N*-alkylquinoliniums through tandem *C*-alkylation and intramolecular *O*-cyclization.

It has also been demonstrated by the same group that 1 3-dihydroxy aromatics (376) could also be successfully annulated with *N*-alkylquinoliniums (377) using the same methodology to achieve a series of dibenzoxazocines (378) in 15–73% yield, depending on the reaction conditions (Scheme 86a). However, a higher reaction temperature is required to realize these annulations, probably due to the unfavorable dearomatization in the *C*-alkylation step.^171^ They has also revealed that even a much less reactive binucleophile such as 2-hydroxynaphthalenes (379) could also be annulated in a similar way to achieve the corresponding methylene-bridged naphthoxazocines (381) in 12–80% yield (Scheme 86b).^172^

**Scheme 87:**
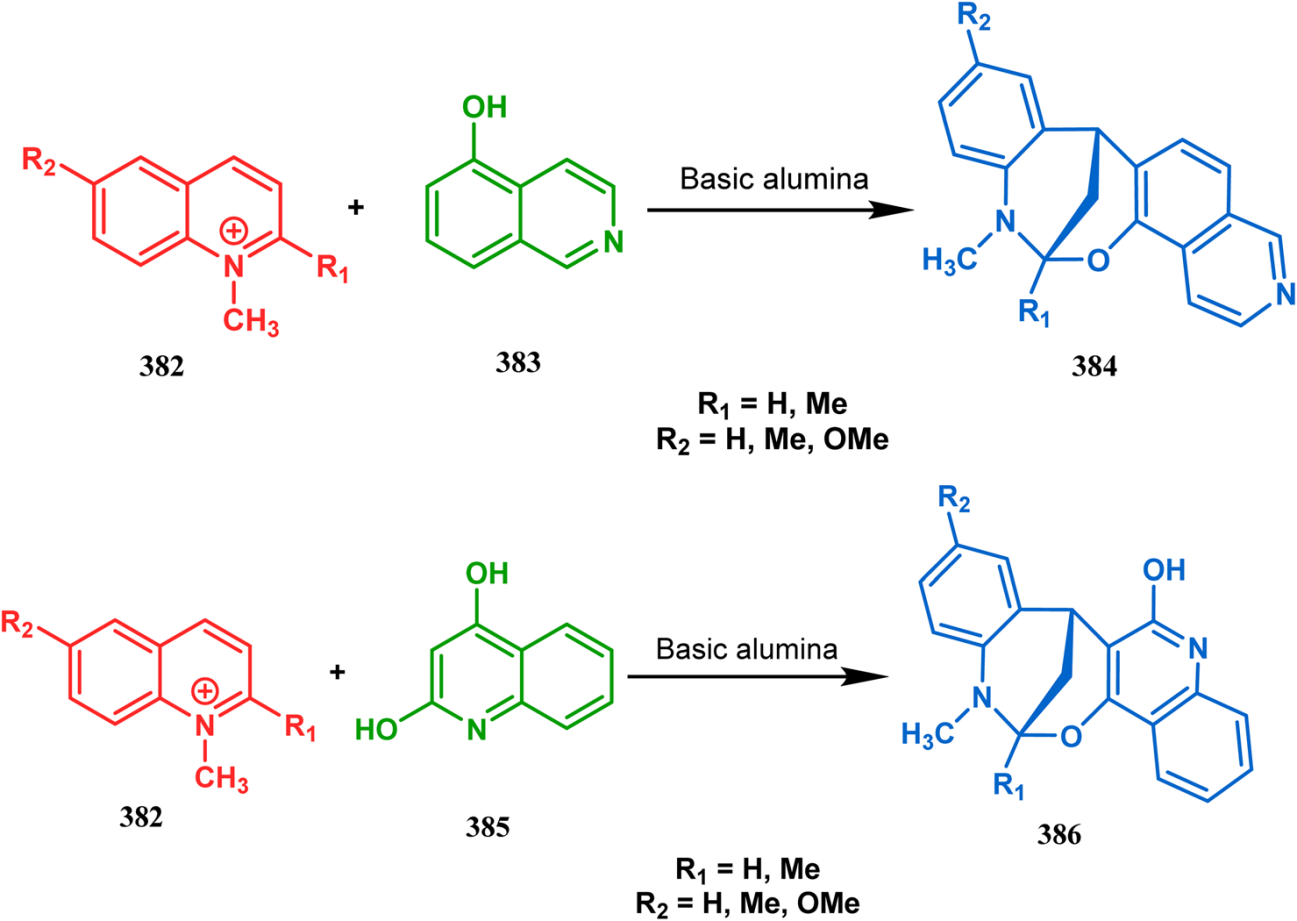
(a) Synthesis of oxazabicylo[3.3.1]nonanes by the reaction of hydroxyisoquinoline and *N*-alkylquinoliniums under microwave irradiation in the presence of basic alumina (b) synthesis of oxazabicylo[3.3.1]nonanes by the reaction of hydroxyquinoline and *N*-alkylquinoliniums under microwave irradiation in the presence of basic alumina.

Apart from the wide ranging investigations of Moghaddam's team, our group also made some contributions toward the synthesis of oxazabicyclo[3.3.1]nonanes. Our initial efforts were dedicated to the synthesis of quinolino/isoquinolino-oxazocines, and through the development of a solvent-free solid-supported methodology, we achieved the target very recently.^173^ Thus, when hydroxyquinolines (383, 385) and *N*-alkylquinoliniums (382) were exposed under microwave irradiation using basic alumina as the solid support, the oxazabicylo[3.3.1]nonanes (384, 386) were obtained in excellent yields (85–89% for 384 and 50–92% for 386) (Scheme 87a and b).

**Scheme 88:**
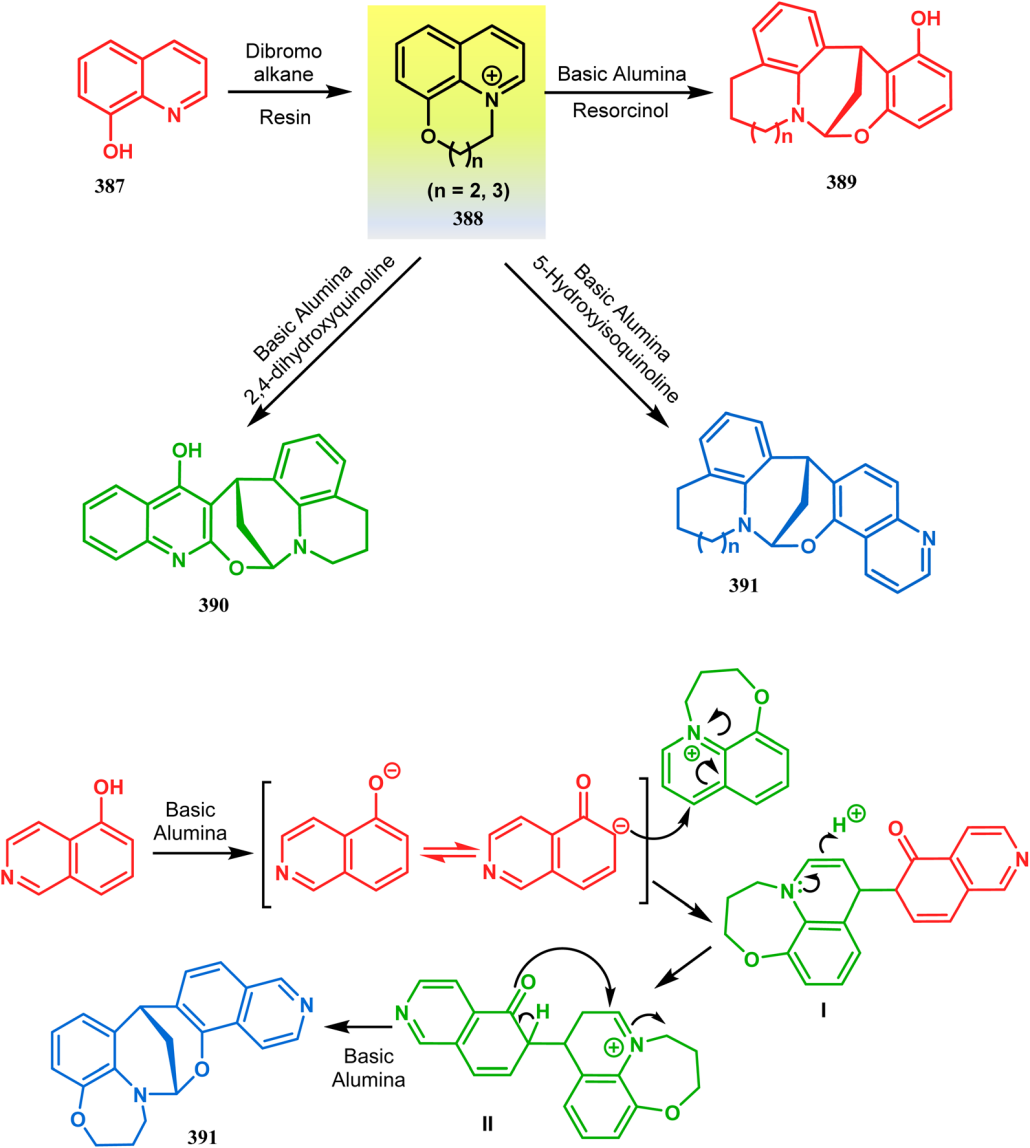
Synthesis of oxazabicyclo[3.3.1]nonane-containing tricycles through initial *C*-alkylation and then intramolecular *O*-alkylation.

In a following letter, we also used this environmentally-benign reaction protocol to synthesize diversely-fused dioxa-2-aza-tricyclo[*n*.3.1.0^2,*n*^]tetra/pentadecanes. The fused tricyclic oxazaquinolinium salts (388), synthesized by our group very recently in high yields from 8-hydroxyquinoline derivatives (387), was treated with binucleophilic phenol/quinoline/isoquinoline derivatives under the described solvent-free protocol and a series of oxazabicyclo[3.3.1]nonane-containing tricycles (389–391) (yield for 389 73–91%, for 390 87–89%, and for 391 77–83%) were accessed effortlessly (Scheme 88).^174^ It is believed that the reaction proceeds through an initial *C*-alkylation on the quinolinium salt to produce an intermediate, followed by an intramolecular *O*-alkylation, thereby producing 391. It is important to note that all these reactions proceeded with initial *C*-alkylation, followed by heteroatom-cyclization and not the other way around, thereby demonstrating a particular regioselective nature of this reaction.

**Scheme 89:**
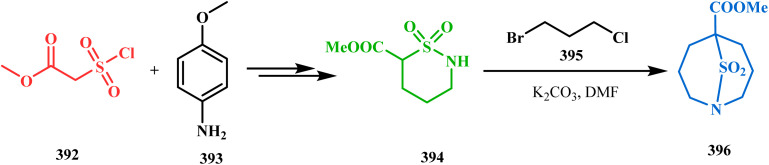
Synthesis of 1-aza-9-thiabicyclo[3.3.1]nonane derivative by the reaction of methyl-2-(chlorosulfonyl) acetate and *p*-anisidine through tandem *C*-alkylation and *N*-alkylation.

Another application of tandem *C*-alkylation and *N*-alkylation was unveiled by Sokolov and Meijere's group. Their route toward the cyclization precursor 394 started from methyl-2-(chlorosulfonyl) acetate (392) and *p*-anisidine (393).^175,176^ The cyclic sultam (394) thus synthesized was then treated with 1-bromo-3-chloropropane (395) under basic conditions to achieve the desired 1-aza-9-thiabicyclo[3.3.1]nonane derivative (396) in 52–68% yield (Scheme 89). The conformation of this bicyclic sultam was confirmed from X-ray crystallographic analysis, which showed that 396 prefers to stay in its chair–chair conformation, as expected from the fact that an alternate chair-boat conformation should be destabilized by the van der Waals repulsion between the SO_2_ and the C3 and C7 methylene groups.^177^

### 3.3. Ring opening of other polycycles

#### 3.3.1. Cyclopropane-type ring opening

Studies on the ring-opening reactions of cyclopropane-type systems have been well explored in recent years. In particular, the ring opening of these systems to produce bicyclo[3.3.1]nonane analogues constitutes a useful way toward the synthesis of this important core moiety. During the end of the 20th century, one such important report appeared in the literature due to Srikrishna and coworkers.

**Scheme 90:**
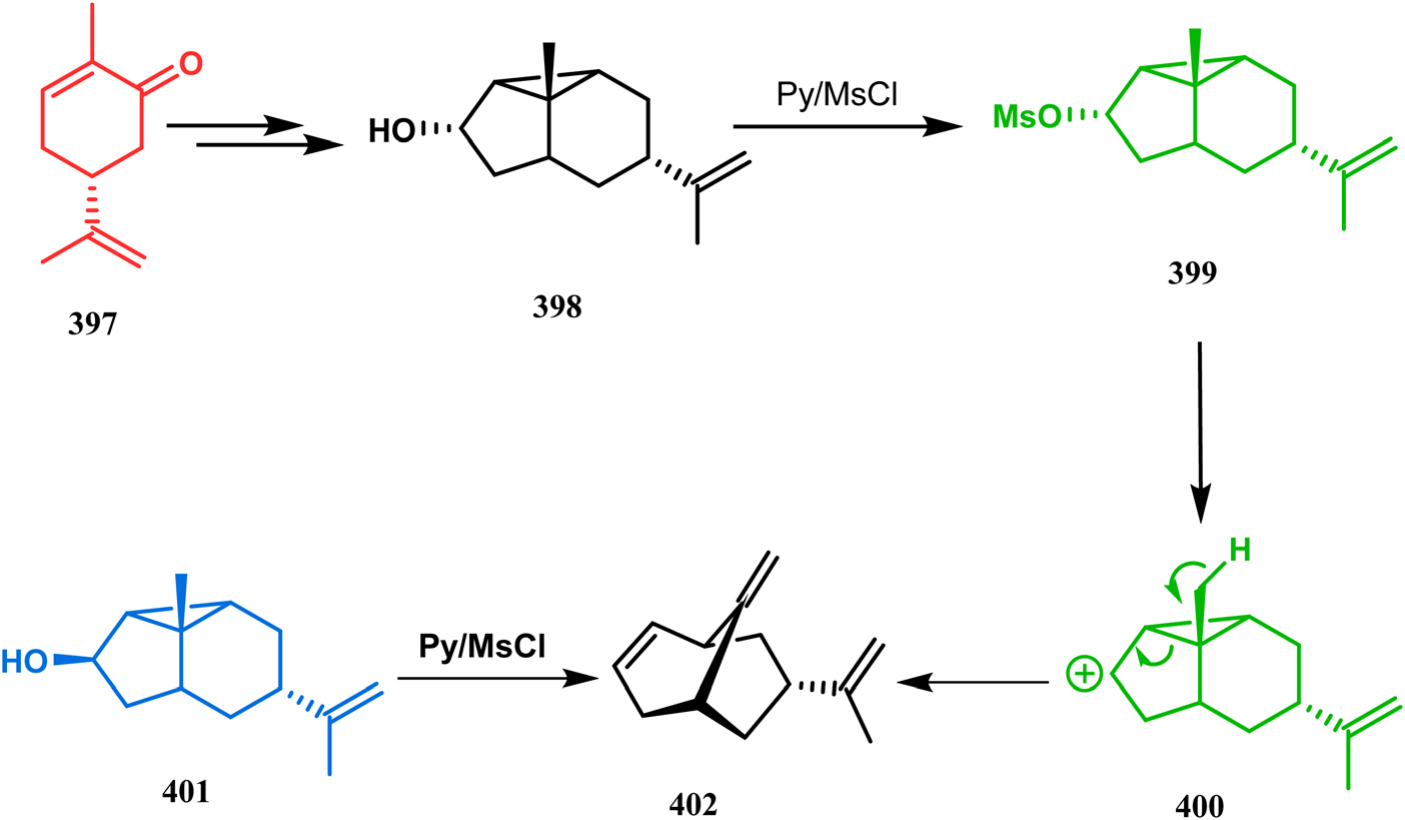
Synthesis of chiral bicyclo[3.3.1]nonanes through cyclopropane-type regioselective ring opening of l-methyltricyclo[4.3.0.0]nonan-8-ols.

Their studies on the regioselective cyclopropane cleavage of *l*-methyltricyclo[4.3.0.0^2,9^]nonan-8-ols unveiled an important route toward chiral bicyclo[3.3.1]nonanes. The ring opening precursor is a tricyclic *endo*-alcohol (398) (yield 87%) in this case, which was derived from *R*-carvone (397) in a stereocontrolled manner. It is believed that the mesylate (399) formed from 398 undergoes an E1-type homo-1,4-elimination reaction to give 402 (yield 76%) through a carbocation intermediate 400. Although a Grob-type fragmentation (concerted E2-type elimination) could also explain the outcome, this possibility was ruled out by the fact that the corresponding *exo*-alcohol (401) (yield 63%) also leads to the same product, thus supporting the E1-type mechanism (Scheme 90).^178^

**Scheme 91:**
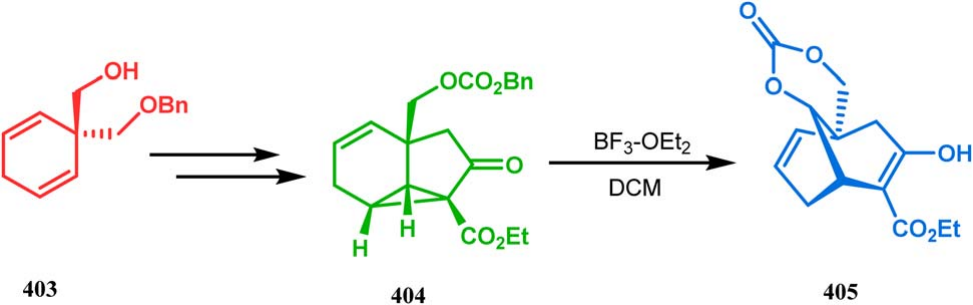
Construction of the bicyclo[3.3.1]nonane moiety through the regioselective ring-opening reaction of the tricycle [4.4.0.0]dec-2-ene.

A closely related approach from a similar type of ring opening precursor is also developed by Nakada and coworkers.^179,180^ They demonstrated a Lewis acid-promoted regioselective ring-opening reaction of the tricycle [4.4.0.0^5,7^]dec-2-ene derivative to achieve a bicyclo[3.3.1]nonane moiety, which has the potential to be used as an intermediate for the synthesis of phloroglucines. Starting from alcohol 403, the required precursor 404 was thus synthesized in a stepwise manner in 93% yield, which upon BF_3_–OEt_2_-promoted ring opening reaction in DCM yielded the desired bicycle 405 in excellent yields (33–93%) (Scheme 91). The reaction is believed to proceed *via* an intramolecular benzyl carbonate attack, leading to cyclopropane ring opening.^180^

**Scheme 92:**
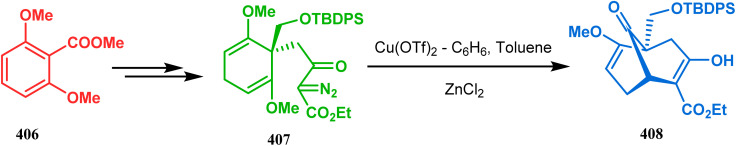
Synthesis of the bicycle core through Lewis acid-mediated cyclopropane ring-opening reaction.

In a following letter, within a year, the same group disclosed another application of Lewis acid-mediated cyclopropane ring-opening reaction to achieve a similar goal. This time, the cyclopropanation and ring opening reaction to yield the desired bicycle (408) were done in one-pot sequence. The idea was to facilitate the formation of a more stabilized carbocationic C9 center (by the adjacent methoxy group) to be generated from the methoxy group-triggered ring-opening reaction. Thus, the chronological transformation of 404 into 407 (yield 64%), followed by Cu(OTf)-mediated cyclopropanation and concommitant ZnCl_2_-promoted ring opening reaction condition, yielded the desired bicycle 408 in 47% yield (Scheme 92).^179^

**Scheme 93:**
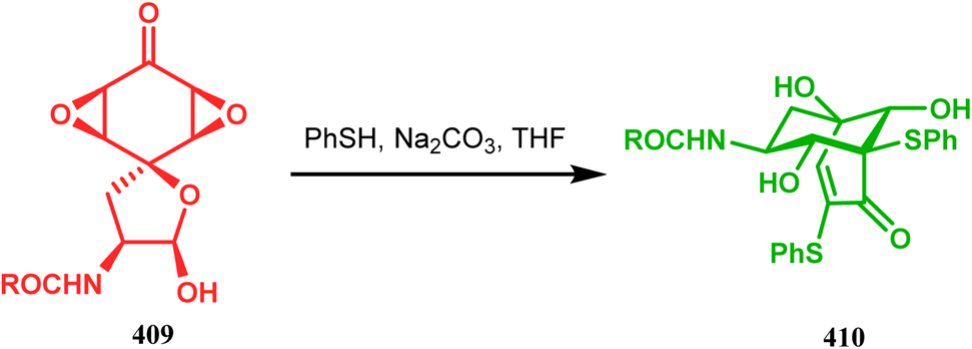
Synthesis of the bicyclo[3.3.1] non3-en-2-one system from spirodiepoxyketone through the three-membered ring-opening and rearangement reaction.

Wipf and coworkers described a unique example of such a 3-membered ring-opening reaction during aranorosin synthesis. They discovered the fascinating transformation of a spirodiepoxyketone (409) into a bicyclo[3.3.1]non3-en-2-one system (410) in 71% yield, when treated with thiophenols under basic conditions (Scheme 93).^181^

**Scheme 94:**
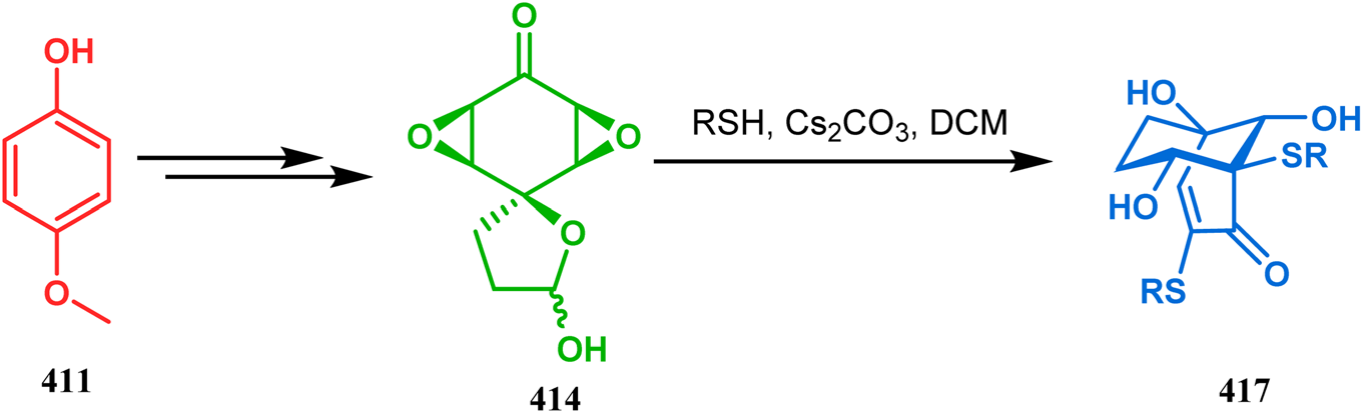
Synthesis of bicyclo[3.3.1]non3-en-2-one system from spirodiepoxyketone obtained from 411 through three-membered ring-opening and rearrangement reaction.

**Scheme 95:**
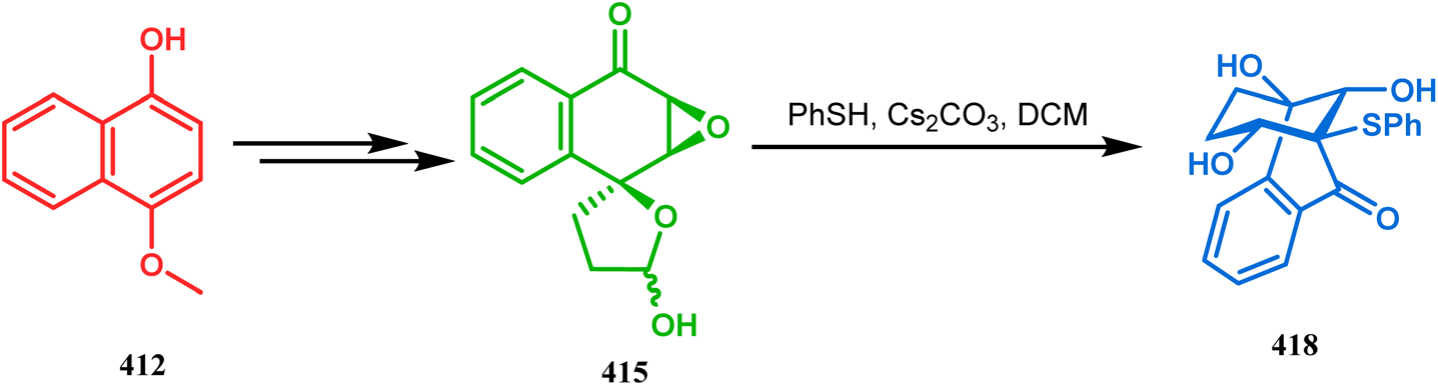
Synthesis of bicyclo[3.3.1]non3-en-2-one system from spiromonoepoxyketone obtained from 412 through three-membered ring-opening and rearrangement reaction.

**Scheme 96:**
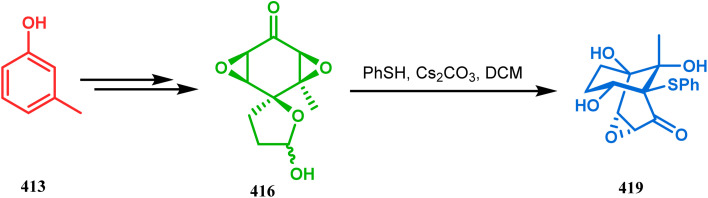
Synthesis of the bicyclo[3.3.1]non3-en-2-one system from spiromonoepoxyketone obtained from 413 through three-membered ring-opening and rearrangement reaction.

Stunning structural similarity between 410 and the bicyclic core of natural products gymnastatins F and Q prompted the authors to explore this methodology further. Very recently, the synthesis of densely-functionalized derivatives of this bicyclic system was reported by the same group. For example, spirodiepoxyketones and monoepoxyketones (414–416) obtained in 95% yield from 411–413, respectively, satisfactorily underwent the described ring-opening/rearrangement cascade, yielding the bicyclo[3.3.1]non3-en-2-ones (417–419) in good yields (53–84%) (Schemes 94–96).^182^ It was also found that although neither electron-donating nor electron-withdrawing substituents in aromatic thiols could affect any parameter of this reaction, the methyl substitution in epoxyketones decelerates product formation.

**Scheme 97:**
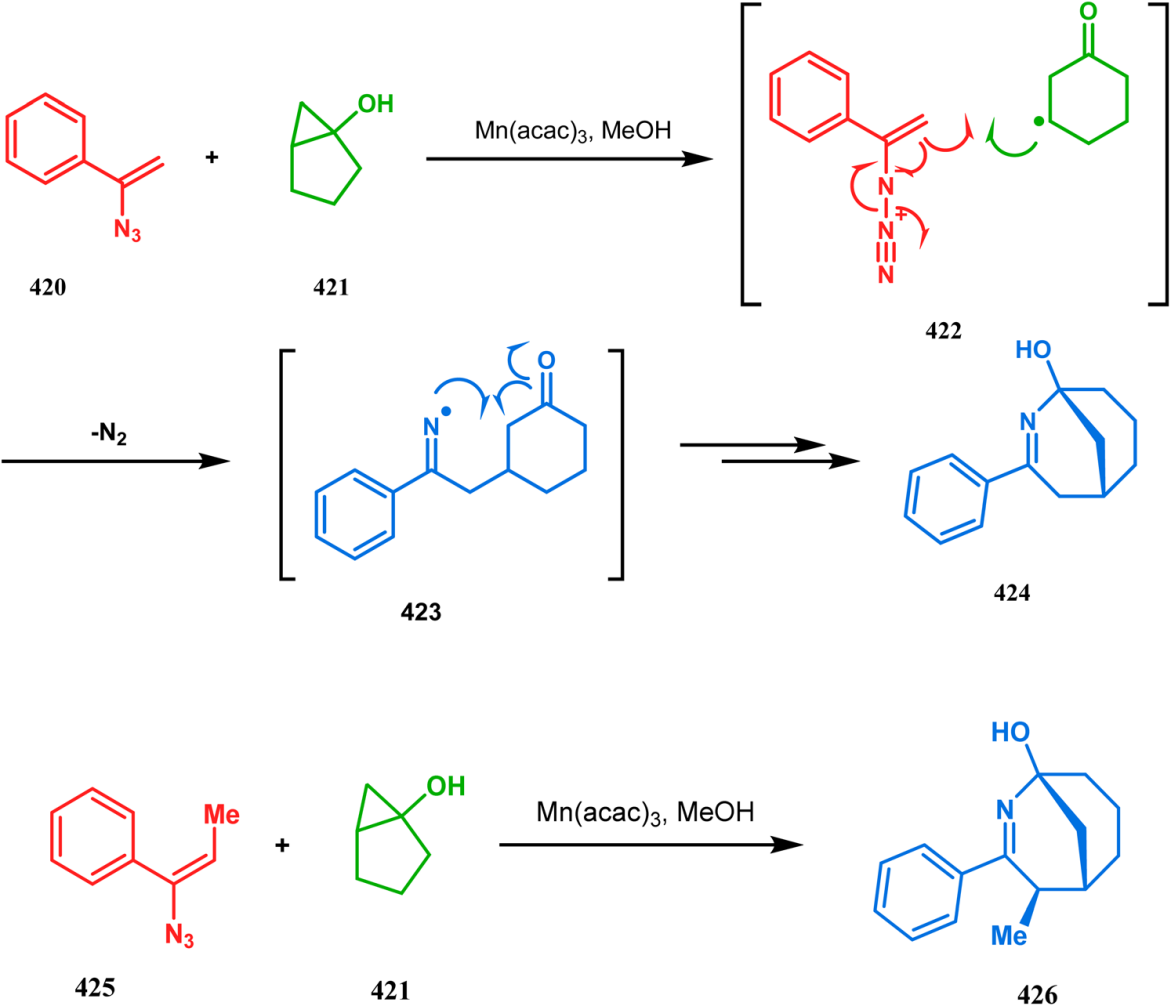
(a) Synthesis of 2-azabicyclo[3.3.1]non2-en-1-ol derivative through Mn(acac)_3_-catalyzed reaction between bicyclic cyclopropanol with vinyl azide (420), followed by intramolecular radical cyclization (b) synthesis of 2-azabicyclo[3.3.1]non2-en-1-ol derivative through Mn(acac)_3_-catalyzed reaction between bicyclic cyclopropanol with vinyl azide derivative (418), followed by intramolecular radical cyclization.

The ring opening of bicycles is also a widely utilized tool to produce azabicyclo[3.3.1]nonanes. A classic example in this catagory should be azabicyclo[3.3.1]nonane analogue synthesis through the ring opening of bicyclic cyclopropanol derivatives. One such report from Chiba and Wang appeared very recently. They designed a useful route toward the 2-azabicyclo[3.3.1]non2-en-1-ol derivatives (429, 433) through Mn(acac)_3_-catalyzed reaction between bicyclic cyclopropanols (428) with vinyl azides (427, 432). The loss of chirality during the reaction proves that a radical pathway was followed during the course of the reaction. It is believed that the Mn(iii) complex promotes the cyclopropane ring opening and forms the reactive radical intermediate 422, which reacts with the vinyl azides and transforms into 424 through the intramolecular radical cyclization of 423 (Scheme 97a).^183^ A β-methyl group in the vinyl azide retards the reaction, corroborated by the slow reaction rate between 421 and the hindered vinyl azide 425 to give bicycle 426 in 87–89% yield (Scheme 97b).

#### 3.3.2. Adamantane-type ring-opening

**Scheme 98:**
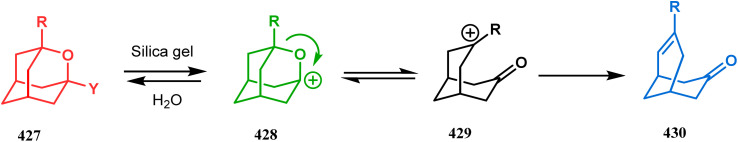
Synthesis of 7-alkylbicyclo[3.3.1]non6-en-3-one from 3-alkyl-2-oxaadamant-l-yl-mesylate through the ring-opening reaction.

The ring opening of adamantane-type systems is also a well-explored technique to achieve bicyclo[3.3.1]nonane units. One such example is the synthesis of bicyclo[3.3.1]non6-en-3-ones. Inspite of several available protocols toward this system,^184,185^ the complexity and lack of generality of those routes prompted Camps and coworkers to unmask a more convenient route to synthesize this important core moiety, leading to the discovery of a unique application of silica gel to achieve 7-alkylbicyclo[3.3.1]non6-en-3-ones. Thus, when 3-alkyl-2-oxaadamant-l-yl-mesylates (427) were treated with silica gel in DCM, the starting material was fragmented and bicycles 430 were obtained through cabocation intermediates 428 and 429 in high yields (71%) (Scheme 98).^186^

**Scheme 99:**
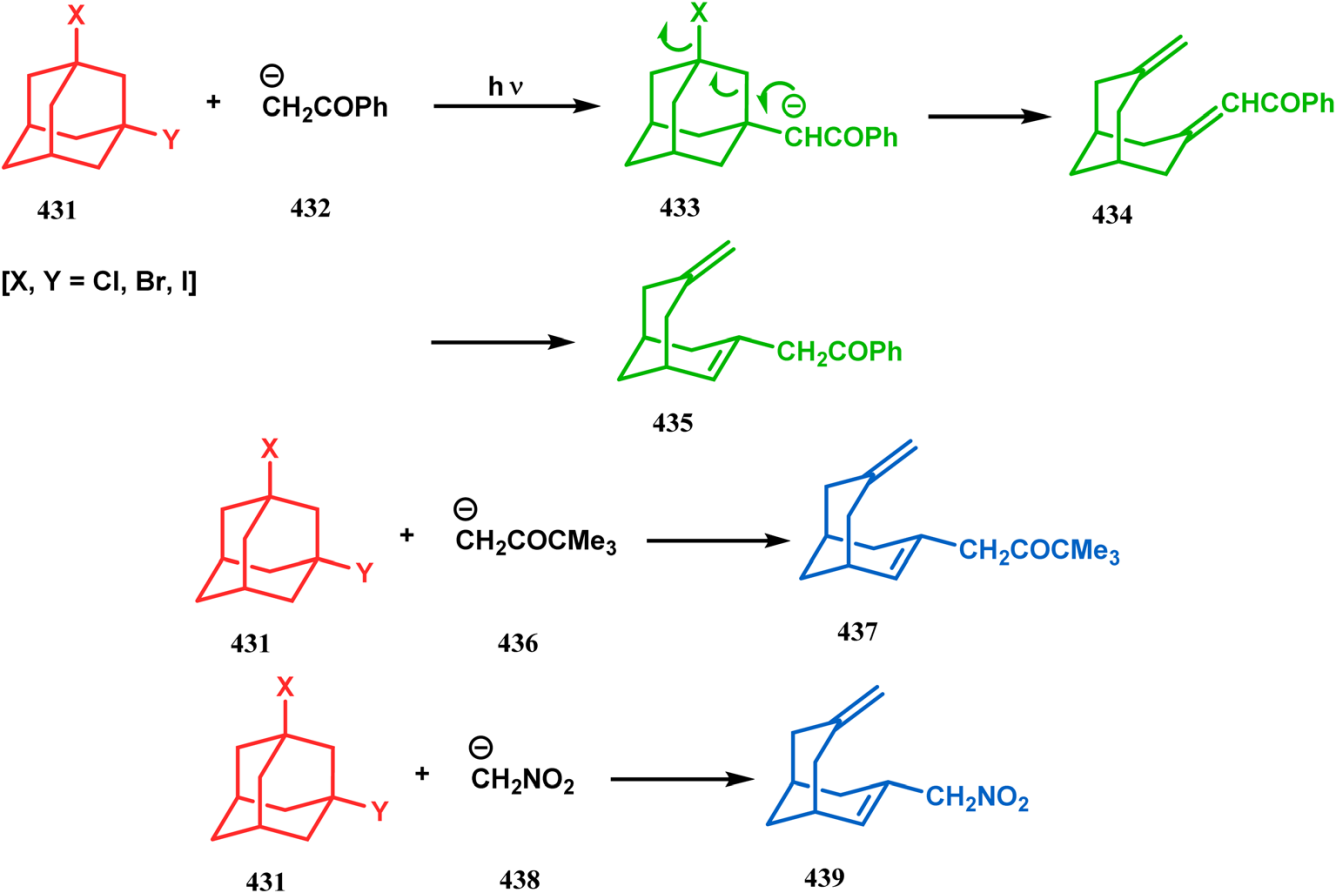
Synthesis of 7-methylidenebicyclo[3.3.1]nonanes through the fragmentation reaction of adamantane ring systems.

**Scheme 100:**
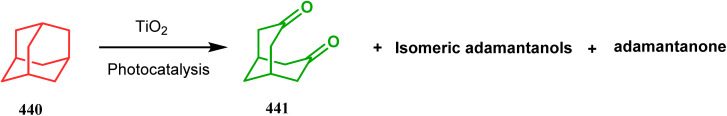
Formation of bicyclo[3.3.1]nonanedione through TiO_2_-mediated photocatalysis of unsubstituted adamantine.

Rossi's group in 1997 demonstrated another fragmentation reaction of adamantane ring systems, yielding 7-methylidenebicyclo[3.3.1]nonane. Although closely related fragmentation reactions of 1-hydroxy-/1-thio-3-bromoadamantanes always require a basic condition,^187^ Rossi's approach was photochemically based, which promotes a very fast ring-opening reaction of 1,3-dihaloadamantanes (431) with acetophenone enolates (432), forming α-(7-methylidenebicyclo[3.3.1]non2-en-1-yl)acetophenone (435) with high yields. The reaction proceeds with an initial substitution reaction on 431 by 432 to form the monohalo adamantane 433, followed by the ring-opening reaction to produce 434 (Scheme 99) which immediately isomerized to 435. Similar reactions with pinacolone enolate (436) to produce 437 (yield 26–81%) are also fast, whereas the nitromethane anion (438) reacted with 431 in a rather decelerated rate, forming bicycle 439 in 11–67% yield (Scheme 99).^188^ The irradiation of unsubstituted adamantane, however, shows a different chemistry. Thus, when Albini and coworkers attempted TiO_2_-mediated photocatalysis of 440, a minor amount of bicyclo[3.3.1]nonanedione (441) was isolated along with two isomeric adamantanols and 2-adamantanone (Scheme 100).^189^

**Scheme 101:**
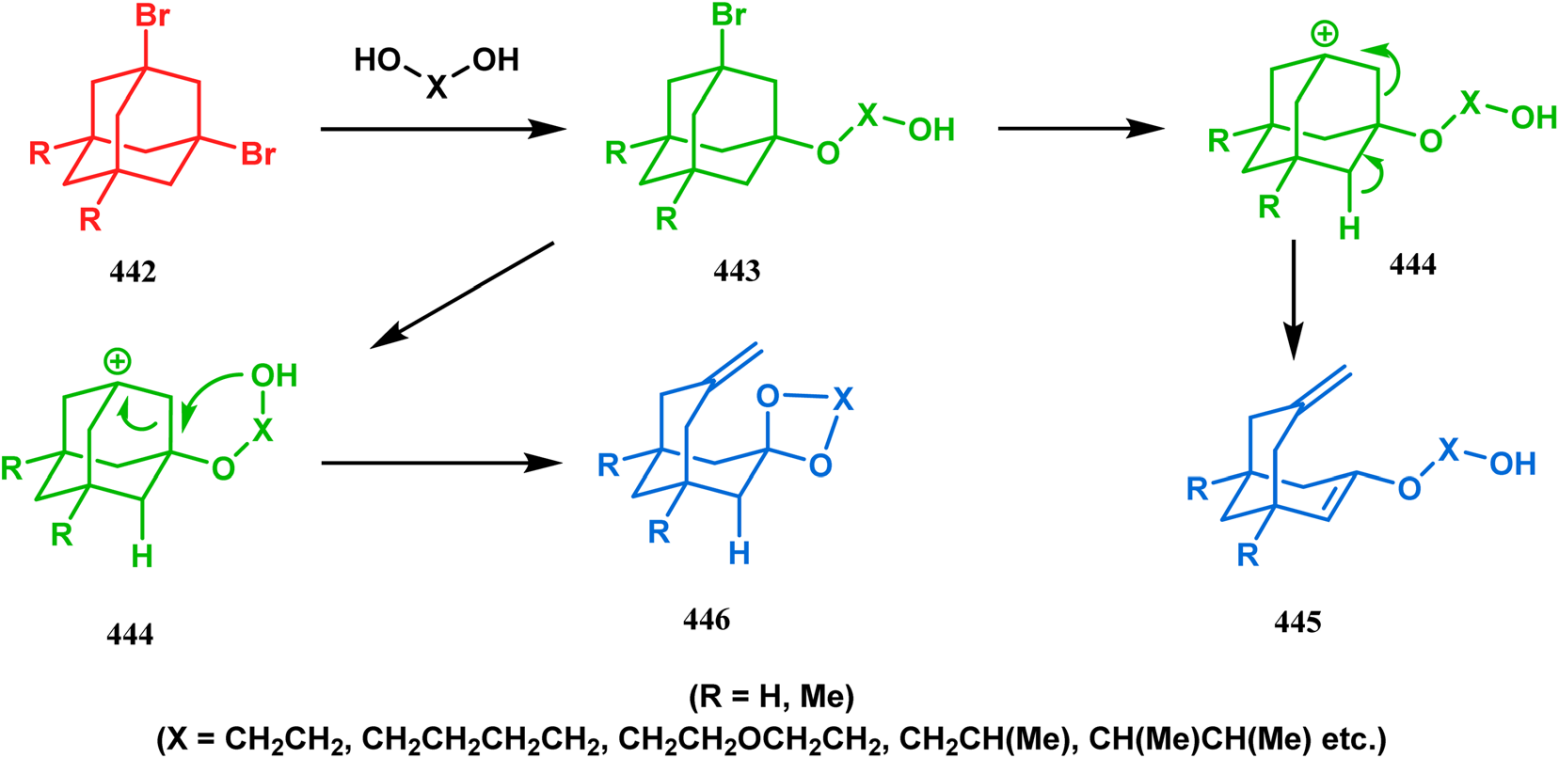
Synthesis of bicyclo[3.3.1]nonane derivatives by the reaction of 1,3-dibromoadamantane with glycols in the presence of sodium glycolate.

The fragmentation reaction of 1,3-disubstituted-adamantanes to produce bicyclo[3.3.1]nonane derivatives has been well investigated in the last 50 years.^190–196^ Toward this goal, very recently, Skomorokhov and coworkers described the reaction of 1,3-dibromoadamantane (442) with glycols in the presence of sodium glycolate to synthesize bicyclo[3.3.1]nonane derivatives (445, 446) (Scheme 101). It is believed that an intermediate adamantyl carbocation (444), formed by the bromide elimination from 443, yielded the differently substituted bicycles in a stepwise manner.^197,198^

**Scheme 102:**
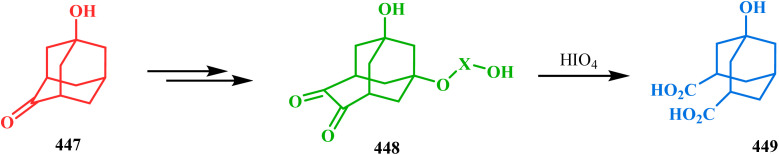
Synthesis of 1,3,7-trisubstituted bicyclo[3.3.1]nonane from 1-hydroxyadamantane-4-one and then the synthesis of bicyclic 1,3-dicarboxylic acid on treatment with HIO_4_.

Another simple and convenient synthesis of 1,3,7-trisubstituted bicyclo[3.3.1]nonanes from adamantane derivatives was described by Majerski's group. Their journey commenced from 1-hydroxyadamantane-4-one (447), which, upon sequential ring expansion and oxidation, yielded the ring opening precursor 448, an adamantane-dione derivative, which on treatment with HIO_4_ yielded the bicyclic 1,3-dicarboxylic acid 449 in 36% yield (Scheme 102).^199^

**Scheme 103:**
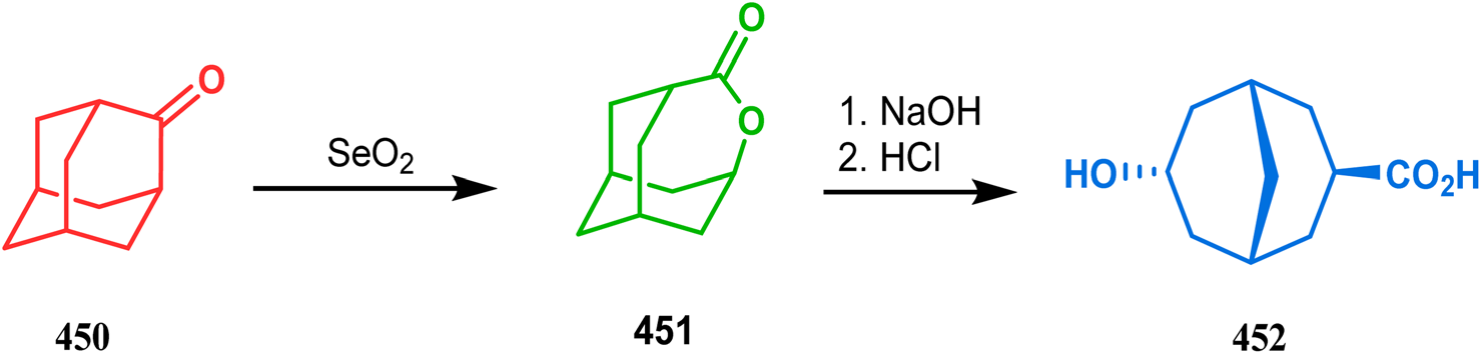
Synthesis of *endo*-hydroxycarboxylic acid from adamantane-2-one.

**Scheme 104:**
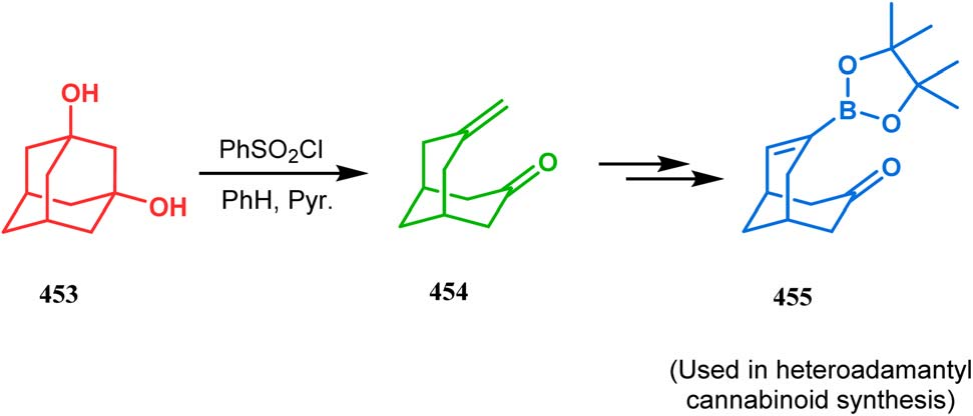
Synthesis of differently-functionalized bicyclo[3.3.1]nonane by the fragmentation of 1,3-adamantanediol.

Nurieva and coworkers also employed such adamantyl precursors to achieve their desired colchicine analogues. Thus, adamantane-2-one (450) was first subjected to selenium dioxide-promoted ring-opening reaction, followed by concomitant lactonization to give a tricyclic lactone 451. The base-catalyzed hydrolysis of this lactone then yielded an *endo*-hydroxy carboxylic acid 452 (Scheme 103).^200^ The fragmentation of 1,3-adamantanediol (453) is also another useful procedure to achieve functionalized bicyclo[3.3.1]nonanes. Few years ago, Iwabuchi and coworkers demonstrated one such useful application. Following their report, 453 could easily be exposed toward Grob fragmentation to give an exocyclic double bond-containing bicyclo[3.3.1]nonane-3-one (454) derivative,^201,202^ which was then used as the precursors for the synthesis of differently functionalized bicyclo[3.3.1]nonanes (455) in 73% yield (Scheme 104).

### 3.4. Other routes

#### 3.4.1. Hosomi–Sakurai & carbonyl-ene reaction

**Scheme 105:**
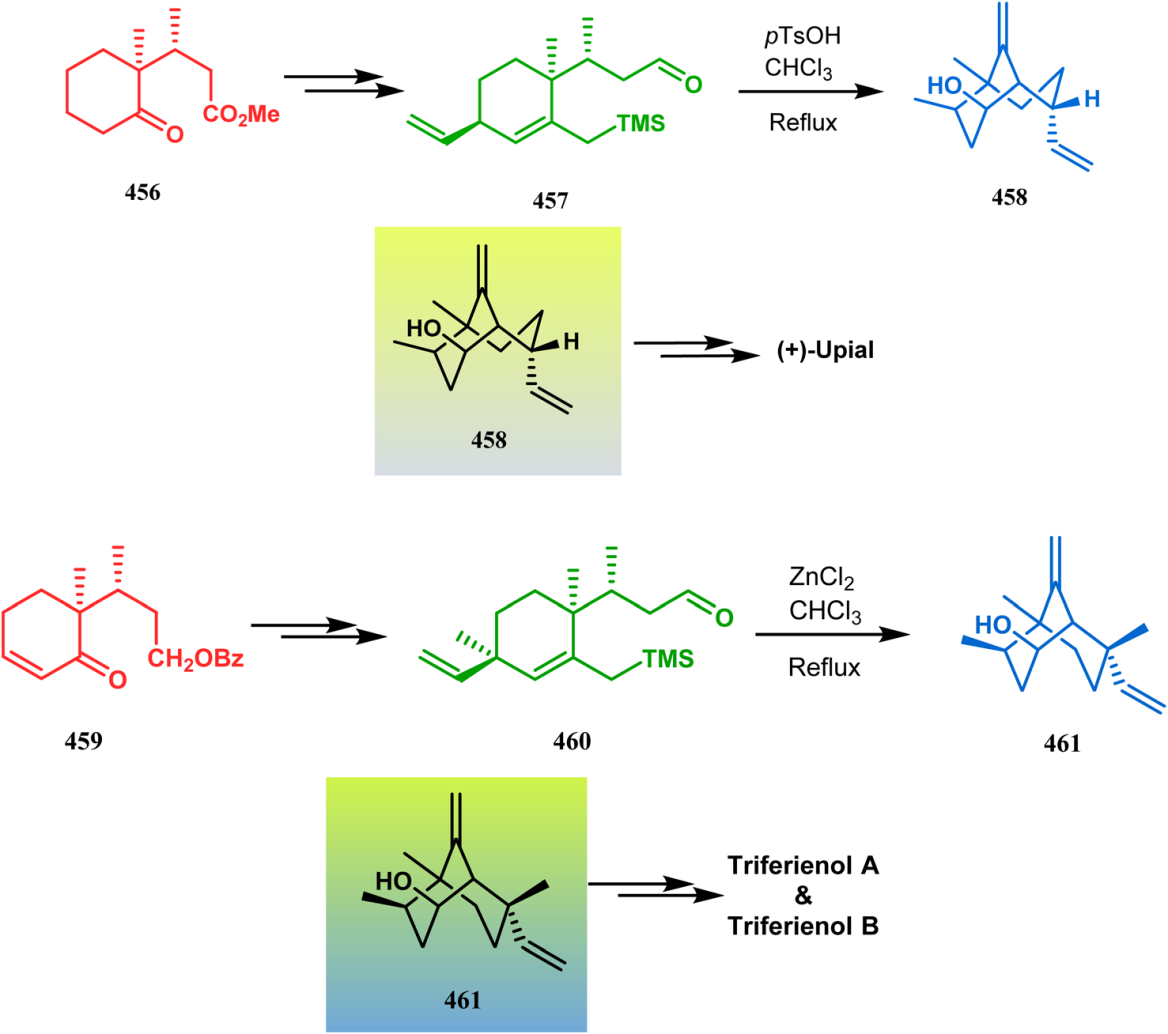
Synthesis of bicyclo[3.3.1]nonane derivatives following Hosomi–Sakurai reaction and the carbonyl–ene reaction from appropriately positioned allyl silane derivatives.

The Hosomi–Sakurai reaction and the carbonyl–ene reaction are the two competitive candidates for preparing bicyclo[3.3.1]nonane derivatives from appropriately positioned allyl silane derivatives. Although both of them share an almost similar reaction condition, they differ in their area of application. Honda and coworkers in their two successive articles exemplified this fact in detail. Their initial report was on the total synthesis of upial, a bicyclo[3.3.1]nonane-containing tricyclic terpenoid. The required aldehyde 457 for this purpose was synthesized in a stereocontrolled manner from the optically active ketone 456 stereoselectively. However, the key step of this transformation is the *p*-toluenesulfonic acid-catalyzed cyclization of aldehyde 457 to give the desired bicycle 458 (yield 74–96%) (Scheme 105). This bicycle was then sequentially converted into upial in a stepwise manner.^203^ However, the *p*TsOH-mediated strategy did not work satisfactorily for the synthesis of diastereomeric sesquiterpenes Triferienols A & B. Although the cyclization precursor 460 obtained from ketone 459 produced the desired bicycle 461 through the intramolecular cyclization in 93% yield, an acetal side product also accompanied it.

It is believed that the extra methyl substituent on the allyl group-containing quaternary carbon center leads to a substantial increase in steric crowding during cyclization, therefore raising the possibility of acetal or half-acetal formation. Therefore, an alternate Lewis acid ZnCl_2_ was introduced, which acted as the initiator of the Hosomi–Sakurai reaction, thereby favoring the cyclization prior to the acetal formation, yielding the desired bicyclic core 461 in excellent yield (93%), which was then transformed into Triferienols A & B in a few steps (Scheme 105).^204^

#### 3.4.2. Mannich reaction

**Scheme 106:**
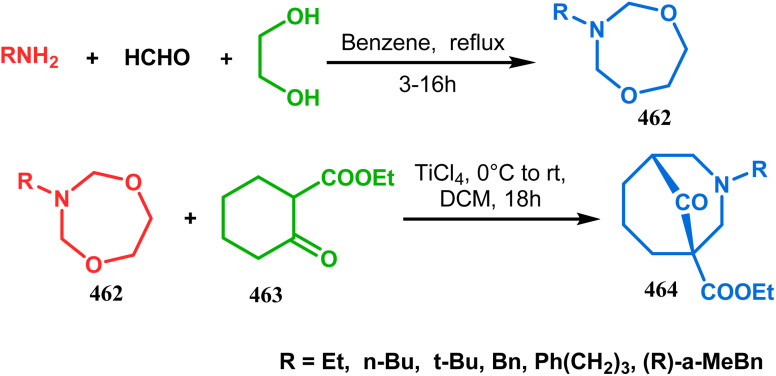
Synthesis of azabicyclo[3.3.1]nonane following Mannich reaction.

Brimble and coworkers demonstrated an elaborated investigation on the application of Mannich reaction to synthesize azabicyclo[3.3.1]nonanes.^202^ Their initial target was to synthesize diversely-substituted dioxazepanes (462) (yield 54–97%) as the Mannich reaction partner of β-keto esters (463). These dioxazepanes, constructed from paraformaldehyde, ethylene glycol, and a primary amine, were then used as the electrophiles for the double Mannich reaction to achieve the desired azabicycles (464). To find the best activator for the desired transformation, a variety of Lewis acids were screened, and methyltrichlorosilane appeared as the best candidate (Scheme 106).^205^

#### 3.4.3. Cycloaddition reaction

**Scheme 107:**
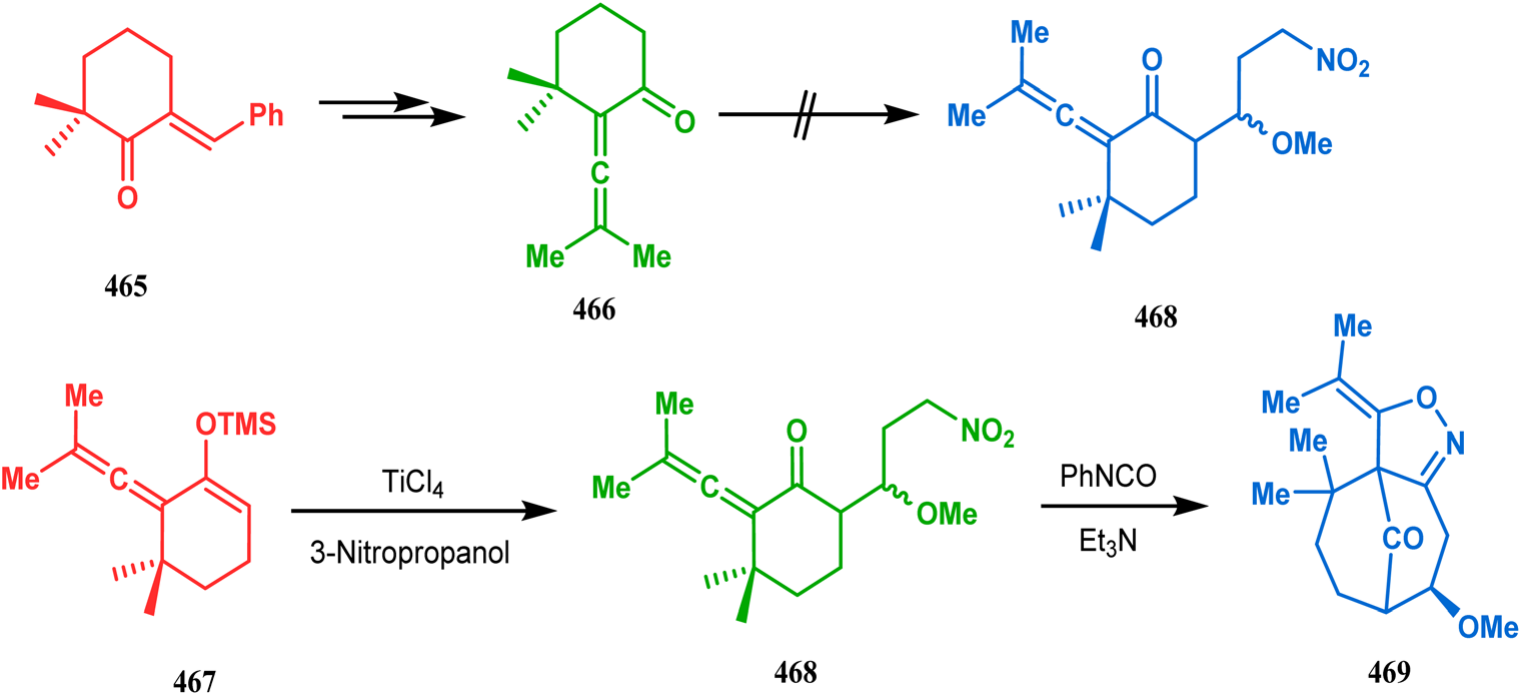
Synthesis of the bicyclo[3.3.1]nonane-containing tricycle through nitrile oxide-allene cycloaddition reaction.

Cycloaddition reactions are widely used in the synthesis of natural products. Bicyclo[3.3.1]nonane-containing natural products are also not an exception. In this regard, a unique application of nitrile oxide-allene cycloaddition^206^ in Hypervolutin A core synthesis was demonstrated by Young and Zeng. Their journey began from the benzylidene ketone 465, which was sequentially transformed into 466 for the synthesis of the cyclization precursor 468. This attempt, however, remained unsuccessful, and an alternate allene derivative 467 was employed in a Lewis acid-promoted Mukaiyama condensation reaction to synthesize 468, a diastereomeric mixture of an allene. This cyclization precursor was then exposed toward phenyl isocyanate to promote the envisioned nitrile oxide-allene cycloaddition reaction, which yielded the desired bicyclo[3.3.1]nonane-containing tricycle 469 in 40% yield (Scheme 107).^207^ Although, both the diastereomers of 468 were subjected to cycloaddition condition, only the ‘*anti*’ cycloadduct (carbonyl group and methoxy group opposite to each other) was produced, indicating the reluctant nature of the ‘*syn*’ diastereoisomer of 468 toward the cycloaddition reaction.

#### 3.4.4. Double condensation reactions

**Scheme 108:**
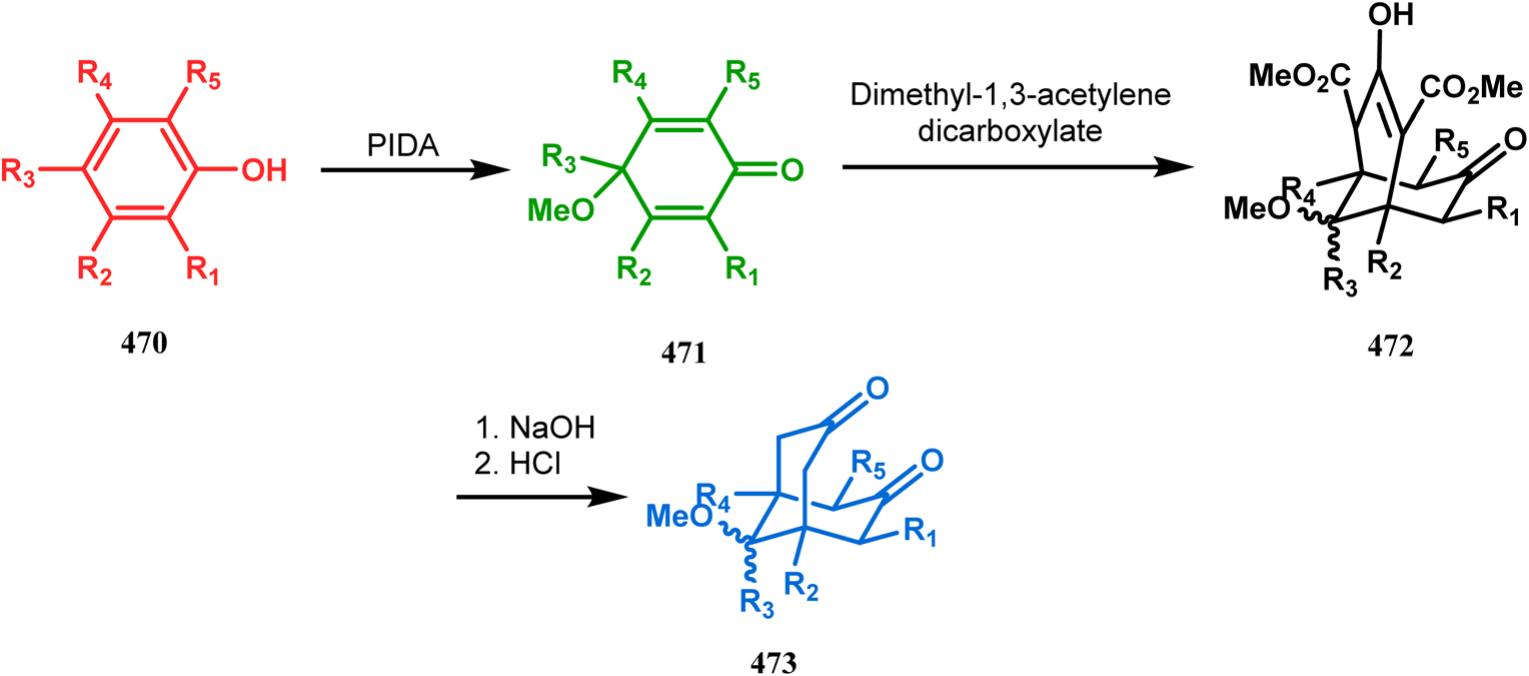
Synthesis of polysubstituted bicyclo[3.3.1]nonane-3,7-dione through phenyliodonium diacetate (PIDA)-mediated phenol oxidation and double Michael condensation reaction.

Multicomponent reactions, especially the 1-step double condensation of two units of 1,3-acetonedicarboxylates with 1,2-dicarbonyl/1,4-dicarbonyl compounds, are a well-explored protocol to achieve bicyclo[3.3.1]nonane-3,7-diones.^208–214^ Following these reports, Camps and coworkers reported the synthesis of 9-methoxy-9-methylbicyclo[3.3.1]nonane-3,7-dione.^215^ A similar report on the synthesis of polysubstituted bicyclo[3.3.1]nonane 3,7-diones from cyclohexa-2,5 dienones by the same group appeared during the beginning of this century. This time, a sequential phenyliodonium diacetate (PIDA)-mediated phenol oxidation and double Michael condensation was employed to achieve the desired bicycles. Thus, starting from phenols 470, polysubstituted bicyclo[3.3.1]nonane-dicarboxylates (472) were obtained through quinones 471. These dicarboxylates upon hydrolysis-decarboxylation produced the targeted bicyclic diones (473) in high yields (15–90%) (Scheme 108).^216^

**Scheme 109:**
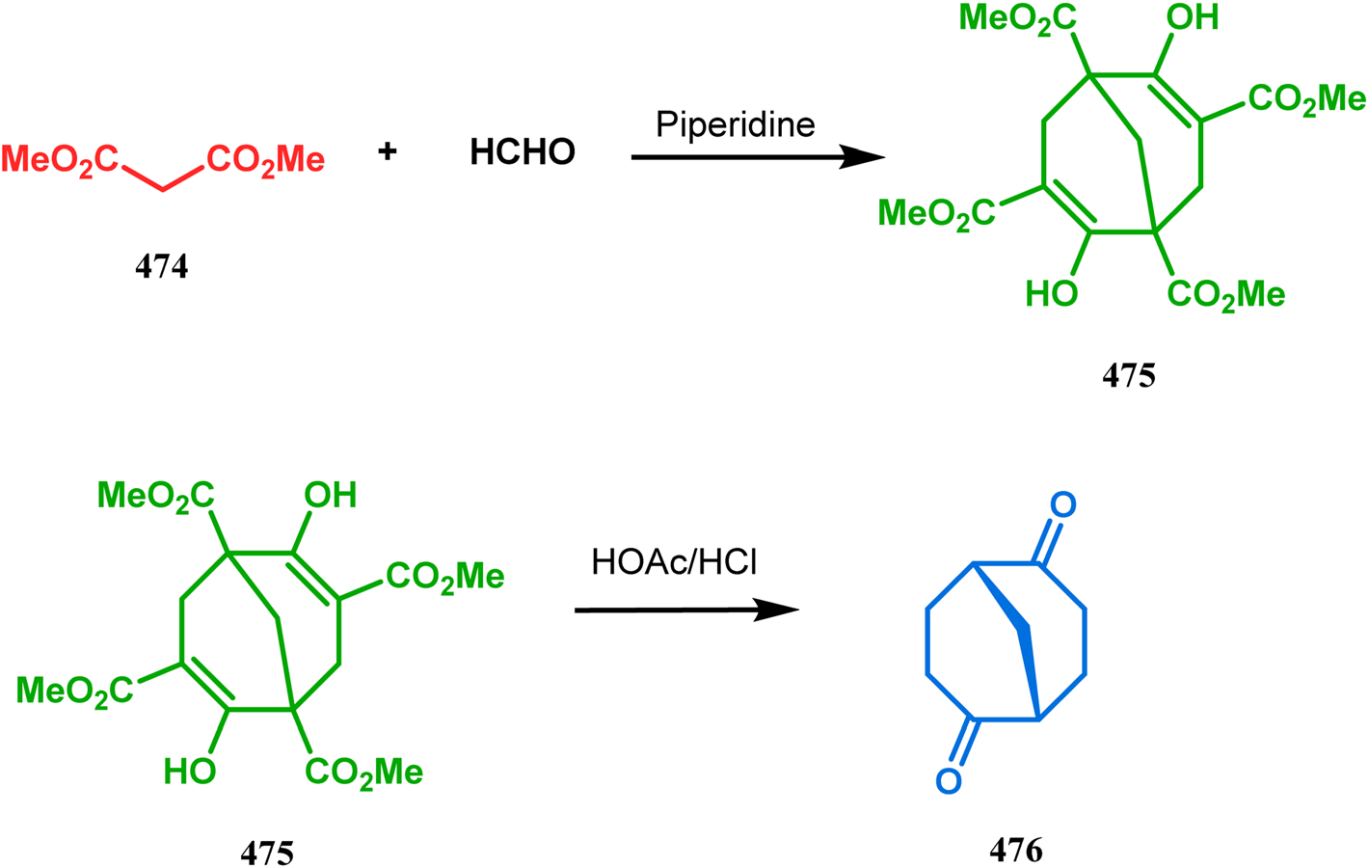
Synthesis of bicyclo[3.3.1]nonane-2,6-dione through condensation between dimethyl malonates and paraformaldehyde.

In a closely related study by Warnmark and coworkers, condensations between dimethyl malonates and paraformaldehyde have been reported. When 474 and formaldehyde were subjected toward the cyclization protocol, two formaldehyde units get condensed with two dimethyl malonate units, resulting in the synthesis of bicyclo[3.3.1]nonane 475, which upon treatment with HOAc/HCl yielded the racemic mixture of bicyclo[3.3.1]nonane-2,6-dione (476) (Scheme 109).^217^ To obtain the pure enantiomers, biotransformation technique was then employed. The fact that the use of enzymes and microorganisms to achieve this important system in their enantiomerically pure form are the most popular methodologies^218,219^ prompted Warnmark's group to apply Baker's yeast to obtain enantiomerically-pure bicyclo[3.3.1]nonane-2,6-dione in a large scale.^217^ In a similar report by Grauslund and coworkers, genetically engineered *Saccharomyces cerevisiae* cells were used as the biocatalyst for the kinetic resolution of racemic bicyclo[3.3.1]nonane-2,6-dione.^220^

#### 3.4.5. Through the solvolysis of other polycycles

**Scheme 110:**
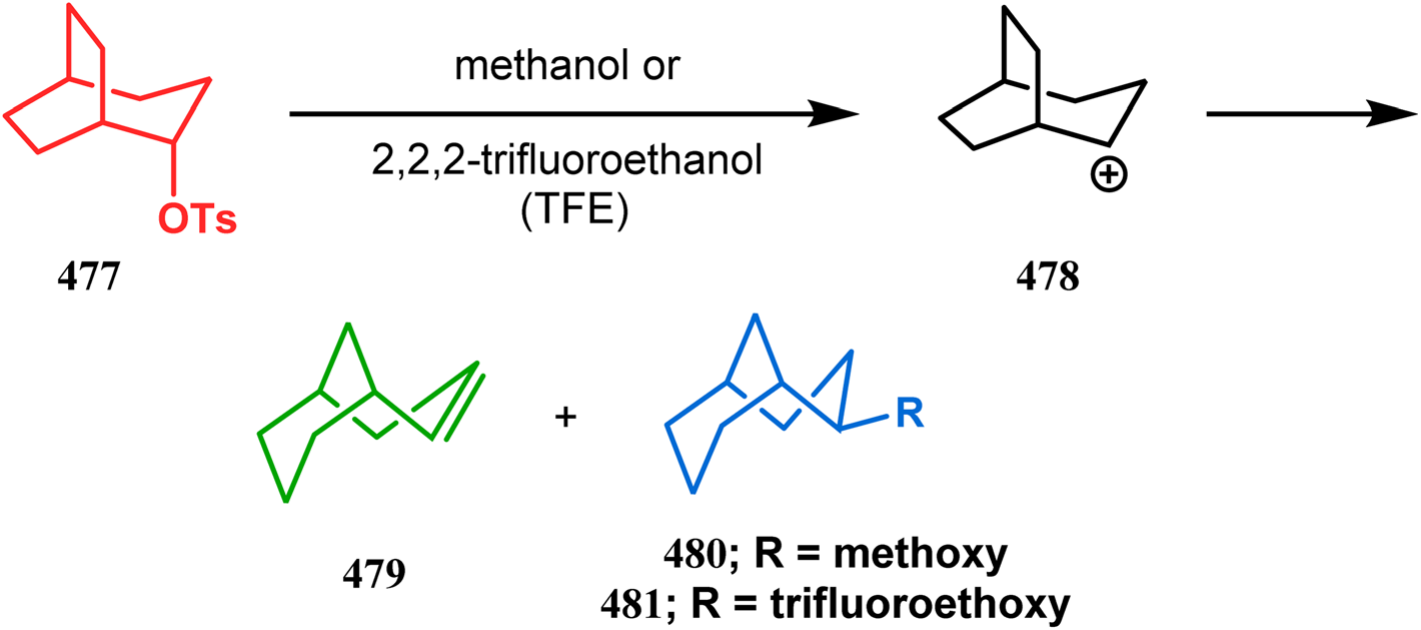
Formation of 2-bicyclo[3.3.1]nonene and *exo*-2-*R*-bicyclo[3.3.1]nonane through solvolysis.

During the solvolysis study of 2-bicyclo[3.2.2]nonyl *p*-toluenesulfonates, Okazaki's group and Schafaer's team found that the solvolysis products of such bicycles are usually enriched with bicyclo[3.3.1]nonanes.^221–224^ It was found that when 477 was solvolyzed in methanol and 2,2,2-trifluoroethanol (TFE) buffered with 2,6-lutidine, 2-bicyclo[3.3.1]nonene (479) (yield 15–47%) and *exo*-2-R-bicyclo[3.3.1]nonane (R = methoxy, 480; R = trifluoroethoxy, 481) were formed along with other bicycles in 12–23% yield. The solvolytic data indicated that these products were formed from the 2-bicyclo[3.2.2]nonyl cation (478), thereby unveiling the evidences for the formation of a classical carbocation intermediate (Scheme 110).^221^

## 4. Reactivity

### 4.1. Ring opening & rearrangement

#### 4.1.1. Baeyer–Villiger oxidation

Since the bridged bicyclic lactones are considered as important synthons for the synthesis of related natural product cores, the Bayer–Villiger oxidation of bridged polycyclic ketones have gained substantial importance. Such oxidation protocols on bicyclo[3.3.1]nonane diones are most extensively studied by Stoncius and coworkers. Their initial report was based on the regioselective transformation of differently positioned carbonyl group-containing bicycles to their corresponding lactones. The treatment of diketones 482 and 483 with mCPBA in DCM yielded the oxabicyclo[3.3.2]decanediones 487a,b and 488a,b in 92 : 8 ratio. The preferential oxidation of the C9 carbonyl group over the C2 carbonyl is determined by kinetic parameters. Thus, although the *c* isomer is thermodynamically more stable than other isomeric ketolactones (according to AM1 calculations), larger torsional strain release during the formation of tetrahedral intermediate through C9 oxidation than that through C2 oxidation (as the former carbonyl is more distorted from sp^2^ hybridization than the latter) favored the *a* and *b* isomers over *c* and *d*. The difference in the migratory aptitudes between C1–C9 and C5–C9 bonds then determines the *a* : *b* ratio. Clearly, the electron-withdrawing effect of both carbonyl groups on the former bond made the latter more nucleophilic, resulting in the preferential migration of the C5–C9 bond and thereby favoring the *a* isomer. This preference was, however, slightly diminished when an ester group was introduced at the C5 position and, as expected, the oxidation of 484 took a much longer completion time and yielded 489a,b in 85 : 1 ratio.

**Scheme 111:**
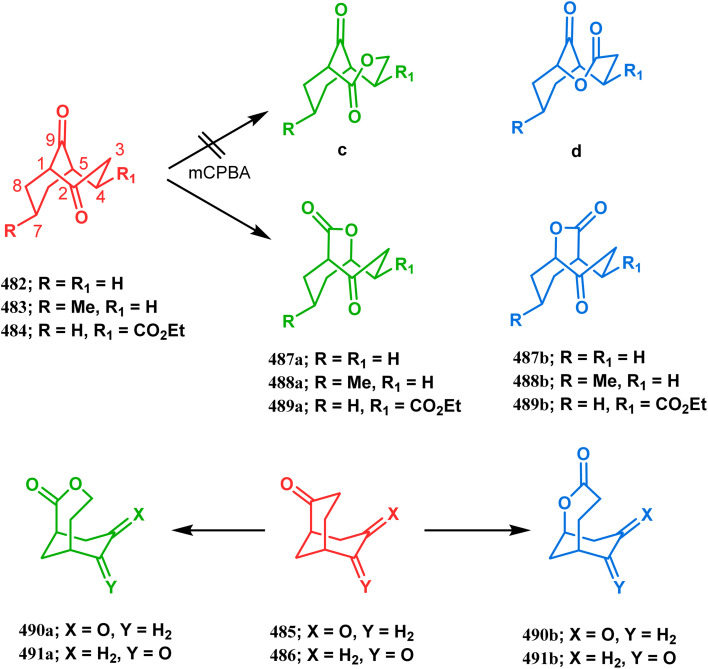
Synthesis of bridged bicyclic lactones through Bayer–Villiger oxidation.

**Scheme 112:**
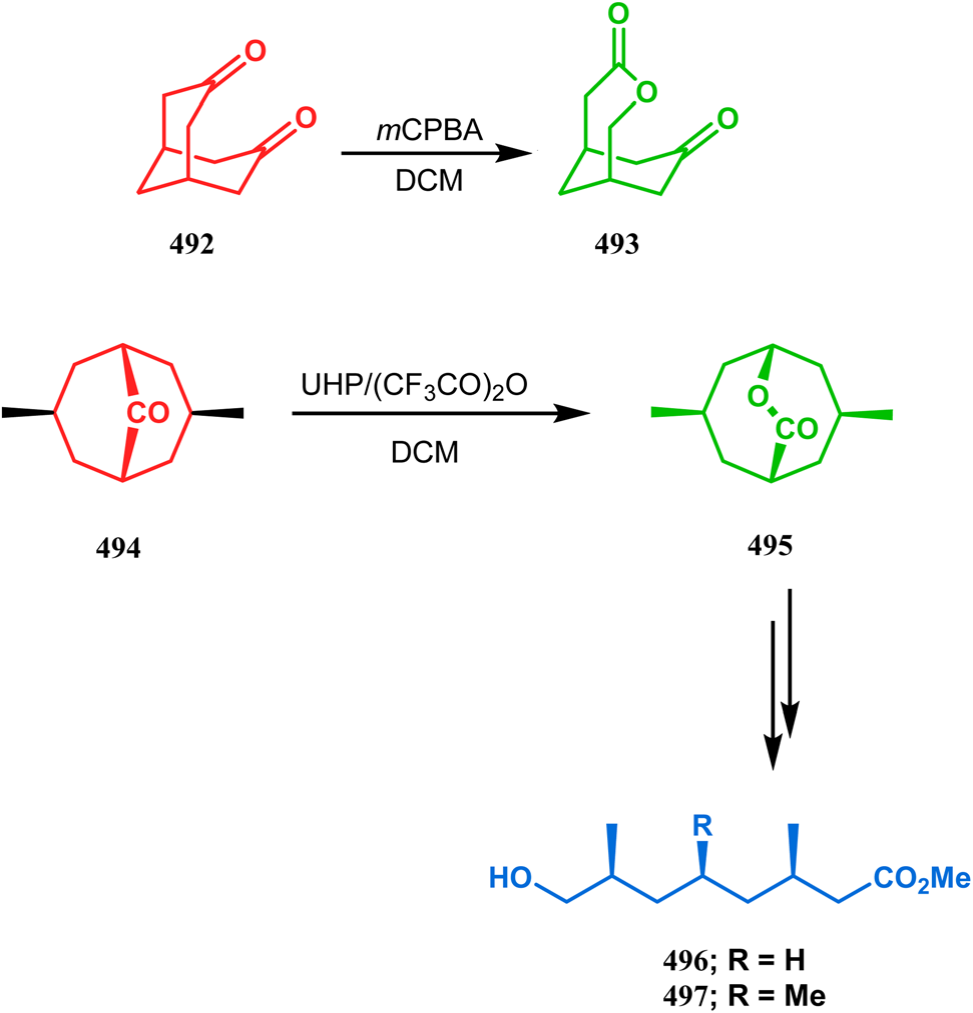
Synthesis of hydroxyacids from bicyclic lactone.

Diketones 486 also demonstrated some interesting features and when exposed to a similar oxidation protocol, the C3 carbonyl group was found to be surprisingly inactive, which could be explained by the steric hindrance caused by the C7 *endo*-proton.^225^ The preferential oxidation of the C6 carbonyl yielded oxabicyclo[4.3.1]decanedione 490a regioselectively, as expected from the lower migratory aptitude of the bridging carbon due to the electron withdrawing effect of the β-keto group. The carbonyl groups of 486 are, however, equivalent in nature and thus remove the question of preferential oxidation, but migratory competition remains and the higher migratory aptitude of the bridging carbon (due to higher strain release) produced 491b regioselectively (Scheme 111).^226,227^ The Bayer–Villiger oxidation of symmetrical dione 492 shows the simplest chemistry and produced the only possible lactone 493.^228^ The absolute configuration determination of the product lactones was also done by Stoncius's group and IR-VCD spectroscopies and chemical correlations, and the chiroptical properties of these lactones were extensively investigated to assign the configurations.^229^ Gambacorta's team also successfully synthesized a similar bicyclic lactone 495 from a simpler bicyclic ketone 494 using mCPBA-promoted Bayer–Villiger oxidation. This bicyclic lactone was then employed to synthesize their targeted hydroxyacids 496 and 497 in 95% yield (Scheme 112).^230,231^

#### 4.1.2. Other oxidative rearrangements

**Scheme 113:**
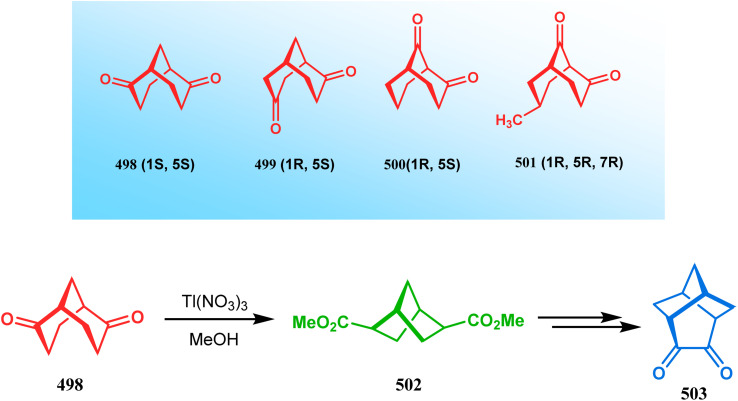
Synthesis of tricyclo[4.3.0.0]nonane-4,5-dione from enantiopure bicyclo[3.3.1]nonane-2, 6-dione with the help of Tl(NO_3_)_3_ in methanol.

Studies on the various properties of bicyclo[3.3.1]nonane-diones, especially on its absolute configuration determination, have gained substantial interest in recent times. For example, diketones 498–501 are configured properly using optical rotation and circular dichroism studies.^232–244^ The reason behind the growing concern in this area is the abundance of this bicycle in natural products and their metabolites^245,246^ and their use as precursors for the synthesis of other important bicycles. For example, thallium(iii) nitrate-promoted oxidative rearrangements are well documented in this regard in the recent literature,^247^ and its successful application in bicyclo[3.3.1]nonane chemistry was first demonstrated by Butkus and coworkers. During the beginning of this century, his team efficiently synthesized chiral tricyclo[4.3.0.0^3,8^]nonane-4,5-dione from enantiopure bicyclo[3.3.1]nonane-2,6-dione employing this oxidation protocol. Thus, when 498 was treated with Tl(NO_3_)_3_ in methanol, the diester 502 was isolated in high yields (85%), which was then sequentially converted into the targeted twistbrendanedione (503) to study its chiroptical properties through ECD and VCD spectroscopies (Scheme 113).^248^

**Scheme 114:**
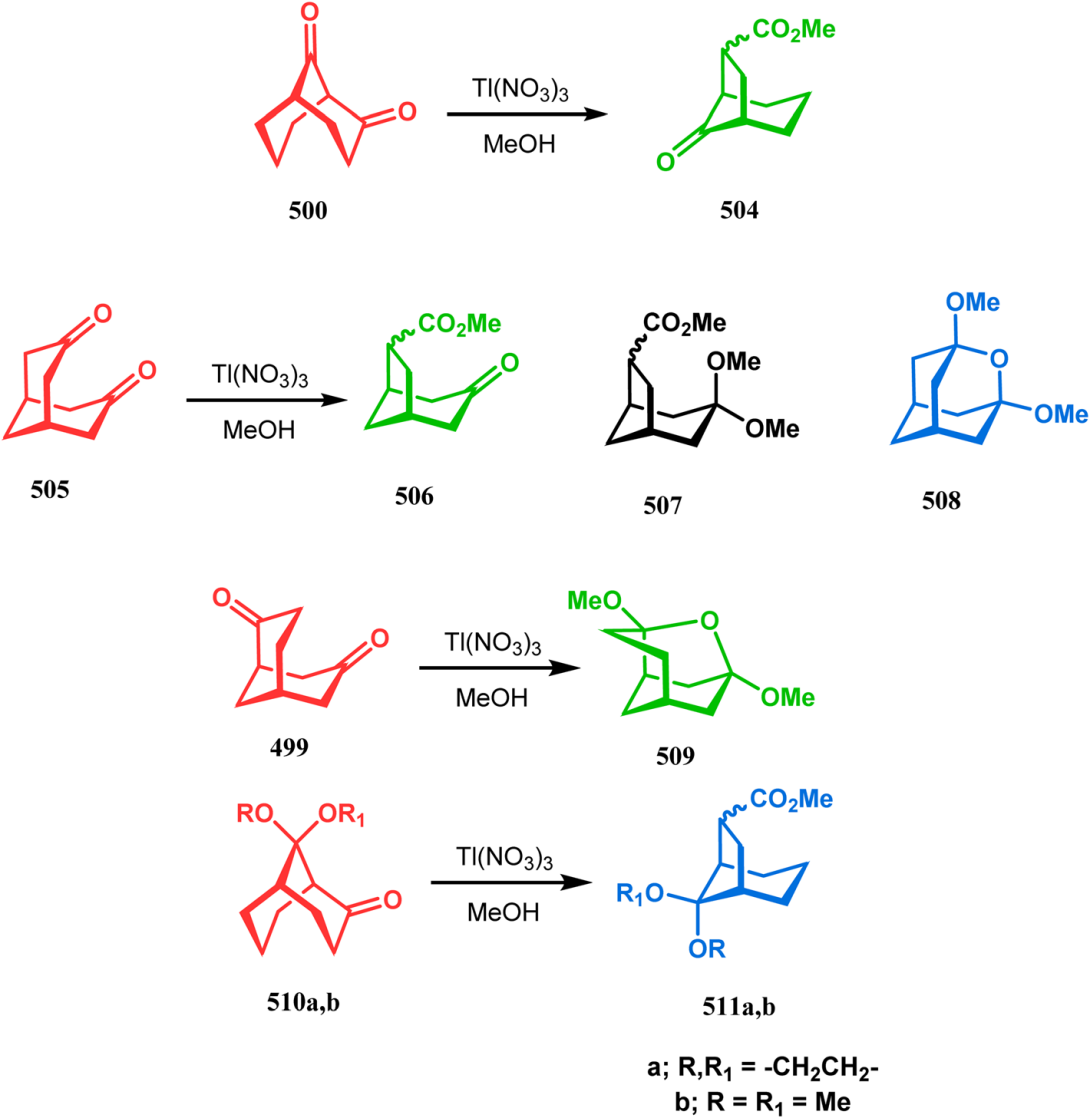
Thallium nitrate [Tl(NO_3_)_3_] oxidation of isomeric diketones for synthesizing cyclic esters or acetals.

However, the Tl(NO_3_)_3_ oxidation of other isomeric diketones gives a mixture of products. Thus, the treatment of diketone 500 in a similar oxidation protocol produced the monocarboxylate 504 as a minor product along with other isomeric bicycles. The symmetrical diketone 505 also gives a mixture of compounds, namely, the oxidative rearrangement product 506, its acetal 507, and an oxaadamantane-type tricyclic acetal 508. The formation of 508 was explained by faster transannular cyclization prior to oxidation due to the close proximity of the carbonyl groups. Similar tricyclic acetal (509) was also isolated when another diketone 499 was subjected to an identical oxidation protocol. The oxidation of monoketones, however, describes a simpler chemistry, for example, the thallium nitrate oxidation of monoketones 510a,b mainly gives the diastereomeric mixture of esters 511a,b (Scheme 114).^249^

**Scheme 115:**
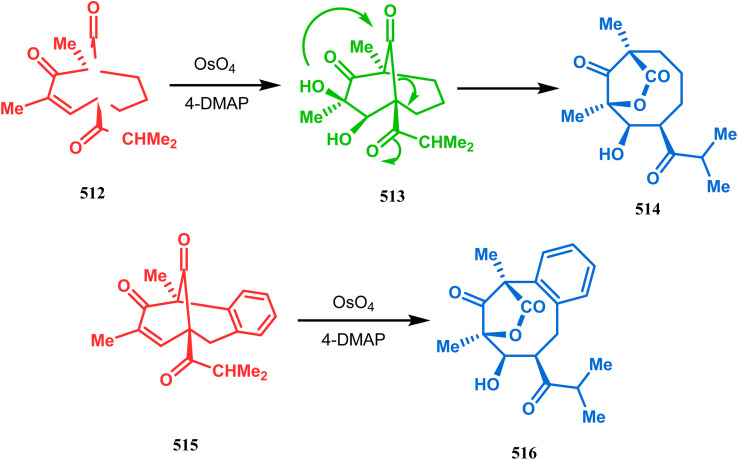
Synthesis of bicyclo[5.2.1]decane and benzo-fused bicyclo[5.2.1]decane through the oxidative fragmentation and concomitant cyclization of bicyclo[3.3.1]nonane diketones.

Nicolaou and coworkers demonstrated a different type of oxidative fragmentation and concomitant cyclization process on similar bicyclo[3.3.1]nonane diketones. Initially, the OsO_4_-mediated dihydroxylation on the double bond of 512 gave the dihydroxy-diketone 513. Immediate intramolecular lactonization and concurrent ring opening reaction promoted by 4-DMAP then yielded the envisioned bicyclo[5.2.1] decane (514). The study was further extended on benzo-fused bicyclo[3.3.1]nonane precursor 515, which under similar conditions produced 516, a benzo-fused bicyclo[5.2.1] decane (Scheme 115).^86^

### 4.2. Ring closing cyclizations

#### 4.2.1. Synthesis of adamantane-type ring systems

**Scheme 116:**
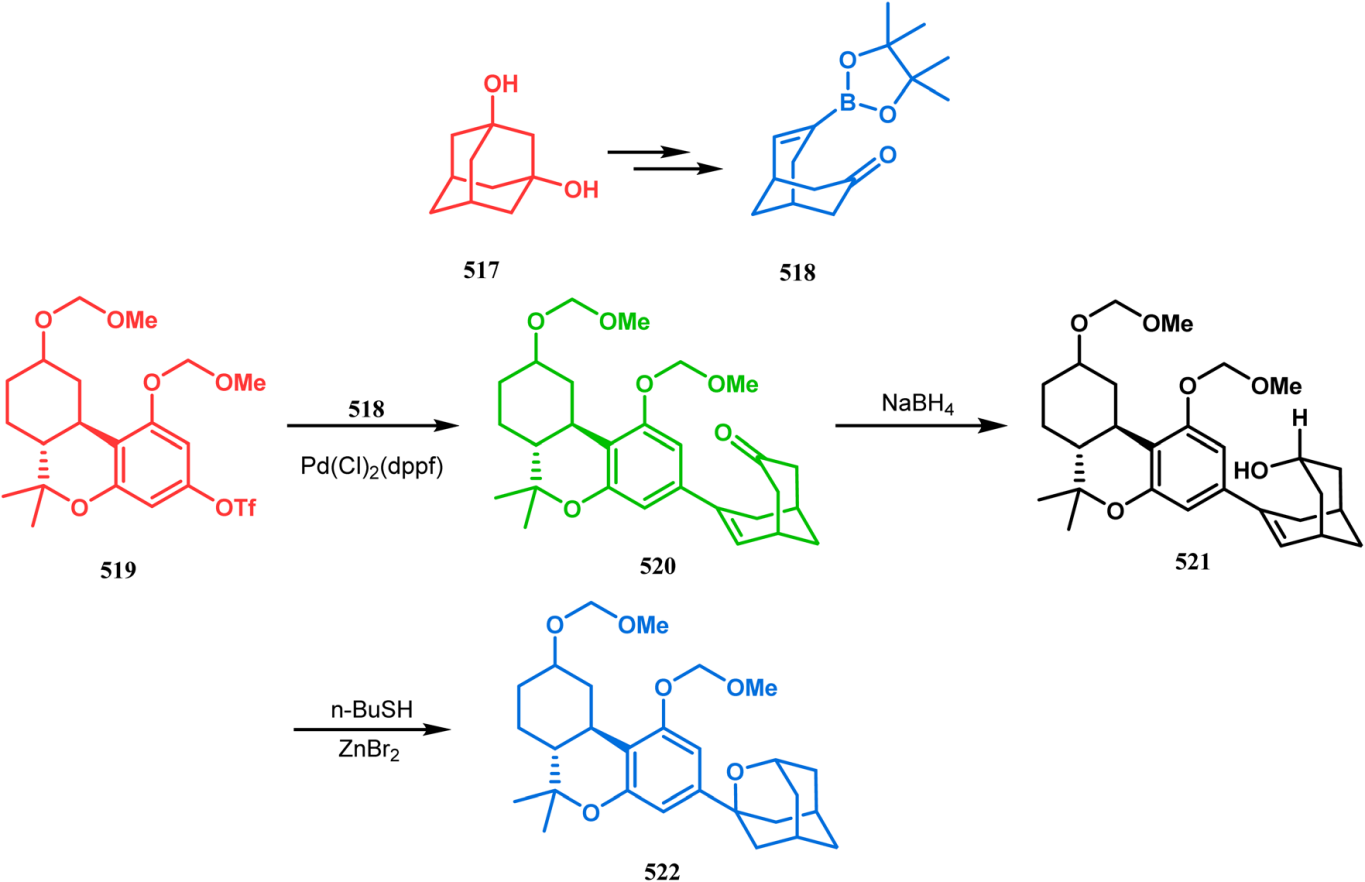
Synthesis of the oxaadamantyl cannabinoid system through reduction and intramolecular ring-closing cyclization process.

**Scheme 117:**
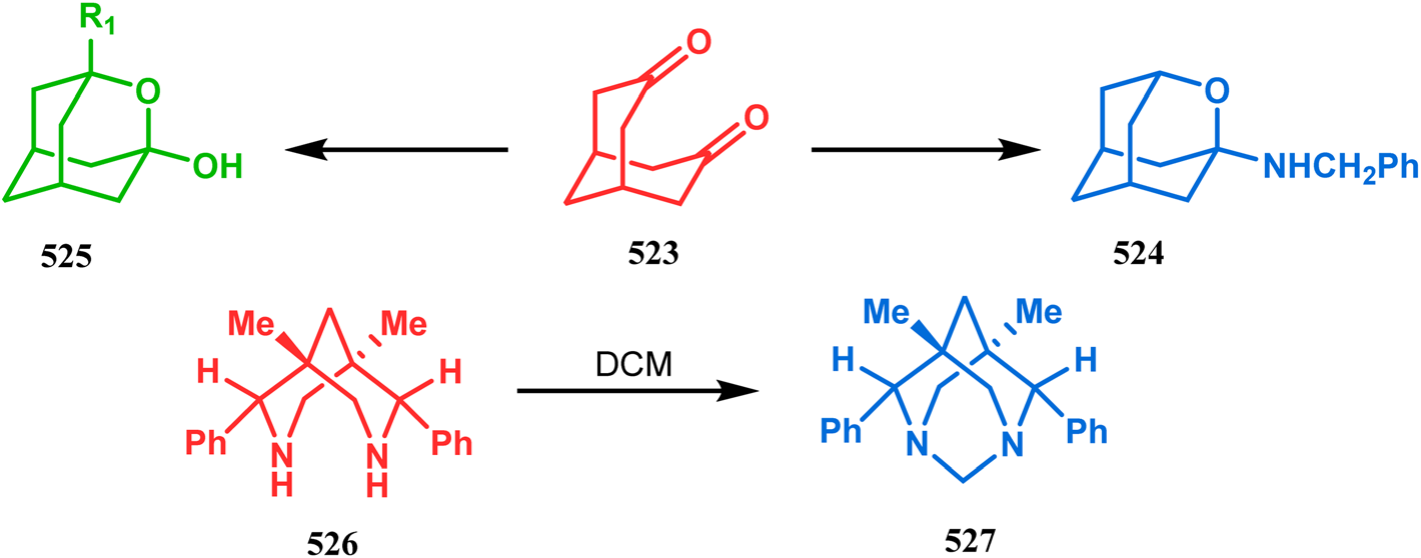
Synthesis of oxadamantyl amine, oxaadamantanol, and heteroadamantyl system by ring-closing cyclization.

Ring-closing cyclization technique to achieve adamantyl and heteroadamantyl systems is one of the most popular tools used in bicyclo[3.3.1]nonane chemistry. One of such important example, as exemplified by Makriyannis and coworkers, is the synthesis of heteroadamantyl cannabinoids, known as CB1 and CB2 receptor antagonists. This group employed the preparation of the required vinyl boronate 518 from adamantanol 517 to react with 519, which yielded the cyclization precursor 520. Again, on reduction and intramolecular cyclization, 520 produced the desired oxaadamantyl cannabinoid 522 (Scheme 116).^201^ Similar oxaadamantane derivatives were also constructed by Vazquez and coworkers to evaluate their potentiality as antivirals, NMDA receptor antagonists, and trypanocidal agents. Starting from diketone 523, secondary oxadamantyl amines 524 were synthesized using an amine condensation-reduction procedure,^250–252^ which was further derivatized into other secondary and tertiary amines. Furthermore, diketone 523 was also employed to construct oxaadamantanol 525 and was used as the precursor for other oxaadamantyl amines.^253,254^ Corey and Chau also reported the synthesis of a polysubstituted diazaadamantane derivative. During the synthesis of chelating bis-amines, this heteroadamantane derivative (527) was accidentally synthesized when 526 was stored in a DCM solution (Scheme 117).^156^

**Scheme 118:**
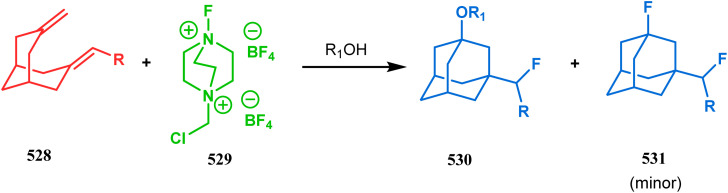
Synthesis of 1-RO-3-fluoromethyladamantanes by the reaction between 1-chloromethyl-4-fluoro-1,4-diazoniabicyclo[2.2.2]octane bis(tetrafluoroborate) and 3,7-bismethylenebicyclo[3.3.1]nonane.

**Scheme 119:**
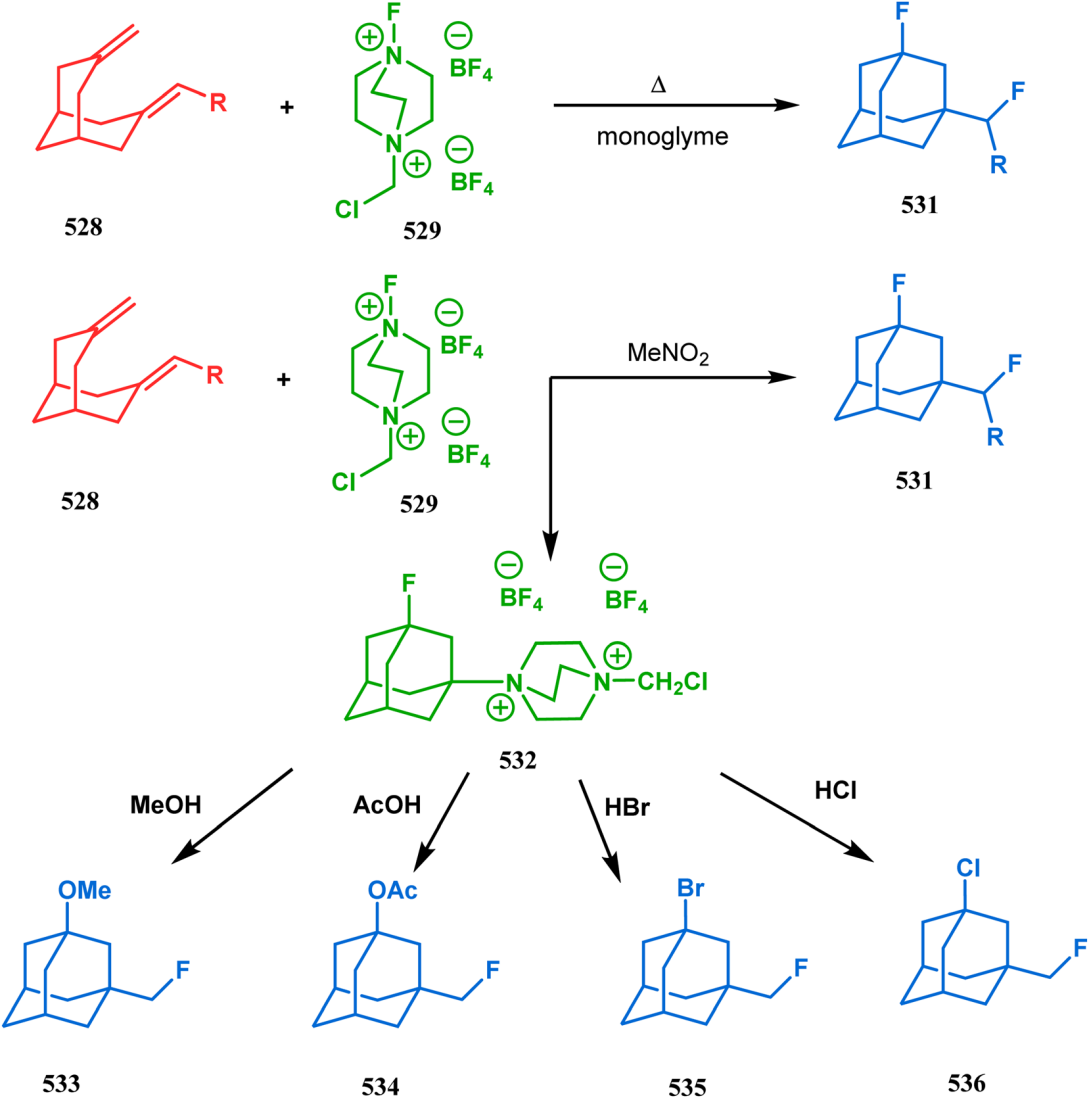
Synthesis of bromo, chloro, methoxy, or acetoxy derivatives of adamantanes by the reaction between 1-chloromethyl-4-fluoro-1,4-diazoniabicyclo[2.2.2] octane bis(tetrafluoroborate) and 3,7-bismethylenebicyclo[3.3.1]nonane in monoglymme and nitromethane solvent, followed by treating the product with HBr, HCl, MeOH, and AcOH, respectively.

Apart from the syntheses of these heteroadamantyl systems, the transannular cyclization of appropriately substituted bicyclo[3.3.1]nonanes promoted by electrophiles such as bromine and iodine is also well documented.^255–258^ The contribution of Serguchev and coworkers in this regard is remarkable. During the beginning of this century, his team unveiled their preliminary report on the reaction between 1-chloromethyl-4-fluoro-1,4-diazoniabicyclo[2.2.2]octane bis(tetrafluoroborate) (F-TEDA-BF_4_) (529) with 3,7-bismethylenebicyclo[3.3.1]nonane (528). Their investigations were carried out in ROH solvents and therefore led to the synthesis of 1-RO-3-fluoromethyladamantanes (530) (R = H, Alk, Ac) along with a slight impurity of 1-fluoro-3-fluoroalkyladamantanes (531) (yield 81–95%) (Scheme 118).^259^ Interestingly, when the same reaction partners (528 and 529) were reacted in monoglyme, the previously found minor product 531 became major and was isolated in high yields. It is believed that the reaction proceeds through the initial addition of electrolphilic fluorine to the double bond to form an adamantyl carbocation, followed by fluoride capture to give 531 (Scheme 118). The essentiality of monoglyme is attributed to the fact that unlike other solvents (DCM, THF, *etc.*), it binds to the liberated BF_3_ during the reaction to form an etherate complex and ensures the release of fluoride ion. The reaction also occurs in nitromethane solvent, but the yield of 531 was low, with 532 as the major product, which could be further derivatized into bromo, chloro, methoxy, or acetoxy adamantanes (533–536) having 50–75% yield (Scheme 119).^260^

**Scheme 120:**
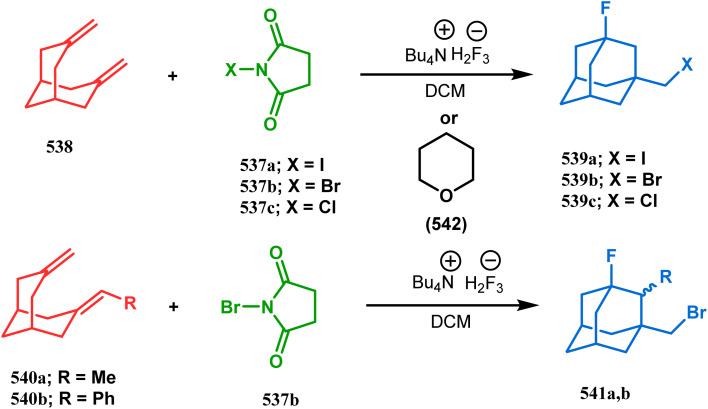
Synthesis of halo-fluoro-substituted adamantanes from bicyclo[3.3.1]nonane dienes with the use of *N*-halosuccinimides in the presence of Bu_4_N^+^H_2_F_3_^−^ in DCM.

**Scheme 121:**
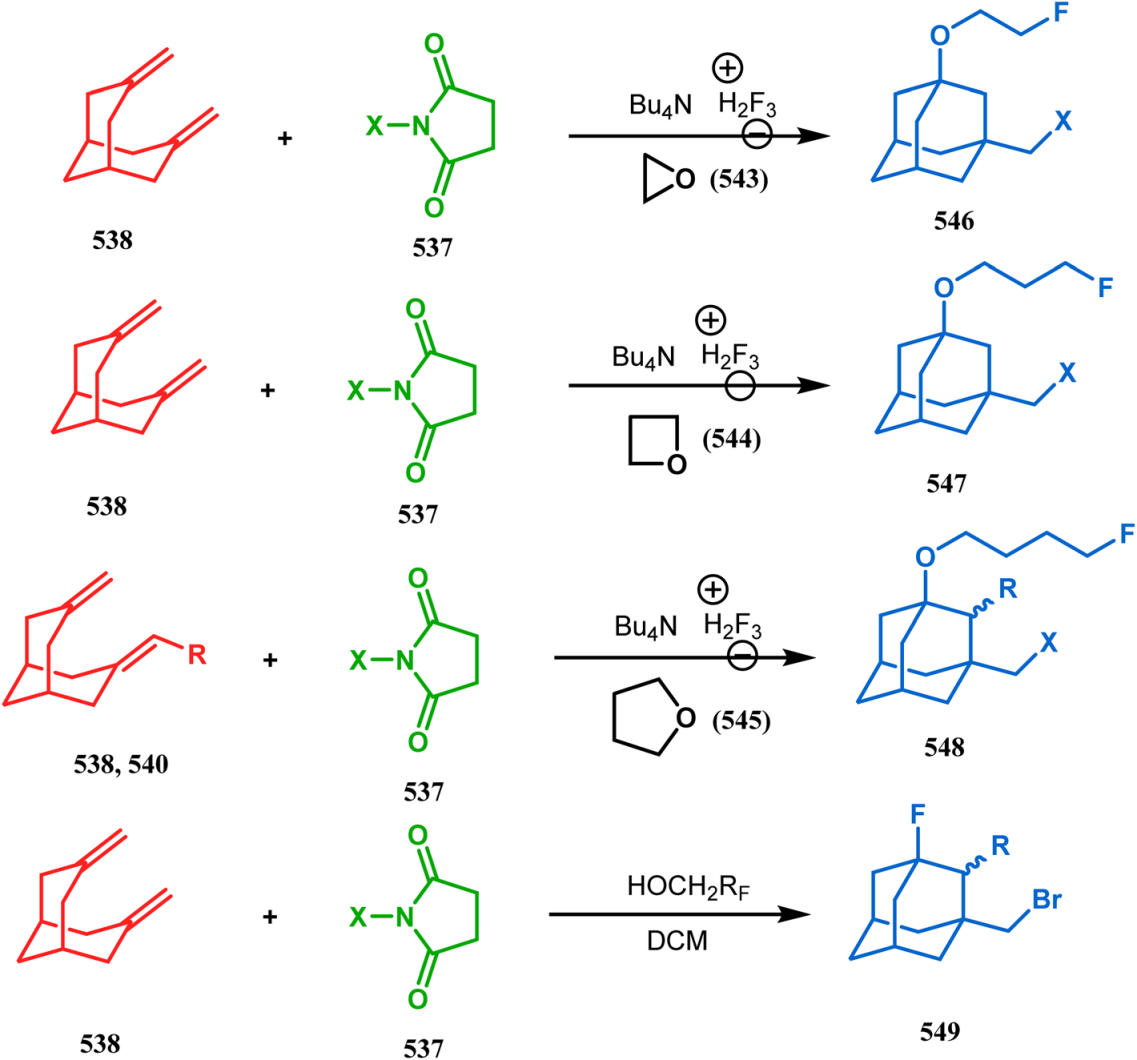
Synthesis of fluoroalkoxy adamantanes and halo-fluoro-substituted adamantanes from bicyclo[3.3.1]nonane dienes in ethylene oxide, oxetane, THF, or polyhalogenated alcohols.

Within a year, the same group published another article, where *N*-halosuccinimides (537) were employed to synthesize the halo-fluoro-substituted adamantanes from bicyclo[3.3.1]nonane dienes. Thus, when diene 538 was treated with 537 in the presence of Bu_4_N^+^H_2_F_3_^−^ in DCM, 1-fluoro-3-halomethyladamantanes 539 were produced in a radical pathway. However, unlike NIS, the ability of NBS and NCS to undergo both homolytic and heterolytic fissions lowered the possibility of radical reaction and thereby decreased the product yield from 539a to 539c (yield 25–60%). Similar reactions on substituted dienes (540) demonstrated regioselective fluorination on more substituted double bond and yielded 541 in good yields (50–65%) (Scheme 120). More interestingly, when such reactions were carried out in cyclic ethers, a cascade process, leading to the formation of fluoroalkoxy adamantanes, occurs. Thus, when 538 and 540 was subjected to similar reaction conditions in ethylene oxide (543), oxetane (544), or THF (545), the products formed were fluoroalkoxy-halomethyl adamantanes (546–548). However, a similar reaction in tetrahydropyran (542) produces usual products, possibly due to its higher solubility. Similar haloalkoxyproducts (549) were also obtained when this reaction was performed in polyhalogenated alcohols (Scheme 121).^261^

#### 4.2.2. Synthesis of noradamantane derivatives

**Scheme 122:**
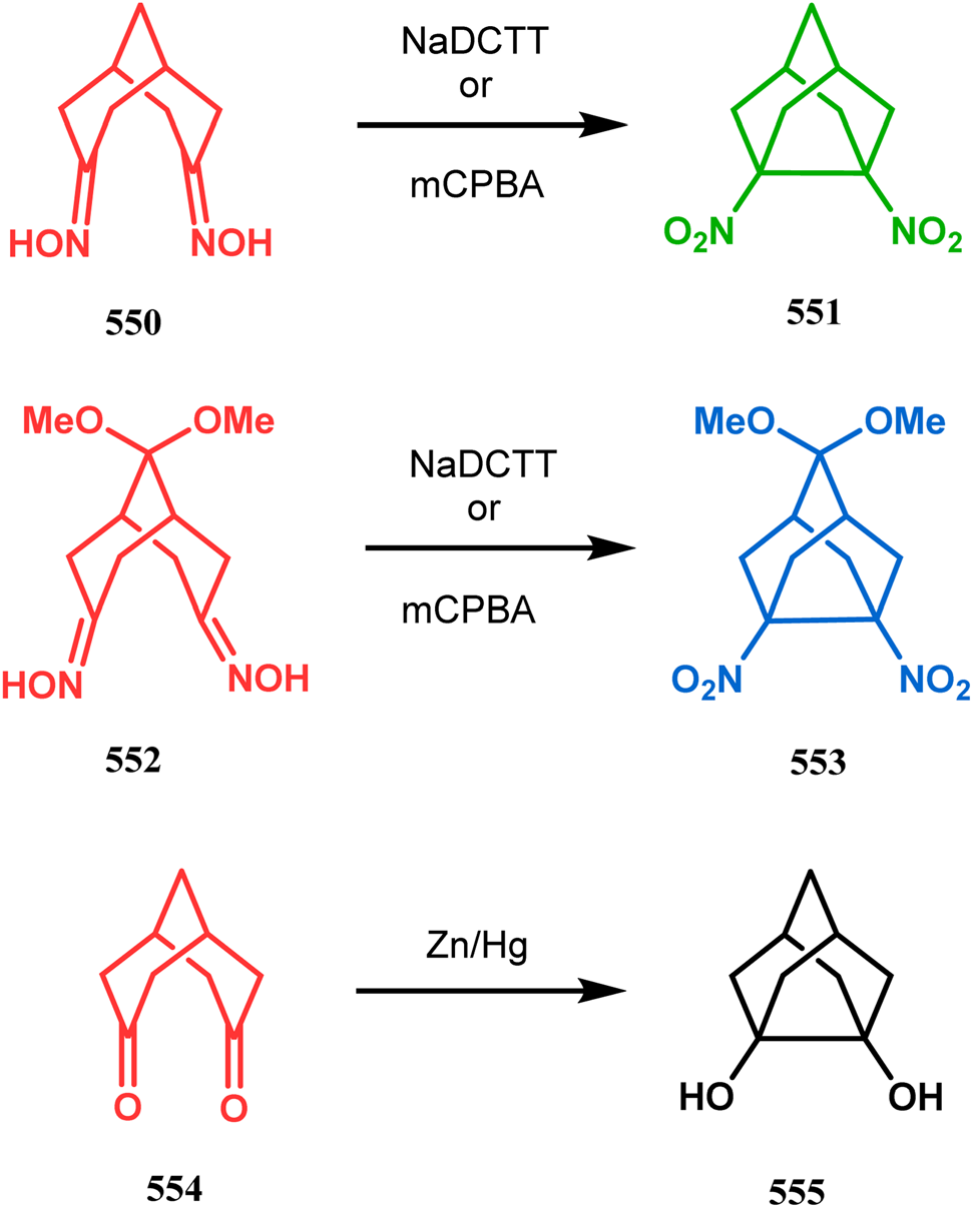
Synthesis of different types of noradamantane derivatives.

The intramolecular cyclization of appropriately functionalized bicyclo[3.3.1]nonanes is also an important tool to produce noradamantane derivatives. Zajac and coworkers made a substantial contribution in this regard. Their initial investigations on 1-sodio-3,5-dichloro-1,3,5-triazine-2,4,6(1H,3H,5H)-trione (NaDCTT) promoted the transformation of diketoxime 550 into the corresponding bis-geminal chloronitro compounds also identified a small amount 3,7-dinitronoradamantane 551 formed during the reaction.^262^ A more elaborated study in this regard revealed that the employment of mCPBA produces the noradamantane 552 as the sole product with good yields.^263^ The use of this peracid was further successfully utilized by Zajac's team to synthesize another noradamantane 553 from 9,9-dimethoxybicyclo[3.3.1]nonane-3,7-dione (552) in high yields.^264^ Reports on the synthesis of a similar noradamantane derivative 555 (yield 85%) from diketone 554 are also found in the literature (Scheme 122).^265–269^

**Scheme 123:**
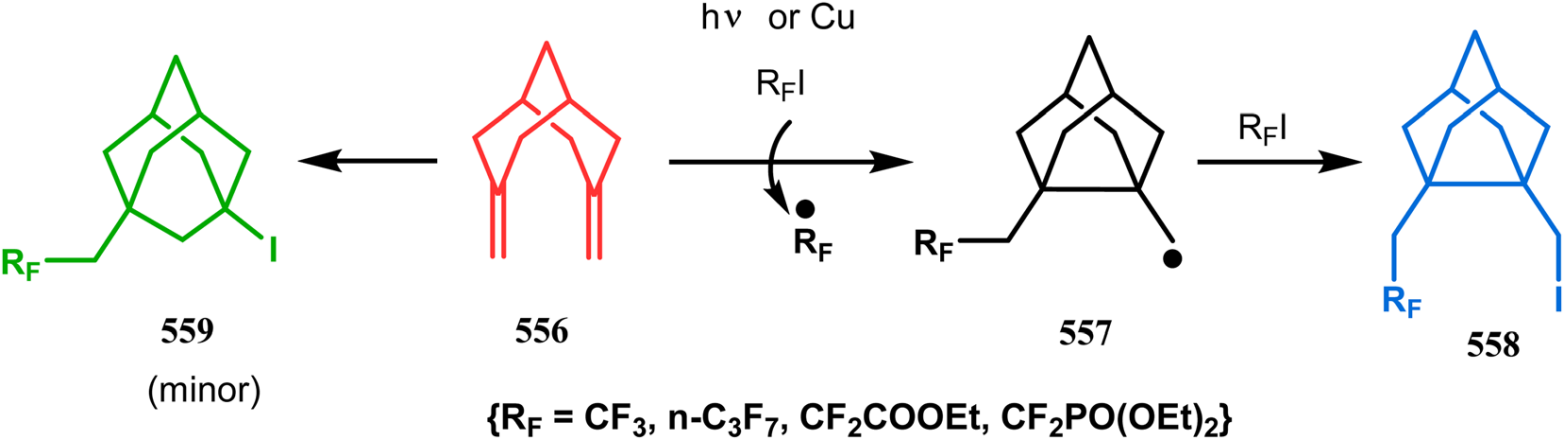
Synthesis of substituted noradamantanes by the transformation of 3,7-dimethylenebicyclo[3.3.1]nonane through the transannular cyclization technique *via* a radical-mediated pathway.

The transformation of 3,7-dimethylenebicyclo[3.3.1]nonane into substituted noradamantanes through the transannular cyclization technique could also be accomplished *via* a radical-mediated pathway. Serguchev and coworkers described one such unique example employing polyfluoroalkyl radicals. Thus, when 556 was exposed toward UV-radiation or Cu-catalysis with R_F_I agents {R_F_ = CF_3_, *n*-C_3_F_7_, CF_2_COOEt, CF_2_PO(OEt)_2_}, corresponding noradamantanes (558) were isolated in quantitative yields. Both the pathways involve the initiation with the formation of the R_F_ radical, which then reacts with 556 to give radical 557. Among the two possible cyclization pathways, namely, the *exo*-trig and *endo*-trig cyclization, the former one is more favorable kinetically, and therefore occurs instantly to give noradamantanes 558. Although, *endo*-trig cyclization could have produced thermodynamically more stable adamantane product (559), the energy barrier between 558 and 559 is too high to be crossed in a high homolyzable solvent such as CBr_4_; therefore, 558 and 559 are found to form in 9 : 1 ratio in CBr_4_ compared to the 3 : 1 ratio in a less homolyzable solvent CCl_4_.^270^ Once this iodomethyl-noradamantane is formed, it was efficiently derivatized into a diverse range of noradamantane derivatives (Scheme 123).

**Scheme 124:**
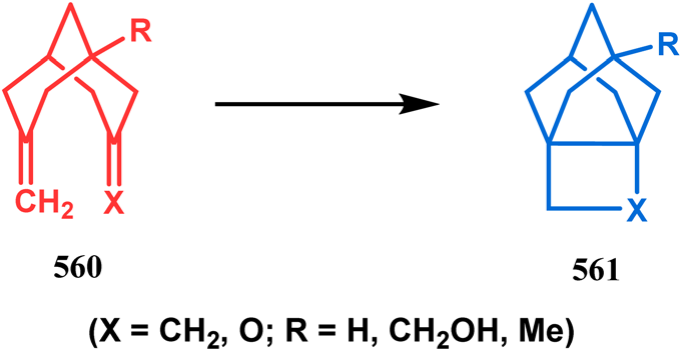
Synthesis of cyclobutene-fused noradamantane-type ring systems through the intramolecular cyclization of 560.

**Scheme 125:**
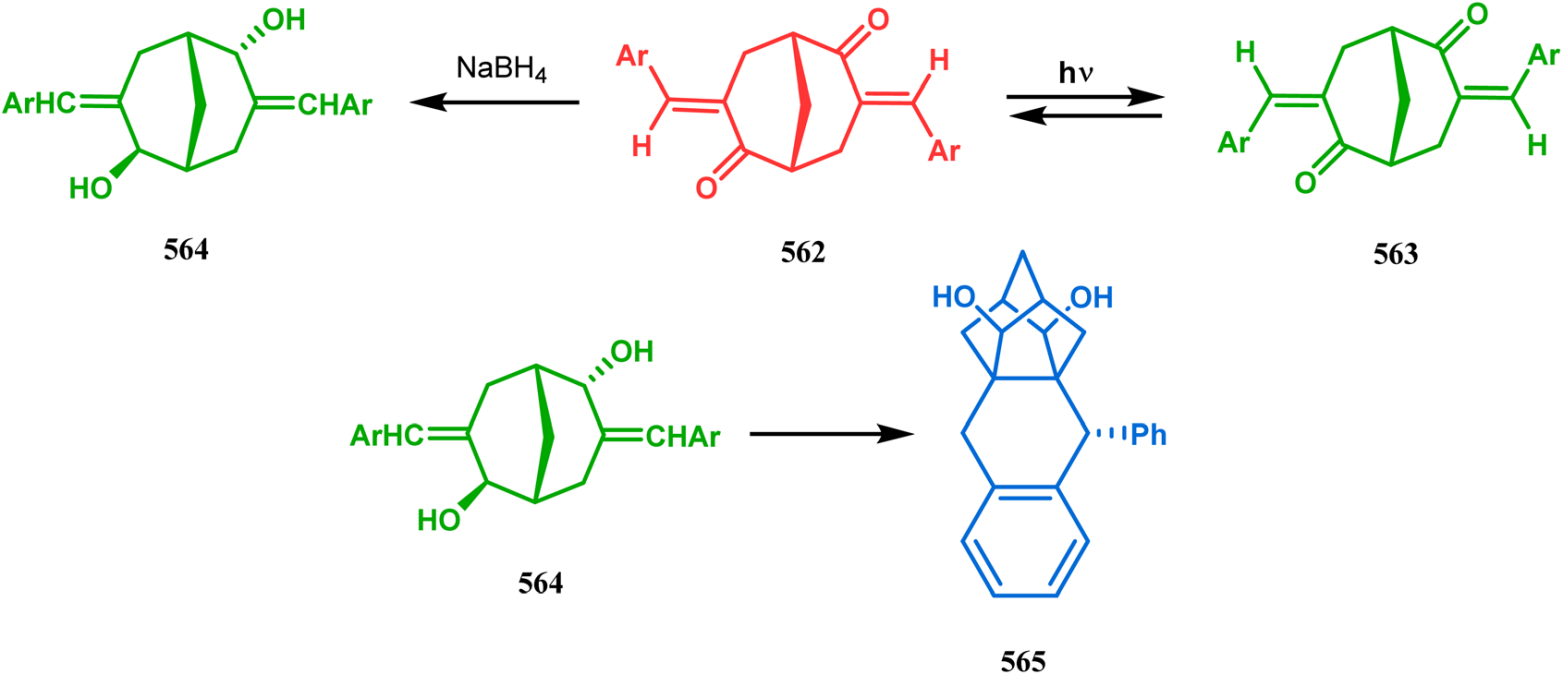
Synthesis of *exo*-7,*endo*-10-dihydroxy-2-phenyl-3,4-benzotetracyclo [4.3.3.1.0] tridecane from a diketodiene upon irradiation of light.

The photochemistry of bicyclo[3.3.1]nonanes having parallel exocyclic double bonds in 3 and 7 positions exhibits some unique observations. Several reports on the intramolecular cyclization of such compounds (560) to give cyclobutane-fused noradamantane-type ring systems (561) (Scheme 124) appeared in the literature during the 1970s.^271,272^ This century also began with one such unique example, exemplified by Averina and coworkers. Their study began from diketodiene 562, which on irradiation transformed into its less stable *cis–cis* isomeric form (563), and no cycloaddition product was formed. However, when the ether solution of diol 564, obtained by the reduction of 562, was subjected to similar reaction conditions, the product becomes *exo*-7,*endo*-10-dihydroxy-2-phenyl-3,4-benzotetracyclo-[4.3.3.1^8,11^.0^1,6^]tridecane (565) (Scheme 125).^273^

#### 4.2.3. Synthesis of other polycycles

**Scheme 126:**
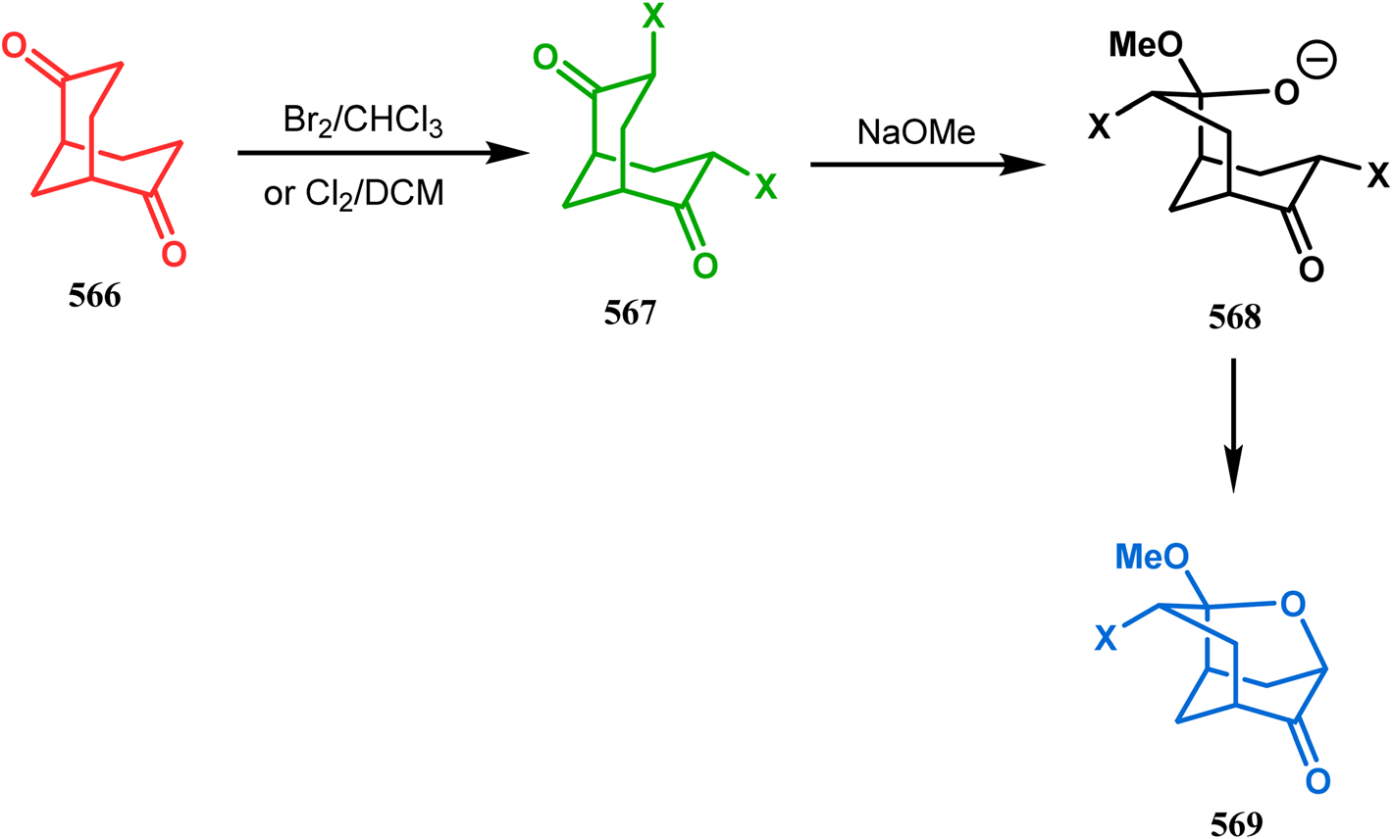
Synthesis of oxatricyclo[4.3.1.0]decanes through the ring closure reactions of α,α′-dihalobicyclo[3.3.1]nonane-diones under Favorskii reaction conditions.

Ring closure reactions of α,α′-dihalobicyclo[3.3.1]nonane-diones under Favorskii reaction condition is a useful protocol to achieve oxatricyclo[4.3.1.0^3.8^]decanes. Butkus and coworkers established this fact efficiently in a pair of subsequent communications. Starting from diketones 566, the Favorskii precursors 567 were synthesized through a simple halogenations reaction. 567, on treatment with NaOMe, formed hemiketal anion 568, which underwent a concomitant cyclization reaction to give the oxatricyclodecanes 569 (Scheme 126).^274,275^

Another interesting report of intramolecular cyclization of a chelating bis-ether was demonstrated by Corey and Chau. They found that (+) 570 on treatment with catalytic amount of *p*-TsOH or H_2_SO_4_ promotes a fascinating intramolecular dehydration reaction and yielded a crystalline twistane-type tricyclic ether 571, as confirmed by the X-ray crystallographic analysis.^156^

**Scheme 127:**
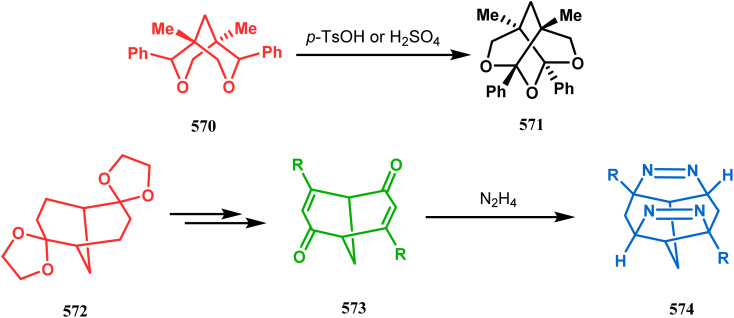
Synthesis of twistane-type tricyclic ether and tetracyclic diazene through intramolecular cyclization.

Synthesis toward a more complicated tetracyclic entity was unveiled by Bechman's team. They demonstrated an efficient route toward C-type bis(diazenes). Their journey began with diketal 572, which was sequentially converted into bicyclo[3.3.1]nonane-diketo-diene 573. The treatment of this precursor with hydrazine then yielded the targeted tetracyclic diazene 574 (Scheme 127).^276^

**Scheme 128:**
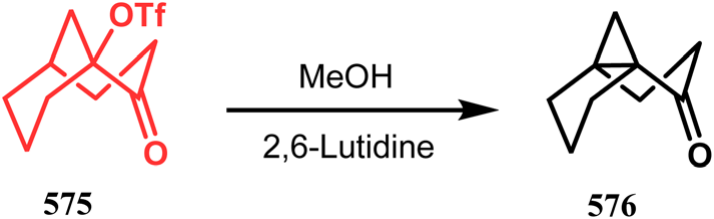
Synthesis of propallen through the solvolysis of 2-oxobicyclo[3.3.1]non-1-yl triflate.

Takeuchi and coworkers synthesized propellan-type ring systems from bicyclo[3.3.1]nonane precursors. As described in their communications, the solvolysis of 2-oxobicyclo[3.3.1]non-1-yl triflate (575) could produce propallen 576 as a minor product.^277,278^ However, other triflate, heptafluorobutyrate, tosylate, and bromo derivatives do not lead to similar propallens (Scheme 128).^279^

### 4.3. Heterocyclization

**Scheme 129:**
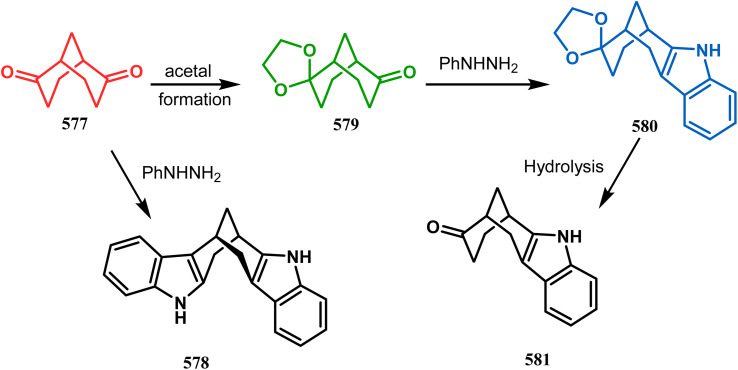
Synthesis of mono and diindolyl products by the reaction of dione and phenylhydrazine.

Heterocyclic compounds constitute more than half of the entire bioactive molecules. Therefore, the derivatization of newer ring systems into their heterocycle-fused analogues has gained immense importance in recent years; in particular, the fusions of the indole ring system with newer cyclic and acyclic core moieties were investigated extensively by several research groups. Bicyclo[3.3.1]nonanes are also not an exception,^280–287^ and the remarkable contribution in this regard from Butkus and coworkers is well documented. Their initial reports were on the synthesis of methanocycloocta[*b*]indoles, which commenced by reacting the racemic dione 577 with phenylhydrazine. The reaction proceeds with an intermediate hydrazone formation to produce the diindolyl product 578.^288,289^ The synthesis of the corresponding monoindoles is quite difficult and requires a modified approach. Thus, dione 577 was first converted into its monoacetal 579, which on treatment with phenyl hydrazine in the presence of acetic acid yielded the monoindolyl monoketal 580. The desired monoindole 581 was obtained by hydrolyzing 580 (Scheme 129).^289^

**Scheme 130:**
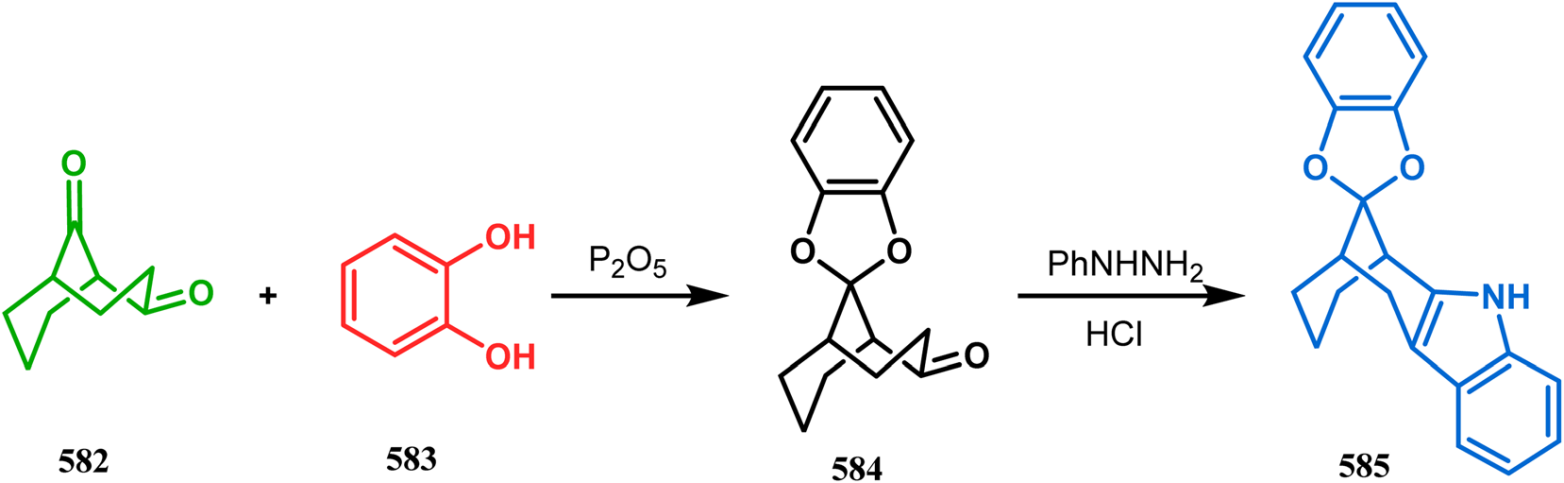
Synthesis of chiral 1′*H*-spiro[1,3-benzodioxole-2,12′-[6′,10′]methanocyclooct [*b*]indole after the monoprotection of diketone with catechol, followed by indolization with phenylhydrazine in the presence of HCl.

Within a couple of years of this report, the synthesis and absolute configuration determination of a spiro[1,3-benzodioxole-methanocyclooct[*b*]indole] was demonstrated by the same group. The synthesis of this monoindolyl derivative (585) also required the monoprotection of diketone 582. Thus, the regioselective protection of 582 with catechol (583)^290^ yielded the protected ketone 585, which on indolization with phenylhydrazine in the presence of HCl (cat. amt.) afforded the chiral 1′H-spiro [1,3-benzodioxole-2,12′-[6′,10′]methanocyclooct [*b*]indole 585 in high yields (Scheme 130).^291^

**Scheme 131:**
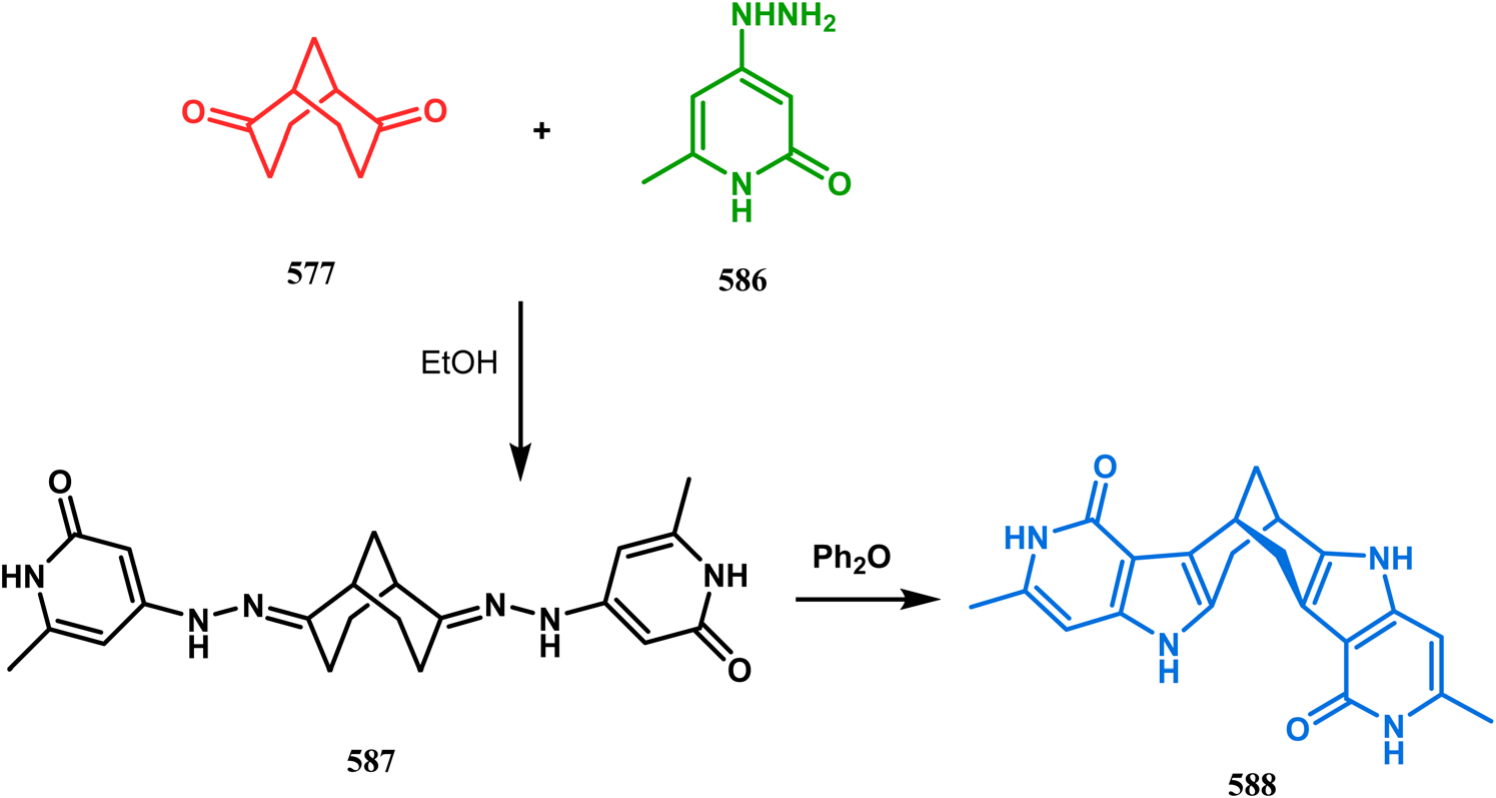
Synthesis of 2,9-diaza-3,10-dimethyl-1,8-dioxo2,5,6,7,9,12,13,14-octahydro-6,13-methanocycloocta[1,2-*b*:5,6*b*′]diindole.

A couple of years later, another article from the same team unveiled the synthetic route toward a *C*_2_-symmetric methylene-bridged product 588. This time, hydrazine 586 obtained from 4-hydroxy-6-methyl-2(1*H*)-pyran-2-one^292,293^ was treated with diketone 577, and the 2,9-diaza-3,10-dimethyl-1,8-dioxo-2,5,6,7,9,12,13,14-octahydro-6,13methanocycloocta[1,2-*b*:5,6*b*′] diindole 588 was obtained through the bishydrazone 587 (Scheme 131).

**Scheme 132:**
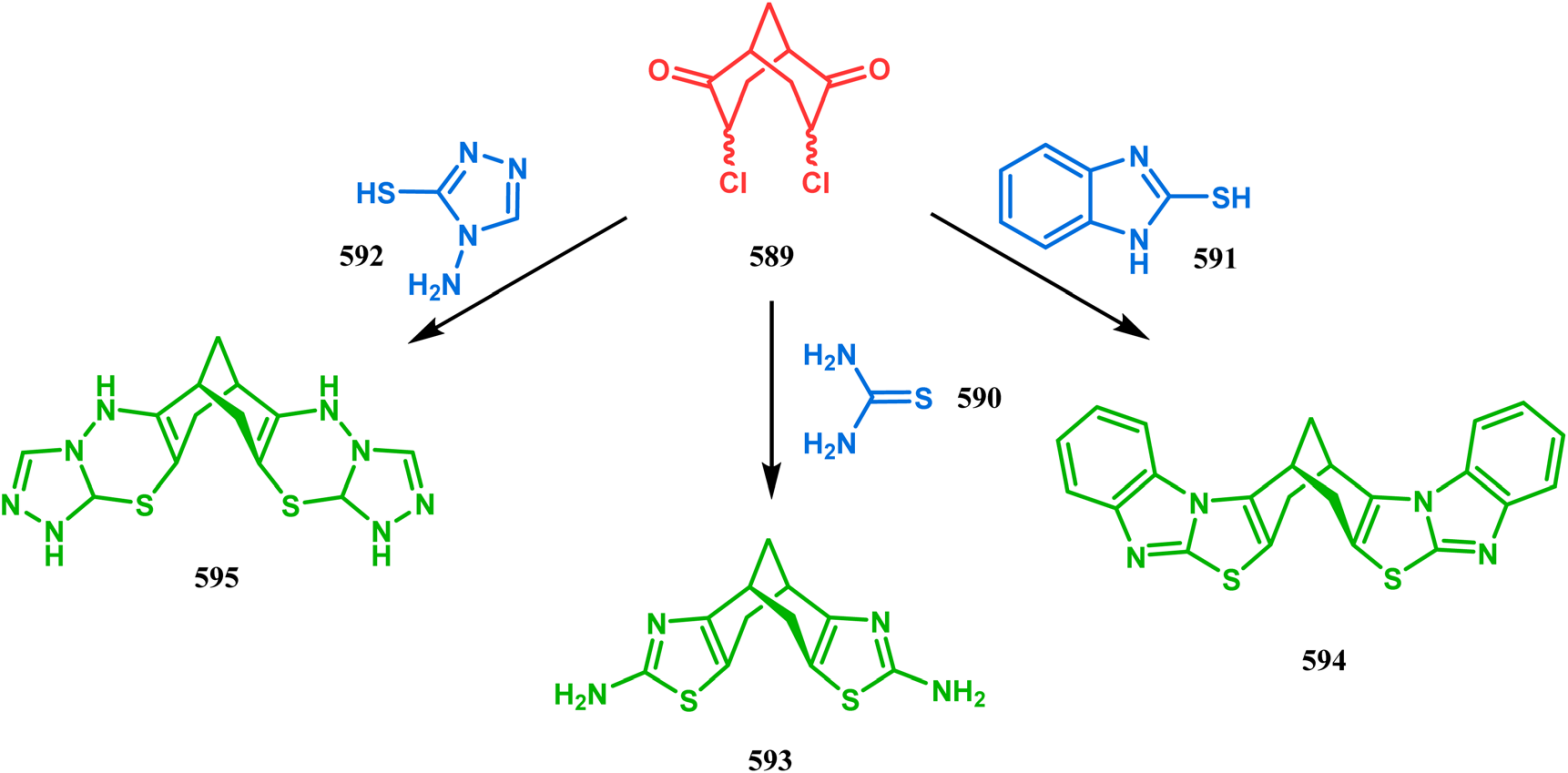
Synthesis of bicyclo[3.3.1]nonane containing bis-thiazole, bis-imidazothiazole, and bis-(triazolo-thiadiazine) from dichlorodione upon condensation with dinucloephiles.

The incorporation of such a self-complementary H-bonding motif 2-pyridone led to the self-assembled aggregation of 588 (Scheme 131),^294^ similar to the Wallentin's report^159^ on the self-aggregation of molecular tweezers. A similar approach toward a helical tubular self-aggregated *C*_2_-symmetric cleft-shaped molecule was also described by this group recently.^295^ Furthermore, a closely related report on the synthesis of similar fused heterocyclic ring-containing bicyclo[3.3.1]nonane derivatives was also published recently, where Labanauskas and coworkers efficiently demonstrated the condensation of appropriately substituted bicyclo[3.3.1]nonanones with amino-triazolothione, thiobenzimidazole, thiocarbamide, *etc.* Thus, when dichloro-dione 589 was condensed with dinucloephiles 590–592, corresponding bicyclo[3.3.1]nonane containing bis-thiazole (593), bis-imidazothiazole (594) and bis-(triazolo-thiadiazine) (595) were obtained in high yields (Scheme 132).

**Scheme 133:**
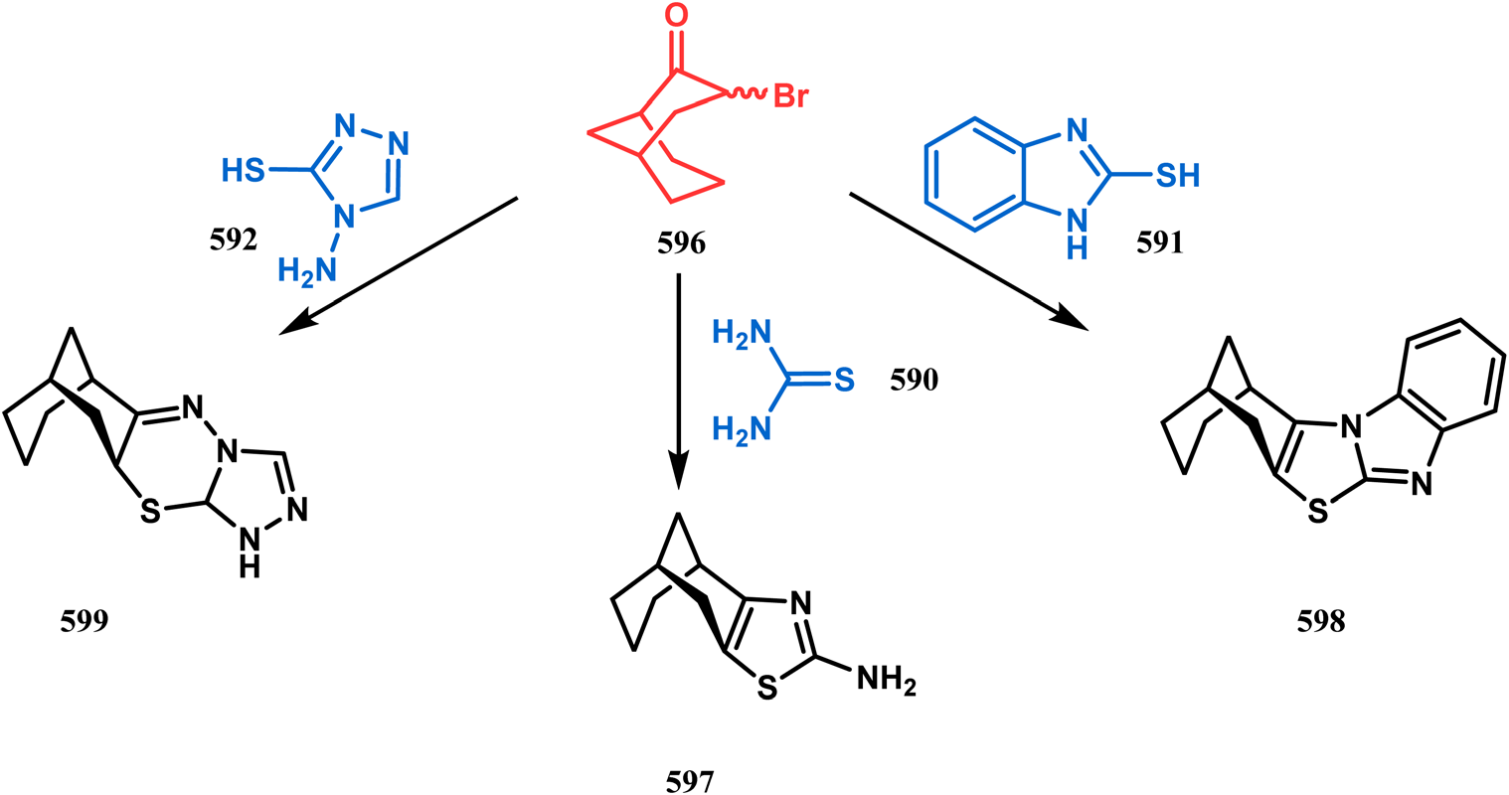
Synthesis of heterocycle-fused bicyclo[3.3.1]nonanes by the reaction of 3-bromobicyclo[3.3.1]nonane-2-one with unchanged dinucleophiles under reflux condition.

The corresponding dibromodione however produced the same products (590–592) with 10–20% lower yield, along with some other byproducts. Similar attempts to synthesize single heterocycle-fused bicyclo[3.3.1]nonanes were also successful. Thus, when 3-bromobicyclo[3.3.1]nonane-2-one (596) was refluxed with unchanged dinucleophiles (590–592), the desired singly heterocyclized bicyclo[3.3.1]nonanes (597–599) were obtained in high yields (Scheme 133).^296^

**Scheme 134:**
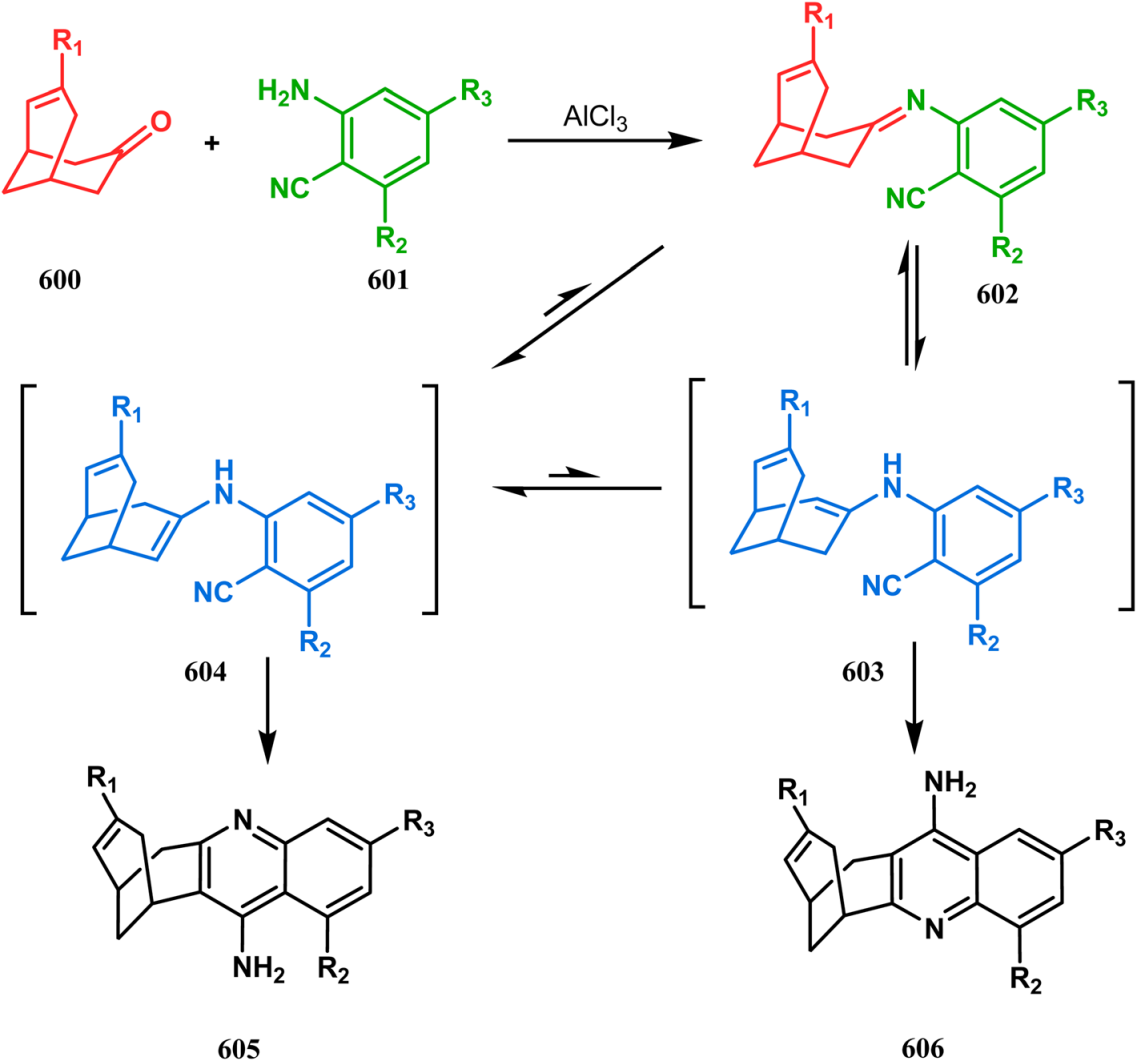
Synthesis of heterocycle-fused bicyclo[3.3.1]nonanes from bicyclo[3.3.1]nonanes upon treatment with differently substituted aminobenzonitriles.

**Scheme 135:**
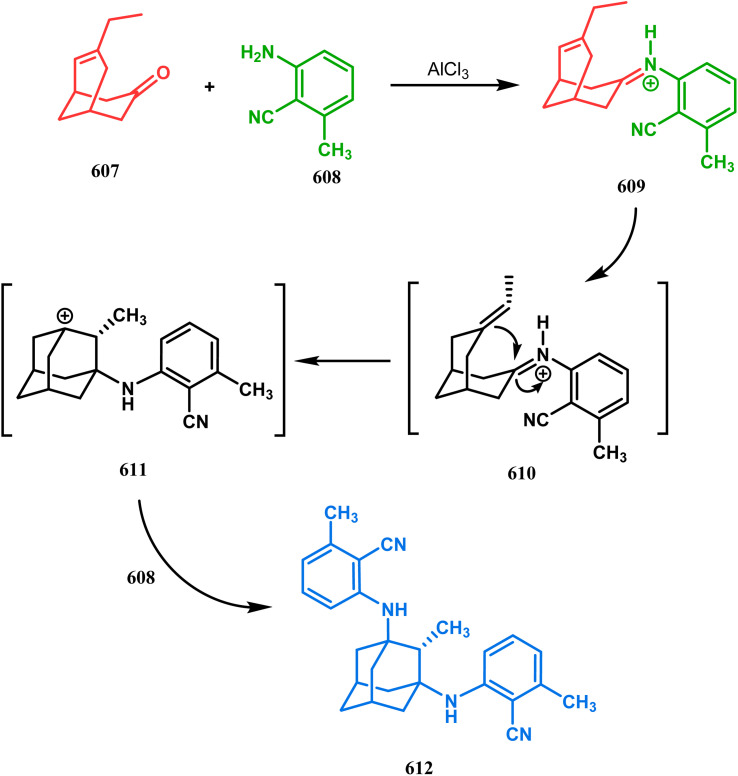
Synthesis of polyfunctionalized adamantane derivative from the reaction of bicyclo[3.3.1] nonenone with aminobenzonitrile through iminium and adamantyl carbocation formation.

Tacrine–Huperzine A hybrids also constitute an important series of heterocycle-fused bicyclo[3.3.1]nonanes. As described by Camps and coworkers, these entities (605) could easily be synthesized from bicyclo[3.3.1]nonanes (600) by treating them with differently substituted aminobenzonitriles (601).^297,298^ The reaction proceeds through initial imine formation (602), followed by intramolecular cyclization. Although the regioisomeric enamines (603, 604) could give rise to both the *syn* and *anti* aminoquinolines, thermodynamically more stable (due to the anti orientations of endocyclic double bond and heterocyclic ring) enamine 603 undergoes preferential cyclization and yields the *anti* product 606 (Scheme 134). More interestingly, when enone 607 was treated with aminobenzonitrile 608, instead of forming a new aminoquinoline analogue, a polyfunctionalized adamantane derivative (612) was produced through iminium 610 and adamantyl carbocation 611 (Scheme 135). After their successful syntheses, these Tacrine–Huperzine A hybrids were screened to check their potential for use as acetylcholinesterase inhibitors.^297^

**Scheme 136:**
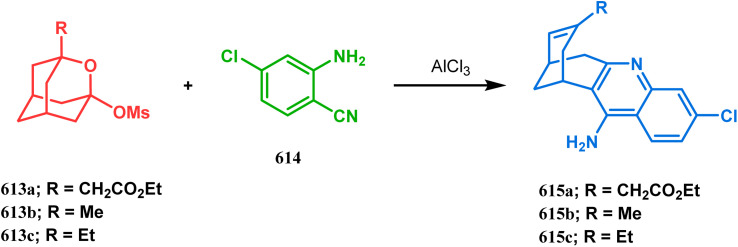
Synthesis of heterocycle-fused bicyclo[3.3.1]nonanes from oxaadamantane mesylate derivatives in one-pot fragmentation–cyclization reaction with aminobenzonitriles under refluxing condition.

Renard's team also developed some huprine scaffolds to investigate their acetylcholinesterase inhibitory properties. The syntheses of these scaffolds also utilized the same relfluxing procedure as developed by Camps's group. Thus, when different aminobenzonitriles were condensed with similar bicycle[3.3.1]nonenones, a diverse series of heterocycle-fused bicyclo[3.3.1]nonanes were produced, and their AChE inhibitory property was evaluated.^299^ Within a few months, in another communication, the same group disclosed a modified synthetic approach toward these heterocycle-fused bicyclo[3.3.1]nonanes. This time, oxaadamantane mesylate derivatives (613) were subjected to a one-pot fragmentation–cyclization reaction with aminobenzonitriles (614) under refluxing condition to produce the targeted heterocycle-fused bicyclo[3.3.1]nonanes (615) (Scheme 136).^300^

**Scheme 137:**
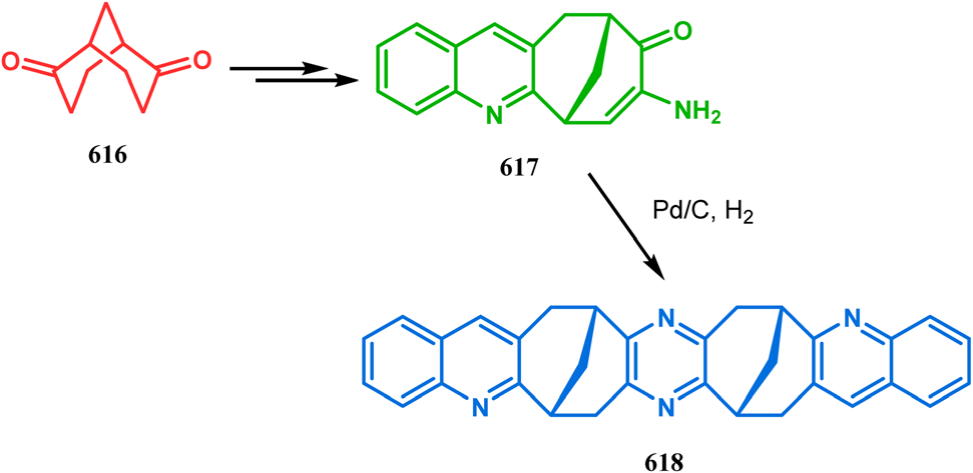
Synthesis of tweezer through the sequential conversion of quinoline-fused amine from a diketone, followed by Pd/C-mediated hydrogenation.

Warnmark and coworkers described an interesting synthetic route toward tweezer 618 to investigate its self-aggregation properties. Initially, diketone 616 was sequentially converted into quinoline-fused amine 617, which upon Pd/C-mediated hydrogenation reaction yielded the desired tweezer 618 (Scheme 137).^159^

### 4.4. Functionalization of bicyclo[3.3.1]nonanes

#### 4.4.1. Reaction with carbonyl groups

**Scheme 138:**
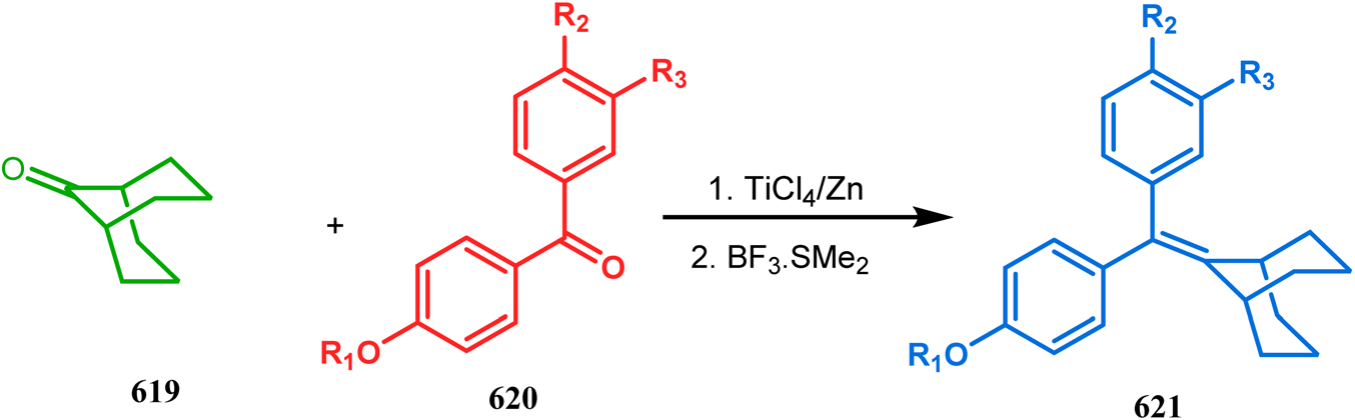
Formation of substituted alkenes of bicyclo[3.3.1]nonane from the reaction of bicyclic ketone and any other ketone by McMurry coupling reaction.

The functionalization of bicyclo[3.3.1]nonanone into diverse series of its analogues through the reaction on the ketone functionalities has been thoroughly investigated in the last few decades. Few years ago, during the investigations on the structural features of estrogen receptor, Pike and coworkers found that estrogen receptors contain an unpopulated room inside the binding pocket of the ligand,^301^ which prompted several research groups to synthesize newer classes of estrogen receptor antagonists. Appropriately substituted bicyclo[3.3.1]nonanes also found their application in this area and are generally synthesized by the McMurry coupling reaction. For example, alkenes 621 were constructed in this manner from bicyclic ketone 619 and another ketone 620 in 54–82% yield (Scheme 138).^302–305^

**Scheme 139:**
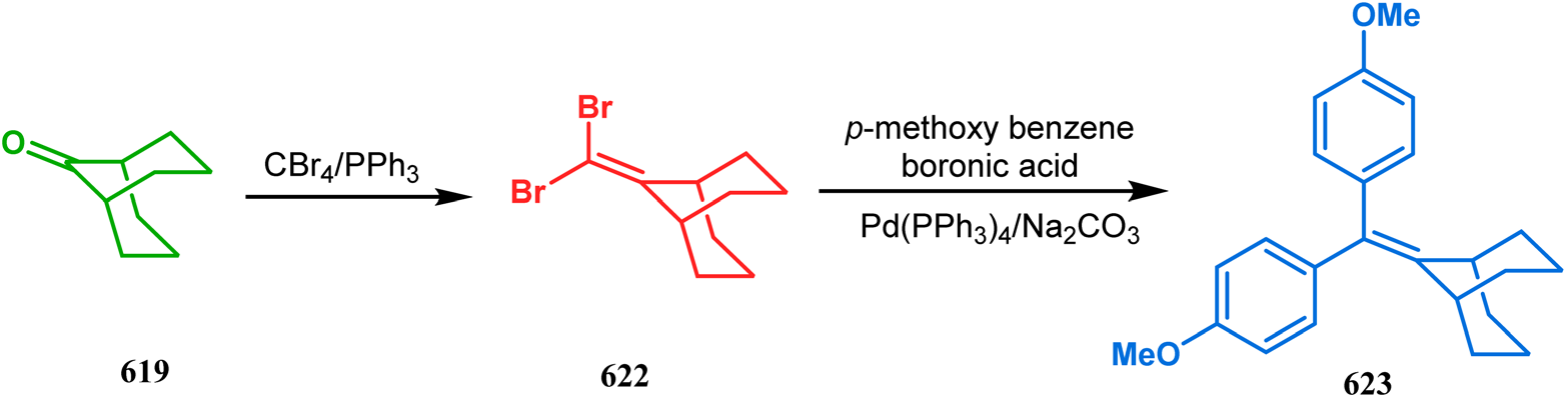
Synthesis of substituted alkenes of bicyclo[3.3.1]nonane by the Suzuki coupling reaction.

**Scheme 140:**
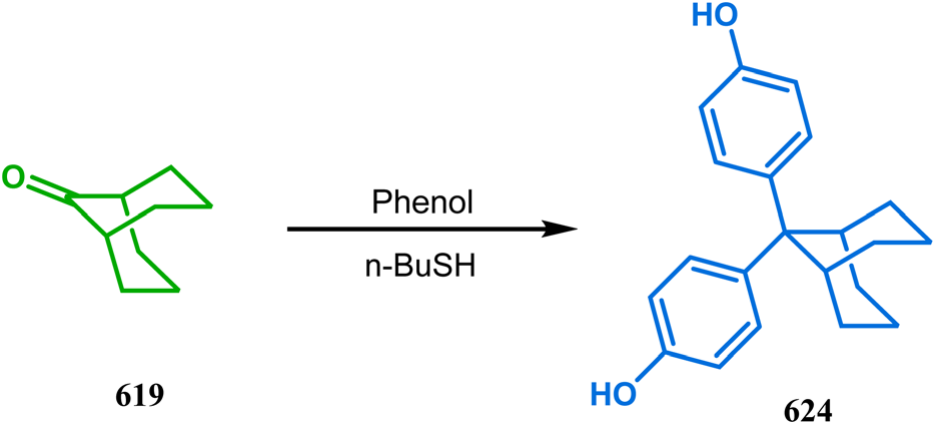
Synthesis of saturated analogue of bicyclo[3.3.1]nonane by treating bicyclo[3.3.1]nonanone with phenol in *n*-BuSH(cat.)/HCl.

**Scheme 141:**
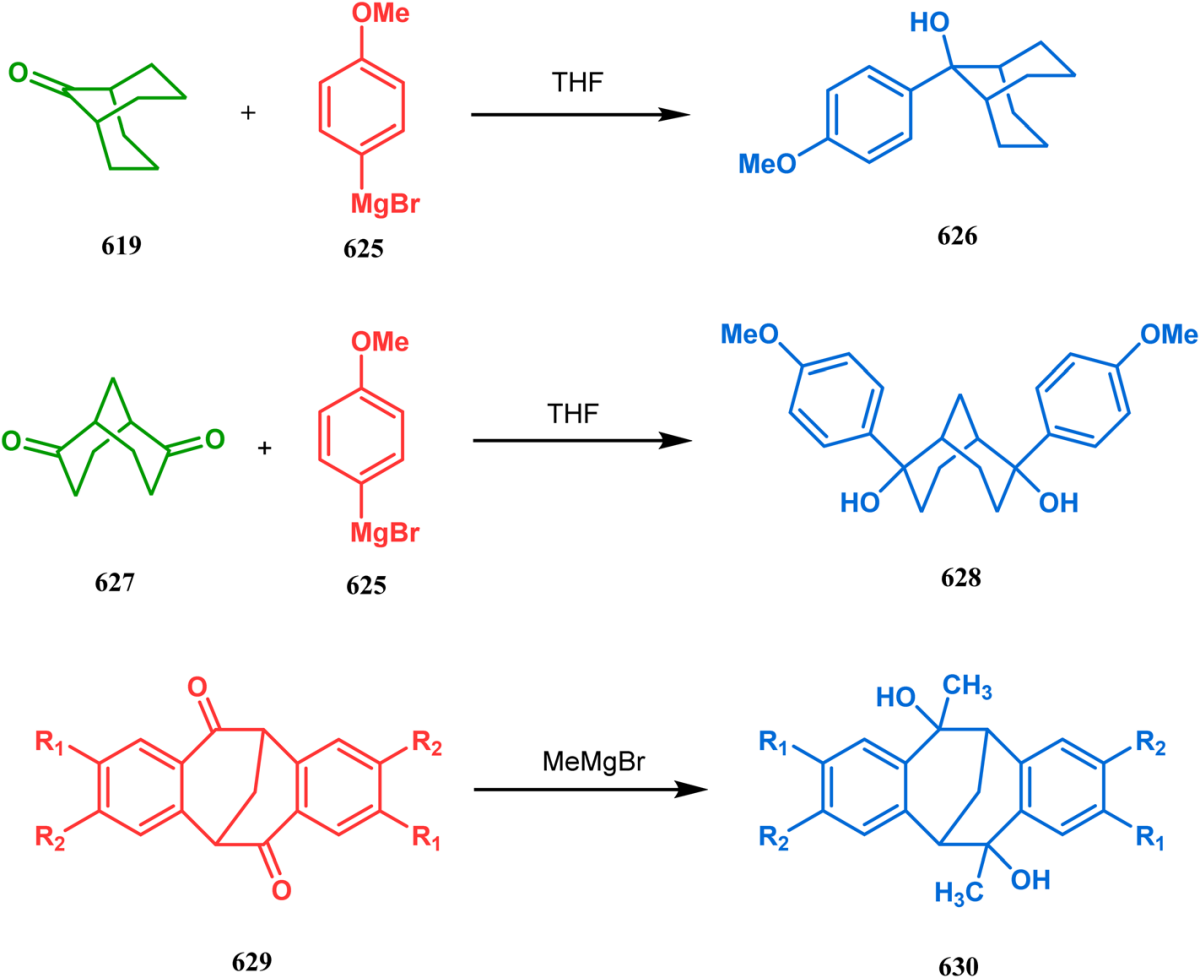
Synthesis of various saturated analogues of bicyclo[3.3.1]nonane by the condensation of Grignard compounds with the carbonyl group.

A similar alkene 623 was synthesized by the Suzuki coupling reaction of 622, which was obtained from ketone 619 with a yield of 61% (Scheme 139). The corresponding saturated analogue (624) could also be obtained from 619 by treating it with phenol in *n*-BuSH (cat.)/HCl in 67% yield (Scheme 140).^304,306^ The synthesis of similar entities (626) from ketone 619 was also accomplished by the condensation of Grignard compounds (625) with the carbonyl group, and the yield was 84%. The diketones 627 and 629 could also be exposed to an identical reaction condition to produce corresponding diols 628 (yield 78%) and 630 (yield 83–87%) (Scheme 141).^307–309^

**Scheme 142:**
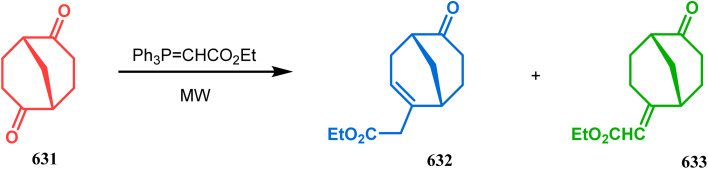
Olefination of dione *via* selective Wittig reaction.

Another type of condensation reaction with the carbonyl group involves the olefination of 631. Selective Wittig reaction in one of the carbonyl groups of 631 produced the olefin 632 exclusively at 230 °C under microwave irradiation. However, the same reaction at lower temperature (190 °C) yielded the inseparable mixture (*E* and *Z*) of the exocyclic product 633 (Scheme 142).^310^ Similar *syn* and *anti* isomers of bicyclo[3.3.1]nonane-based dioximes were also synthesized and reported in the last century by several groups.^311,312^

**Scheme 143:**
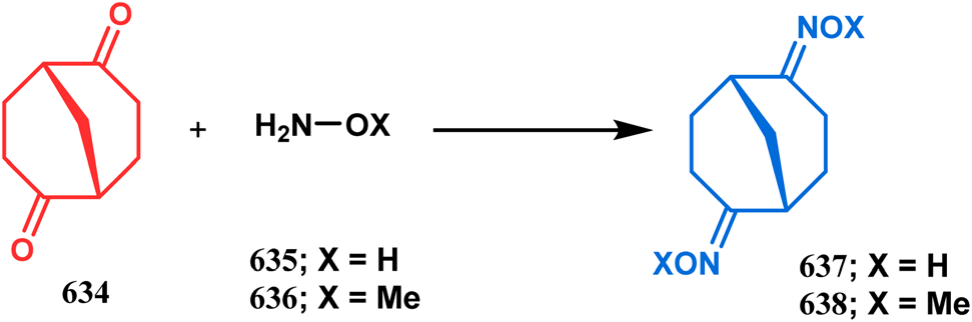
Synthesis of bicyclo[3.3.1]nonane-based dioximes through the condensation of diketone with hydroxylamine.

**Scheme 144:**
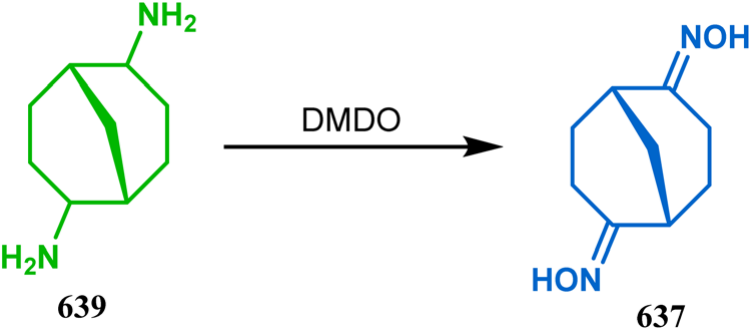
Synthesis of bicyclo[3.3.1]nonane-based dioximes from diamine by DMDO oxidation.

Their strategy involved the simple condensation of diketone 634 with hydroxyl amines (635 and 637) to give dioximes 637 and 638 (Scheme 143).^311^ However, 637 could also be obtained by DMDO-oxidation (dimethyldioxirane) of diamine 639 (Scheme 144).^312,313^ Grosu and coworkers studied the capability of these entities to form supramolecular aggregates and from their single crystal X-ray crystallographic studies, it was found that the syn isomer of 637 forms a supramolecular wheel (*via* six hydrogen bondings), and the corresponding *anti* isomer leads to a cyclic dimer *via* four hydrogen bonds.^314^

**Scheme 145:**
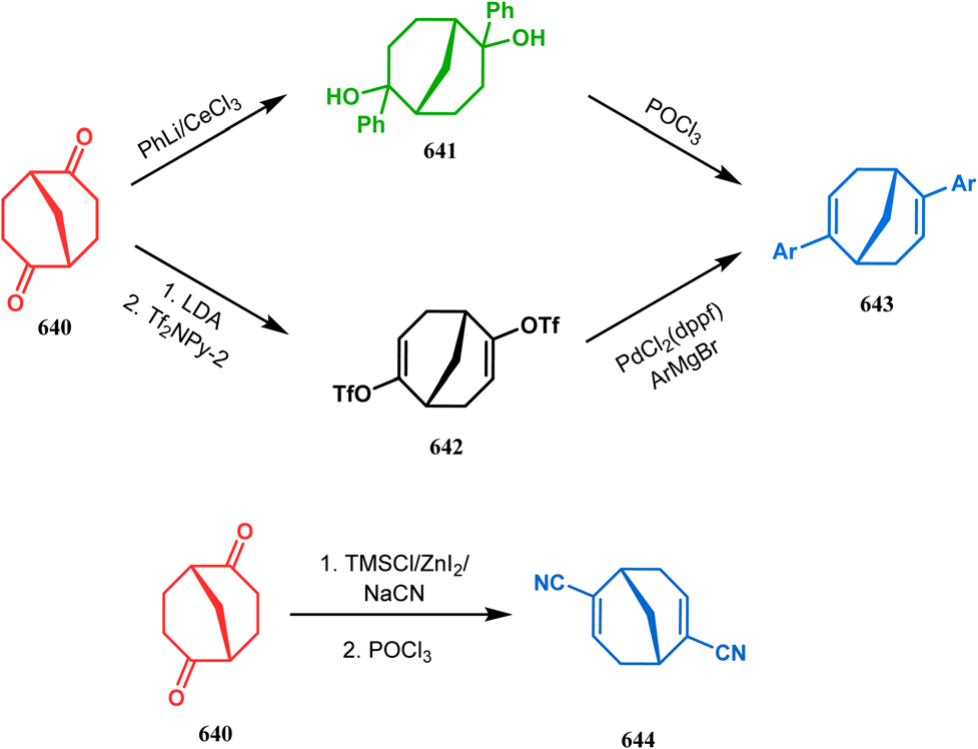
(a) Synthesis of bicyclo[3.3.1]nonane-based chiral diene ligands (b) synthesis of dicyanobicyclo[3.3.1]nonane.

During the synthesis of novel chiral diene ligands, Hayashi and coworkers described the synthesis of 643, a chiral ligand based on the bicyclo[3.3.1]nonane framework. Starting from dione 640, a couple of phenylation reactions produced the diol 641, which yielded the diene 643 on dehydration in 76–97% yield (Scheme 145a).

An alternative route toward this goal proceeded through triflate 642, followed by Pd-catalyzed arylation. This diene was then successfully employed as a chiral ligand in the Suzuki reaction and the addition of aryl/alkenyl boronic acids in α,β-unsaturated ketones.^315–317^ To synthesize differently functionalized bicyclo[3.3.1]nonane frameworks, the dione 640 is widely employed. For example, Quast and coworkers described a careful cyanation on dione 640 to achieve the dicyanobicyclo[3.3.1]nonane 644 (Scheme 145b).^318^

**Scheme 146:**
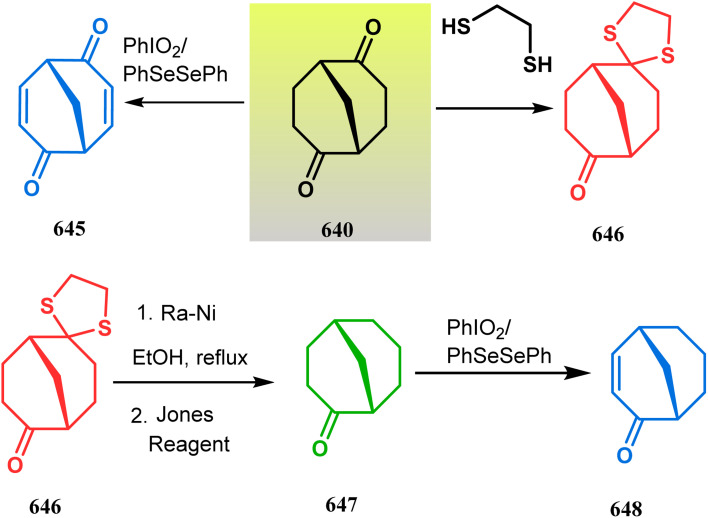
Synthesis of mono-enone and bis-enone analogues of bicyclo[3.3.1]nonane.

Warnmark's group studied the crystal structure of 644 and found the presence of weak hydrogen bonding among the bicyclic units, thereby forming a 2D net-like crystal structure.^319^ Dione 640 is also used to synthesize its bis-enone (645) and mono-enone analogues (648), as reported by Butkus and coworkers (Scheme 146).^320^

#### 4.4.2. Substitution at the α-position of the carbonyl group

**Scheme 147:**
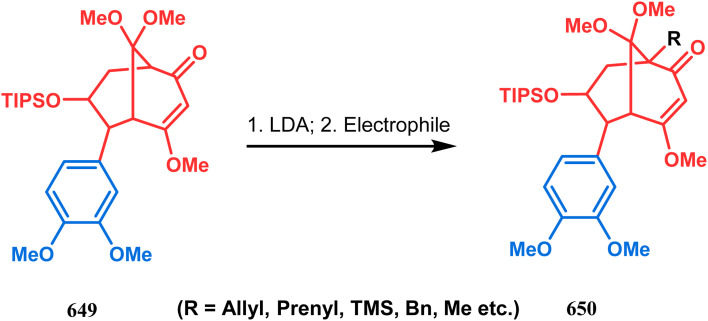
Metallation at the bridgehead position during the synthesis of the bicyclic core of garsubellin A.

Substitution at the bridgehead position of bridged ketones is always a difficult process as it disobeys Bredt's rule. However, Simpkins and coworkers utilized some efficient lithium amide bases (655a,b) to facilitate metallation at the bridgehead position. During the synthesis of the bicyclic core of garsubellin A, they successfully utilized a rigioselective lithiation procedure to achieve 650 from 649 using LDA as the base (Scheme 147).^321^

**Scheme 148:**
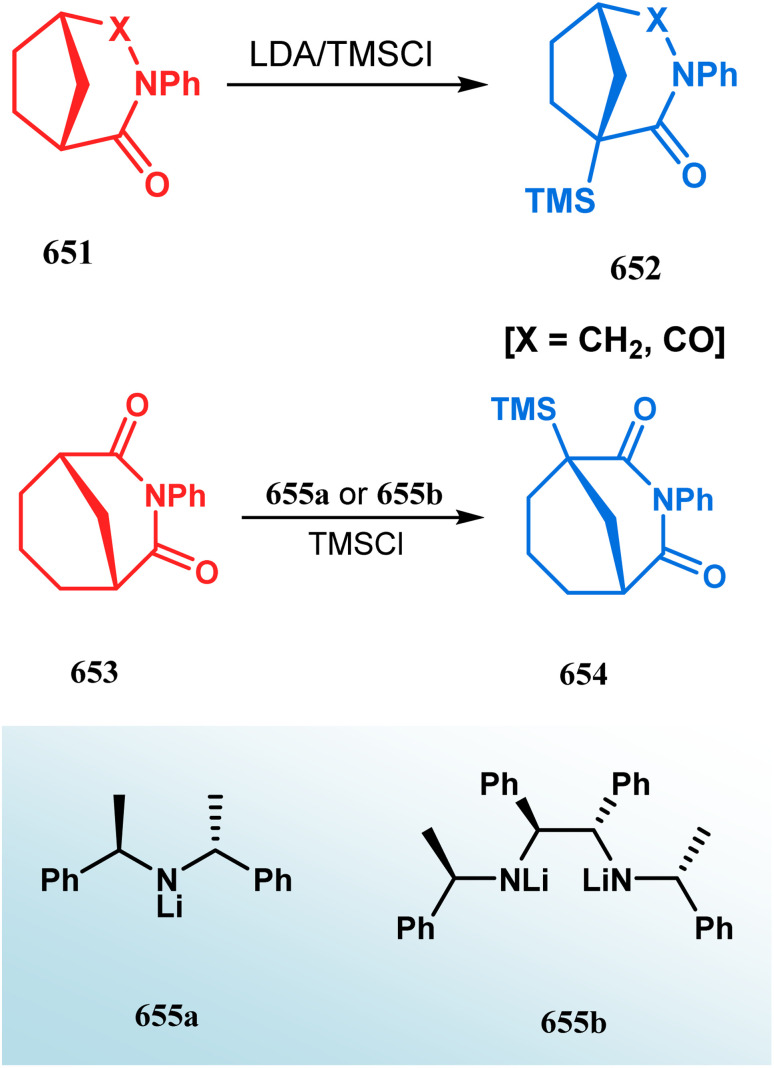
(a) Synthesis of bridgehead-substituted product (b) synthesis of bridgehead-substituted product.

A similar LDA-promoted substitution reaction on analogous bicyclic systems demonstrated different results. It showed that although LDA-mediated TMS-substitution of bicycles 651 produces the corresponding bridgehead substituted products (652) in good yields (Scheme 148a), the same reaction with bicyclo[3.3.1]nonane 653 gives low product yield. Thus, amide bases 655a,b were employed to produce product 654 in high yields (Scheme 148b).^322^

#### 4.4.3. Amide formation

**Scheme 149:**
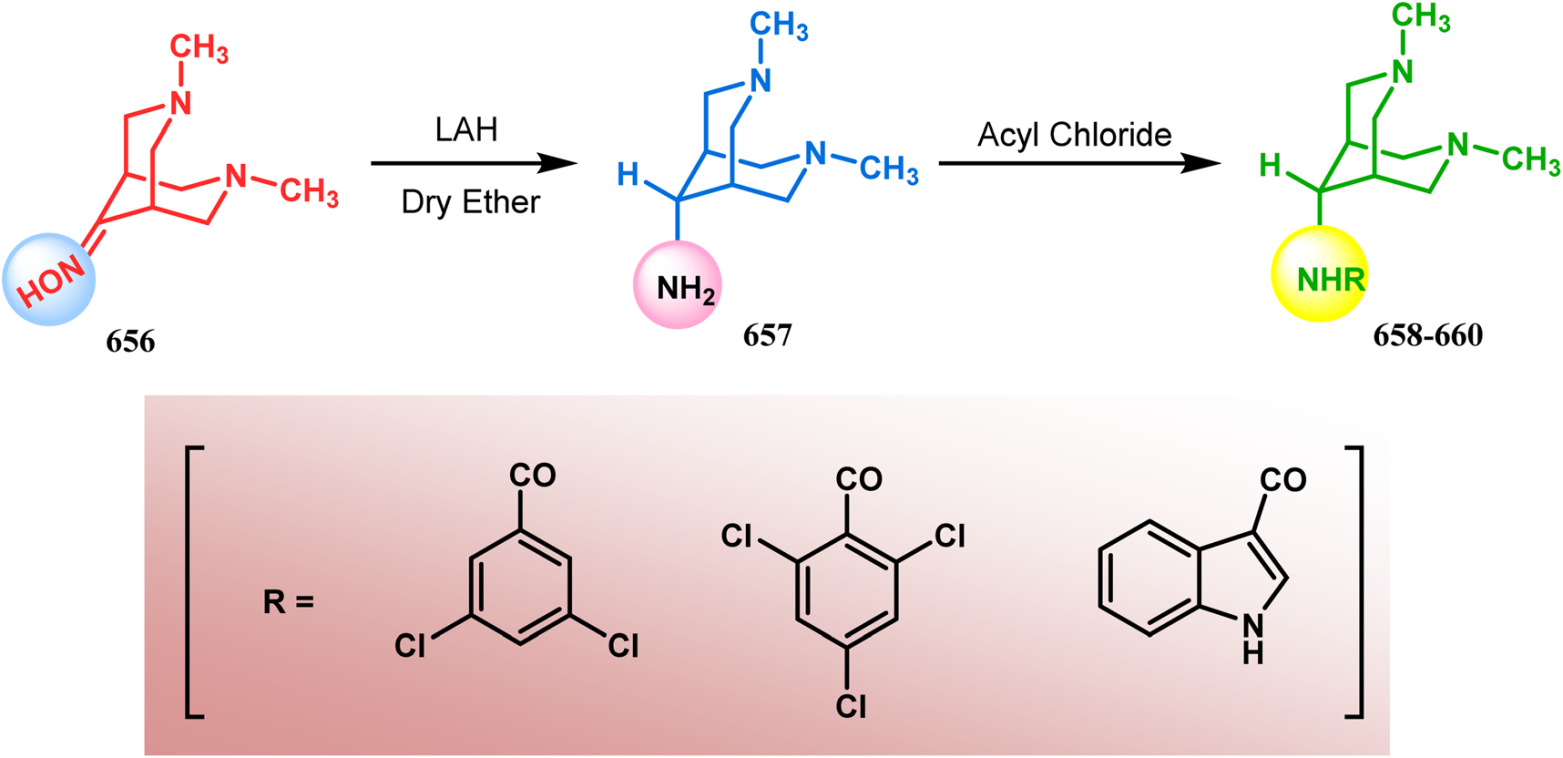
Formation of amides from the amines of bicyclo[3.3.1]nonanes.

Amide formation from the amines of bicyclo[3.3.1]nonanes is also well documented in the literature. For example, to study the structural and conformational properties, some amides based on bicyclo[3.3.1]nonane framework were described by Galvez and coworkers. The required amine (657) for this purpose was constructed by reducing the corresponding oxime (656) using lithium aluminium hydride. Once the amine is formed, corresponding amides (658–660) were synthesized using a diverse range of acyl chlorides in DCM (Scheme 149).^323^

**Scheme 150:**
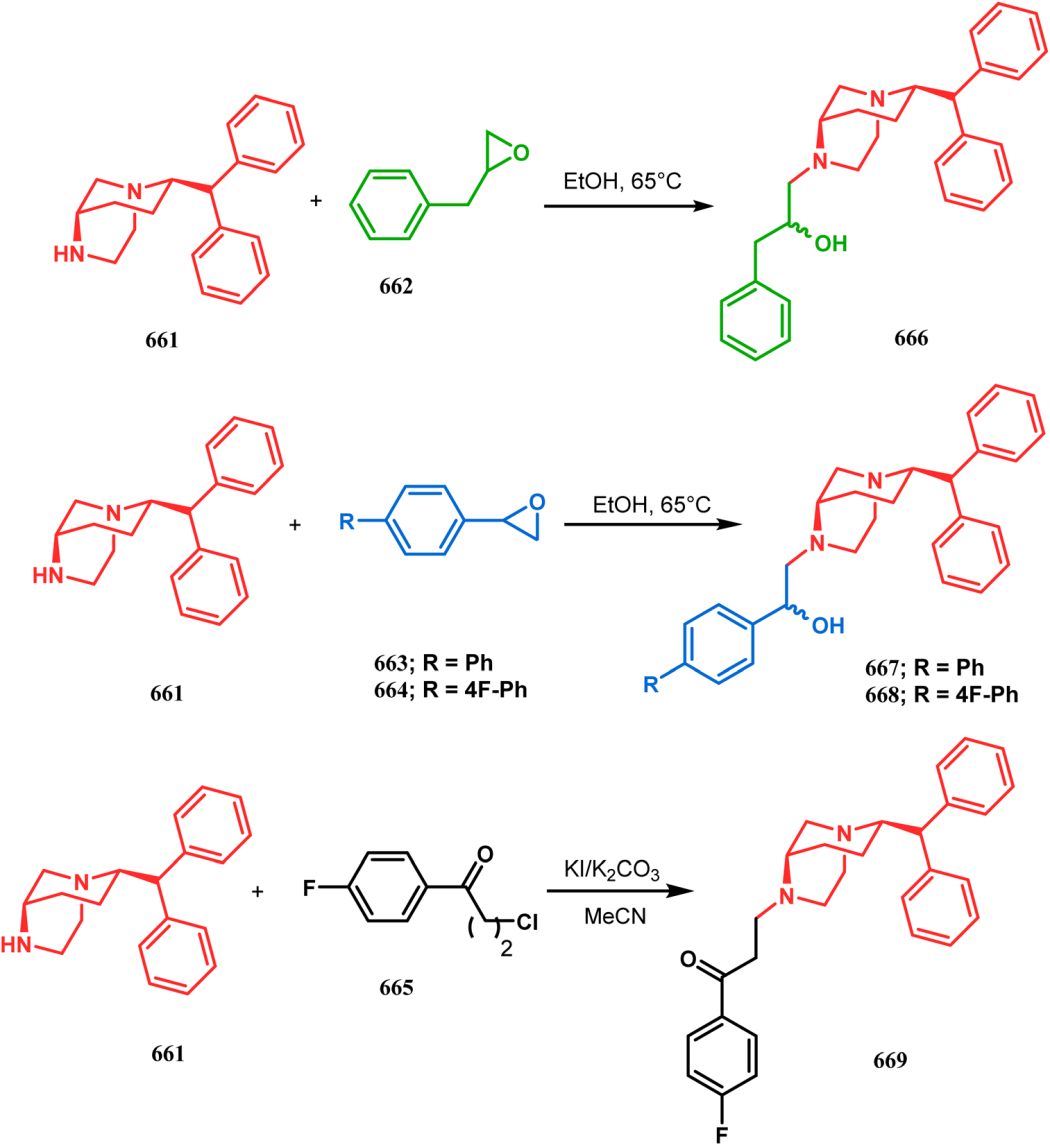
Synthesis of amides from the reactions of optically-active amine with differently substituted 2,3-epoxypropyl entities and 3-chloro-4′-fluoro-propiophenone.

Similar amide formation reactions were also reported by Dutta and coworkers. They first synthesized the optically active amine (661) following their earlier reported procedure,^324^ which was then separately treated with differently substituted 2,3-epoxypropyl entities (662–664), 3-chloro-4′-fluoro-propiophenone (665), and the corresponding amides (666–669) were isolated in good yields (Scheme 150). These amide linkages were then further modified, and their interaction with dopamine, serotonin, and norepinephrine transporters was investigated.

**Scheme 151:**
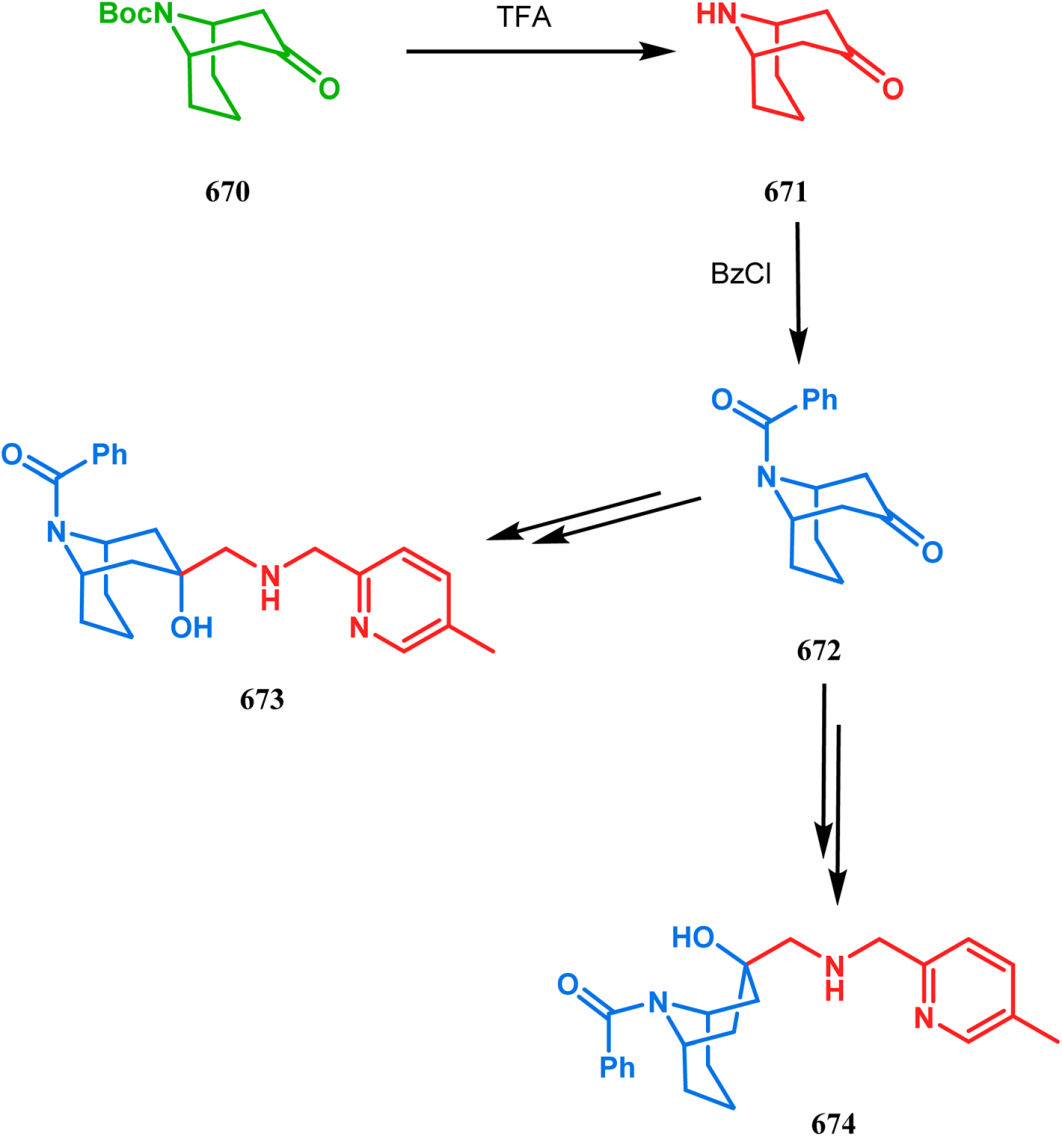
Synthesis of bicyclo[3.3.1]nonane-based pyridylmethylamines.

**Scheme 152:**
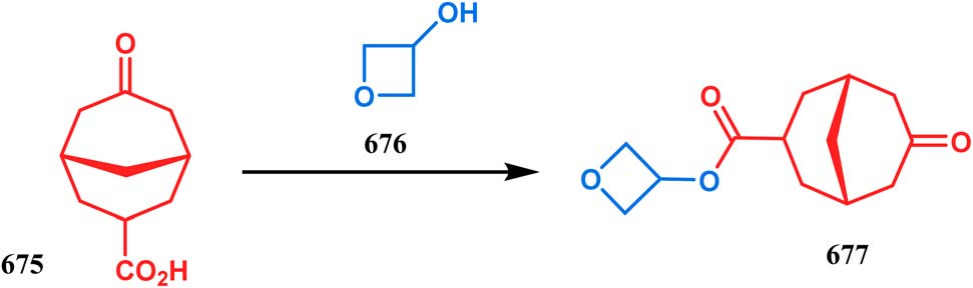
Synthesis of bicyclo[3.3.1]nonane-based ester.

Another report on bicyclo[3.3.1]nonane-based amide formation reaction was published by Gmeiner and coworkers. While searching for selective 5-HT_1A_ superagonists, they synthesized some novel bicyclo[3.3.1]nonane-based pyridylmethylamines (673, 674) from 670 (Scheme 151). The synthesis involves an initial boc-deprotection, followed by amide bond formation with benzoyl chloride to give amide 672, which was then sequentially converted into targeted pyridylmethylamines (673, 674).^325^ Apart from these amide formation reaction of bicyclo[3.3.1]nonane-amines, the ester formation of the corresponding carboxylic acids is also well documented. Kiryukhin and coworkers described one such example, where carboxylic acid 675 was reacted with 3-hydroxyoxetane 676 and formed the ester 677 (Scheme 152).^326^

#### 4.4.4. Metal complexation

**Scheme 153:**
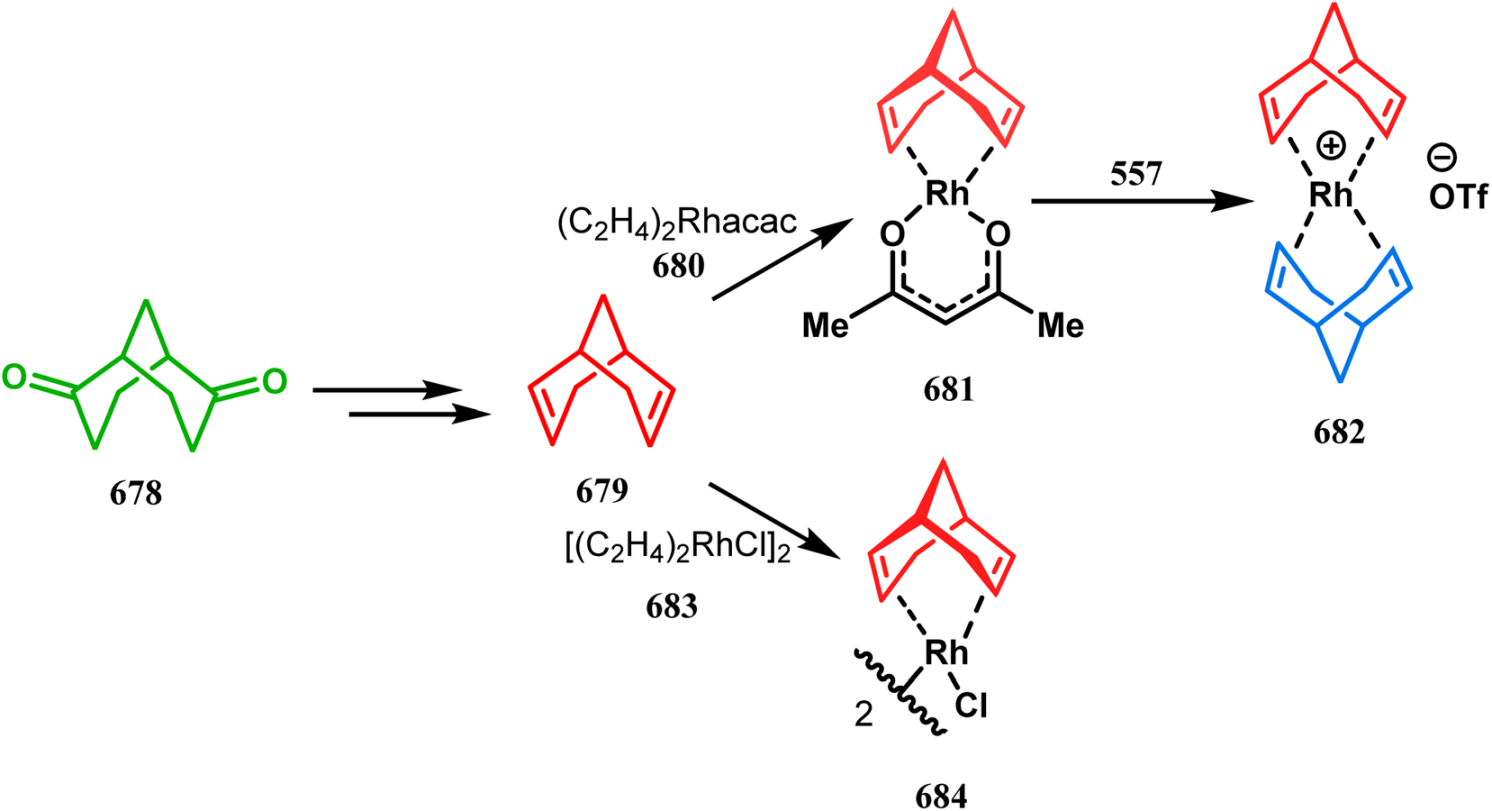
Synthesis of rhodium complex.

The application of appropriately substituted bicyclo[3.3.1]nonane derivatives for the complexation reaction with rhodium, titanium, and zirconium chlorides or acetates is also reported by several research groups. Brown and coworkers described one such unique example a few years ago. They employed the enantiopure diene 679, obtained from diketone 678, to complex it with the diethylene complex of rhodium acetate (680) and yielded a new rhodium complex (681). Further exposure of 681 with diene 679 for another one hour in TMSOTf produced the sandwiched complex 682. A similar reaction of 679 with the dirhodium complex 683 also gave another complex 684 (Scheme 153).^327^

**Scheme 154:**
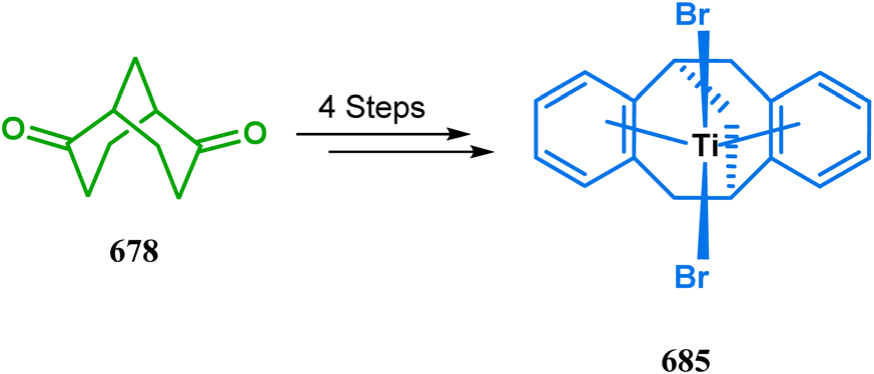
Synthesis of titanium complex.

**Scheme 155:**
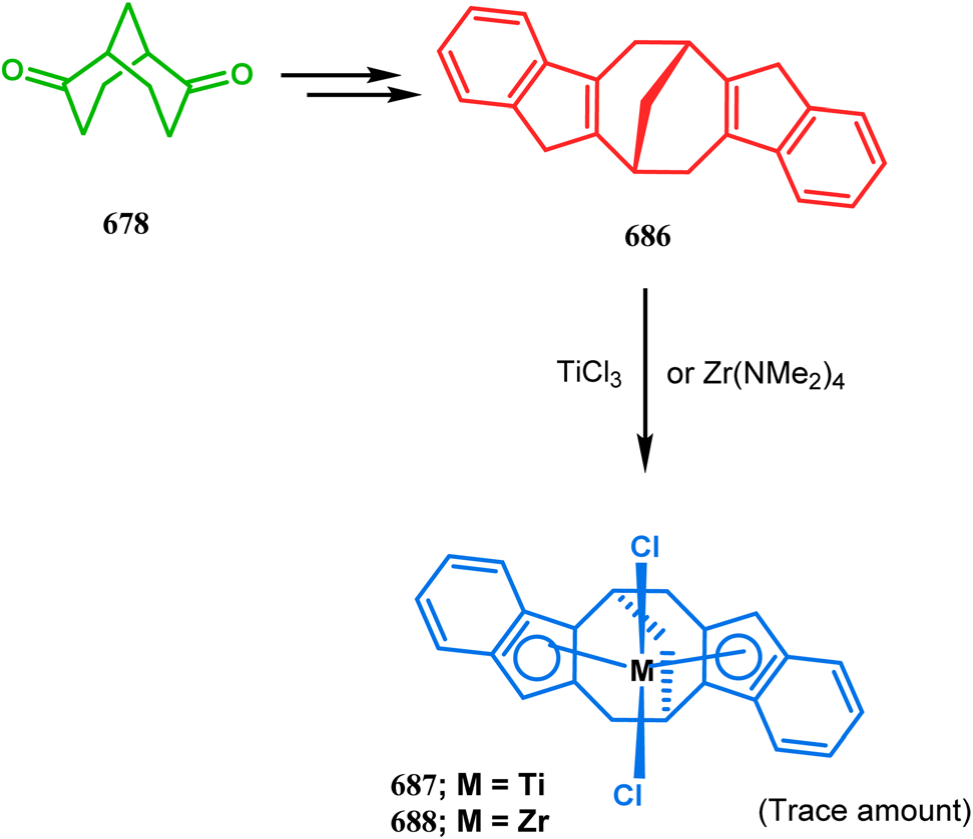
Synthesis of titanium and zirconium complex.

The synthesis of a similar titanium complex was also reported in the literature by Grossman and coworkers. Starting from bicyclo[3.3.1]nonane-2,6-dione (678), this *C*_2_-symmetric, chiral ansa–titanocene complex (685) was synthesized in four steps (Scheme 154).^328^ Halterman and coworkers also attempted the synthesis of similar titanium and zirconium complexes. Their journey also began from dione 678, and the cleft-shaped bis(indene) 686 was constructed for its metallation reaction. Although the crude product obtained from the reaction of 686 with TiCl_3_ or ZrCl_4_ showed new signals in the ^1^HNMR spectrum, the complexes (687, 688) could not be isolated (Scheme 155).

#### 4.4.5. Other reactions

##### 4.4.5.1. Aldol condensation

**Scheme 156:**
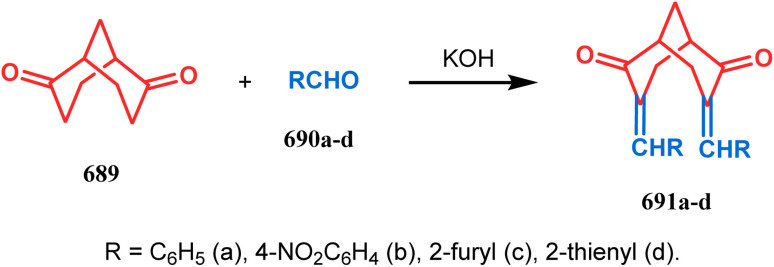
Synthesis of bicyclo[3.3.1]nonane-based α,β-unsaturated diene-dione *via* aldol condensation.

The aldol condensation of bicyclo[3.3.1]nonanones is also reported in the literature. In one such example, Moiseev and coworkers employed KOH to condense dione 689 with aldehydes 690, which yielded the α,β-unsaturated diene-dione 691 in high yields (Scheme 156).^329^ Interestingly, no monocondensation product was formed during the reaction. Even if the reaction was carried out employing 689 and 690 in 1 : 1 mole ratio, only the double-condensation product was produced.

##### 4.4.5.2 Michael addition reaction

**Scheme 157:**
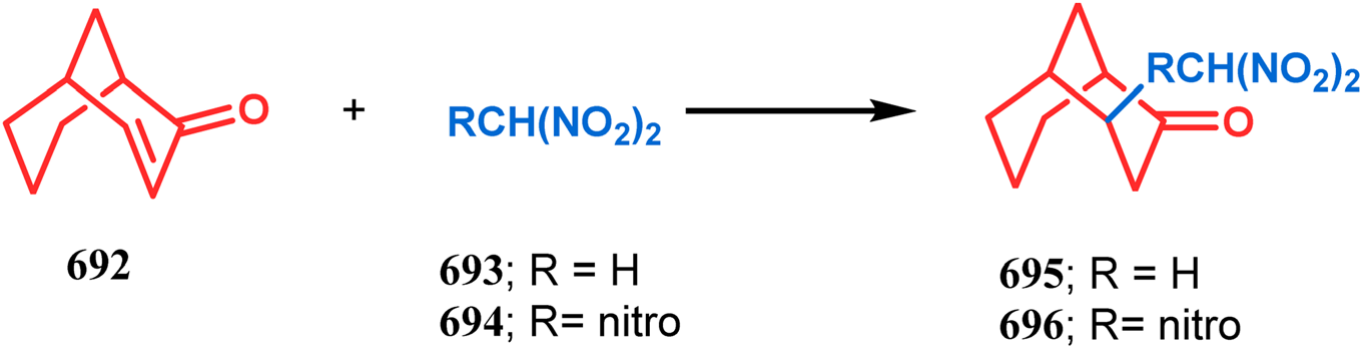
Synthesis of bicyclo[3.3.1]nonane-containing nitroalkanes.

**Scheme 158:**
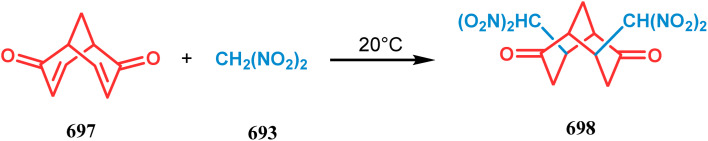
Synthesis of bicyclo[3.3.1]nonane-containing nitroalkanes.

**Scheme 159:**
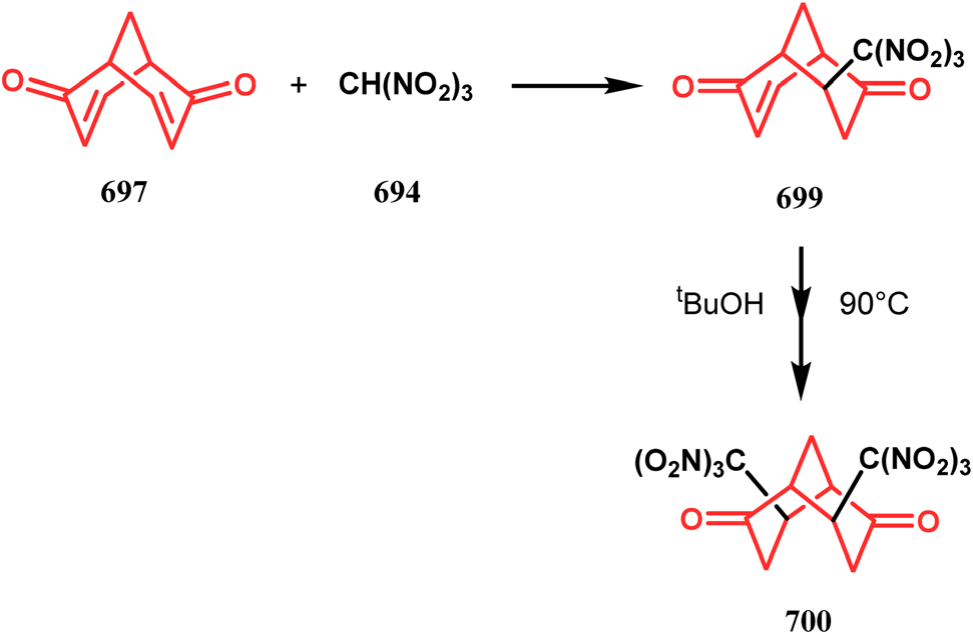
Synthesis of bicyclo[3.3.1]nonane-containing nitroalkanes from the reaction of diene-dione with nitroform.

The michael addition reactions of α,β-unsaturated ketones in bicyclo[3.3.1]nonane series is also well-documented in the literature. Smirnov and coworkers investigated the addition of diethylmalonate and mononitroalkanes several years ago.^330^ Following their report, Zavarzin's group demonstrated a similar approach employing polynitroalkanes.^331^ As expected, the more reactive dinitro and trinitroalkane Michael donors (693, 694) reacts with enone 692 at a much faster rate to form bicyclo[3.3.1]nonane-containing nitroalkanes (695, 696) in high yields (Scheme 157). Although the first reaction required triethylamine as the catalyst, the latter did not. However, a similar reaction of nitroform (694) with diene-dione 697 allows only one molecule of nitroform to get condensed to form 699 (Scheme 159), whereas under identical conditions, two molecules of 693 react with 697 to form 698 (Scheme 158). Increased steric crowding in the former case may be responsible for this observation. It was also found that much higher temperature (90 °C) and a high boiling solvent (Bu^*t*^OH) is required to construct the double condensed product (700) of nitroform and diene-dione 697 (Scheme 159).^330,331^

##### 4.4.5.3 Amination reaction

**Scheme 160:**
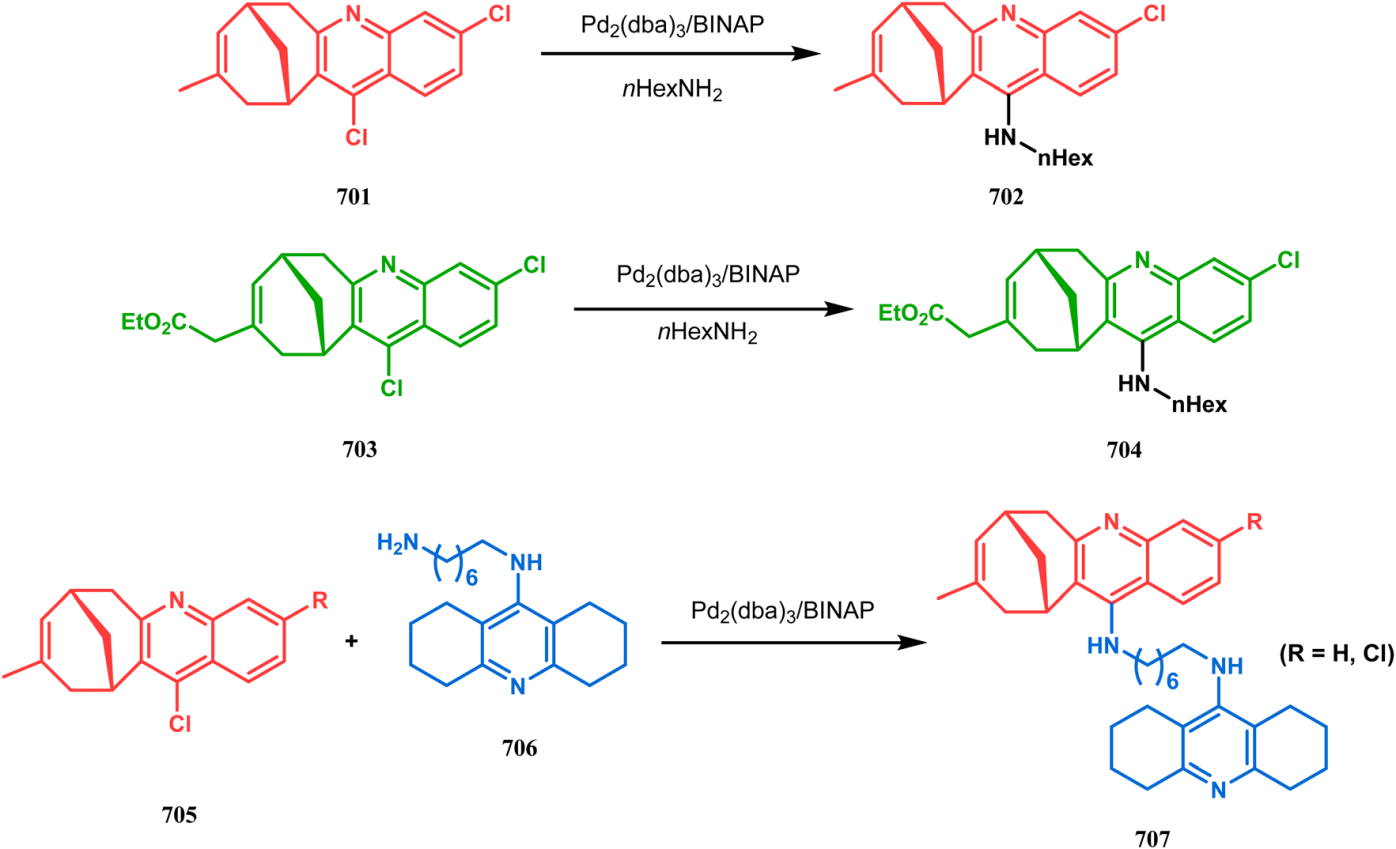
Synthesis of aminoquinolines by palladium-catalyzed amination reactions of bicyclo[3.3.1]nonane.

Palladium-catalyzed amination reactions in the bicyclo[3.3.1]nonane series is widely investigated by Renard and coworkers. Since huprine-like aminoquinolines exhibit interesting pharmacological properties, studies on the synthesis of similar analogues have received much attention in recent years. Renard's team, through a thorough screening of solvents, bases, ligands, and Pd-catalysts, found that haloquinolino-bicyclo[3.3.1]nonanes 701 could be converted into corresponding aminoquinolines 702 using Pd_2_(dba)_3_ as the catalyst, BINAP as the ligand, cesium carbonate as the base, and dioxane as the solvent with high yields. Bicyclo[3.3.1]nonanes 703 and 705 are also well-tolerated under similar reaction conditions, and the corresponding aminoquinolines 704 and 707 were obtained in good yields (Scheme 160).^332^

**Scheme 161:**
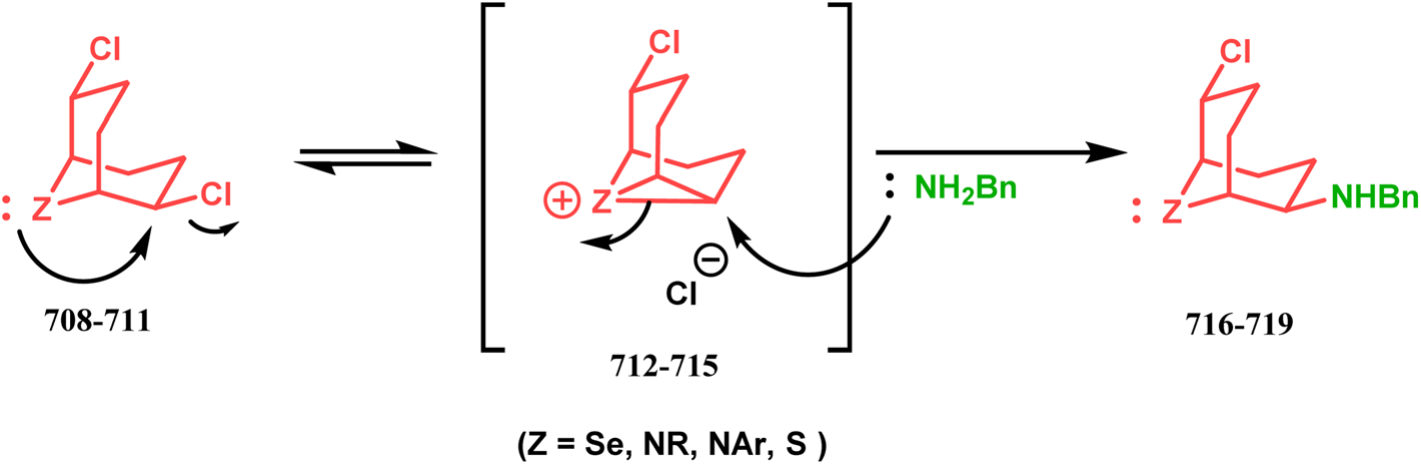
Synthesis of bis(amino)-bicyclo[3.3.1]nonanes.

Another amination reaction in bicyclo[3.3.1]nonane series was described by Finn and coworkers. They demonstrated the amination of thia-, aza-, and selena-bicyclo[3.3.1]nonane dichlorides (708–711), which proceeds through an internal anchimeric assistance from the ring nitrogen, sulphur, or selenium center. Thus, when dichlorides 708–711 were reacted with benzylamine, bis(amino)-bicyclo[3.3.1]nonanes 716–719 were obtained through a cationic intermediate (712–715) in high yields. A similar reaction with other nuclophiles also proceeds in a similar pathway to produce corresponding substitution products (Scheme 161).^163^

##### 4.4.5.5 Thiolation

**Scheme 162:**
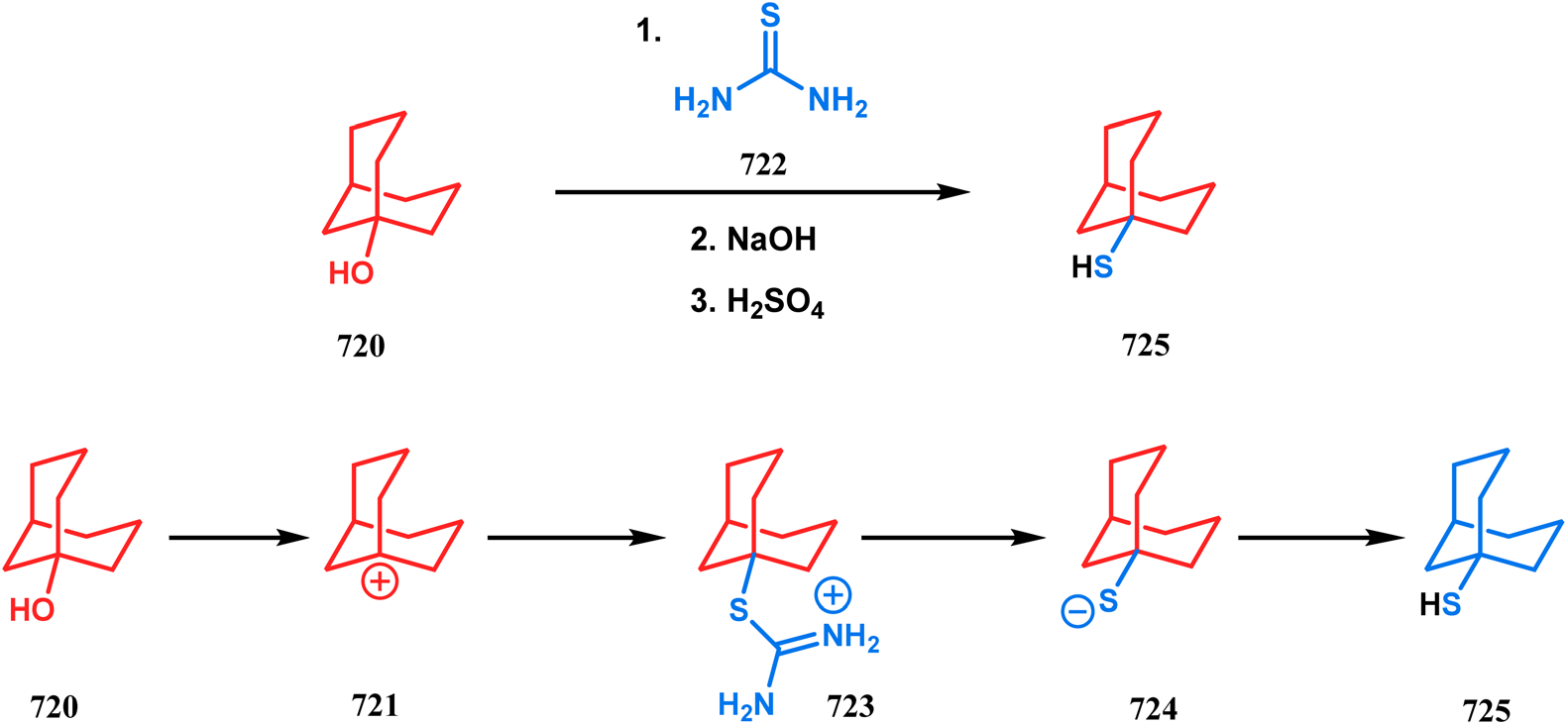
Synthesis of bicyclo[3.3.1]nonane-based thiol by the thiourea-mediated thiolation of bicyclo[3.3.1]nonalol.

Thiolated organic frameworks get easily attached with metal surfaces to form self-assembled monolayers (SAMs), which are used in molecular electronics. Fokin and Schreiner's group contributed substantially in this regard. Their studies involved the thiourea-mediated thiolation of bicyclo[3.3.1]nonalol (720) to form the corresponding thiol 725. The reaction proceeds with initial carbocation (721) formation from 720, followed by the nucleophilic addititon of thiourea (722) to form intermediate 723. The elimination of carbonate and ammonia then produced sulfide 724. The immediate acidification of 724 yielded the desired thiol 725 in excellent yields (Scheme 162).^333^

##### 4.4.5.6 Bicyclo[3.3.1]nonane scaffold and its anticancer activity

**Fig. 1:**
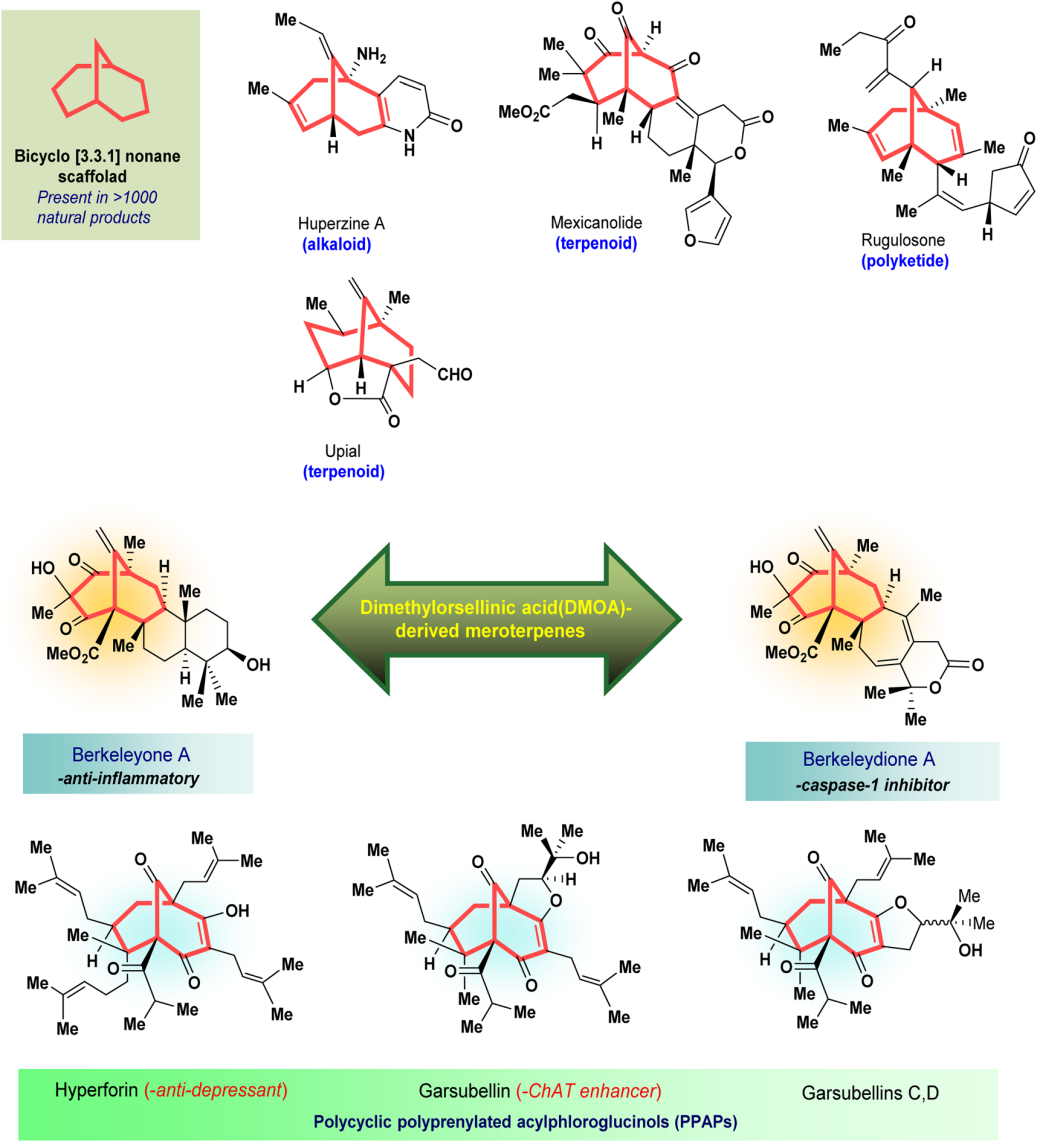
A family of meroterpenes as an abundant source of bicyclo[3.3.1]nonane.

Of late, the peril of cancer is monopolizing the whole world as a salient cause of death due to the unrestrained and abnormal proliferation of cells. Natural products obtained from medicinal plants acquire remarkable potential as dexterous drugs for a number of diseases and thereby hold a great source for the discovery of new drugs. Being present in more than 1000 natural products capable of curing several neurodegenerative diseases as well as parasitic and bacterial infections, the most important bioactive bicyclo[3.3.1]nonane architecture renders it a very attractive moiety to exhibit cancer inhibiting potential. Therefore, bicyclo[3.3.1]nonane-containing natural products are being highly applauded by researchers to be developed as chemotherapeutics for the treatment of specific cancers. It has been seen that the bicyclo[3.3.1]nonane scaffold is present as the core moiety in many biologically important alkaloids, polyketides, and terpenoids. For example, it plays a key role in huperzine (neuroprotective alkaloid), rugulosone (antimalarial polyketide), mexicanolide (cytotoxic limonoid), and upial (sesquiterpene). Moreover, the mixture of terpenoids and polyketides constitutes one kind of natural products known as meroterpenes, which serve as an affluent source of bicyclo[3.3.1]nonane. The derivatives of 3,5-dimethylorsellinic acid (DMOA) and acylphloroglucinol (APs) are very efficient in displaying various biological activities. Keleyone A and berkeleydione are two such crucial derivatives of DMOA that exhibit antiinflammatory and caspase-1 inhibitory potency, respectively. The neuroactive hyperforin and garsubellins is two important polycyclic polyprenylated acylphoroglucinol (PPAs) possessing bicyclo[3.3.1]nonane scaffolds. In practice, PPAs are very competent to bring on different biological activities; hence, PPAs are an important structure in drug discovery for infectious diseases, neuroscience, as well as oncology (Fig. 1).^334^

Hyperforin was the first bicyclic polyprenylated acylphloroglucinol (BPAP) that was isolated in 1971 from a medicinal plant named St. John's wort to treat a plethora of diseases in ancient Greece. As hyperforin was highly admired due to its noticeable antidepressant activity, scientists focused their attention into the discovery and isolation of various natural BPAPs from the plants of Clusiaceae and Hypericaceae family, where all the natural BPAPs are seen to be very efficient to display various biological activities. Keleyone A and berkeleydione are two such crucial derivatives of DMOA that exhibit antiinflammatory and caspase-1 inhibitory potency, respectively. The neuroactive hyperforin and garsubellins are two important polycyclic polyprenylated acylphoroglucinol (PPAs) possessing the bicyclo[3.3.1]nonane scaffold. In practice, PPAs are very competent to bring on different biological activities and are commonly classified as a highly oxygenated acylphloroglucinol with isoprenyl or geranyl side chains. The biosynthesis of BPAPs is accomplished by condensing three malonyl-CoA units and one acyl-CoA unit to construct the polyketide moiety, which is subsequently converted into acylphloroglucinol through Dieckmann cyclization. After that, the prenylation of this moiety produces monocyclic polyprenylated acylphloroglucinols (MPAPs), which in turn undergoes several cyclizations to form the varied frameworks of BPAPs and their analogues. Inspite of having astounding anticancer, antidepressant, antiinflammatory, antioxidant, and antimicrobial activities of all the BPAPs, scientists are facing challenges to find out lead compounds among all the BPAPs. The most important and extensively studied biological function of BPAPs is its incredible anticancer activity. The most promising anticancer activity is observed for hyperfoin, nemorosone, guttiferones, oblongifolin C, clusianone, and garcinol. Most of these BPAPs have been found to reveal their anticancer potential through the induction of cancer cell death or by the inhibition of cancer cell survival, adhesion, proliferation, invasion, angiogenesis, as well as metastasis, triggering some signaling pathways.^335^
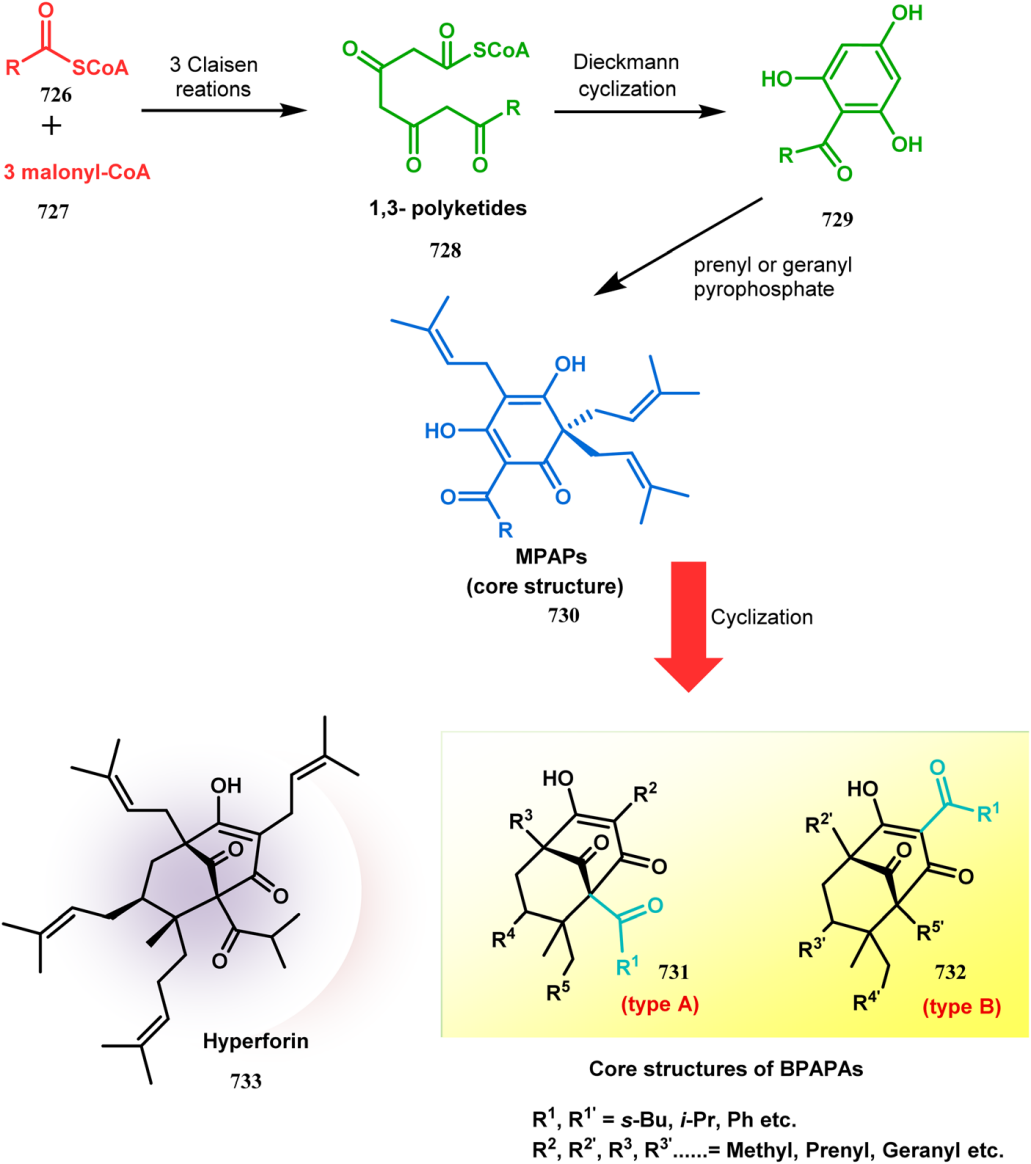


**Fig. 2:**
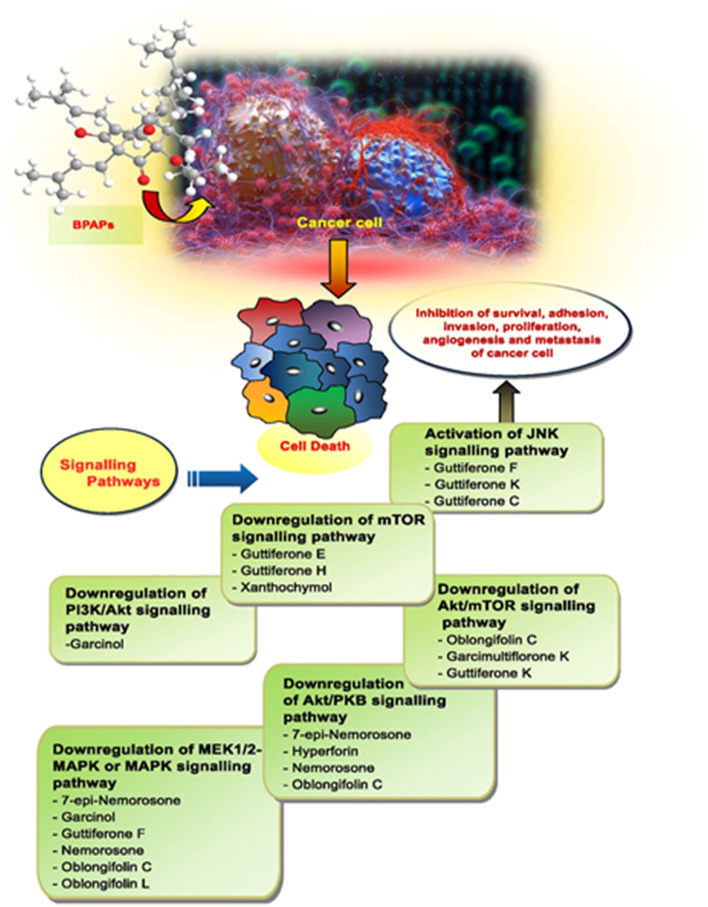
Different anticancer signaling pathways induced by various BPAPs.

It has been studied that hyperforin conveys its anticancer activities mostly toward leukaemia, colorectal carcinoma, nonsmall cell lung cancer, and muscle-invasive bladder cancer as a result of provoking mitochondrial intrinsic/extrinsic pathways through the release of cytochrome C and the reduction of mitochondrial membrane potential (MMP). Hence, it is very much efficient to endorse the upregulation of proapoptotic proteins (Bak, Bad, procaspase-3/9) and the suppression of antiapoptotic proteins such as Mcl-1, XIAP, and C-FLIP. Hyperforin is best suited for the treatment of acute myeloid leukaemia (AML) and chronic lymphoid leukaemia (CLL), triggering apoptosis through the modulation of the PI3K/Akt signaling pathway, where it destroys the kinase activity of serine/threonine protein kinase B (PKB)/Akt1 and thereby encourages the activation of proapoptotic Bad and procaspases-9/-3. In case of CCL, hyperfoin is seen to upregulate Noxa, which in turn stimulates the emergence of proapototic Bak protein from Mcl-1 and thus helps the apoptogenic factors to be released from the mitochondria, leading to cell death. In line with this, hyperforin downregulates the NF-κβ P65 and thereby restrains the expression of antiapoptotic and tumor growth proteins. Also, it facilitates the cell cycle arrest at the G1 phase by hindering the formation of cyclin D1. Moreover, hyperforin is adept at inducing apoptosis, creating endoplasmic reticulum (ER) stress and damaging DNA through the increase in ROS level and calcium signaling in the cytoplasm of cancer cells. It has been detected that vascular endothelial growth factor (VEGF) and matrix metalloproteinases-9 (MMP-9) protein level are suppressed on the treatment of hypoforin along with the suppression of *P*-glycoprotein expression, which indicates the high anticancer proficiency of hyperforin. It can be mentioned that hyperforin can also be ascribed to have antiangiogenic, antigenotoxic, and anticlastogenic properties (Fig. 2).^336^

**Fig. 3:**
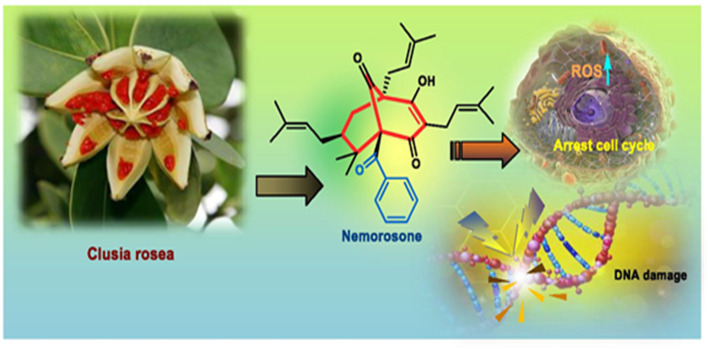
*Nemorosone* isolated from *Clusia rosea* and its anticancer activity.

Nemorosone is also a potent anticancer compound containing the bicylo[3.3.1]nonane moiety. It is isolated from the flora resin of *Clusia rosea* plant (Fig. 3). This naturally obtained compound has been found to induce apoptosis by activating the unfolded protein response (UPR) in case of pancreas cancer. Nemorosone is seen to be very active to resist the progression of cell cycle in leukaemia cells after targeting the Akt/PKB signal transducer in association with diminishing the formation of cyclins A, B1, D1, E, as well as c-Myb levels. It is also very potent against neuroblastoma cells by triggering the activity of caspase-3 and then obstructing the kinase activity of ERK1/2 or regulating the Akt/PKB signaling pathway. To prevent oestrogen receptor alpha positive (ERα+) breast cancer, nemorosone may successfully be used as an adjuvant owing to its inhibitory action toward 17-β-estradiol (E2) without any genotoxicity. The mechanistic study reveals that nemorosone can destroy the ERα+ cells by arresting the progression of cell cycle at the G_0_/G_1_ phase by modifying the expression of Akt, ERK1/2, and other genes associated with cell cycle, apoptosis, or hormone receptor. Moreover, nemorosone can act as a chemosensitizer toward the doxorubicin-resistant colon carcinoma cells (LoVo Dox) and can bring on apoptosis through cell cycle arrest with the production of profuse ROS and changing of inner mitochondrial membrane potential (MMP).^337^ The proliferation and then metastasis of human colorectal carcinoma (CRC) cells were found to be inhibited on the treatment of nemorosone as it is capable of diminishing the epithelial–mesenchymal transition (EMT)-related markers in CRC cells.^338^ 7-*epi*-nemorosone, an epimer of nemorosone is also efficient to cause the apoptosis of cancer cells. It has been studied that 7-*epi*-nemorosone can suppress the expression of androgen receptor (AR) and the production of prostate-specific antigen (PSA) (Fig. 3).

Therefore, it can alter the MEK1/2 as well as Akt/PKB transducers in androgen-dependent prostate carcinoma cells (LNCaP) along with the downregulation of cyclins D1/D3 and cyclin-dependent kinase (CDK) 4/6. The anticancer potential of nemorosone against two human colorectal cancer cell lines, HT-29 and LoVo, was tested. The corresponding IC_50_ values have been provided in Table 1.

**Table 1:** IC_50_ values for treating nemorosone against HT-29 and LoVo cells

IC_50_ (μM)		
HT-29		LoVo
24 h	57.1 ± 3.7	64.3 ± 4.7
48 h	33.4 ± 2.8	35.9 ± 9.1
72 h	25.7 ± 3.3	22.8 ± 6.2

**Fig. 4:**
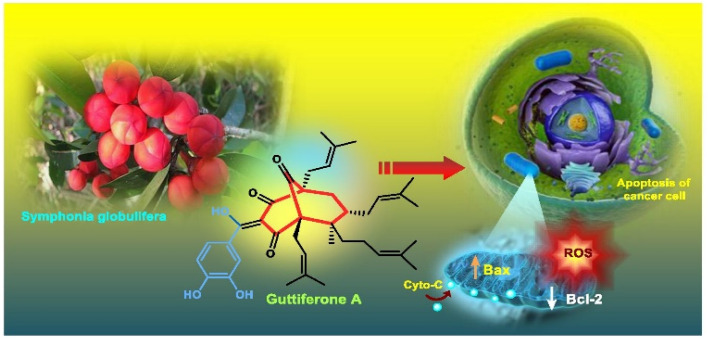
Guttiferone A isolated from *Symphonia globulifera* and its anticancer activity.

The potential anticancer compound, guttiferone A, which possesses the bicyclo[3.3.1]nonane core, was first isolated from the root of *Symphonia globulifera* and recently isolated from *Garcinia livingstonei* and *Garcinia macrophylla*. The remarkable anticancer property of this compound was attracted by the researchers; hence, they were eager to study its mechanisms of action in cancer cells. It has been observed that this compound can act as an inhibitor of serine and cysteine proteases and accelerates the production of ROS, which leads to a decrease in mitochondrial membrane potential (MMP) in MCF-7 breast cancer cells.^339^ Consequently, proapoptotic Bax protein becomes upregulated, whereas antiapoptotic Bcl-2 becomes downregulated on the treatment of guttiferone A. It also conveys cytotoxity toward hepatocellular carcinoma. Its anticancer activity was accounted for ATP depletion, NADPH depletion, decrease in MMP, uncoupling of membrane, Ca^2+^ efflux, cyclosporine A/EGTA-insensitive membrane permeabilization, and ROS accumulation.^340^ The IC_50_ value was found to be 15 μM against the MCF-7 breast cancer cell lines upon treatment of guttiferone A in a dose-dependant manner (Fig. 4).

**Fig. 5:**
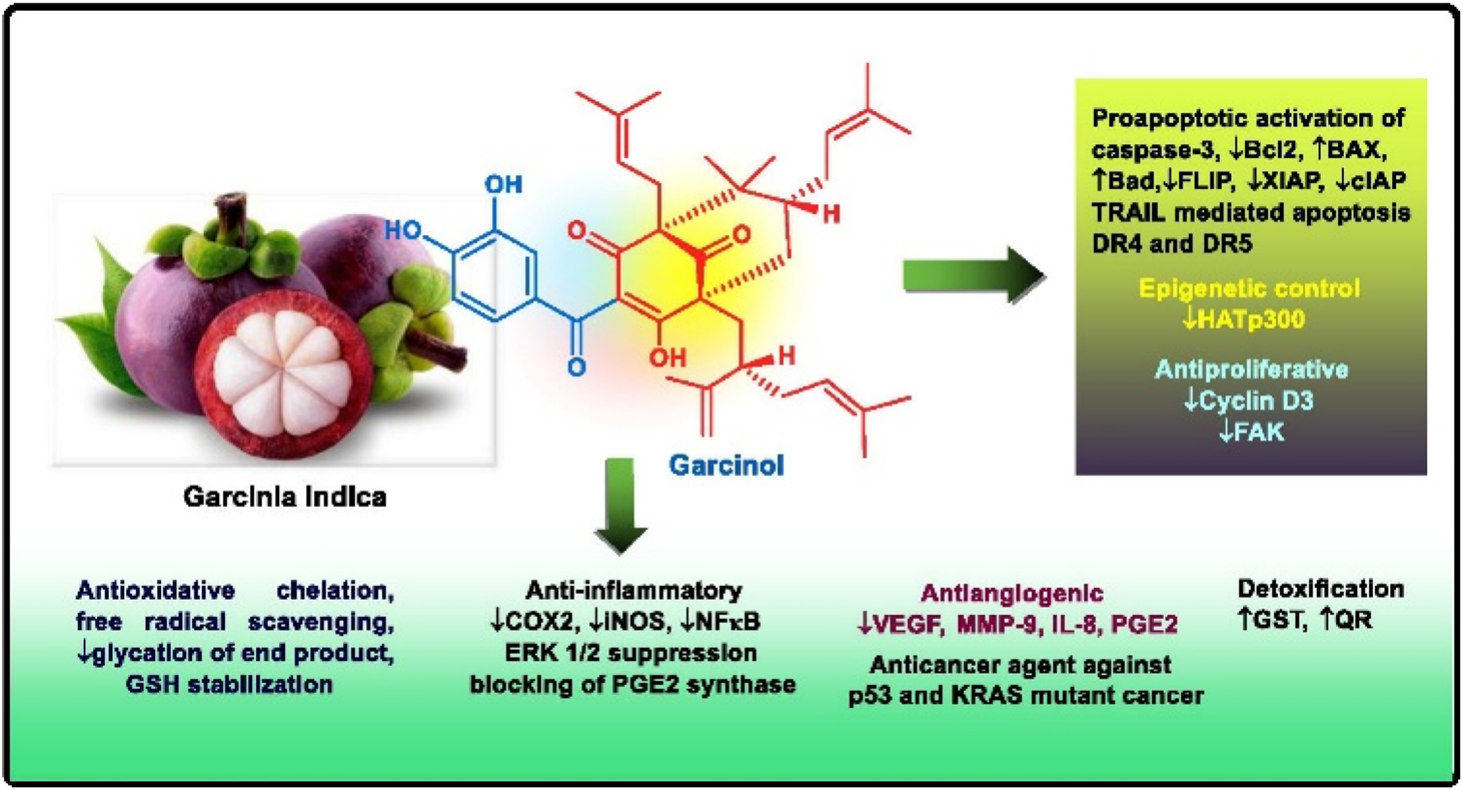
Different mechanistic targets for the execution of anticancer activities by garcinol or camboginol.

Garcinol is also an important polyisoprenylated benzophenone containing the bicyclo[3.3.1]nonane moiety; this phytochemical can be extracted from the rind of *Garcinia indica* fruit, widely renowned as *Kokum* or *Mangosteen* as well as from the leaves of this plant, a medicinal plant profusely found in tropical regions. Among all the chemical components obtained from this fruit extract including citric acid, oxalic acid, hydroxycitric acid (HCA), and hydroxycitric acid lactone, the benzophenone derivatives such as garcinol and its isomer isogarcinol are very important in context to their diverse biological activities (Fig. 5). The anticarcinogenic capabilities of garcinol seem to be evolved due to its antiinflammatory, antioxidative, antiangiogenic, and proapoptotic activities, rendering remarkable epigenetic influences through the inhibition of histone acetyltransferases (HATs) enzymes and posttranscriptional deregulation in the expression of miRNA profiles accountable to carcinogenesis.^341^ In light of the *in vitro* and *in vivo* studies, it has been revealed that the antineoplastic efficacies of garcinol is manifested in consequence of inhibition of various cellular incidents with the regulation of transcription factors JAK/STAT3 and NF-κB in tumor cells, thereby seizing the rapid escalation of malignant cells. Therefore, the potential anticancer aptitude of garcinol has recently been reflected in different oncological reformations in breast cancer, colon cancer, hepatocellular carcinoma, leukemia, prostate cancer, pancreatic cancer, head and neck cancer, *etc.*, and this behavior of garcinol has drawn it toward preclinical trials. Although a clean perception of its mechanism of action is still a matter of profound research, it has been notified that garcinol is capable of suppressing tumorigenesis by diminishing the expression of COX2, iNOS, NF-κB, and STAT3 function along with the inhibition of histone deacetylase 11 (HDAC11) among all other HDACs. Also, it can restrain the function of 5-lypoxygenase and microsomal prostaglandin E2 synthase 1(mPGES-1).^342^

Esophageal cancer is also a very serious type of cancer and common cause of death due to its poor prognosis as well as irregular geographical distribution. The two main histological types of esophageal cancers are (1) esophageal adinocarcinoma and (2) esophageal squamous cell carcinoma (ESCC). The ESCC type of esophageal cancer is seen to pervade mostly in Eastern Asia and Africa. The statistical report from United States, China, and Europe reveals that less than 21% of esophageal cancer-affected people can survive upto 5 years. Cancer cell metastasis is assumed to be the prime cause of death in esophageal cancer. Garcinol has been seen to be very active to hold back the metastasis and is thus potent toward esophageal cancer.^343^

**Fig. 6:**
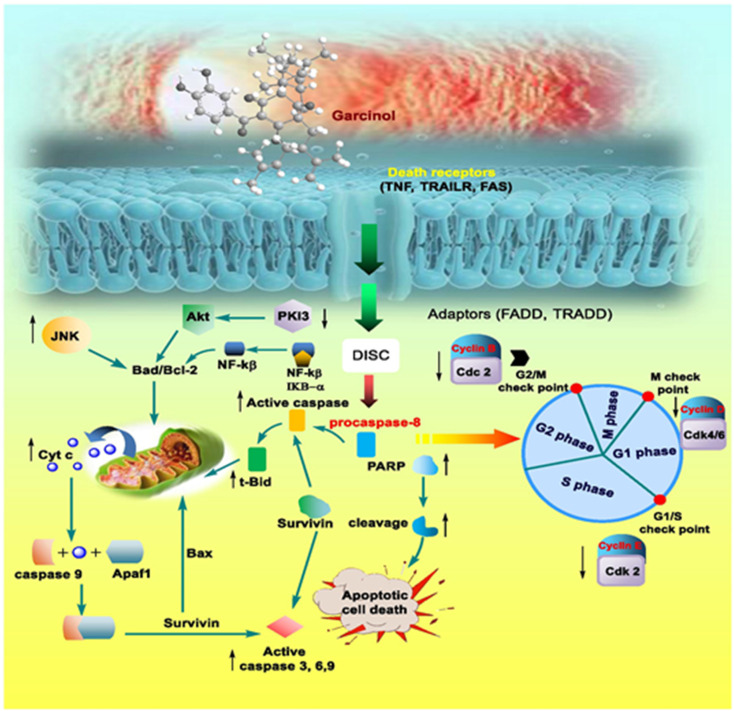
Schematic representation of the mechanism of action of garcinol (↑ upregulation and ↓ downregulation) on treatment against cancer cells.

As garcinol is a histone acetyltransferase (HAT) inhibitor, it is very much efficient to bring on cell cycle arrest and apoptosis in the cancer cell (Fig. 6). Besides, garcinol is also very potent to resist angiogenesis and metastasis, two important hallmarks of cancer by modulating several proinflammatory signaling pathways, which leads to the inhibition of angiogenesis in malignant cells. Upon the intraperetoneal administration of garcinol in xenograft mice model, Li *et al.* observed that garcinol can deregulate the activity of oncogenic transcription factors such as STAT3/NF-κB in a dose as well as time-dependant manner in case of head and neck carcinoma (HNSCC). On the other hand, the inactivation of STAT3/NF-κB can pin down the overexpression of several kinases such as janus kinase 1/2 (JAK1/2), TGF-β-activated kinase 1 (TAK1), c-Src, and inhibitor of Iκβ kinase (IKK) in HNSCC cells and garcinol was successfully reported to hinder the growth in an athymic nu/nu mice. Therefore, a number of studies have been accomplished with the potency of garcinol against different types of cancers (Table 2).

**Table 2:** Anticancer effects of garcinol observed in *in vitro* studies

Types of cancer	Effects	Garcinol concentration
Melanoma, glioblastoma, cervical cancer, breast cancer, leukemia, lung cancer, hepatocellular carcinoma, pancreatic cancer, colon cancer, prostate cancer	Increased apoptosis	2.5–50 μM
Melanoma, hepatocellular carcinoma, leukemia, colon cancer, pancreatic cancer	↑Caspase-3, ↑caspase-9	0–50 μM
Breast cancer, hepatocellular carcinoma, lung cancer	Cell cycle arrest, ↓cyclinsB, D1, D3, and E	0–50 μM, 500 ppm
Melanoma, glioblastoma, breast cancer, hepatocellular carcinoma, colon cancer	↑Bax, ↑Bad, Bcl-2, Bcl-xl	0–50 μM
Breast cancer, oral squamous cell carcinoma, prostate cancer, pancreatic cancer	↓NF-κB signaling pathway	0–50 μM
Breast cancer, gallbladder cancer, pancreatic cancer, prostate cancer, colon cancer	↓MMP2, ↓MMP9	0–30 μM
Breast cancer, pancreatic cancer, prostate cancer, hepatocellular carcinoma	p-STAT3 and STAT3 signaling pathway	0–50 μM
Oral squamous cell carcinoma, breast cancer, hepatocellular carcinoma, colon cancer, prostate cancer, pancreatic cancer	↓VEGF	0–25 μM
Hepatocellular carcinoma, prostate cancer, pancreatic cancer	↓IL-6	0–25 μM
Glioblastoma, lung cancer, breast cancer, pancreatic cancer	↑mi RNA	0–40 μM
Esophageal cancer, breast cancer	HAT inhibition	0–50 μM

**Fig. 7:**
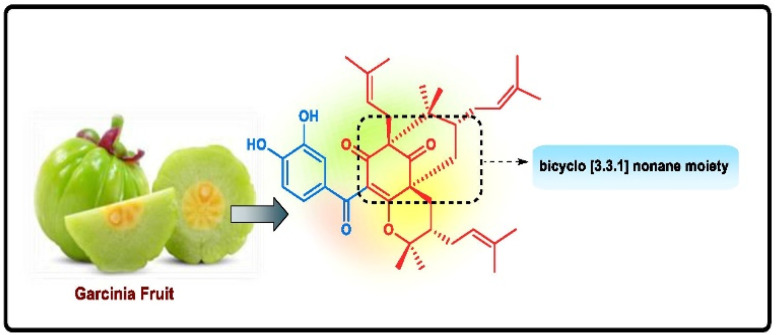
The chemical structure of *cambogin* (isogarcinol) extracted from garcinia fruit having bicyclo[3.3.1]nonane core moiety.

Isogarcinol or cambogin, which is known to be capable of bringing about astounding anticancer activity, also belongs to polycyclic polyprenylated acylphoroglucinol (PPAs) group having bicyclo[3.3.1]nonane as a core moiety (Fig. 7). This valuable compound can also be extracted from *Garcinia genus*, which was traditionally used for treating cancer throughout Southern Asia. Cambogin was found to have remarkable anticancer activity due to its impressive proapoptotic effects on medulloblastoma as well as breast cancer cells. Among all the types of cancer, breast cancer is now assumed to be the most serious type of cancer in women, which is mainly seen to be come out due to some risk factors such as obesity, hormone replacement therapy during menopause, consumption of alcohol, and ionizing radiation. Recently, it is unfortunate that the multifarious growth of breast tumor and bone metastasis can not be restrained by simple mastectomy and chemotherapy. Therefore, the deterrence and healing of breast cancer necessitates the urgent development of novel therapeutic approaches with the use of effective chemotherapeutic agents. Xu *et al.* isolated a number of polycyclic polyprenylated acylphoroglucinol (PPAs) from *Garcinia genus* and assessed their effect toward the proliferation of breast cancer cells.^344^ However, cambogin is the best among the tested compounds that exhibits notable inhibitory potential toward the rapid growth of breast cancer cells. After the treatment of cambogin against a number of cancer cell lines such as HeLa (human cervical carcinoma), HepG2 (human hepatic carcinoma), A549 (human lung carcinoma), HCT116 (human colon carcinoma), SK-BR-3 (ER^−^ PR^−^ HER2^+^), MCF-7(ER^+^PR^+^HER2^−^), and triple negative breast cancer (TNBC) cell line, MDA-MB-468 (ER^−^ PR^−^ HER2^−^), it was identified that cambogin was much more adept in showing the best cytotoxicity toward breast cancer cell lines, whereas cambogin was reluctant toward normal HMEC-1 cells at a similar dosage.^345^

It was also apparent that MCF-7 cells were very much susceptible to cambogin treatment in a time- and dose-dependant manner. In addition, MDA-MB-468 and SK-BR-3 cells were also compelled not to be proliferated under the time- and dose-dependant treatment of cambogin.

**Fig. 8:**
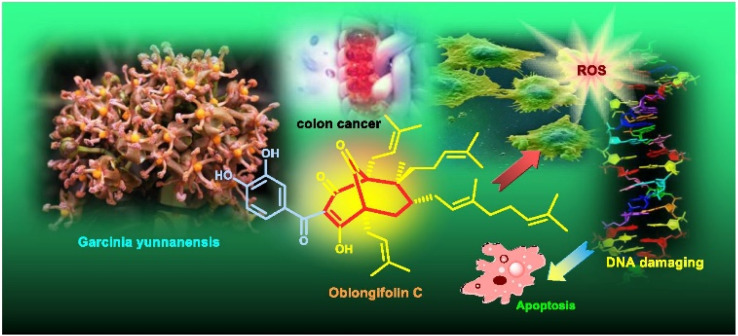
The chemical structure of cambogin oblongifolin C extracted from *Garcinia yunnanensis Hu* having bicyclo[3.3.1]nonane core moiety.

Oblongifolin C is another naturally-obtained biologically active compound with the bicyclo[3.3.1]nonane core moiety. It is mainly extracted from *Garcinia yunnanensis* Hu, and this compound has been observed to be very competent to exhibit promising anticancer activity *in vitro* as well as *in vivo* (Fig. 8). It is well known to inhibit human silent information regulator 1 and 2 (SIRT1 and SIRT2), where SIRT1 is much more susceptible to oblongifolin C than SIRT2. It is efficient to restrict the proliferation of cancer cells, thereby causing cellular apoptosis through the activation of proapoptotic Bax protein along with mitochondrial dysfunction. Oblongifolin C has been seen to increase the sensitivity of gemcitabine-resistant pancreatic cancer cells by suppressing the Src, ERK/MAPK, Akt pathways and regulating the action of the proteins that are responsible for cell cycle progression.

**Fig. 9:**
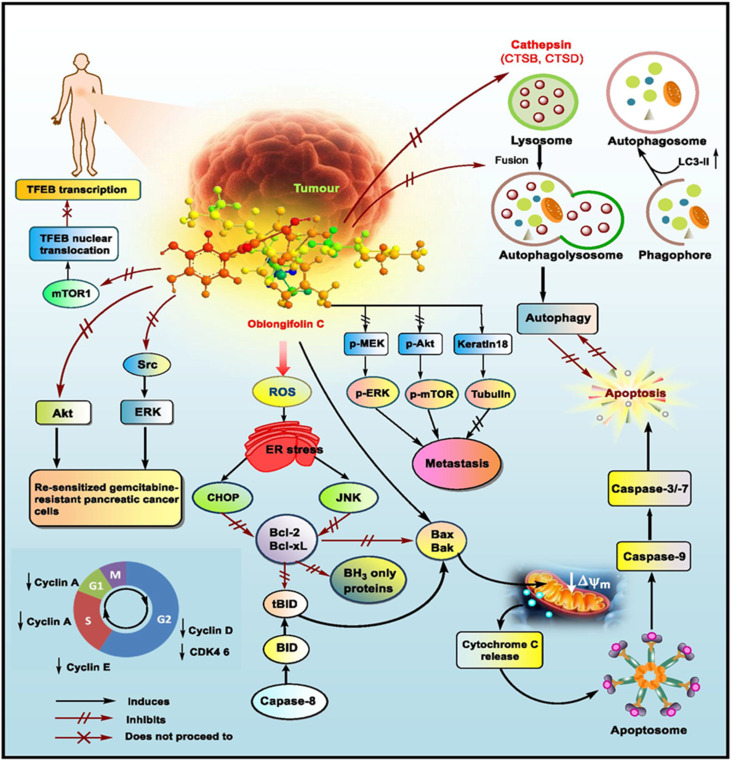
Mechanism of anticancer action induced by oblongifolin C.

In human colorectal cells, oblongifolin C, along with guttiferone K, can cause apoptosis by enhancing the profuse production of ROS and increase the phosphorylation of the JNK protein. Moreover, oblongifolin C can create ER stress, which may lead to the destruction of cancer cells by triggering the transcription factor CHOP and activating the JNK kinases. Oblongifolin C is a very powerful anticancer agent as it causes DNA damage by rupturing the DNA double-strand and inhibits the repair mechanism to mend the damage of DNA. It is very efficient to restrain the metastasis of cancer through keratin 18/tubulin, MEK/ERK, and Akt/mTOR signaling pathways. Also, it can inhibit the autophagic flux by obstructing the autophagosome-lysosome fusion and by changing the lysosomal proteolytic activity (Fig. 9).^346^

Guttiferone k is an anticancer natural compound that is also obtained from *Garcinia yunnanensis* Hu having the bicyclo[3.3.1]nonane core moiety. It accelerates c-MYC protein degradation and stabilizes the FBXW7 protein levels. Consequently, it exerts anticancer activity through the obstruction of cell cycle re-entry in quiescent cancer cells. In line with this, it can trigger Akt-mTOR-mediated autophagy. It is seen to be very proficient to hinder the metastasis in human hepatocellular carcinoma *in vitro* as well as *in vivo* through the modulation of actin binding protein profiling 1 (PFN 1).^347^

Cluisianone and its derivative, which is isolated from *Garcinia parvifolia*, can produce excellent cancer annihilation property that induces mitochondrial dysfunction and apoptotic cell death. It compels the downregulation of the β-tubulin proteins in cancer cells and impairs the activity of CDK1 and cyclin B1 to inhibit the progression of the cell cycle.^348^ Its active epimer, 7-*epi*-cluisianone, succeeded in bringing about cell death in 25 cancer cell lines out of 60 human cancer cell types through the modification of the immune system, prevention of angiogenesis, along with cancer cell invasion in the body.

**Fig. 10:**
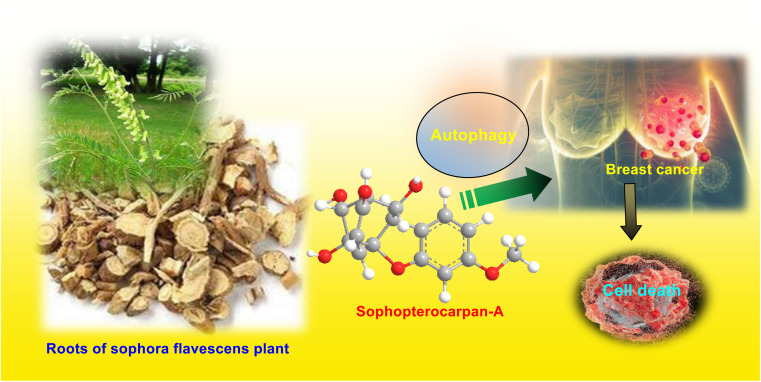
Sophopterocarpan-A isolated from the roots of *Sophora flavescenes* and its anticancer activity.

**Scheme 163:**
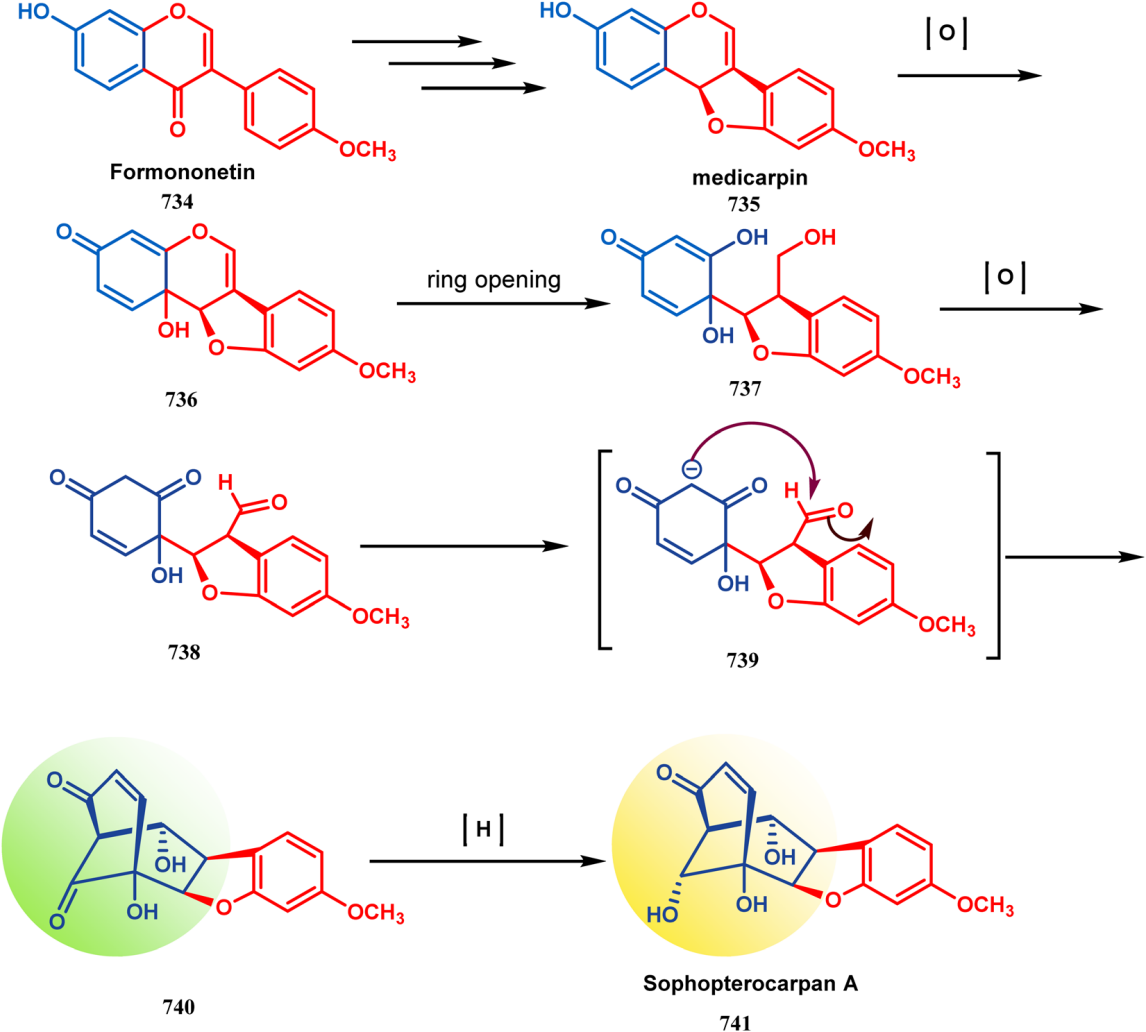
Synthesis of sophopterocarpan-A.

The remarkable pharmacological activities of some alkaloids, terpenoids, dibenzoyl glycosides, and flavonoids-containing bicyclo[3.3.1]nonane moiety isolated from the roots of *sophora flavescens*, a well known deciduous shrub, attracted the researchers for use in the treatment of cancer. Therefore, Zhang *et al.*^349^ synthesized highly potential sophopterocarpan-A (741) having a benzotetrahydrofuran-fused bicyclo[3.3.1]nonane ring, which triggered the autophagic pathway to destroy the cancer cells (Fig. 10, Scheme 163). The *in vitro* anticancer proficiency of sophopterocarpan A was monitored against breast cancer cell line (MCF-7), human lung carcinoma cell line (A549), and human liver cancer cell line (HepG2). It was found that this compound was highly competent to inhibit the growth of MCF-7 cells rather than other cancer cell lines, revealing an IC_50_ of 29.36 μM. The tumor growth inhibition potential of this compound was found to take place through the autophagy mechanism, where cellular dilapidation eliminated the cancer endorsing factors and thereby led to the demolition of cancer cells. This autophagic mechanism was analyzed in the autophagy-detecting system with respect to curcumin as the positive control.^350^

**Scheme 164:**
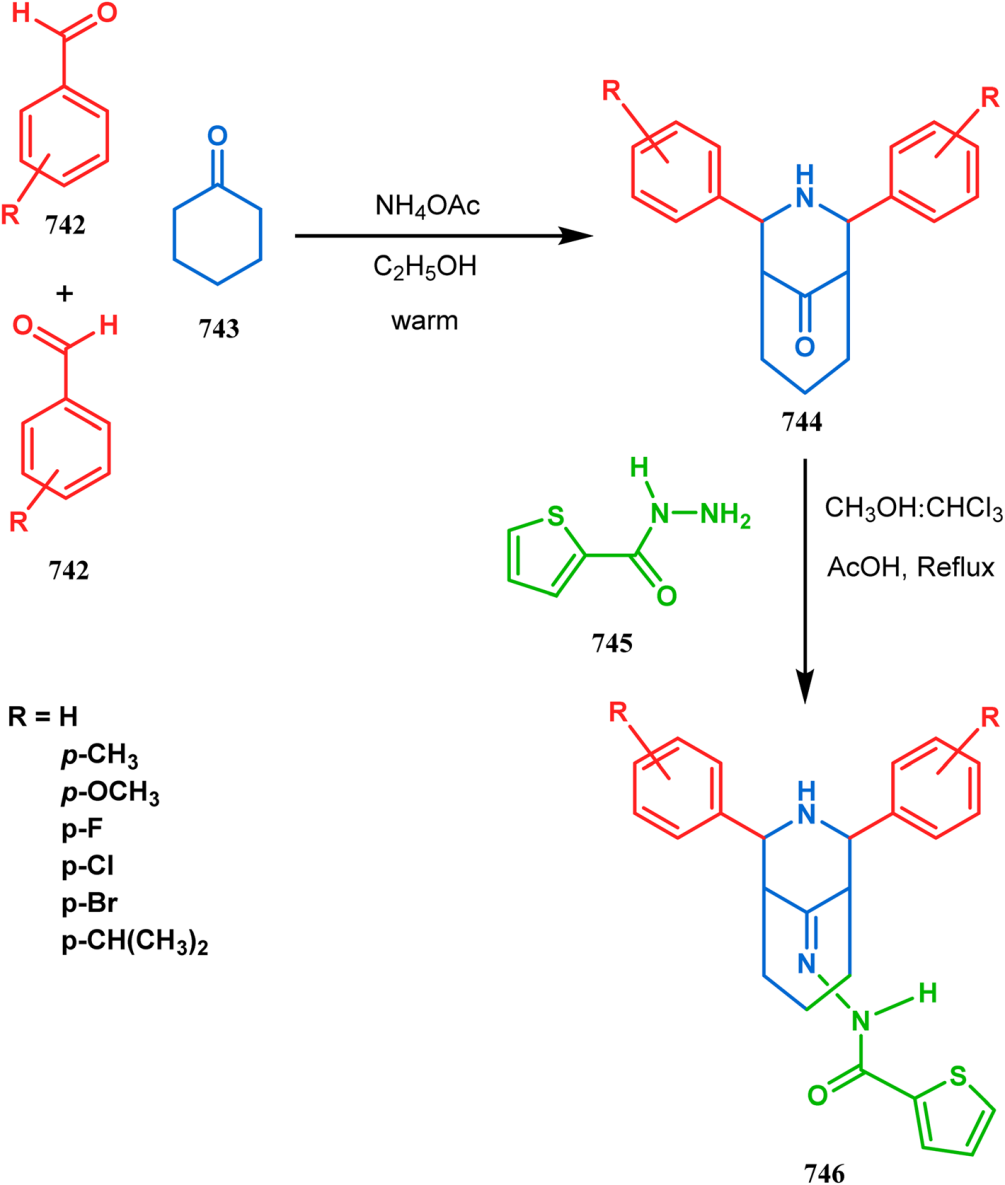
Synthesis of halogen group-substituted 2,4-diaryl-3-azabicyclo[3.3.1]nonan-9-one.

Human liver cancer is now being considered as a very crucial cause of death throughout the world. Oxidative stress due to the excessive production of reactive oxygen species (ROS) in cells has been diagnosed as one of the various hallmarks of cancer. In this case, researchers have found out that the antioxidant can act as a protective substance to prevent multistage carcinogenesis because a living organism has its own enzymatic as well as nonenzymatic antioxidant functions, which can withstand against oxidative damages induced by ROS. Therefore, much more attention had been paid for seeking out some synthetic antioxidants capable of targeting various signaling pathways in cancer. Researchers observed that naturally obtained diterphenoid or norditerphenoid alkaloids possessing 3-azabicyclo[3.3.1]nonane pharmacophore have many biological activities. It was found that the derivatives of 2,4-diaryl-3-azabicyclo[3.3.1]nonan-9-one exhibited excellent antitumor activity along with antimicrobial activity. To correlate the antioxidant property and cytotoxicity of the natural product, T. Balasankar *et al.* synthesized electron-withdrawing halogen group-substituted 2,4-diaryl-3-azabicyclo[3.3.1]nonan-9-one, and they observed strong cytotoxicity but poor antioxidant properties of the synthesized compounds, which may be due to the prooxidant effect (Scheme 164).^350^ But with the substitution of electron-donating groups (–CH_3_, –OCH_3_, –CH(CH_3_)_2_) in the aryl rings of azabicyclo[3.3.1]nonan-9-ones, the antioxidant property was increased and cytotoxicity was decreased.

**Scheme 165:**
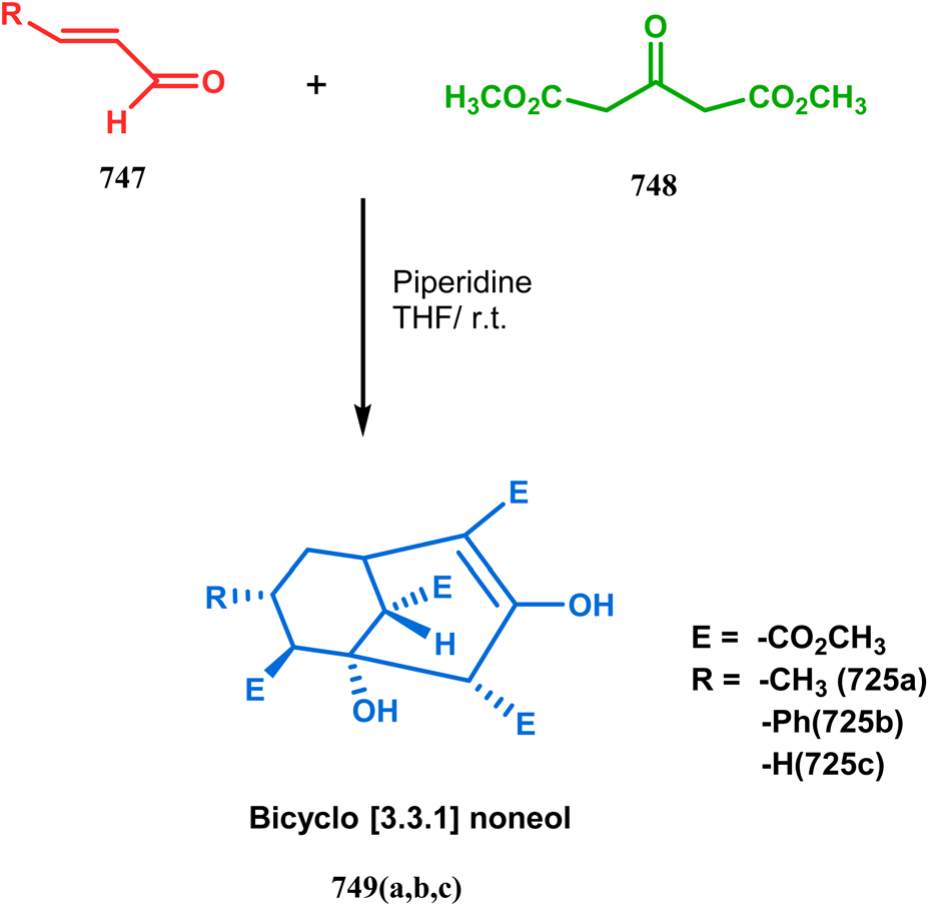
Synthesis of bicyclo[3.3.1]nonenols.

To know the antagonist drug potency, they recorded the IC_50_ values of the compounds by treating the compounds against human liver hepatocellular carcinoma (HepG2) cells. The introduction of electron-withdrawing –F, –Cl, and –Br substitution at the para position of diaryl rings in the azabicyclo[3.3.1]nonan-9-ones exhibited strong cytotoxicity compared to the substitution of the electron donating –CH_3_, –OCH_3_, and –CH(CH_3_)_2_ groups. Among all the compounds, the fluoro (-F)-substituted compound showed the best cytotoxicity (3.76 μg mL^−1^) for 48 h of incubation. The image of the compounds-treated apoptotic cells using Hoechst stain under a fluorescent microscope revealed considerable cell shrinkage and chromatin condensation (pyknosis) along with the disintegration of the nucleus. Also, they studied the free radical scavenging activity of all these compounds against DPPH̄, OH̄, and O_2̄_^−^ radicals. It was recorded that the methoxy (–OCH_3_)–substituted compound showed the best antioxidant property (Scheme 165, Table 3).

**Table 3:** Bicyclo[3.3.2]nonene derivatives (749–752)

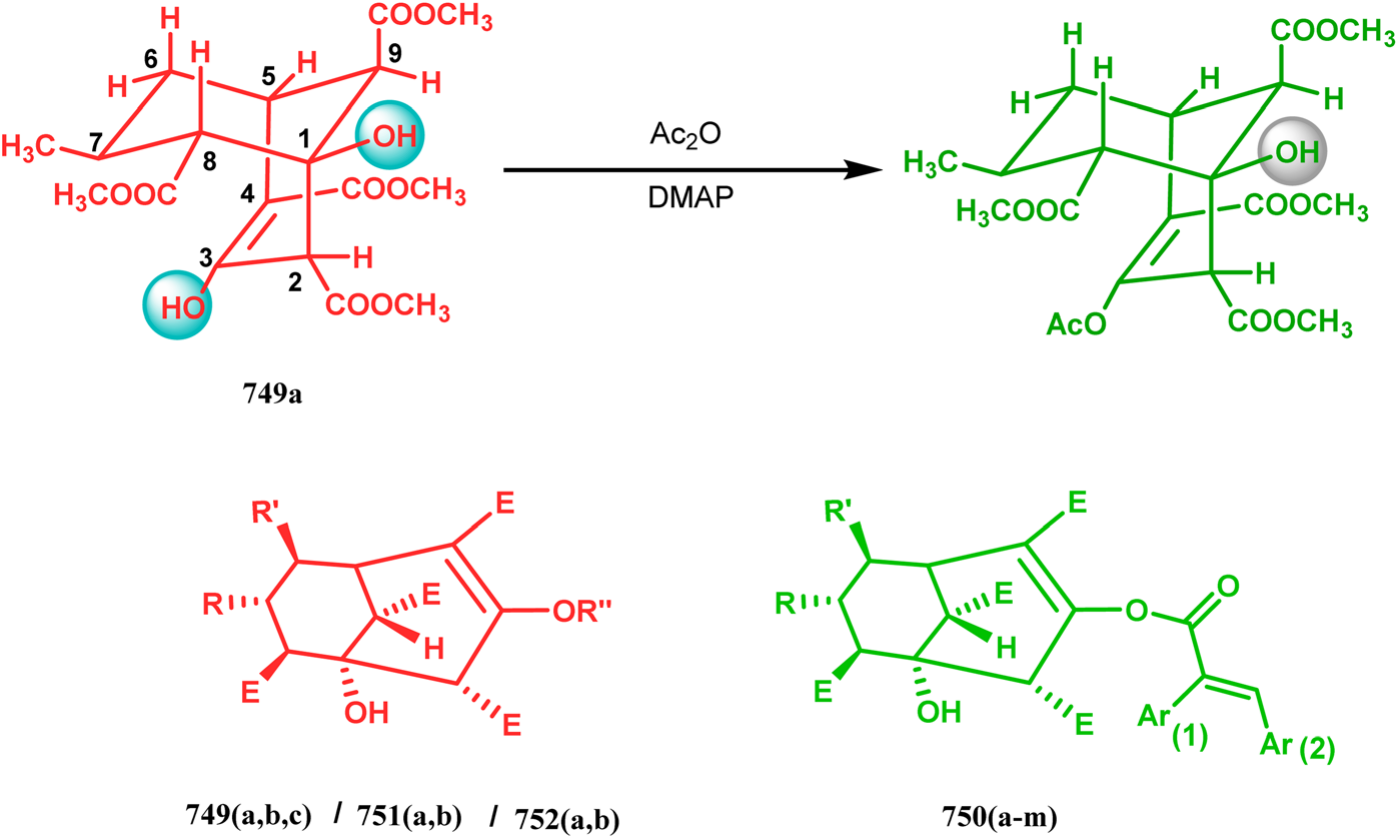							
Compd	R	R′	R′′	Compd	R	Ar(1)	Ar(2)
749a	Me	H	H	750a	Me	H	Ph
749b	Ph	H	H	750b	Me	H	4-Chlorophenyl
749c	H	H	H	750c	Me	Ph	Ph
751a	Me	H	–COPh	750d	Me	Ph	4-Chlorophenyl
751b	Ph	H	–COPh	750e	Me	Ph	2,6-Difluorophenyl
752a	Me	Me	H	750f	Me	4-Chlorophenyl	Ph
752b	Ph	Me	H	750g	Me	2,4-Dichlorophenyl	Ph
—	—	—	—	750h	Me	4-Nitrophenyl	Ph
—	—	—	—	750i	Me	4-Methoxyphenyl	Ph
—	—	—	—	750j	Me	4-Chlorophenyl	4-Chlorophenyl
—	—	—	—	750k	Me	2,4-Dichlorophenyl	2,4-Dichlorophenyl
—	—	—	—	750l	Ph	H	4-Chlorophenyl
—	—	—	—	750m	Ph	4-Chlorophenyl	4-Chlorophenyl

As bicyclo[3.3.1]nonane is a very important framework in biologically-active natural products, it always deserves the evaluation of its biological importance. Therefore, J. Valgeirsson and coworkers synthesized bicyclo[3.3.1]nonenols, and their anticancer activities were screened against various human cancer cell lines (Scheme 164). The prepared bicyclo[3.3.1]noneols were sent to the National Cancer Institute (NCI) to justify their *in vitro* cytotoxic behavior against 60 tumor cell lines. The compounds showed anticancer activity in the GI_50_ range of 1–100 μM (Table 4).^351^

**Table 4:** GI_50_ values (μM) of bicyclo[3.3.1]nonenes against different human cancer cell lines

Compds.	MOLT-4 (leukemia)	H522 (lung)	KM12 (colon)	SF-539 (CNS)	M14 (melanoma)	SK-OV-3 (ovarian)	A498 (renal)	DU-145 (prostate)	MCF-7 (breast)
749a	26.3	25.2	23.5	18.4	20.5	26.0	19.4	20.2	34.8
749b	11.6	6.71	11.7	12.6	10.8	17.4	17.2	12.9	5.93
749c	29.4	10.6	21.6	18.6	22.6	19.2	16.5	44.7	35.6
750a	>100	5.59	27.9	19.8	25.5	21.4	13.3	18.0	19.3
750b	15.8	6.81	34.1	17.6	23.2	24.6	—	39.4	—
750c	—	45.4	>100	>100	>100	>100	>100	>100	>100
750d	>100	5.99	>100	>100	>100	>100	5.55	>100	>100
750e	>100	6.76	>100	>100	>100	>100		>100	—
750f	79.2	>100	>100	>100	>100	>100	>100	>100	>100
750g	>100	>100	>100	>100	>100	>100	>100	>100	>100
750h	3.53	77.2	>100	51.5	>100	>100	>100	>100	>100
750i	>100	35.3	>100	>100	>100	80.3	>100	>100	>100
750j	3.62	10.8	26.0	>100	>100	>100	>100	>100	>100
750k	>100	64.1	>100	>100	>100	>100	>100	>100	>100
750l	3.45	1.96	4.33	3.41	3.46	24.6	6.04	5.80	—
750m	2.22	3.84	>100	>100	30.1	64.0	—	>100	—
750a	46.1	13.2	28.4	14.5	28.3	29.5	26.4	59.2	29.0
750b	3.64	—	8.6	2.20	1.95	10.6	16.3	9.40	3.02
752a	>100	26.9	34.7	35.7	67.5	49.4	>100	>100	38.1
752b	86.0	18.9	>100	>100	88.1	>100	—	>100	—

## 5. Conclusion and future perspectives

The synthesis, derivatization, and bioevaluation of bicyclo[3.3.1]nonane and its heteroanalogues have always been the center of growing interest. Although this important moiety was discovered more than a century ago, chemists are still developing so far unknown enities based on this important framework, while biologists continue to unveil newer areas where its phamacophoric potential is being tested. Also, the global necessity of employment of green chemistry in recent times has witnessed the fast-growing efforts of researchers to investigate the possible environment-friendly modifications of these methodologies using several green tools, including solventless techniques, solid-supports, microwave irradiations, and water as the solvent. Besides, several occurrence of bicyclo[3.3.1]nonane in a numerous bioactive natural products and its anticancer activity have attracted the attention of several research groups worldwide to develop novel synthetic routes for the construction of this bicycle with selective functionalization, both from asymmetric and nonasymmetric point-of-view to design anticancer scaffolds. It can be hoped that the chemistry of bicyclo[3.3.1]nonanes will be beneficial for developing selective anticancer drugs in the imminent future.

## Conflicts of interest

There are no conflicts to declare.

## Supplementary Material

## References

[cit1] Demeunynck M. and Tatibouet A., Recent Developments in Tröger’s Base Chemistry, In Progress in Heterocyclic Chemistry, ed. G. W. Gribble and T. L. Gilchrist, Pergamon, Oxford, UK, 1999, vol. 11, p. 1

[cit2] Chen J. S., Watson W. H., Kagan J., Agdeppa D. A., Chen S.-A. (1978). The structure of diethyl (1R*,4S*,5R*,8R*)-4,8-dimethyl-2,3;6,7-dibenzo-9-oxabicyclo[3.3.1]nona-2,6-diene-4,8-dicarboxylate. Acta Crystallogr., Sect. B: Struct. Crystallogr. Cryst. Chem..

[cit3] Levkin P. A., Strelenko Y. A., Lyssenko K. A., Schurig V., Kostyanovsky R. G. (2003). Temperature-dependent racemic compound-conglomerate crystallization of 2,3:6,7-dibenzobicyclo[3.3.1]nona-2,6-diene-4,8-dion. Tetrahedron: Asymmetry.

[cit4] Naemura K., Fukunaga R., Komatsu M., Yamanaka M., Chikamatsu H. (1989). Synthesis and Enantiomer Recognition of Dipodands and Crown Ethers Containing the 2,3:6,7-Dibenzobicyclo[3.3.1]nona-2,6-diene Residue as the Chiral Subunit. Bull. Chem. Soc. Jpn..

[cit5] Tröger J. (1887). Ueber einige mittelst nascirenden Formaldehydes entstehende Basen. J. Prakt. Chem..

[cit6] Harmata M., Kahraman M. (2000). Congeners of Troeger's base as chiral ligands. Tetrahedron: Asymmetry.

[cit7] Bailly C., Laine W., Demeunynck M., Lhomme J. (2000). Enantiospecific Recognition of DNA Sequences by a Proflavine Tröger Base. Biochem. Biophys. Res. Commun..

[cit8] Johnson R. A., Gorman R. R., Wnuk R. J., Crittenden N. J., Aiken J. W. (1993). Troeger's base. An alternate synthesis and a structural analog with thromboxane A2 synthetase inhibitory activity. J. Med. Chem..

[cit9] Sharma A., Besnard C., Gǵeńee L., Lacour J. (2012). Asymmetric synthesis of ethano-Tröger bases using CuTC-catalyzed diazo decomposition reactions. Org. Biomol. Chem..

[cit10] Haritakun R., Srikitikulchai P., Khoyaiklang P., Isaka M., Isariotins A. -D. (2007). Alkaloids from the Insect Pathogenic Fungus Isaria tenuipes BCC 7831. J. Nat. Prod..

[cit11] Blount J. F., Dunlop R. W., Erickson K. L., Wells R. J., Robert J. (1982). Two diterpenes with new carbocyclic ring systems from an Australian collection of the brown alga Dictyota dichotoma. Aust. J. Chem..

[cit12] Dewanjee S., Maiti A., Das A. K., Mandal S. C., Dey S. P. (2009). Swietenine: A potential oral hypoglycemic from Swietenia macrophylla seed. Fitoterapia.

[cit13] Baggett S., Protiva P., Mazzola E. P., Yang H., Ressler E. T., Basile M. J., Weinstein B., Kennelly E. J. (2005). Bioactive Benzophenones from Garcinia xanthochymus Fruits. J. Nat. Prod..

[cit14] Amagata T., Minoura K., Numata A., Gymnastatins F. −H. (2006). Cytostatic Metabolites from the Sponge-Derived Fungus Gymnascella dankaliensis. J. Nat. Prod..

[cit15] Kraus G. A., Jeon I. (2008). Progress towards the synthesis of papuaforin A: selective formation of α-bromoenones from silyl enol ethers. Tetrahedron Lett..

[cit16] Namjoshi O. A. and Cook J. M., Sarpagine and related alkaloids, The Alkaloids, 2016, vol. 76**,**pp. 1099–4831.(Book Chapter) 10.1016/bs.alkal.2015.08.002 PMC4864735 26827883

[cit17] Buchanan G. L. and Lloyd D. Ed., Logos Press: London, The Biosynthesis of Carbocyclic Compounds, Topics in Carbocyclic Chemistry, 1969, vol. 1, p. 199

[cit18] (h) Demeunynck M. and Tatibouet A., Progress in Heterocyclic Chemistry, ed. G. W. Gribble and T. L. Gilchrist, Elsevier Science Ltd., Oxford, 1999, vol. 11, p. 1

[cit19] Filippini M.-H., Rodriguez J. (1999). Synthesis of Functionalized Bicyclo[3.2.1]octanes and Their Multiple Uses in Organic Chemistry. Chem. Rev..

[cit20] Casanova J., Koukoua G., Waegell B. (1990). BICYCLO[4.2.1]nonane skeleton-conformational-analysis, synthesis, reactivity. Bull. Soc. Chim. Fr..

[cit21] Allinger N. L., Tribble M. T., Miller M. A., Wertz D. H. (1971). Conformational analysis. LXXI. Calculation of nuclear magnetic resonance spectra by semiempirical methods. I. Proton chemical shifts in hydrocarbons. J. Am. Chem. Soc..

[cit22] Engler E. M., Andose J. D., Schleyer P. V. R. (1973). Critical evaluation of molecular mechanics. J. Am. Chem. Soc..

[cit23] Osawa E., Aigami K., Imamoto Y. (1979). Application of Force Field Calculations to Organic Chemistry. IV: Steric Analysis of Synthesis and Structure of 1,4- Dihydroxytricyclo (6.4.0.0) Dodecane-7,10-Dione. Dynamic Conformational Calculations of its Hydrocarbon Skeleton and related systems (Bicyclo (3.3.1) nonane and Bicyclo (3.3.2) Decane). J. Chem. Soc., Perkin Trans. 2.

[cit24] Mastryukov V. S., Popik M. V., Dorofeeva O. V., Golubinskii A. V., Vilkov L. V., Belikova N. A., Allinger N. L. (1981). Chair-boat equilibriums in bicyclo[3.3.1]nonane at 65 and 400.degree.C studied by electron diffraction and molecular mechanics. J. Am. Chem. Soc..

[cit25] Anet F. A. L., Basus V. J. (1973). Detection of a crown family conformation in cyclooctane by proton and carbon-13 nuclear magnetic resonance. *J. Am. Chem. Soc.*.

[cit26] Paroli R. M., R Gilson D. F., Butler I. S. (1998). Spectroscopic and Differential Scanning Calorimetric Studies of the Order-Disorder Phase Transition in Bicyclononanone. J. Solid State Chem..

[cit27] Jaime C., Osawa E., Takeuchi Y., Camps P. (1983). Application of empirical potential energy calculations to organic chemistry. Part 19. Conformational preference in 2,4-dimethoxybicyclo[3.3.1]nonan-9-one and related molecules. Analysis of vicinal NMR coupling constants in multiple rotor system by combined molecular mechanics and generalized Karplus equation. J. Org. Chem..

[cit28] Gundertofte K., Liljefors T., Norrby P.-O., Pettersson I. (1996). A comparison of conformational energies calculated by several molecular mechanics methods. J. Comput. Chem..

[cit29] Raber D. J., Janks C. M., Johnston Jr. M. D., Raber N. K. (1980). Structure elucidation with lanthanide induced shifts. 9. Bicyclo [3.3.1]nonan-9-one. Tetrahedron Lett..

[cit30] Mora A. J., Fitch A. N. (1999). The low-temperature structures of bicyclo[3.3.1]nonan-9-one and 3-azabicyclo[3.2.2]nonane. Z. Kristallogr..

[cit31] Anet F. A. L., Bradley C. H., Buchanan G. W. (1971). Direct detection of the axial conformer of methylcyclohexane by 63.1MHz carbon-13 nuclear magnetic resonance at low temperatures. J. Am. Chem. Soc..

[cit32] Grilli S., Lunazzi L., Mazzanti A. (2000). Conformational Studies by Dynamic NMR. 76.^1^ Stereodynamics of Ring Inversion of Bicyclo[3.3.1]nonan-9-one. J. Org. Chem..

[cit33] Jeyaraman R., Avila S. (1981). Chemistry of 3-azabicyclo[3.3.1]nonanes. Chem. Rev..

[cit34] Quast H., Mü ller B. (1980). Stereochemie von Tetraaryl-3, 7-diazabicyclo [3.3. 1] nonanen und Tetraaryl-1, 3-diazaadamantanen. Chem. Ber..

[cit35] Jackman L. M., Dunne T. S., Müller B., Quast H. (1982). Conformation in solution of tetraaryl-3, 7-diazabicyclo[3.3.1]nonanes and tetra-and pentaaryl-1, 3-diazaadamantanes. A nuclear magnetic resonance study. Chem. Ber..

[cit36] (b) Allinger N. L. and Yuh Y. H., QCPE, 1980, No 395

[cit37] McCabe P. H., Milne N. J., Sim G. A. (1989). Conformations of derivatives of 3,7-diazabicyclo[3.3.1]nonan-9-one. Comparison of 3-ethoxycarbonyl-7-methyl-1,5-diphenyl-3,7-diazabicyclo[3.3.1]nonan-9-one and 3,7-di(ethoxycarbonyl)-1,5-diphenyl-3,7-diazabicyclo[3.3.1]nonan-9-one: effect of a nucleophile ⋯ electrophile interaction on molecular geometry. J. Chem. Soc., Perkin Trans. 2.

[cit38] Gdaniec M., Pham M., Połoński T. (1997). Conformation and Stereodynamics of N, N′-Dinitroso-2,4,6,8-tetraaryl-3,7-diazabi-cyclo[3.3.1]nonanes. J. Org. Chem..

[cit39] Mikhailov B. M. and Bubnov Y. N., Organoboron Compounds in Organic Synthesis, Harwood Academy Science Publishing, Chur, 1984

[cit40] Gunter H. , NMR-Spectroscopie, Georg Thieme Verlag, Stuttgart, 1973

[cit41] Gurskii M. E., Gridnev I. D., Bubnov Y. N., Pelter A., Rademacher P. (1999). Conformational behavior of 3-borabicyclo [3.3. 1] nonanes: 1. Study of molecular dynamics in 3-methoxy-7α-phenyl-1, 5-dimethyl-3-borabicyclo [3.3. 1] nonane. J. Organomet. Chem..

[cit42] Gurskii M. E., Lyssenko K. A., Karionova A. L., Belyakov P. A., Potapova T. V., Antipin M. Y., Bubnov Y. N. (2004). Unique stereochemistry of 3-borabicyclo [3.3. 1] nonane derivatives. Russ. Chem. Bull..

[cit43] Lyssenko K. A., Antipin M. Y., Gurskii M. E., Bubnov Y. N., Karionova A. L., Boese R. (2004). Characterization of the B⋯π-system interaction via topology of the experimental charge density distribution in the crystal of 3-chloro-7α-phenyl-3-borabicyclo[3.3.1]nonane. Chem. Phys. Lett..

[cit44] Zefirov N. S. and Palyulin V. A., *In* Topics in Stereochemistry, John Wiley & Sons, Inc., Hoboken, NJ, 1991, vol. 20, pp. 171–230

[cit45] Choo J., Kim S., Joo H., Kwon Y. J. (2002). The molecular structure and conformation of bicyclo [3.3. 1] nonan-9-one: ab initio and DFT calculations. J. Mol. Struct.: THEOCHEM.

[cit46] Zefirov N. S. (1977). The problem of conformational effects. Tetrahedron.

[cit47] Pisarev S. A., Palyulin V. A. (2021). Conformational effects of 1, 5, 9-substitution in symmetric bicyclo [3.3.1] nonane analogues. Mendeleev Commun..

[cit48] Gleiter R., Kobayashi M., Zefirov N. S., Palyulin V. A. (1977). Photoelectron-spectra and interaction of lone e pairs in 3, 7, 9-triheteroderivatives of bicyclo [3.3.1] nonane. Proc. Natl. Acad. Sci..

[cit49] Ferro-Costas D., Otero N., Grana A. M., Mosquera R. A. (2012). A QTAIM- based energy partitioning for understanding the physical origin of conformational preferences: Application to the Z effect in O=C-X-R and related units. J. Comput. Chem..

[cit50] Tsubomoto Y., Hayashi S., Nakanishi W., Mapp L. K., Coles S. J. (2018). High-resolution X-ray diffraction determination of the electron density of 1-(8-PhSC_10_H_6_)SS(C_10_H_6_SPh-8′)-1′ with the QTAIM approach: evidence for S_4_ σ(4c-6e) at the naphthalene *peri*-positions. RSC Adv..

[cit51] Bushmarinov I. S., Antipin M. Yu., Akhmetova V. R., Nadyrgolova G. R., Lyssenko K. A. (2008). Stereoelectronic Effects in N-C-S Systems: Experimental and ab Initio AIM Study. J. Phys. Chem. A.

[cit52] Bushmarinov I. S., Fedyanin I. V., Lyssenko K. A., Lapteva V. L., Pisarev S. A., Palyulin V. A., Zefirov N. S., Antipin M. Y. (2011). The “Hockey Sticks” Effect Revisited: The Conformational and Electronic Properties of 3,7-Dithia-1,5-diazabicyclo[3.3.1]nonane from the QTAIM Perspective. J. Phys. Chem. A.

[cit53] Jeyaraman R., Avila S. (1981). Chemistry of 3-azabicyclo [3.3. 1] nonanes. Chem. Rev..

[cit54] Kolocouris A. (2009). CHax⋯Yax Contacts in Cyclohexane Derivatives Revisited−Identification of Improper Hydrogen-Bonded Contacts. J. Org. Chem..

[cit55] Hulme A. T., Johnston A., Florence A. J., Fernandes P., Shankland K., Bedford C. T., Welch G. W. A., Sadiq G., Haynes D. A., Motherwell W. D. S., Tocher D. A., Price S. L. (2007). Search for a Predicted Hydrogen Bonding Motif− A Multidisciplinary Investigation into the Polymorphism of 3-Azabicyclo [3.3. 1] nonane-2, 4-dione. J. Am. Chem. Soc..

[cit56] Martinez A. G., Barcina J. O., Albert A., Cano F. H., Subramanian L. R. (1993). 7, 7-Diphenylnorbornane: The first cofacial diphenylmethane derivative. Tetrahedron Lett..

[cit57] Barnes J. C., Paton J. D., Damewood J. R., Mislow K. (1981). Crystal and molecular structure of diphenylmethane. J. Org. Chem..

[cit58] Martinez A. G., Barcina J. O., Cerezo A. F., Rivas R. G. (1998). Hindered rotation in diphenylmethane derivatives. Electrostatic vs charge-transfer and homoconjugative aryl− aryl interactions. J. Am. Chem. Soc..

[cit59] Casarini D., Rosini C. (2003). Conformational Studies by Dynamic NMR. 93.1 Stereomutation, Enantioseparation, and Absolute Configuration of the Atropisomers of Diarylbicyclononanes. J. Org. Chem..

[cit60] Sergeyev S. (2009). Recent Developments in Synthetic Chemistry, Chiral Separations, and Applications of Tröger's Base Analogues. Helv. Chim. Acta.

[cit61] Peters J. A. (1979). Synthesis of Bicyclo [3.3. 1] nonanes. Synthesis.

[cit62] Knoevenagel E. (1894). 1, 5-Diketone. Justus Liebigs Ann. Chem..

[cit63] Knoevenagel E. (1903). Ueber Condensationsproducte von Acetylaceton mit Aldehyden. Ber. Dtsch. Chem. Ges..

[cit64] Knott P. A., Mellor J. M. (1971). Synthesis of bicyclo[3,3,1]nona-3,7-diene-2,6-diones and bicyclo[3, 3,1]-nona-3,6- diene-2,8-diones. *J. Chem. Soc. C*.

[cit65] Theobald D. W. (1969). On the reaction of acetoacetic ester with (+)-carvone: An oxabicyclo [3.3. 1] nonene and some bicyclo [3.3. 1] nonanolones. Tetrahedron.

[cit66] Rabe P. (1908). Zur Kenntniss der 1, 5-Diketone. Ueber die Umlargerung von 1, 5-Diketonen in cyklische Ketonalkohole and uber die Synthese bicyklischer Ketonalkohole mit Bruckenbindung. Justus Liebigs Ann. Chem..

[cit67] Rabe P. (1904). Synthesen bicyclischer Systeme mit Brückenbindung. III. Mittheilung: Ueber die Anlagerung von Acetessigester an Methyl-cyclohexenon. Ber. Dtsch. Chem. Ges..

[cit68] Meerwein H., Schurmann W. (1913). Uber eine Synthese von Abkommlingen des Bicyclo-[1, 3, 3]-nonans. Justus Liebigs Ann. Chem..

[cit69] Meerwein H. , German Patent 277467, 1913 Chem. Zentralbl., 1914, 2, 740

[cit70] Meerwein H., Kiel F., Klosgen G., Schoch E. (1922). Über bicyclische und polycyclische Verbindungen mit Brückenbindung. Über das Bicyclo-[1, 3, 3]-nonan und seine Abkömmlinge. J. Prakt. Chem..

[cit71] Aoyagi K., Nakamura H., Yamamoto Y. (1999). A Concise and Stereospecific One-Shot Synthesis of Bicyclo[3.3.1]nonenols from Dimethyl 1,3-Acetonedicarboxylate and Enals via the Sequential Michael Addition−Intramolecular Aldolization. J. Org. Chem..

[cit72] Wang D., Crowe W. E. (2010). One-carbon bridge stereocontrol in Robinson annulations leading to bicyclo [3.3. 1] nonanes. Org. Lett..

[cit73] Theobald D. W. (1969). On the reaction of acetoacetic ester with (+)-carvone: An oxabicyclo [3.3. 1] nonene and some bicyclo [3.3.1] nonanolones. Tetrahedron.

[cit74] Kraus G. A., Hon Y. S. (1985). Bridgehead intermediates in organic synthesis: two direct syntheses of (.+-.)-lycopodine. J. Am. Chem. Soc..

[cit75] Kraus G. A., Hon Y. S. (1987). The Total Synthesis of Lycopodine using Bridgehead Intermediates. Heterocycles.

[cit76] Grossman R. B., Ley S. V. (1994). Chemistry of insect antifeedants from Azadirachta indica (Part 17): Synthesis of model compounds of azadirachtin. Unusual effect of remote substituents on the course of the oxidative ring contraction reaction. Tetrahedron.

[cit77] Kraus G. A., Jeon I. (2005). Preparation of complex bridged bicyclic ring systems from 3,3-diacetoxy-2-phenylsulfonylpropene and β-keto esters. Tetrahedron.

[cit78] Murayama K., Tanabe T., Ishikawa Y., Nakamura K., Nishiyama S. (2009). A synthetic study on gymnastatins F and Q: the tandem Michael and aldol reaction approach. Tetrahedron Lett..

[cit79] Usuda H., Kanai M., Shibasaki M. (2002). Studies toward the Total Synthesis of Garsubellin A: A Concise Synthesis of the 18-epi-Tricyclic Core. Org. Lett..

[cit80] Usuda H., Kanai M., Shibasaki M. (2002). Studies toward the total synthesis of garsubellin A: synthesis of 8-deprenyl-garsubellin A. Tetrahedron Lett..

[cit81] Shimizu Y., Kuramochi A., Usuda H., Kanai M., Shibasaki M. (2007). A new approach for the construction of a highly congested bicyclic system in polycyclic polyprenylated acylphloroglucinols (PPAPs). Tetrahedron Lett..

[cit82] Shimizu Y., Shi S.-L., Usuda H., Kanai M., Shibasaki M. (2010). The first catalytic asymmetric total synthesis of ent-hyperforin. Tetrahedron.

[cit83] Mehta G., Bera M. K. (2008). A concise approach towards the bicyclo [3.3. 1] nonan-9-one core present in the phloroglucin natural product hyperforin. Tetrahedron Lett..

[cit84] Pouplin T., Tolon B., Nuhant P., Delpech B., Marazano C. (2007). Synthetic Studies Towards Bridgehead Diprenyl-Substituted Bicyclo[3.3.1]nonane-2,9-diones as Models for Polyprenylated Acylphloroglucinol Construction. Eur. J. Org. Chem..

[cit85] Itagaki N., Kimura M., Sugahara T., Iwabuchi Y. (2005). Organocatalytic Entry to Chiral Bicyclo[3.n.1]alkanones via Direct Asymmetric Intramolecular Aldolization. Org. Lett..

[cit86] Nicolaou K. C., Carenzi G. E. A., Jeso V. (2005). Construction of Highly Functionalized Medium-Sized Rings: Synthesis of Hyperforin and Perforatumone Model Systems. Angew. Chem., Int. Ed..

[cit87] Ciochina R., Grossman R. B. (2003). A New Synthetic Approach to the Polycyclic Polyprenylated Acylphloroglucinols. Org. Lett..

[cit88] Michaelides I. N., Darses B., Dixon D. J. (2011). Acid-Catalyzed Synthesis of Bicyclo[3.n.1]alkenediones. Org. Lett..

[cit89] Kuninobu Y., Morita J., Nishi M., Kawata A., Takai K. (2009). Rhenium-catalyzed formation of bicyclo [3.3. 1] nonene frameworks by a reaction of cyclic β-keto esters with terminal alkynes. Org. Lett..

[cit90] Klein A., Miesch M. (2003). New cascade reactions starting from acetylenic ω-ketoesters: an easy access to electrophilic allenes and to 1, 3-bridgehead ketones. Tetrahedron Lett..

[cit91] Liu H. J., Ho L. K., Lai H. K. (1981). Annelation of β-keto thiolesters and synthetic application. Can. J. Chem..

[cit92] Guo J., Bai X., Wang Q., Bu Z. (2018). Diastereoselective Construction of Indole-Bridged Chroman Spirooxindoles through a TfOH catalyzed Michael Addition-Inspired Cascade Reaction. J. Org. Chem..

[cit93] Takagi R., Nerio T., Miwa Y., Matsumura S., Ohkata K. (2004). Construction of the bicyclo[3.3.1]nonenone core by successive Michael reactions of 2-cyclohexenone derivatives. Tetrahedron Lett..

[cit94] Qi J., Porco Jr. J. A. (2007). Rapid Access to Polyprenylated Phloroglucinols via Alkylative Dearomatization−Annulation: Total Synthesis of (±)-Clusianone. J. Am. Chem. Soc..

[cit95] Takagi R., Inoue Y., Ohkata K. (2008). Construction of the adamantane core of Plukenetione-type polycyclic polyprenylated acylphloroglucinols. J. Org. Chem..

[cit96] Gambacorta A., Tofani D., Tafi A., Farah M. A. (2001). Chair–boat equilibrium as driving force
in epimerization of 3,7-dimethylbicyclo[3.3.1]nonan-2,9-dione derivatives. Stereocontrolled synthesis of the 3-exo,7-exo- and 3-endo,7-exo-dimethylbicyclo[3.3.1]nonan-9-ones. Tetrahedron.

[cit97] Kraus G. A., Dneprovskaia E., Nguyen T. H., Jeon I. (2003). Synthesis of a model system for the preparation of phloroglucinol containing natural products. Tetrahedron.

[cit98] Boeckman Jr. R. K., Arvanitis A., Voss M. E. (1989). Synthetic studies directed toward naturally occurring cyclooctanoids. 1. A total synthesis of (.+-.)-ceroplastol I. J. Am. Chem. Soc..

[cit99] Kalaivani D., Vasuki M., Santhi S. (2011). Mechanism and linear free energy relationships in the kinetics of formation of bicyclo[3.3.1]nonane derivatives from 1,3,5-trinitrobenzene,phenyl-substituted1-benzyl-1-(ethoxycarbonyl)-2-propanones, and triethylamine. Int. J. Chem. Kinet..

[cit100] Heim R., Wiedemann S., Williams C. M., Bernhardt P. V. (2005). Expedient construction of the vibsanin E core without the use of protecting groups. Org. Lett..

[cit101] Tilly D. P., Williams C. M., Bernhardt P. V. (2005). Construction of the cyclovibsanin core via a biogenetically modeled approach. Org. Lett..

[cit102] Schwartz B. D., Tilly D. P., Heim R., Wiedemann S., Williams C. M., Bernhardt P. V. (2006). Towards the Total Synthesis of Vibsanin E, 15-*O*-Methylcyclovibsanin B, 3-Hydroxyvibsanin E, Furanovibsanin A, and 3-*O*-Methylfuranovibsanin A. Eur. J. Org. Chem..

[cit103] Mak J. Y. W., Williams C. M. (2012). Enantioselective total synthesis of (-)-neovibsanin G and (-)-14-epi-neovibsanin G. *Chem. Commun.*.

[cit104] Siegel D. R., Danishefsky S. J. (2006). Total synthesis of garsubellin A. *J. Am. Chem. Soc*
**.**.

[cit105] Tsukano C., Siegel D. R., Danishefsky S. J. (2007). Differentiation of nonconventional “carbanions”—the total synthesis of nemorosone and clusianone. Angew. Chem., Int. Ed..

[cit106] Jackson W. P., Ley S. V., Whittle A. J. (1980). Selenium-mediated cyclization reactions of alkenyl-substituted β-ketoesters. Chem. Commun..

[cit107] Nicolaou K. C., Pfefferkorn J. A., Kim S., Wei H. X. (1999). Synthesis of the fully functionalized bicyclic core of garsubellin A. J. Am. Chem. Soc..

[cit108] Nicolaou K. C., Pfefferkorn J. A., Cao G.-Q., Kim S., Kessabi J. (1999). A facile method for the solution and solid-phase synthesis of substituted [3.3. 1] bicycles. Org. Lett..

[cit109] Hediger M. E. (2004). Design, synthesis, and evaluation of aza inhibitors of chorismate mutase. Bioorg. Med. Chem..

[cit110] Bartlett P. A., Johnson C. R. (1985). An inhibitor of chorismate mutase resembling the transition-state conformation. J. Am. Chem. Soc..

[cit111] Bartlett P. A., Nakagawa Y., Johnson C. R., Reich S. H., Luis A. J. (1988). Chorismate mutase inhibitors: synthesis and evaluation of some potential transition-state analogs. Org. Chem..

[cit112] Spessard S. J., Stoltz B. M. (2002). Progress toward the synthesis of garsubellin A and related phloroglucins: the direct diastereoselective synthesis of the bicyclo [3.3. 1] nonane core. Org. Lett..

[cit113] Casarini D., Rosini C. (2003). Conformational Studies by Dynamic NMR. 93.1 Stereomutation, Enantioseparation, and Absolute Configuration of the Atropisomers of Diarylbicyclononanes. J. Org. Chem..

[cit114] Rodeschini V., Simpkins N. S., Wilson C. (2007). Kinetic resolution in a bridgehead lithiation mediated by a chiral Bis-lithium amide: Assignment of the absolute configuration of clusianone. J. Org. Chem..

[cit115] Rodeschini V., Ahmad N. M., Simpkins N. S. (2006). Synthesis of (+/−)-Clusianone: High-Yielding Bridgehead and Diketone Substitutions by Regioselective Lithiation of Enol Ether Derivatives of Bicyclo[3.3.1]nonane-2,4,9-triones. Org. Lett..

[cit116] Nuhant P., David M., Pouplin T., Delpech B., Marazano C. (2007). α, α′-Annulation of 2, 6-Prenyl-Substituted Cyclohexanone Derivatives with Malonyl Chloride: Application to a Short Synthesis of (±)-Clusianone. Formation and Rearrangement of a Biogenetic-Like Intermediate. Org. Lett..

[cit117] Ahmad N. M., Rodeschini V., Simpkins N. S., Ward S. E., Blake A. J. (2007). Synthesis of polyprenylated acylphloroglucinols using bridgehead lithiation: the total synthesis of racemic clusianone and a formal synthesis of racemic garsubellin A. J. Org. Chem..

[cit118] Kende A. S., Roth B., Sanfilippo P. J. (1982). Facile, palladium (II)-mediated synthesis of bridged and spirocyclic bicycloalkenones. J. Am. Chem. Soc..

[cit119] Kende A. S., Roth B., Sanfilippo P. J., Blacklock T. J. (1982). Mechanism and regioisomeric control in palladium (II)-mediated cycloalkenylations. A novel total synthesis of (.+-.)-quadrone. J. Am. Chem. Soc..

[cit120] Gao Y.-Q., Hou Y., Zhu L., Chen J., Li R., Zhang S.-Y., He Y.-P., Xie W. (2020). Visible-Light Driven Synthesis of Polycyclic Benzo[d][1,3]oxazocine From 2-Aminochalcone. Chem. Commun..

[cit121] Gao Y.-Q., Hou Y., Zhu L., Chen G., Xu D., Zhang S.-Y., He Y.-P., Xie W. (2019). A bio-inspired synthesis of hybrid flavonoids from 2-hydroxychalcone driven by visible light. RSC Adv..

[cit122] Yang Z., He Y., Toste F. D. (2016). Biomimetic Approach to the Catalytic Enantioseiective Synthesis of Flavonoids. J. Am. Chem. Soc..

[cit123] Wang F., Chen F., Qu M., Li T., Liu Y., Shi M. (2013). A Pd(II)-catalyzed asymmetric approach toward chiral [3.3.1]-bicyclic ketals using 2-hydroxyphenylboronic acid as a pro-bis(nucleophile). Chem. Commun..

[cit124] Ito Y., Aoyama H., Hirao T., Mochizaki A., Saegusa T. (1979). Cyclization reactions via oxo-. pi.-allylpalladium (II) intermediates. J. Am. Chem. Soc..

[cit125] Drouin J., Boaventura M. A., Conia J. M. (1985). Cyclization of acetylenic carbonyl compounds via their silyl enol ether derivatives: a new intramolecular C-vinylation induced by mercury(II) salts. Stereochemistry and functionalization of the intermediate vinylmercurial. J. Am. Chem. Soc..

[cit126] Huang H., Forsyth C. J. (1993). A stereoselective total synthesis of (±)-erythrodiene. Tetrahedron Lett..

[cit127] Huang H., Forsyth C. J. (1995). Anti Selective Spirocarbomercuration: Synthesis and Stereochemistry of the Spirobicyclic Sesquiterpenes Spirojatamol and Erythrodiene. J. Org. Chem..

[cit128] Boaventura M. A., Drouin J. (1987). Cyclisation de composés acétyléniques carbonyls via leur ether d’énol silylé. II. Synthése et réactivité de quelques ethers d’énols derives d’aldéhydes ou de cétones acétyléniques terminaux. Bull. Soc. Chim. Fr..

[cit129] Boaventura M. A., Drouin J., Theobald F., Rodier N. (1987). La cyclisation des composés carbonyls acétyléniques via leurs ethers d’énol silyliques: une nouvelle C-vinylation intramoléculaire induite par les sels mercuriques. Détermination radiocristallographique de la stéréochimie de l’intermédiaire vinylmercurique. Bull. Soc. Chim. Fr..

[cit130] Forsyth C. J., Clardy J. (1990). Total syntheses of (+)-and (-)-didemnenones A and B. Anti selectivity in the intramolecular carbomercuration reaction. J. Am. Chem. Soc..

[cit131] Shigehisa H., Jikihara T., Takizawa O., Nagase H., Honda T. (2008). An exceptional palladium-catalyzed alkenylation of silyl enol ether in the absence of a fluoride additive. Tetrahedron Lett..

[cit132] Gravel D., Benoît S., Kumanovic H., Sivaramakrishnan H. (1992). On the palladium catalyzed reaction of methallyl-1, 1-diacetate with cyclic β-ketoesters. Intervention of hidden mechanisms. Tetrahedron Lett..

[cit133] Kuramochi A., Usuda H., Yamatsugu K., Kanai M., Shibasaki M. (2005). Total synthesis of (±)-garsubellin A. J. Am. Chem. Soc..

[cit134] Shimizu Y., Kuramochi A., Usuda H., Kanai M., Shibasaki M. (2007). A new approach for the construction of a highly congested bicyclic system in polycyclic polyprenylated acylphloroglucinols (PPAPs). Tetrahedron Lett..

[cit135] Shimizu Y., Shi S.-L., Usuda H., Kanai M., Shibasaki M. (2010). The first catalytic asymmetric total synthesis of ent-hyperforin. Tetrahedron.

[cit136] Dias A. de O., Augusti R., dos Santos E. N., Gusevskaya E. V. (1997). Convenient one-pot synthesis of 4, 8-dimethyl-bicyclo [3.3. 1] non-7-en-2-ol via platinum/tin catalyzed hydroformylation/cyclization of limonene. Tetrahedron Lett..

[cit137] Finlay McC., Walton J. C. (1987). Formation of bicyclo [3.2. 1] octane, bicyclo [4.2. 1] nonane, and bicyclo [3.3. 1] nonane by transannular radical cyclisations. Chem. Commun..

[cit138] Quirante J., Escolano C., Massot M., Bonjoch J. (1997). Synthesis of 2-azabicyclo[3.3.1]nonanes by means of (carbamoyl)dichloromethyl radical cyclization. Tetrahedron.

[cit139] Quirante J., Torra M., Diaba F., Escolano C., Bonjoch J. (1999). Synthesis of enantiopure 2-azabicyclo [3.3.1] nonanes by a radical ring closure. Tetrahedron.

[cit140] Ward J., Caprio V. (2006). A radical mediated approach to the core structure of huperzine A. Tetrahedron Lett..

[cit141] Ward J., Caprio V. (2009). Synthesis of the bicyclo [3.3.1] nonane core of huperzine A and novel pyridine-fused tricycles by cyclisation of pyridine-based radicals. Heterocycles.

[cit142] Olga N. Z., Evgeniya V. N., Vladimir I. C., Irina S. S., Danil I. P., Mikhail V. O., Natalia V. G. (2010). Design, synthesis and biotest of a bicyclo [3.3. 1] nonane analogue of 2-amino-5, 6-dihydro-4H-1, 3-thiazine. Mendeleev Commun..

[cit143] Badger G. M., Cook J. W., Walker T. (1949). The synthesis of piperidine derivatives. Part III. 5-Phenyl-1-azabicyclo[3.3.1] nonane. J. Chem. Soc..

[cit144] Slowinski F., Ayad O. B., Vache J., Saady M., Leclerc O., Lochead A. (2010). Synthesis of new bridgehead substituted azabicyclo-[2.2. 1] heptane and-[3.3. 1] nonane derivatives as potent and selective α7 nicotinic ligands. Org. Lett..

[cit145] Graetz B., Rychnovsky S., Leu W.-H., Farmer P., Lin R. (2005). C2-Symmetric nitroxides and their potential as enantioselective oxidants. Tetrahedron: Asymmetry.

[cit146] Michel P., Rassat A. (2000). An easy access to 2, 6-dihydroxy-9-azabicyclo. J. Org. Chem..

[cit147] Pinna G. A., Murineddu G., Curzu M. M., Villa S., Vianello P., Borea P. A., Gessi S., Toma L., Colombo D., Cignarella G. (2000). Synthesis, modelling,
and μ-opioid receptor affinity of N-3 (9)-arylpropenyl-N-9 (3)-propionyl-3, 9-diazabicyclo [3.3. 1] nonanes. ILFarmaco.

[cit148] Lesma G., Pilati T., Sacchetti A., Silvani A. (2008). New chiral diamino ligands as sparteine analogues. Application to the palladium-catalyzed kinetic oxidative resolution of 1-phenyl ethanol. Tetrahedron: Asymmetry.

[cit149] Kuhl U., Englberger W., Haurand M., Holzgrabe U. (2000). Diazabicyclo [3.3. 1] nonanone-type Ligands for the Opioid Receptors. Arch. Pharm. Pharm. Med. Chem..

[cit150] Stoll I., Mix A., Rozhenko A. B., Neumann B., Stammler H.-G., Mattay J. (2008). Kemp's triacid attached to octa-O-methyl resorc [4] arenes: conformations in solution and comparative binding studies with various 2-amino pyridines. Tetrahedron.

[cit151] Park C.-M. (2006). Concise synthesis of 3, 7-dioxa-9-aza-bicyclo [3, 3, 1]-nonane. J. Org. Chem..

[cit152] Yang R.-F., Huang P.-Q. (2010). Studies towards an Enantioselective Total Synthesis of Sarain A: A Concise Asymmetric Construction of the Diazatricyclic Core. Chem. - Eur. J..

[cit153] Paredes R., Abonia R., Cadavid J., Moreno-Fuquen R., Jaramillo A., Hormaza A., Ramirez A., Kennedy A. (2002). A mechanistic study of the ammonolysis of alkyl acetoacetates in water. Formation of 1, 5-dimethyl-2, 6, 9-triaza-bicyclo [3.3. 1] nonane-3, 7-dione as the main product. Tetrahedron.

[cit154] Rudler H., Parlier A., Hamon L., Herson P., Chaquin P., Daran J.-C. (2009). Straightforward access to tetrahydropyridine and piperidine-fused fluorolactones from pyridines and bis (trimethylsilyl) ketene acetals. Tetrahedron.

[cit155] Petrov V. A., Marshall W. (2010). Acid catalyzed cyclodimerisation of 2, 2-bis (trifluoromethyl)-4-alkoxy-oxetanes and-thietanes. Synthesis of 2, 2, 6, 6-tetrakis(trifluoromethyl)-4,8-dialkoxy-1,5-dioxocanes and 3, 3, 7, 7- tetrakis(trifluoromethyl)-9-oxa-2, 6-dithia-bicyclo[3.3.1] nonane. Beilstein J. Org. Chem..

[cit156] Chau F. H. V., Corey E. J. (2006). Short and simple synthesis of chelating bis-ethers and bis-amines in the bicyclo [3.3. 1] nonane series. Tetrahedron Lett..

[cit157] Fleischhauert J., Harmatatt M., Kahramantt M., Koslowskit A., Welch C. J. (1997). The determination of the absolute configuration of a chiral molecular tweezer using CD spectroscopy. Tetrahedron Lett..

[cit158] Harmata M., Barnes C. L. (1990). Molecular clefts. 3. The crystal structure of a chiral molecular tweezer and its guest. J. Am. Chem. Soc..

[cit159] Wallentin C.-J., Wixe T., Wendt O. F., Bergquist K.-E., Warnmark K. (2010). Synthesis and Self-Aggregation of Enantiopure and Racemic Molecular Tweezers Based on the Bicyclo [3.3. 1] nonane Framework. Chem. – Eur. J..

[cit160] Weil E. D., Smith K. J., Gruber R. J. (1966). Transannular addition of sulfur dichloride to cyclooctadienes. J. Org. Chem..

[cit161] Corey E. J., Block E. (1966). New synthetic approaches to symmetrical sulfur-bridged carbocycles. J. Org. Chem..

[cit162] Lautenschlaeger F. K. (1966). The reaction of sulfur dichloride with cis,cis-1,5-cyclooctadiene. Can. J. Chem..

[cit163] Accurso A. A., Cho S.-H., Amin A., Potapov V. A., Amosova S. V., Finn M. G. (2011). Thia-, aza-, and selena [3.3. 1] bicyclononane dichlorides: Rates vs internal
nucleophile in anchimeric assistance. J. Org. Chem..

[cit164] Herzog U., Borrmann H. (2004). Organosilicon chalcogenides with trisilane units-bicyclo [3.3.1]nonanes, bicyclo[3.2.2.]nonanes and spiro[4.4]nonanes. J. Organomet. Chem..

[cit165] Robertson A., Bradaric C., Frampton C. S., McNulty J., Capretta A. (2001). Novel chiral phosphines derived from limonene: the synthesis and structure of 4, 8-dimethyl-2-phosphabicyclo [3.3. 1] nonane. Tetrahedron Lett..

[cit166] Polas A., Wilton-Ely J. D. E. T., Slawin A. M. Z., Foster D. F., Steynberg P. J., Green M. J., Cole-Hamilton D. J. (2003). Limonene-derived phosphines in the cobalt-catalysed hydroformylation of alkenes. Dalton Trans..

[cit167] Wang J. F., Liao Y. X., Kuo P. Y., Gau Y. H., Yang D. Y. (2006). Synthesis and characterization of [1] benzopyrano [4, 3-d][1,3] benzooxazocin-13-one and its derivatives. Synlett.

[cit168] Moghaddam F. M., Mirjafary Z., Saeidian H., Taheri S., Doulabi D., Kiamehr M. (2010). Facile entry to polycyclic indolylhydroquinoline skeletons via tandem C-alkylation and intramolecular S-alkylation. Tetrahedron.

[cit169] Moghaddam F. M., Mirjafary Z., Saeidian H., Taheri S., Soltanzadeh B. (2010). Synthesis of eight-membered hydroquinolines related to alkaloid skeletons via addition of 4-hydroxycoumarin or 4-hydroxypyran-2-one to quinolinium salts. Tetrahedron.

[cit170] Moghaddam F. M., Mirjafary Z., Saeidian H., Taheri S., Khodabakhshi M. R. (2010). A new and convenient approach to heterotetracyclic benzoxazocines through addition of 1, 3-dicarbonyl compounds to quinolinium salts. Tetrahedron Lett..

[cit171] Moghaddam F. M., Saeidian H., Mirjafary Z., Taheri S., Kiamehr M. (2010). The synthesis of dibenzazocines via tandem dinucleophilic addition of phenols to quinolinium salts. Arkivoc.

[cit172] Moghaddam F. M., Taheri S., Mirjafary Z., Saeidian H., Kiamehr M., Tafazzoli M. (2011). A Facile Synthesis of Bridged Polycyclic Naphthooxazocine Skeletons: Eight-Membered-Ring Constructions via Tandem Dinucleophilic Addition of Naphthalenols to Quinolinium Salts. Helv. Chim. Acta.

[cit173] Mondal S., Paira R., Maity A., Naskar S., Sahu K. B., Hazra A., Saha P., Banerjee S., Mondal N. B. (2011). Basic alumina supported tandem synthesis of bridged polycyclic quinolino/isoquinolinooxazocines under microwave irradiation. Tetrahedron Lett..

[cit174] Paira R., Mondal S., Maity A., Sahu K. B., Naskar S., Saha P., Hazra A., Kundu S., Banerjee S., Mondal N. B. (2011). An easy access to diversely fused dioxa-2-aza-tricyclo [n. 3.1. 02, n] tetra/pentadecanes under solvent-free condition. Tetrahedron Lett..

[cit175] Rassadin V. A., Tomashevskiy A. A., Sokolov V. V., Potekhin A. A. (2008). Khim. Geterotsykl. Soed..

[cit176] Rassadin V. A., Tomashevskiy A. A., Sokolov V. V., Ringe A., Magull J., Meijere A. (2009). Facile Access to Bicyclic Sultams with Methyl 1-Sulfonylcyclopropane-1-carboxylate Moieties. Eur. J. Org. Chem..

[cit177] Rassadin V. A., Grosheva D. S., Tomashevskiy A. A., Sokolov V. V., Yufit D. S., Kozhushkov S. I., de Meijere A. (2010). Bicyclic sultams with a nitrogen at the bridgehead and a sulfur atom in the apex position: Facile preparation and conformational properties. Eur. J. Org. Chem..

[cit178] Srikrishna A., Vijaykumar D. (1998). Facile
formation of chiral bicyclo [3.3. 1] nonenes via regioselective cyclopropane cleavage of 1-methyltricyclo [4.3. 0.02, 9] nonan-8-ols. Tetrahedron Lett..

[cit179] Abe M., Nakada M. (2007). Synthetic studies on phloroglucins: a new approach to the bicyclo[3.3.1]nonane system via the regioselective ring-opening of the methoxycyclopropane. Tetrahedron Lett..

[cit180] Abe M., Nakada M. (2006). New construction of the bicyclo [3.3. 1] nonane system via Lewis acid promoted regioselective ring-opening reaction of the tricyclo [4.4. 0.05, 7] dec-2-ene derivative. Tetrahedron Lett..

[cit181] Wipf P., Jeger P., Kim Y. (1998). Thiophilic ring-opening and rearrangement reactions of epoxyketone natural products. Bioorg. Med. Chem. Lett..

[cit182] Hammill J. T., Contreras-García J., Virshup A. M., Beratan D. N., Yang W., Wipf P. (2010). Synthesis and chemical diversity analysis of bicyclo[3.3.1]non-3-en-2-ones. Tetrahedron.

[cit183] Wang Y.-F., Chiba S. (2009). Mn (III)-mediated reactions of cyclopropanols with vinyl azides: synthesis of pyridine and 2-azabicyclo [3.3. 1] non-2-en-1-ol derivatives. J. Am. Chem. Soc..

[cit184] Sasaki T., Eguchi S., Tom T. (1970). Synthesis of adamantane derivatives. XII. Schmidt reaction of adamantan-2-one. J. Org. Chem..

[cit185] Kimoto K., Imagawa T., Kawanisi M. (1972). Reduction of 7-Methylenebicyclo [3.3.1] nonan-3-one and Related Compounds: Structural Investigation of the Products. Bull. Chem. Soc. Jpn..

[cit186] Camps P., Achab R. E., Font-Bardia M., G∼irbig D., Morral J., Mufioz-Torrero D., Solans X., Simon M. (1996). Easy synthesis of 7-alkylbicyclo [3.3. 1] non-6-en-3-ones by silica gel-promoted fragmentation of 3-alkyl-2-oxaadamant-1-yl mesylates. Tetrahedron.

[cit187] Fischer W., Grog C. A. (1978). Infrared and Raman spectroscopic studies of benzothiazolinic spiropyrans and merocyanines. Helv. Chim. Acta.

[cit188] Lukach A. E., Santiago A. N., Rossi R. A. (1997). Reactions of 1,3-Dihaloadamantanes with Carbanions in DMSO: Ring-Opening Reactions to Bicyclo[3.3.1]nonane Derivatives by the SRN1 Mechanism. J. Org. Chem..

[cit189] Cermenati L., Dondi D., Fagnoni M., Albini A. (2003). Titanium dioxide photocatalysis of adamantine. Tetrahedron.

[cit190] Fort Jr. R. C. , Adamantane. The Chemistry of Diamond Molecules, Marcel Dekker, New York, 1976

[cit191] Bagrii E. I. , Adamantany (Adamantanes), Nauka, Moscow, 1989

[cit192] Grob C. A., Baumann W. (1955). Helv. Chim. Acta.

[cit193] Grob C. A. (1957). Das Prinzip der Äthylogie in der organischen Chemie. Experientia.

[cit194] Stetter H., Tacke P. (1962). Neue Synthesen von 2.4-disubstituierten s-Triazinen. Angew. Chem..

[cit195] Grob C. A., Schwartz W. (1964). Die synchrone Fragmentierung von γ-Amino-cycloalkylhalogeniden 1. Teil. Die Solvolyse von 1-Amino-3-brom-adamantanen. Fragmentierungs-Reaktionen. 10. Mitteilung. Helv. Chim. Acta.

[cit196] Shiryaev A. K., Moiseev I. K., Bartsev A. V. (1990). A New Fragmentation of N-Substituted 1-Halo-3-aminoadamantanes (I). Zh. Obshch. Khim..

[cit197] Skomorokhov M. Y., Klimochkin Y. N. (2001). Cyclization of 7-Methylene-3-[2-(4-nitrobenzoyloxy) ethoxy]-bicyclo[3.3.1]non-2-ene in the Presence of Acids. Russ. J. Org. Chem..

[cit198] Skomorokhov M. Y., Klimochkin Y. N. (2011). Reaction of Dibromoadamantanes with Glycols in the Presence
of Sodium Glycolate. Russ. J. Org. Chem..

[cit199] Klaić L., Veljković J., Mlinarić-Majerski K. (2002). Convenient synthesis of novel 1, 3, 7-trisubstituted bicyclo [3.3. 1] nonane derivatives. Synth. Commun..

[cit200] Nurieva E. V., Semenova I. S., Nuriev V. N., Shishov D. V., Baskin I. I., Zefirova O. N., Zefirov N. S. (2010). Diels-alder reaction as a synthetic approach to bicyclo [3.3. 1] nonane colchicine analogs. Russ. J. Org. Chem..

[cit201] Dixon D. D., Sethumadhavan D., Benneche T., Banaag A. R., Tius M. A., Thakur G. A., Bowman A., Wood J. T., Makriyannis A. (2010). Heteroadamantyl cannabinoids. J. Med. Chem..

[cit202] Shibuya M., Tomizawa M., Suzuki I., Iwabuchi Y. (2006). 2-Azaadamantane N-Oxyl (AZADO) and 1-Me-AZADO: Highly Efficient Organocatalysts for Oxidation of Alcohols. J. Am. Chem. Soc..

[cit203] Takahashi K., Watanabe M., Honda T. (2008). Highly Efficient Stereocontrolled Total Synthesis of (+)-Upial. Angew. Chem., Int. Ed..

[cit204] Takahashi K., Akao R., Honda T. (2009). Efficient diastereoselective synthesis of trifarane-type sesquiterpenes, trifarienols A and B. J. Org. Chem..

[cit205] Sparrow K., Barker D., Brimble M. A. (2012). An efficient synthesis of 3-alkyl-1, 5, 3-dioxazepanes and their use as electrophiles in double-Mannich reactions. Tetrahedron.

[cit206] Mukaiyama T., Hoshino T. (1960). The Reactions of Primary Nitroparaffins with Isocyanates. J. Am. Chem. Soc..

[cit207] Young D. G. J., Zeng D. (2002). A Preliminary Approach to Nonenolizable β,β-Tricarbonyls: Assembly of a Hyperevolutin Prototype. J. Org. Chem..

[cit208] Bertz S. H., Dabbagh G. (1982). Synthesis of Bicyclo [3.3.1] nonane Derivatives under Physiological Conditions. Angew. Chem., Int. Ed. Engl..

[cit209] Bertz S. H. (1985). Series on chemistry under physiological conditions. Part 7. Tetramethyl 3,7-dihydroxybicyclo[3.3.1]nona-2,6-diene-2,4,6,8-tetracarboxylate: a useful companion to Meerwein's ester. Topological analysis of bicyclo[3.3.1]nonane synthesis. J. Org. Chem..

[cit210] Sands R. D. J. (1983). Reaction of dimethyl 3-oxoglutarate with 1,3-dicarbonyl compounds. J. Org. Chem..

[cit211] McDonald I. A., Dreiding A. S. (1973). Triasteranetrione. *Helv. Chim. Acta*.

[cit212] Stetters H., Lennartz J. (1977). Über Verbindungen mit Urotropin-Struktur, LIX. Ringschlüsse auf der Basis des Bicyclo [3.3. 1] nonan-3, 7, 9-trions. Liebigs Ann. Chem..

[cit213] Stetter H., Mayer J. (1959). Über Verbindungen mit Urotropin-Struktur, XV. Synthese des 2-Oxa-adamantan-Ringsystems. Chem. Ber..

[cit214] Aranda G., Bernassau J. M., Fetizon M., Hanna I. (1985). Conformational analysis, synthesis, and carbon-13 spectroscopy of 9, 9-dimethylbicyclo [3.3. 1] nonane derivatives. J. Org. Chem..

[cit215] Camps P., El Achab R., Font-Bardia M., GoÈrbig D., Morral J., MunÄoz-Torrero D., Solans X., Simon M. (1996). Easy synthesis of 7-alkylbicyclo [3.3. 1] non-6-en-3-ones by silica gel-promoted fragmentation of 3-alkyl-2-oxaadamant-1-yl mesylates. Tetrahedron.

[cit216] Camps P., GonzaÂlez A., MunÄoz-Torrero D., Simon M., ZuÂnÄiga A., Martins M. A., Font-Bardia M., Solans X. (2000). Synthesis of Polysubstituted Bicyclo [3.3. 1] nonane-3, 7-diones from Cyclohexa-2, 5-dienones and Dimethyl 1, 3-Acetonedicarboxylate. Tetrahedron.

[cit217] Wallentin C.-J., Orentas E., Butkus E., Wärnmark K. (2009). Baker's yeast for sweet dough enables large-scale synthesis of enantiomerically pure bicyclo [3.3. 1] nonane-2, 6-dione. Synthesis.

[cit218] Naemura K., Ida H., Fukuda R. (1993). Lipase YS-catalyzed enantioselective transesterification of alcohols of bicarbocyclic compounds. Bull. Chem. Soc. Jpn..

[cit219] Miyazawa M., Nobata M., Okamura S., Muraoka O., Tanabe G., Kameoka H. (1998). Stereoselective reduction of (±)-bicyclo [3.3. 1] nonane-2, 6-dione by microorganisms. J. Chem. Technol. Biotechnol..

[cit220] Carlquist M., Wallentin C.-J., Wärnmark K., Gorwa-Grauslund M. F. (2008). Genetically engineered Saccharomyces cerevisiae for kinetic resolution of racemic bicyclo [3.3. 1] nonane-2, 6-dione. Tetrahedron: Asymmetry.

[cit221] Okazaki T., Terakawa E., Kitagawa T., Takeuchi K. (2000). Solvolysis of 2-Bicyclo[3.2.2]nonyl p-Toluenesulfonate. Evidence for the Formation of Classical Carbocation Intermediates. J. Org. Chem..

[cit222] Schaefer J. P., Endres L. S., Moran M. D. (1967). Bicyclo [3.3. 1] nonanes. III. Preparation and reactions of bicyclo [3.2. 2] nonanes. J. Org. Chem..

[cit223] Rademacher P., Wiesmann R. F. (1994). Transanulare Wechselwirkungen in difunktionellen mittleren Ringen, 4. Spektroskopische und theoretische Untersuchungen an bicyclischen Boraalkenen. Chem. Ber..

[cit224] Schaefer J. P., Lark J. C., Flegal C. A., Honig L. M. (1967). Bicyclo [3.3. 1] nonanes. II. Synthesis and reactions of simple derivatives. J. Org. Chem..

[cit225] Momose T., Atarashi S., Muraoka O. (1974). Novel steric factors operating on the Baeyer-villiger oxidationof bicyclo [3,3,1] Nonan-3-one system. Tetrahedron Lett..

[cit226] Butkus E., Stoncius S. (2001). Stereoselective Baeyer–Villiger oxidation of some bridged bicyclic diketones. *J. Chem. Soc., Perkin Trans. 1*.

[cit227] Butkus E., Stoncius S., Zilinskas A. (2001). Chirality.

[cit228] Stoncius S., Berg U., Butkus E. (2004). Chiral bicyclic keto lactones: Determination of the absolute configuration by the study of chiroptical properties and chemical correlation. Tetrahedron: Asymmetry.

[cit229] Stephens P. J., McCann D. M., Devlin F. J., Flood T. C., Butkus E., Stoncius S., Cheeseman J. R. (2005). Determination of Molecular Structure Using Vibrational Circular Dichroism Spectroscopy: The Keto-lactone Product of Baeyer−Villiger Oxidation of (+)-(1R,5S)-Bicyclo[3.3.1]nonane-2,7-dione. J. Org. Chem..

[cit230] Gambacorta A., Tofani D., Lupattelli P., Tafi A. (2002). Desymmetrisation of meso-methylcyclooctanones. Highly enantioselective synthesis of C8 syn-isoprenoid and syn, syn-deoxypropionate subunits from a bicyclo[3.3.1]nonane precursor. Tetrahedron Lett..

[cit231] Gambacorta A., Turchetta S., Farah M. E. (1992). BICYCLO[3.3.1]nonane approach to polymethyl alternating systems, the syn-and anti-1,5-dimethyl systems. Gazz. Chim. Ital..

[cit232] Snatzke G., Wolfram B. (1972). Circulardichroismus—L: Chiroptische eigenschaften von thiaadamantanderivaten. Tetrahedron.

[cit233] Gerlach H. (1978). Racematspaltung und Bestimmung der absoluten Konfiguration von 2, 6-disubstituierten Bicyclo [3.3. 1] nonanen. Helv. Chim. Acta.

[cit234] Berg U., Butkus E. (1993). Enantiomer separation and circular dichroism spectra of bicyclo[3.3.1] nonanedione. J. Chem. Res., Synop..

[cit235] Berg U., Butkus E. (1994). An analysis of the circular dichrosm of bicyclo [3.3.1] nonanediones. J. Chem. Res., Synop..

[cit236] Butkus E., Stoncius S., Zilinskas A. (2001). Determination of the absolute configuration of bicyclo [3.3. 1] nonane-2, 7-dione by circular dichroism spectroscopy and chemical correlation. Chirality.

[cit237] Hoffmann G., Wiartalla R. (1982). Ein rascher Zugang zu optisch aktiven Adamantan-Derivaten. Tetrahedron Lett..

[cit238] Butkus E., Berg U., Zilinskas A., Kubilius R., Stoncius S. (2000). Enantiomer separation and absolute configuration of densely functionalized 2-oxatricyclo [4.3. 1.03, 8] decanes by CD spectroscopy and chemical correlation. Tetrahedron: Asymmetry.

[cit239] Butkus E., Zilinskas A., Stoncius S., Rozenbergas R., Urbanova M., Setnicka V., Bour P., Volka K. (2002). Synthesis and chiroptical properties of enantiopure tricyclo [4.3. 0.03, 8] nonane-4, 5-dione (twistbrendanedione). Tetrahedron: Asymmetry.

[cit240] Malinauskiene J., Kadziauskas P., Malinauskas A., Kulys J. (1999). Enzymatic Enantioseparation of Bicyclo [3.3. 1] nonane-2, 6-diones. Monatsh. Chem..

[cit241] Butkus E., Berg U., Malinauskiene J., Sandström J. (2000). Synthesis and Chiroptical Properties of Methanocycloocta[b]indoles, Synthesis and Chiroptical Properties of Methanocycloocta[b]indoles. J. Org. Chem..

[cit242] (a) Lightner D. A. and Gurst J. E., Organic Conformational Analysis and Stereochemistry, Wiley, New York, 2000, ch. 4, pp. 63–94

[cit243] Stephens P. J., McCann D. M., Butkus E., Stoncius S., Cheeseman J. R., Frisch M. J. (2004). Determination of absolute configuration using concerted ab initio DFT calculations of electronic circular dichroism and optical rotation: bicyclo [3.3. 1] nonane diones. J. Org. Chem..

[cit244] Rodeschini V., Simpkins N. S., Wilson C. (2007). Kinetic resolution in a bridgehead lithiation mediated by a chiral Bis-lithium amide: Assignment of the absolute configuration of clusianone. J. Org. Chem..

[cit245] Butkus E. (2001). Stereocontrolled synthesis and reactions of bicyclo [3.3. 1] nonanes. Synlett.

[cit246] Fraga B. M. (2002). Natural sesquiterpenoids. *Nat. Prod. Rep*..

[cit247] Ferraz H. M. C. , Silva Jr. L. F. and Vieira T. O., Thallium (III) in organic synthesis, Synthesis, 1999, pp. 2001–2023

[cit248] Butkus E., Zilinskas A., Stoncius S., Rozenbergas R., Urbanova M., Setnicka V., Volka P. B. K. (2002). Synthesis and chiroptical properties of enantiopure tricyclo [4.3. 0.03, 8] nonane-4, 5-dione (twistbrendanedione). Tetrahedron: Asymmetry.

[cit249] Kubilius R., Bagdziunas G., Butkus E. (2011). Ring contraction/transannular cyclization of chiral bicyclo [3.3. 1] nonanediones mediated by thallium (III) nitrate. Tetrahedron Lett..

[cit250] Duque M. D., Camp P., Profire L., Montaner S., Vázquez S., Sureda F. X., Mallol J., López-Querol M., Naesens L., De Clercq E., Prathalingam S. R., Kelly J. M. (2009). Synthesis and pharmacological evaluation of (2-oxaadamant-1-yl) amines. Bioorg. Med. Chem..

[cit251] Geigy A. G. , *Patent GB* 1123609, 1968

[cit252] Gagneux A. R., Meier R. (1969). 1-Substituted 2-heteroadamantanes. Tetrahedron Lett..

[cit253] Stetter H., Tacke P. (1963). Uber Verbindungen mit Urotropin-Struktur, XXVII Uber eine Fragmentierung in der Adammantan-Reihe. *Chem. Ber.*.

[cit254] Camps P., El Achab R., Font-Bardia M., Görbig D. M., Morral J., Muñoz-Torrero D., Solans X., Simon M. (1996). Easy synthesis of 7-alkylbicyclo [3.3. 1] non-6-en-3-ones by silica gel-promoted fragmentation of 3-alkyl-2-oxaadamant-1-yl mesylates. Tetrahedron.

[cit255] Krasutskii P. A., Serguchev Y. A., Yurchenko A. G., Khotkevich A. V. (1983). Synthesis, in Vitro Pharmacology, and Molecular Modeling of Very Potent Tacrine–Huperzine A Hybrids as Acetylcholinesterase Inhibitors of Potential Interest for the Treatment of Alzheimer's Disease. Teor. Eksp. Khim..

[cit256] Krasutskii P. A., Khotkevich A. V., Serguchev Y. A., Yurchenko A. G. (1985). Mechanism of transannular bromination reactions of diolefins of the bicyclo [3.3. 1] nonane series. Teor. Eksp. Khim..

[cit257] Serguchev Y. A., Khotkevich A. V., Krasutskii P. A. (1985). Dokl. Akad. Nauk. UkrSSR Ser. B..

[cit258] Krasutskii P. A., Ambrosienko N. B., Rodionov V. N., Yurchenko A. G., Parnes Z. N., Bolestova G. I. (1985). Zh. Org. Khim..

[cit259] Serguchev Y. A., Lourie L. F., Ponomarenko M. V. (2000). Selective transannular cyclization of 3, 7-bismethylenebicyclo [3.3.1] with F-TEDA-BF_4_ in protic. Mendeleev Commun..

[cit260] Serguchev Y. A., Lourie L. F., Ponomarenko M. V. (2002). Difluorination ability of F-TEDA-BF_4_ in the transannular cyclization of bicyclo[3.3.1]nonane dienes in monoglyme. Mendeleev Commun..

[cit261] Serguchev Y. A., Ponomarenko M. V., Lourie L. F., Chernega A. N. (2003). Synthesis of halo-fluoro-substituted adamantanes by electrophilic transannular cyclization of bicyclo [3.3. 1] nonane dienes. J. Fluorine Chem..

[cit262] Walters T. R., Zajac Jr. W. W., Woods J. M. (1991). New reagents for the synthesis of gem-halonitro compounds from oximes. J. Org. Chem..

[cit263] Camps P., Munoz-Torrero D. (1994). Alternative syntheses of bridgehead polycyclic 1,2-diamines and 2-aminoalcohols from di- and mono-oximes of some bicyclic diketones: Highly improved synthesis of tricyclo[3.3.1.03,7]nonane-3,7-diamine. Tetrahedron Lett..

[cit264] Zajac Jr. W. W. , U.S. Pat., 5105301, 1992

[cit265] Borden W. T., Ravindranathan T. (1971). Transannular ring closure by reduction of cyclooctane-1, 5-diones. Synthesis of a bisnoradamantan-1-o1. J. Org. Chem..

[cit266] Baughman G. L. (1964). Dibromination of Adamantane. J. Org. Chem..

[cit267] Zalikowski J. A., Gilbert K. E., Borden W. T. (1980). Oxidation of 7-(hydroxymethyl) bicyclo [3.3. 1] nonan-3-ol. Convenient synthesis of bicyclo [3.3. 1] nonane-3, 7-dione. J. Org. Chem..

[cit268] Bertz S. H. (1985). Tetramethyl 3,7-dihydroxybicyclo[3.3.1] nona-2,6-diene-2,4,6,8-tetracarboxylate: a useful companion to meerwein's ester. Topological analysis of bicyclo[3.3.1] nonane synthesis. J. Org. Chem..

[cit269] Ioannou S., Nicolaides A. V. (2009). An improved synthesis of diiodonoradamantane. Tetrahedron Lett..

[cit270] Ponomarenko M. V., Serguchev Y. A., Ponomarenko B. V., Roschenthaler G.-V., Fokin A. A. (2006). Experimental and theoretical studies on the transannular cyclizations of 3, 7-dimethylenebicyclo [3.3. 1] nonane with polyfluoroalkyl radicals. J. Fluorine Chem..

[cit271] Mori T., Kimoto K., Kawanisi M., Nozaki H. (1969). Preparation and reactions of 1,5-polymethylene-bridged 3,7-dimethylenebicyclo[3.3.1]nonan-9-ones. Tetrahedron Lett..

[cit272] Yurchenko A. G., Veroshchenko A. T., Stepanov F. N. (1970). Photoisomerization of 3, 7-dimethylenebicyclo [3.3.1] nonane. Zh. Org. Khim..

[cit273] Averina N. V., Borisova G. S., Borisenko A. A., Zefirov N. S. (2001). Photochemistry of 3, 7-Bis(arylmethylene)bicyclo[3.3.1]nonane Derivatives. Russ. J. Org. Chem..

[cit274] Butkus E. P., Zilinskas A. J., Kadziauskas P. P., Kubilius R. R. (1997). Russ. Chem. Bull..

[cit275] Butkus E., Kubilius R., Stoncius S., ilinskas A. Z. (1999). ntramolecular ring closure via ether bond in reaction of α, α′-halogeno bicyclo [3.3.1] nonanediones under basic conditions. *J. Chem. Soc., Perkin Trans. 1*.

[cit276] Beckmann E., Bahr N., Cullmann O., Yang F., Kegel M., Vögtle M., Exner K., Keller M., Knothe L., Prinzbach H. (2003). Proximate,“Parallel-In-Plane” Preoriented Bis (diazenes)− In-Plane Delocalized Bis (homoconjugated) 4N/5 (6) e Anions. Eur. J. Org. Chem..

[cit277] Takeuchi K., Ohga Y., Yoshida M., Ikai K., Shibata T., Kato M., Tsugeno A. (1997). Solvolyses of 2-Oxo Bridgehead Compounds: A Critical Examination of π-Conjugative Stabilization of α-Carbonyl Carbocations. J. Org. Chem..

[cit278] Takeuchi K., Ohga Y., Tokunaga K., Tsugeno A. (1996). Formation of a propellanone in the solvolysis of a 2-oxo bicyclic bridgehead compound. Tetrahedron Lett..

[cit279] Tokunaga K., Tachibana S., Okazaki T., Ohga Y., Kitagawa T., Takeuchi K. (2000). Structural factors for the formation of propellane-type products in the solvolysis of bicyclic bridgehead compounds. J. Org. Chem..

[cit280] Baranova T. Y., Zefirova O. N., Averina N. V., Boyarskikh V. V., Borisova G. S., Zyk N. V., Zefirov N. S. (2007). Russ. J. Org. Chem..

[cit281] Trudell M. L., Cook J. M. (1989). Total synthesis of (.+-.)-suaveoline. J. Am. Chem. Soc..

[cit282] Fu X., Cook J. M. (1993). General approach for the synthesis of ajmaline-related alkaloids. Enantiospecific total synthesis of (-)-suaveoline, (-)-raumacline, and (-)-Nb-methylraumacline. J. Org. Chem..

[cit283] Bailey P. D., Morgan K. M., Smith D. I., Vernon J. M. (2000). New asymmetric routes to ajmaline and suaveoline indole alkaloids. J. Chem. Soc., Perkin Trans. 1.

[cit284] Bailey P. D., Morgan K. M., Smith D. I. (2000). The total synthesis of (−)-suaveoline. J. Chem. Soc., Perkin Trans. 1.

[cit285] Baranova T. Yu., Zefirova O. N., Averina N. V., Boyarskikh V. V., Borisova G. S., Zyk N. V., Zefirov N. S. (2007). Synthetic approach to preparation of indole derivatives fused with a bicyclo [3.3.1] nonane framework. Russ. J. Org. Chem..

[cit286] Butkus E., Berg U., Malinausiene J., Sandstrom J. (2000). Synthesis and Chiroptical Properties of Methanocycloocta[b]indoles. J. Org. Chem..

[cit287] Klusacek H., Musso H., Asterane V. (1970). Studien in der Tricyclo[3.3.1.02.8]nonan-Reihe. Chem. Ber..

[cit288] Kadziauskas P., Butkus E., Vasiulyte J., Averina N. V., Zefirov N. S. (1979). Synthesis of indoles condensed with a bicyclo [3.3.1]nonane skeleton. Chem. Heterocycl. Compd..

[cit289] Butkus E., Berg U., Malinauskieneù J., Sandstrom J. (2000). Synthesis and Chiroptical Properties of Methanocycloocta[b]indoles. J. Org. Chem..

[cit290] Berg U., Butkus E., Stončius A. (1995). Stereochemistry of the reduction of bicyclo[3.3.1]nonane-2,9-dione by complex hydrides. J. Chem. Soc., Perkin Trans. 2.

[cit291] Butkus E., Malinauskiene J., Stoncius S. (2003). Synthesis, chiroptical properties and absolute configuration of spiro [1,3-benzodioxole-methanocyclooct[b]indole]. Org. Biomol. Chem..

[cit292] Wang C.-S. (1970). Structure and chemistry of 4-hydroxy-6-methyl-2-pyridone. J. Heterocycl. Chem..

[cit293] Bisagni E., Ducrocq C., Civier A. (1976). Aza-indoles—IV: Méthode générale de synthèse des aza-5 indoles fonctionnalisés sur leur sommet 4 et de derivés polycycliques apparentés. Tetrahedron.

[cit294] Stoncius S., Butkus E., Zÿilinskas A., Larsson K., Ohrstrom L., Berg U., Warnmark K. (2004). Design and Synthesis of a C2-Symmetric Self-Complementary Hydrogen-Bonding Cleft Molecule Based on the Bicyclo[3.3.1]nonane and 4-Oxo-5-azaindole Framework. Formation of Channels and Inclusion Complexes in the Solid State. J. Org. Chem..

[cit295] Stoncius S., Orentas E., Butkus E., Ohrstrom L., Wendt O. F., Warnmark K. (2006). An Approach to Helical Tubular Self-Aggregation Using C2-Symmetric Self-Complementary Hydrogen-Bonding Cavity Molecules. J. Am. Chem. Soc..

[cit296] Labanauskas L., Zilinskas A., Visniakova S., Urbelis G., Gedrimaite O., Rozenbergas R., Podgursky A. (2008). Synthesis of bicyclo[3.3.1]nonane derivatives containing fused heterocyclic rings. ARKIVOC.

[cit297] Camps P., El Achab R., Gorbig D. M., Morral J., Munoz-Torrero D., Badia A., Banos J. E., Vivas N. M., Barril X., Orozco M., Luque F. J. (1999). Synthesis, in Vitro Pharmacology, and Molecular Modeling of Very Potent Tacrine−Huperzine A Hybrids as Acetylcholinesterase Inhibitors of Potential Interest for the Treatment of Alzheimer's Disease. J. Med. Chem..

[cit298] Badia A., Banos J. E., Camps P., Contreras J., Gorbig D. M., Munoz-Torrero D., Simon M., Vivas N. M. (1998). Synthesis and evaluation of tacrine–Huperzine a hybrids as acetylcholinesterase inhibitors of potential interest for the treatment of alzheimer's disease. Bioorg. Med. Chem..

[cit299] Ronco C., Sorin G., Nachon F., Foucault R., Jean L., Romieu A., Renard P.-Y. (2009). Synthesis and structure–activity relationship of Huprine derivatives as human acetylcholinesterase inhibitors. Bioorg. Med. Chem..

[cit300] Ronco C., Jean L., Renard P.-Y. (2010). Improved synthetic pathway for the derivatization of huprin. Tetrahedron.

[cit301] Pike A. C., Brzozowski A. M., Hubbard R. E., Bonn T., Thorsell A. G., Engstrom O., Ljunggren J., Gustafsson J., Carlquist M. (1999). Structure of the ligand-binding domain of oestrogen receptor beta in the presence of a partial agonist and a full antagonist. EMBO J..

[cit302] Coe P. L., Scriven C. E. (1986). Crossed coupling of functionalised ketones by low valent titanium (the McMurry reaction): a new stereoselective synthesis of tamoxifen. J. Chem. Soc., Perkin Trans. 1.

[cit303] McMurry J. E., Krepski L. R. (1976). Synthesis of unsymmetrical olefins by titanium(0) induced mixed carbonyl coupling. Some comments on the mechanism of the pinacol reaction. J. Org. Chem..

[cit304] Muthyala R. S., Sheng S., Carlson K. E., Katzenellenbogen B. S., Katzenellenbogen J. A. (2003). Bridged Bicyclic Cores Containing a 1,1-Diarylethylene Motif Are High-Affinity Subtype-Selective Ligands for the Estrogen Receptor. J. Med. Chem..

[cit305] Kieser K. J., Kim D. W., Carlson K. E., Katzenellenbogen B. S., Katzenellenbogen J. A. (2010). Characterization of the Pharmacophore Properties of Novel Selective Estrogen Receptor Downregulators (SERDs). J. Med. Chem..

[cit306] Liaw D.-J., Liaw B.-Y., Chung C.-Y. (1999). Synthesis and characterization of new adamantane-type cardo polyamides. Acta Polym..

[cit307] Muthyala R. S., Carlson K. E., Katzenellenbogen J. A. (2003). Exploration of the bicyclo[3.3.1]nonane system as a template for the development of new ligands for the estrogen receptor. Bioorg. Med. Chem. Lett..

[cit308] Uemura M., Isobe K., Hayashi Y. (1985). (η6-arene) tricarbonylchromium complex in organic synthesis: stereoselective alkylation at benzylic position of (η^6^-Arene) tricarbonylchromium. Tetrahedron Lett..

[cit309] Liaw D.-J., Liaw B.-Y., Chung C.-Y. (1999). Synthesis and characterization of new adamantane-type cardo polyamides. Acta Polym..

[cit310] Wu J., Li D., Wu H., Sun L., Dai W.-M. (2006). Microwave-assisted regioselective olefinations of cyclic mono- and di-ketones with a stabilized phosphorus ylid. Tetrahedron.

[cit311] Strenge A., Rademacher P. (1999). Spectroscopic and Theoretical Investigations of Monocyclic Dioximes and Dimethoximes with Six-, Eight-, and Ten-Membered Ring. Eur. J. Org. Chem..

[cit312] Camps P., Muñoz-Torrero D., Muñoz-Torrero V. (1995). Unusual oxidation of bridgehead polycyclic 1, 2-diamines and 2-aminoalcohols with dimethyldioxirane: Formation of dioximes and monooximes by cleavage of the central carbon-carbon bond. Tetrahedron Lett..

[cit313] Camps P., Muñoz-Torrero D. (1994). Alternative synthesis of bridgehead polycyclic 1,2-diamines and 2-aminoalcohols from di- and mono-oximes of some bicyclic diketones: Highly improved synthesis of tricycle[3.3.1.0]nonane-3,7-diamine. Tetrahedron Lett..

[cit314] Tosa N., Bende A., Varga R. A., Terec A., Bratu I., Grosu I. (2009). H-Bond-Driven Supramolecular Architectures of the Syn and Anti Isomers of the Dioxime of Bicyclo[3.3.1]nonane-3,7-dione. J. Org. Chem..

[cit315] Otomaru Y., Kina A., Shintani R., Hayashi T. (2005). C2-Symmetric bicyclo[3.3.1]nona-2,6-diene and bicyclo[3.3.2]deca-2,6-diene: new chiral diene ligands based on the 1,5-cyclooctadiene framework. Tetrahedron: Asymmetry.

[cit316] Shintani R., Ichikawa Y., Takatsu K., Chen F.-X., Hayashi T. (2009). Tuning the Chiral Environment of C2-Symmetric Diene Ligands: Development of 3,7-Disubstituted Bicyclo[3.3.1]nona-2,6-dienes. J. Org. Chem..

[cit317] Otomaru Y., Tokunaga N., Shintani R., Hayashi T. (2005). C2-Symmetric Bicyclo[3.3.1]nonadiene as a Chiral Ligand for Rhodium-Catalyzed Asymmetric Arylation of N-(4-Nitrobenzenesulfonyl)arylimines. Org. Lett..

[cit318] Quast H., Witzel M., Peters E.-M., Peters K., von Schnering H. G. (1995). Syntheses and structures of 3,7-substituted barbaralanes. Liebigs Ann..

[cit319] Wallentin C.-J., Orentas E., Johnson M. T., Bathori N. B., Butkus E., Wendt O. F., Warnmark K., Ohrstrom L. (2012). Synthetic and crystallographic studies of bicyclo[3.3.1]nonane derivatives: from strong to weak hydrogen bonds and the stereochemistry of network formation. CrystEngComm.

[cit320] Orentas E., Bagdziunas G., Berg U., Zilinskas A., Butkus E. (2007). Enantiospecific Synthesis and Chiroptical Properties of Bicyclic Enones. Eur. J. Org. Chem..

[cit321] Ahmad N. M., Rodeschini V., Simpkins N. S., Ward S. E., Wilson C. (2007). Synthetic studies towards garsubellin A: synthesis of model systems and potential mimics by regioselective lithiation of bicyclo[3.3.1]nonane-2,4,9-trione derivatives from catechinic acid. Org. Biomol. Chem..

[cit322] Giblin G. M. P., Kirk D. T., Mitchell L., Simpkins N. S. (2003). Bridgehead Enolates: Substitution and Asymmetric Desymmetrization of Small Bridged Carbonyl Compounds by Lithium Amide Bases. Org. Lett..

[cit323] Fernfindez M. J., Huertas R. M., Galvez E., Server-Carrio J., Martinez-Ripoll M., Bellanato J. (1995). Synthesis and structural and conformational study of some amides derived from 3,7-dimethyl-3,7-diazabicyclo[3.3.1]nonan-9-amine. J. Mol. Struct..

[cit324] Kolhatkar R., Cook C. D., Ghorai S. K., Deschamps J., Beardsley P. M., Reith M. E., Dutta A. K. (2004). Further Structurally Constrained Analogues of cis-(6-Benzhydrylpiperidin-3-yl)benzylamine with Elucidation of Bioactive Conformation: Discovery of 1,4-Diazabicyclo[3.3.1]nonane Derivatives and Evaluation of Their Biological Properties for the Monoamine Transporters. J. Med. Chem..

[cit325] Bollinger S., H€ubner H., Heinemann F. W., Meyer K., Gmeiner P. (2010). Novel Pyridylmethylamines as Highly Selective 5-HT1A Superagonists. J. Med. Chem..

[cit326] Kiryukhin M. V., Nurieva E. V., Shishov D. V., Nuriev V. N., Zyk N. V., Zefirov N. S., Zefirova O. N. (2007). Seriya 2: Khimiya.

[cit327] Mayr M., Bataille C. J. R., Gosiewska S., Raskatov J. A., Brown J. M. (2008). Synthesis and rhodium complexation of enantiomerically enriched bicyclo [3.3. 1] nona-2, 6-diene. Tetrahedron: Asymmetry.

[cit328] Grossman R. B., Tsai J. C., Davis W. M., Gutierrez A., Buchwald S. L. (1994). Synthesis and structure of a C2-symmetric, doubly bridged ansa-titanocene complex. Organometallics.

[cit329] Pimenov A. A., Makarova N. V., Zemtsova M. N., Moiseev I. K. (2001). Synthesis of α, β- Unsaturated Ketones on the Basis of Bicyclo [3.3.1] nonane-2, 6-dione. Russ. J. Org. Chem..

[cit330] Smirnov G. A., Sevost'yanova V. V., Klimova T. A. (1981). Izv. Akad. Nauk, Ser. Khim..

[cit331] Krayushkin M. M., Sevostyanova V. V., Yarovenko V. N., Zavarzin I. V. (2009). Reactions of α,β-unsaturated ketones of the bicyclo[3.3.1]nonane series with nitro alkanes. Russ. Chem. Bull. Int. Ed..

[cit332] Ronco C., Jean L., Outaabout H., Renard P. Y. (2011). Palladium-Catalyzed Preparation of N-Alkylated Tacrine and Huprine Compounds. Eur. J. Org. Chem..

[cit333] Tkachenko B. A., Fokina N. A., Chernish L. V., Dahl J. E. P., Liu S., Carlson R. M. K., Fokin A. A., Schreiner P. R. (2006). Functionalized Nanodiamonds Part 3: Thiolation of Tertiary/Bridgehead Alcohols. Org. Lett..

[cit334] Shen X., Ting C. P., Xu G., Maimone T. J. (2020). Programmable meroterpene synthesis. Nat. Commun..

[cit335] Phang Y., Wang X., Lu Y., Fu W., Zheng C., Xu H. (2020). Bicyclic polyprenylated acylphloroglucinols and their derivatives: structural modification, structure-activity relationship, biological activity and mechanism of action. Eur. J. Med. Chem..

[cit336] Wolf R. J., Hilger R. A., Hoheisel J. D., Warner J., Holtrup F. (2013). In *Vivo* Activity and Pharmacokinetics of Nemorosone on Pancreatic Cancer Xenografts. PLoS One.

[cit337] Frion-Herrera Y., Gabbia D., Scaffidi M., Zagni L., Cuesta-Rubio O., De Martin S., Carrara M. (2020). The Cuban Propolis Component Nemorosone Inhibits Proliferation and Metastatic Properties of Human Colorectal Cancer Cells. Int. J. Mol. Sci..

[cit338] Wu H. M., Li Y. M. (2017). In vitro antitumor activity of guttiferone-A in human breast cancer cells is mediated via apoptosis, mitochondrial mediated oxidative stress and reactive oxygen species production. J. BUON.

[cit339] Pardo-Andreu G. L., Nunez-Figueredo Y., Tudella V. G., Cuesta-Rubio O., Rodrigues F. P., Pestana C. R., Uyemura S. A., Leopoldino A. M., Alberici L. C., Curti C. (2011). The anti-cancer agent guttiferone-A permeabilizes mitochondrial membrane: Ensuing energetic and oxidative stress implications. Toxicol. Appl. Pharmacol..

[cit340] Kopytko P., Piotrowska K., Janisiak J., Tarnowski M. (2021). Garcinol—A Natural Histone Acetyltransferase Inhibitor and New Anti-Cancer Epigenetic Drug. Int. J. Mol. Sci..

[cit341] Son S. I., Su D., Ho T. T., Lin H. (2020). Garcinol Is an HDAC11 Inhibitor. ACS Chem. Biol..

[cit342] Aggarwal V., Singh Tuli H., Kaur J., Aggarwal D., Parashar G., Chaturvedi Parashar N., Kulkarni S., Kaur G., Sak K., Kumar M., Ahn K. S. (2020). Garcinol Exhibits Anti-Neoplastic Effects by Targeting Diverse Oncogenic Factors in Tumor Cells. Biomedicines.

[cit343] Lim S. H., Lee H. S., Lee C. H., Choi C.-I. (2021). Pharmacological Activity of Garcinia indica (Kokum): An Updated Review. Pharmaceuticals.

[cit344] Shen K., Xie J., Wang H., Zhang H., Yu M., Lu F., Tan H., Xu H. (2015). Mol. Cancer Ther..

[cit345] Sui H., Tan H., Fu J., Song Q., Jia R., Han L., Lv Y., Zhang H., Zheng D., Dong L. (2020). The active fraction of Garcinia yunnanensis suppresses the progression of colorectal carcinoma by interfering with tumorassociated macrophage-associated M2 macrophage polarization in vivo and in vitro. FASEB J..

[cit346] Shen K., Xi Z., Xie J., Wang H., Xie C., Lee C. S., Fahey P., Dong Q., Xu H. (2016). Guttiferone K suppresses cell motility and metastasis of hepatocellular carcinoma by restoring aberrantly reduced profiling. Oncotarget.

[cit347] Reis F. H., Pardo-Andreu G. L., Nunez-Figueredo Y., Cuesta-Rubio O., Martin-Prida J., Uyemura S. A., Curti C., Alberici L. C. (2014). Clusianone, a naturally occurring nemorosone regioisomer, uncouples rat liver mitochondria and induces HepG2 cell death. Chem.-Biol. Interact..

[cit348] Taylor W. F., Yanez M., Moghadam S. E., Moridi Farimani M., Soroury S., Ebrahimi S. N., Tabefam M., Jabbarzadeh E. (2019). 7-epi-Clusianone, a Multi-Targeting Natural Product with Potential Chemotherapeutic, Immune-Modulating, and Anti-Angiogenic Properties. Molecules.

[cit349] Zhu H., Yang Y.-N., Xu K., Xie J., Feng Z.-M., Jiang J.-S., Zhang P.-C. (2013). Org. Biomol. Chem..

[cit350] Manimaran M., Ganapathi A., Balasankar T. (2015). Synthesis, Spectral, Anti-Liver Cancer and Free Radical Scavenging Activity of New Azabicyclic Thienoyl Hydrazone Derivatives. Open J. Med. Chem..

[cit351] Garrison J. K. F., Jonsson S., Valgeirsson J. (2004). Synthesis and antitumor activity of bicyclo[3.3.1]nonenol derivatives. Bioorg. Med. Chem..

